# *Clostridium autoethanogenum* isopropanol production *via* native plasmid pCA replicon

**DOI:** 10.3389/fbioe.2022.932363

**Published:** 2022-08-05

**Authors:** Robert Nogle, Shilpa Nagaraju, Sagar M. Utturkar, Richard J. Giannone, Vinicio Reynoso, Ching Leang, Robert L. Hettich, Wayne P. Mitchell, Sean D. Simpson, Michael C. Jewett, Michael Köpke, Steven D. Brown

**Affiliations:** ^1^ LanzaTech Inc., Skokie, IL, United States; ^2^ Center for Cancer Research, Purdue University, West Lafayette, IN, United States; ^3^ Oak Ridge National Laboratory, Oak Ridge, TN, United States; ^4^ Department of Chemical and Biological Engineering, Northwestern University, Evanston, IL, United States; ^5^ Center for Synthetic Biology, Northwestern University, Evanston, IL, United States; ^6^ Simpson Querrey Institute, Northwestern University, Evanston, IL, United States; ^7^ Chemistry of Life Processes Institute, Northwestern University, Evanston, IL, United States; ^8^ Robert H. Lurie Comprehensive Cancer Center, Northwestern University, Evanston, IL, United States

**Keywords:** acetogen, syngas, genome, clostridia, biofuel, ethanol, synthetic biology

## Abstract

*Clostridium autoethanogenum* is a model gas-fermenting acetogen for commercial ethanol production. It is also a platform organism being developed for the carbon-negative production of acetone and isopropanol by gas fermentation. We have assembled a 5.5 kb pCA plasmid for type strain DSM10061 (JA1-1) using three genome sequence datasets. pCA is predicted to encode seven open-reading frames and estimated to be a low-copy number plasmid present at approximately 12 copies per chromosome. RNA-seq analyses indicate that pCA genes are transcribed at low levels and two proteins, CAETHG_05090 (putative replication protein) and CAETHG_05115 (hypothetical, a possible Mob protein), were detected at low levels during batch gas fermentations. Thiolase (*thlA*), CoA-transferase (*ctfAB*), and acetoacetate decarboxylase (*adc*) genes were introduced into a vector for isopropanol production in *C. autoethanogenum* using the native plasmid origin of replication. The availability of the pCA sequence will facilitate studies into its physiological role and could form the basis for genetic tool optimization.

## Introduction

*Clostridium autoethanogenum* is a model acetogen that uses a wide range of CO, CO_2_, and H_2_ gas mixes for carbon and energy sources *via* the Wood–Ljungdahl pathway (Marcellin et al., 2016). *C. autoethanogenum* has been used to investigate the fundamentals of electron bifurcation and acetogen bioenergetics (Mock et al., 2015). A genetic toolbox exists that includes heterologous expressions *via* plasmid and chromosomal integration, gene deletion, CRISPR systems, validated genetic parts, and codon adaptation algorithms (Joseph et al., 2018; Fackler et al., 2021). The *C. autoethanogenum* DSM10061 restriction system has been characterized as part of a study to improve DNA transfer efficiencies into *Clostridium* spp. (Woods et al., 2019). Furthermore, plasmid DNA synthetic biology toolkit developments and the application of plasmids for clostridia genetics have been described recently (Joseph et al., 2018). *C. autoethanogenum* is used as a biocatalyst to produce ethanol in commercial scale gas fermentations (Fackler et al., 2021). Recently, the bacterium has been engineered through a series of gene deletions and *via* chromosomal heterologous expressions to generate strains for acetone or isopropanol production at commercially relevant production rates (Liew et al., 2022).

Because of its industrial relevance and status as an acetogen model organism, there has been significant efforts generating a highly polished and manually annotated genome sequence. The initial wild-type *C. autoethanogenum* strain JA1-1 (DSM10061) genome is a draft assembly that consisted of 100 contigs (Bruno-Barcena et al., 2013). The application of long-read sequencing technology facilitated the generation of a closed chromosome sequence for *C. autoethanogenum* DSM10061 (Brown et al., 2014; Utturkar et al., 2015). The DSM10061 genome sequence underwent further polishing *via* Illumina MiSeq and Sanger sequencing and manual annotation updates were applied (Humphreys et al., 2015). In an earlier survey, conducted before *C. autoethanogenum* was isolated, plasmid bands ranging from 3 to >100 kb were detected in 26 strains representing 21 species across the genus *Clostridium* (Lee et al., 1987). In this study, we identify a native 5,499 bp plasmid present in the raw DNA sequence data from three prior genome-sequencing studies. We characterize the plasmid, expression of encoded proteins and demonstrate its utility for synthetic biology through the heterologous expression of genes encoding thiolase, CoA transferase subunits A and B, and acetoacetate decarboxylase for isopropanol production.

## Results

### Plasmid DNA assembly and estimated copy number

**FIGURE 1:**
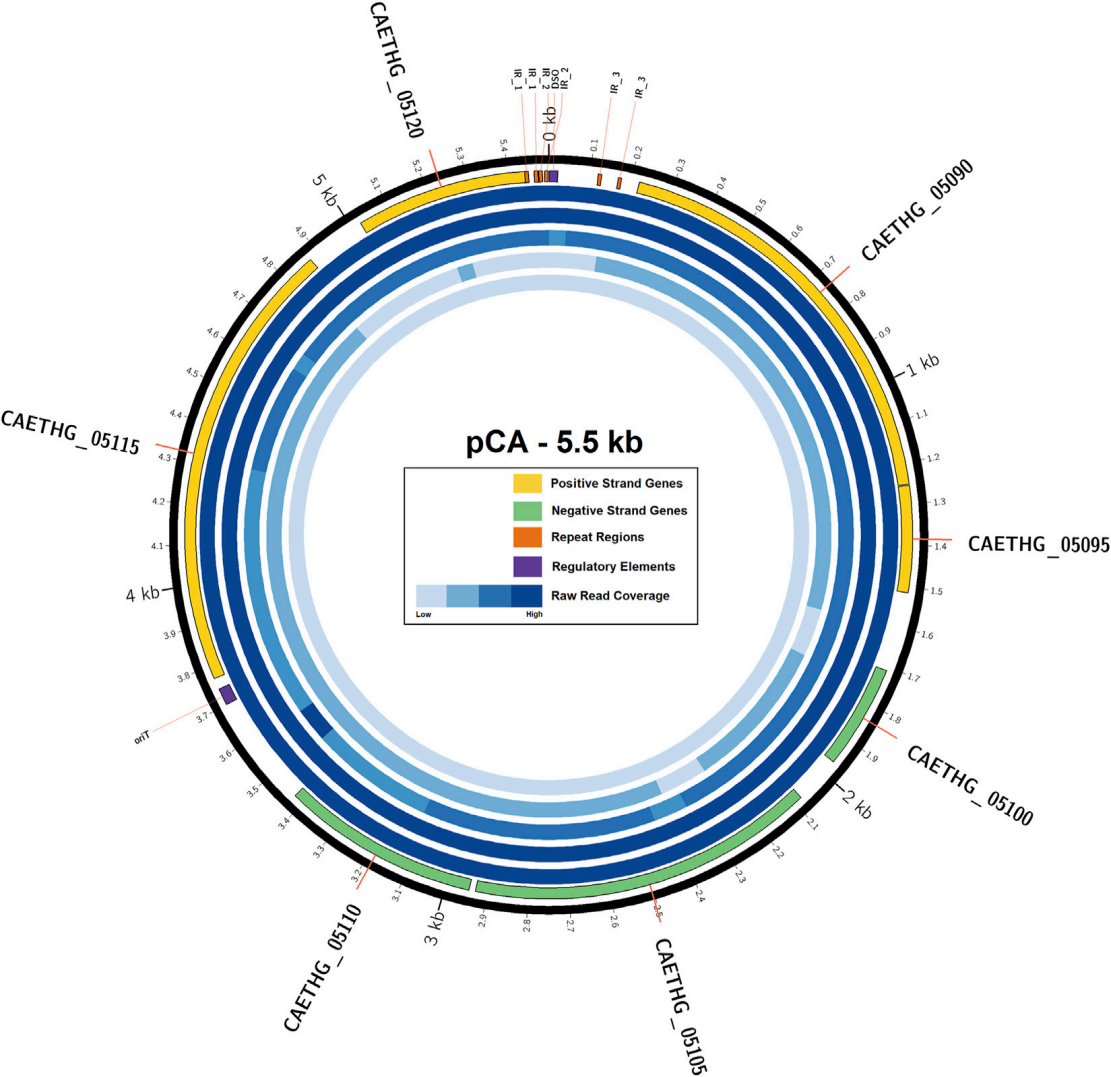
Plasmid pCA, its genes, and sequence coverage. The outermost ring (black) represents the circular pCA plasmid sequence (5.49 KB). The next inner ring represents the seven plasmid genes encoded on positive (yellow) and negative (green) strands, regulatory elements (purple), and repeat regions (orange). The next five rings represent the raw-read coverage from Illumina, 454, Ion Torrent and PacBio technology, respectively. Feature coordinates are also available in the GenBank submission.

**FIGURE 2:**
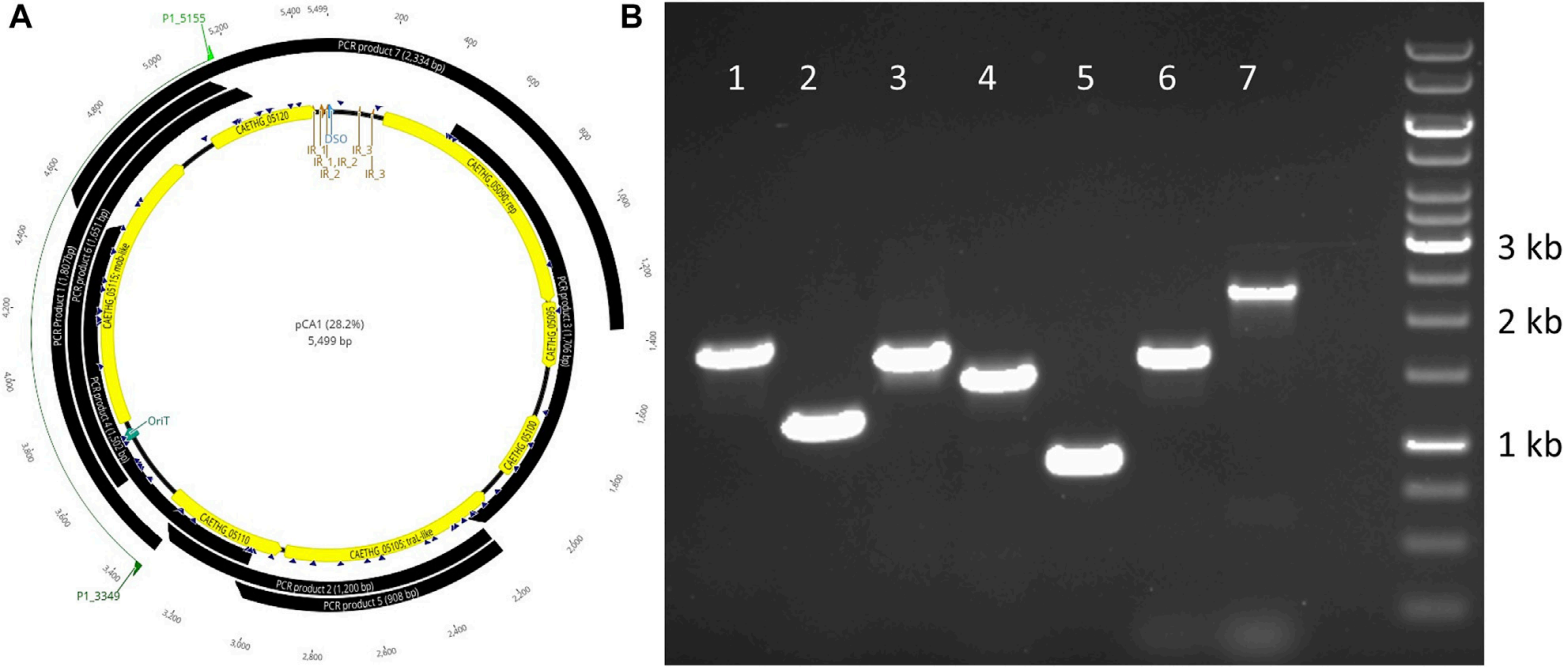
Verification of the plasmid pCA sequence *via* PCR and agarose electrophoresis. PCR product locations relative to the gene locations on pCA are shown **(A)** and associated amplicons for respective products 1–7 **(B)**.

*C. autoethanogenum* pCA is a novel 5,499 bp circular plasmid identified in strain DSM10061. It is predicted to encode seven open reading frames (ORFs) and have a 28.2% GC content (Figure 1). Plasmid pCA was assembled from a previously published *C. autoethanogenum* DSM10061 Illumina dataset using plasmidSPAdes (Antipov et al., 2016), which was specifically developed to extract and assemble plasmid data from whole genome-sequencing projects. Previously reported Illumina data (Utturkar et al., 2015) were assembled into two plasmidSPAde sequence outputs with different coverage values (×132 and ×672), different GC contents (28 and 44%), and different lengths (5,598 bp and 5,626 bp). Both sequences appeared to be circular based on the initial dot-plot analysis. Data from an independent chromosome mapping study (Humphreys et al., 2015) underwent plasmidSPAdes assembly as part of this study, which also produced two short similar sequence outputs (5,626 and 5,516 bp). After annotation, the higher coverage and G + C content sequences were determined to be phiX control DNA (G + C 45 %) and were thus discarded. The Circlator software (Hunt et al., 2015) did not assemble the pCA ends, so they were joined manually (see Methods). The number of reads mapped to the assembled plasmid sequence for each technology were 73,571(Illumina), 7,945 (454), 5,705 (Ion-Torrent), and 72 (PacBio). The Illumina and PacBio datasets were sequenced to higher coverages (>100 x) relative to 454 and ion torrent datasets which had lower coverages (<50 x) (Utturkar et al., 2015). Illumina coverage for plasmid pCA is estimated to be approximately ×1,994 (73,571 reads ×149 bp or 10,962,079 bases/5.499 kb), or 15.8 plasmid copies per chromosome (based on reported ×126 chromosome coverage). The estimated number of plasmid per chromosome sequence for the second study is ∼10.4 copies (×2,272.5/×219.4). Genome data from a chemostat study (Martin et al., 2016) indicates ∼11.7 copies (×2,271/×193.6). Taken together, the mean plasmid number is estimated to be 12.6 (+/−2.8 S.D.) copies per cell, indicating that pCA is a low copy number plasmid. The presence of a circular pCA plasmid was verified through a series of overlapping PCR reactions (Figure 2).

### Sequence similarity

A BLASTN analysis of the pCA sequence showed the highest similarity scores to the *Clostridium tyrobutyricum* strain Cirm BIA 2237 chromosome (GenBank: CP038158.1) matching several regions [709/905 nt (78%) and 129/157 nt (82%)], which encode putative hypothetical proteins followed by plasmid sequences in other clostridia. Each of the seven predicted pCA proteins (CAETHG_05090-50120) have identical matches to previously described *C. autoethanogenum* DSM10061 proteins (NCBI accessions; OVY48499.1, OVY48498.1, OVY48497.1, OVY48503.1, OVY48502.1, OVY48501.1, and OVY48500.1, respectively) (Martin et al., 2016). CAETHG_05100, 05, 10, and 20, begin with ATG start sites, CAETHG_05090 has a TTG start site (Leu) and CAETHG_05095 and CAETHG_05115 begin with a GTG start site (Val).

**FIGURE 3:**
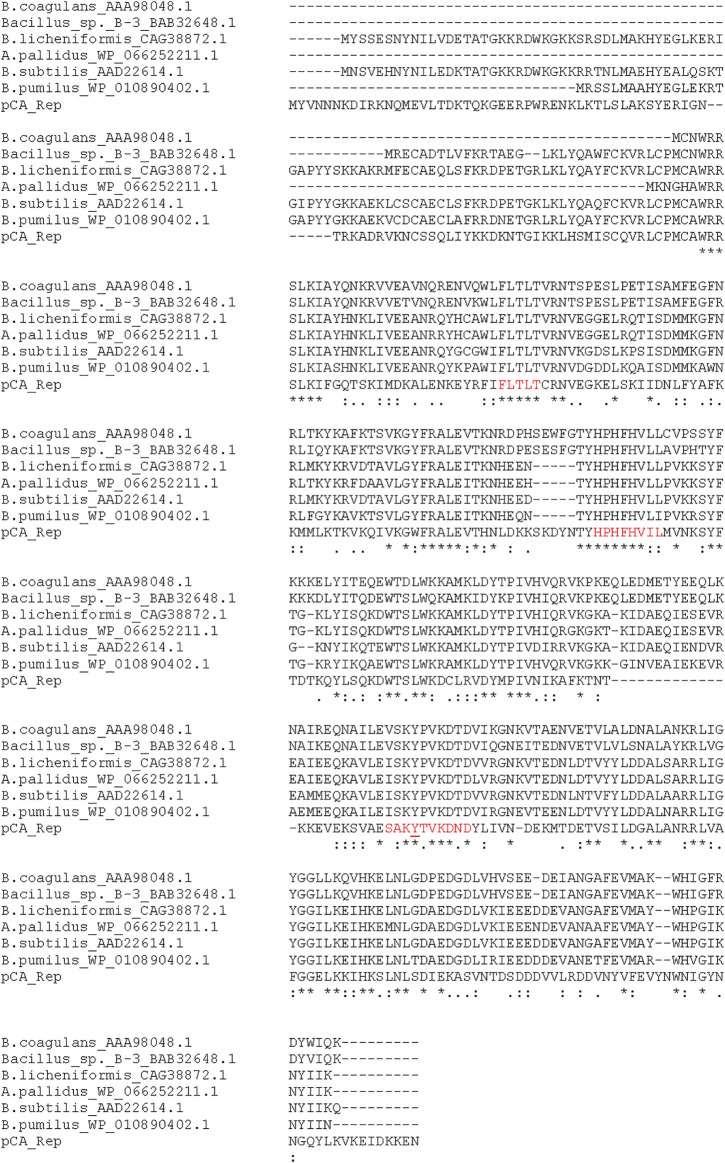
pUB110/pMV158-like replication protein in pCA. Replication protein sequences from *Bacillus* species and Rep protein from pCA (pCA-Rep) were aligned using ClustalW. The conserved regions are highlighted in red and the putative active site tyrosine residue is underlined.

Protein CAETHG_05090 is the 344 aa putative plasmid replication protein, which has a 48–61% identity to plasmid borne replication proteins from Clostridia species and 40–46% identity to other Gram-positive bacteria and particularly from *Bacillus* species (Figure 3). The CAETHG_05090 replication protein shares similarity with the pUB110 plasmid family replication proteins, including 100% identity across several conserved regions and an active site tyrosine residue. An 22 bp sequence in the 5′ region of CAETHG_05090 is a possible double strand origin (DSO) of replication (*ori*) for the leading strand replication based on sequence similarity, with inverted repeats flanking it (Supplementary Figure S1). A possible minus single strand *ori* (SSO) for lagging strand replication is identified, although less sequence conservation is observed (Supplementary Figure S2), along with a region for a possible origin of transfer (*oriT*) (Figure 1). CAETHG_05095 and CAETHG_05100 are putative hypothetical proteins. ORF CAETHG_05105 is predicted to encode a 282 aa protein containing transmembrane domains and having 27% identity with the *Lactobacillus* spp. conjugal transfer pilus assembly protein TraL. CAETHG_05110 is predicted to encode a 160 aa protein with a signal peptide. Open reading frame CAETHG_05115 is predicted to encode a hypothetical protein that shares 35–40% identity with mobility (MOB) proteins from pUB110, pMV158, and *C. saccharoperbutylacetonicum* shuttle vector pNAK1. Sequences upstream of pCA_06 (3,700–3,741 bases) share >90% identity with the core *oriT*s from pUB110/pMV158 that consist of PalD inverted repeats and a recombination site (Supplementary Figure S3) and this may form a part of the pCA *oriT*. CAETHG_05120 encodes a putative 141 aa hypothetical protein.

### Gene expression analysis

**FIGURE 4:**
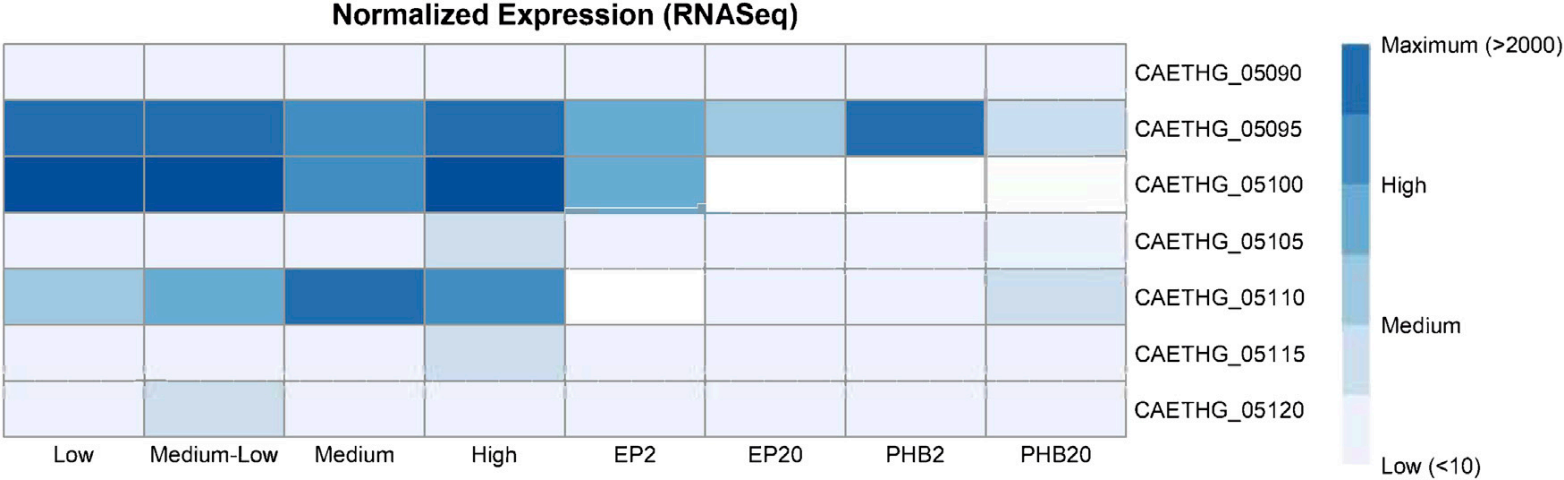
Expression of plasmid genes in the published RNASeq data. Normalized (FPKM) and mean expression of the plasmid genes corresponding to varying biomass concentration (low, medium-low, medium, and high), and recombinant poly-3-hydroxybutyrate (PHB) and empty plasmid (EP) strains grown using syngas (20% H_2,_ denoted in sample label by 20) or steel mill off-gas (2% H_2_ denoted in sample label by 2). See original articles for details (Table 1).

To examine the pCA gene expression, we re-analyzed the RNA-seq data from a study on recombinant poly-3-hydroxybutyrate (PHB)-producing *C. autoethanogenum* and control strains (de Souza Pinto Lemgruber et al., 2019) and another study with profiles for the steady-state chemostat cultures grown on syngas (Valgepea et al., 2017). DNA sequence read alignment rates to pCA genes were low, measuring between 0.5–0.7% and 0.9–1.3% across both datasets. Transcripts for the CAETHG_05090 putative replication gene were detected at low levels for most samples (17/20) (Figure 4). CAETHG_05095 had the highest levels of expression for plasmid genes, but its detection was variable (detected in 11 of 20 samples), followed by CAETHG_05100. Normalized FPKM values indicate CAETHG_05105 (*traL-like)* was expressed in all *C. autoethanogenum* strain DSM 19630 samples and in five of eight samples for PHB-producing strains. CAETHG_05115 was detected at a low level in the PHB study (7 of 8), and infrequently (3 of 12) in the other study. CAETHG_05110 and CAETHG_05120 were at or below detection limit levels.

In an initial assessment to determine whether plasmid genes ultimately produced detectible protein products, batch-culture grown *C. autoethanogenum* was sampled at either lag- or early log-phases (*n* = 3) and processed for LC-MS/MS-based proteomic measurements. Across all samples and both growth states, a total of 13,693 peptide analytes (FDR <0.01) were identified. These high-confidence peptide identifications mapped to 1,892 proteins, with 1,647 at a protein-level FDR ≤0.05, and 1492 at FDR ≤0.02. Two plasmid proteins (CAETHG_05090 and CAETHG_05115) were detected at approximately the 28th and 22nd percentiles within the entire proteome (out of 1647), respectively, when sorted by decreasing the median abundance. This suggests that their presence is at a low level relative within the proteome. We present CAETHG_05090 and CAETHG_05115 proteomics data, along with the twenty most highly expressed proteins identified for the strain (Supplementary Table S1). As expected, Wood–Ljungdahl pathway proteins were among the most abundant. Across the two growth conditions, a total of 97 proteins exhibited statistically significant differences in abundance (Benjamini-Hochberg corrected *p*-value ≤ 0.05; |log2 difference| > 1), with 25 proteins higher during the lag-phase and 72 higher during the log-phase (Supplementary Table S2).

### Utility of the plasmid pCA replicon for isopropanol production by gas fermentation

**FIGURE 5:**
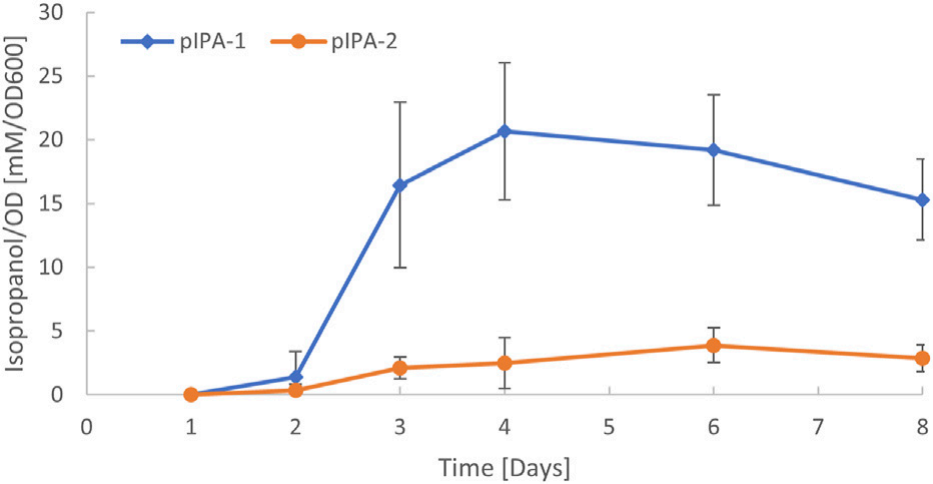
*C. autoethanogenum* isopropanol production *via* heterologous expression using two different origins of replication. Plasmids pIPA-1 and pIPA-2 contain the same acetone production genes, promoter sequences, and differ in origins from pBP1 (Woods et al., 2019) or from native plasmid pCA, respectively. Plasmid details are available in supplemental GenBank files. Acetone is converted to isopropanol via a native chromosomal secondary alcohol dehydrogenase, as previously described (Köpke et al., 2014).

To test the functionality of the native plasmid origin of replication, it was amplified from *C. autoethanogenum* and used for the heterologous expressions of thiolase (*thlA*), CoA-transferase (*ctfAB*), and acetoacetate decarboxylase (*adc*) genes under control of the IPL-tet3n0 promoter (TCT​ATC​ATT​GAT​AGG​TTA​TAA​TGA​ACA​TTG​TAG​AAT​TCC​CAT​AAT​AAA​GAA​AGA​ATT​TTA​AAT​AAA​GGA​GGA​ACA​CA) (Nagaraju et al., 2016) for *C. autoethanogenum* isopropanol production [the final reduction step in the isopropanol pathway is catalyzed by a native secondary alcohol dehydrogenase (Köpke et al., 2014)]. We cloned the genes along with the identified pCA origin into the modular pMTL8X25X plasmid (Heap et al., 2009). As control, we used the same construct but with the pBP1 origin present in the original pMTL8225X plasmid. Each plasmid was transformed into *C. autoethanogenum* and the resulting strains were tested for isopropanol production during gas fermentation. When the pathway was expressed from the original pMTL80000 plasmid with the pBP1 origin of replication, approximately 33 mM of isopropanol was observed (Figure 5). We also observed isopropanol production when the pathway was expressed from a plasmid using the native pCA plasmid origin of replication. However, production levels were lower using the pCA origin, with approximately 7 mM of isopropanol produced (Figure 5), indicating a lower copy number. This demonstrates the utility of the plasmid for gene expression in *C. autoethanogenum* and how it can be used to modulate gene expression levels. It was not determined if the native plasmid was maintained or lost after the introduction of plasmid pIPA-2.

## Discussion

In this study, we identify the novel 5.49 kb *C. autoethanogenum* plasmid pCA as being present, but previously unreported in three independent Illumina genome datasets. Plasmids are common among clostridia and pCA, similar to most (Davis et al., 2005), are also likely cryptic. Cell wall-associated DNase activity has been previously attributed as an impediment to the recovery of intact clostridial plasmid DNA (Blaschek and Klacik, 1984; Roberts et al., 1986; Hussain and Purnima, 1993) and this coupled with the low copy number of pCA may have contributed to it not being characterized previously. An earlier screen for plasmid DNA in the genus *Clostridium* identified a 5.4 kb plasmid in *Clostridium aceticum* and while the size is similar (5.5 kb) to the plasmid from this study, the restriction profiles are distinct (Lee et al., 1987).

The traditional methods for plasmid sequencing involved purification of plasmid DNA, end-fragment or shot-gun sequencing, followed by gap closure using primer-walking. *De novo* assembly of short-read next-generation sequence data often leads to fragmented genome assemblies that complicate the plasmid assembly and identification (Arredondo-Alonso et al., 2017). The *C. autoethanogenum* DSM10061 chromosome was assembled as one contig using single-molecule sequencing. The assembled chromosome featured repeats, putative prophage sequence, low G + C content, and nine copies of the rRNA gene operons, all of which present challenges to short-read sequencing technologies (Brown et al., 2014). Plasmid DNA was likely excluded during the PacBio library preparation, owing to a size selection for high molecular weight genomic DNA to produce long, single molecule sequence reads, as observed previously (Margos et al., 2017). Furthermore, the hierarchical genome assembly process, when implemented with a seed read length cut-off greater than plasmid size, can result in small plasmids being lost from the main assembly (Forde et al., 2014; Koren and Phillippy, 2015). Different technologies, bioinformatics approaches, and challenges in plasmid reconstruction have been described (Hunt et al., 2015b). The *Escherichia coli* strain K-12 genome, arguably the most well-studied single organism (Riley et al., 2006), and others model organisms such as *Desulfovibrio alaskensis* G20 (Hauser et al., 2011), and *Zymomonas mobilis* ZM4, have similarly been improved over time (Yang et al., 2009, 2018). The description of plasmid pCA further improves the *C. autoethanogenum* DSM10061 genome sequence and the utility of its origin of replication was demonstrated for isopropanol production (Figure 5).

*Bacillus* species plasmids that show the most similarity to pCA are of the non-conjugative rolling-circle type that can be mobilized for conjugative transfer in the presence of conjugation machinery. They replicate by a rolling circle mechanism *via* a single strand DNA intermediate enabled by a replication protein, plus a double strand (DSO) origin of replication (*ori*) for leading strand replication and a minus single strand (SSO) *ori* for lagging strand replication. They are broadly classified into five families based on replication protein and DSO homology (Ilyina and Koonin, 1992). We have identified a putative replication protein that has conserved consensus motifs, including an active site tyrosine and conserved 18 bp DSO sequence flanked by inverted repeats. SSO sequences within a family share less homology and certain plasmids have more than one SSO (Ilyina and Koonin, 1992). pCA may replicate by a rolling circle mechanism and is potentially a non-conjugative plasmid, but this requires further study. An improved understanding of pCA replication could assist in determining whether it can coexist with other plasmids and transferability. The presence of pCA may prevent the efficient transformation of some heterologous plasmids that rely on the same replication mechanism, but replication co-existence (e.g., pCB102 of the pMTL80000 plasmids) could be useful for dual plasmid expressions similar to the pETDuet system for *E. coli* (Novy et al., 2002). The pCA copy number appears lower than with pMTL80000 plasmids (pBP1 origin), further expanding to the suite of tools to modulate the expression in clostridia.

Plasmid pCA appears to be a small, selfish DNA element with limited coding potential and expression, present at approximately 12 copies per cell, although this requires examining a greater range of conditions and the use of more quantitative methods. A proteomic analysis generated a comprehensive profile of *C. autoethanogenum* DSM10061 growing in a batch culture and under the conditions analyzed here (lag-vs. early log-phase); plasmid proteins were either detected at low levels or not at all. The physiological role of the pCA plasmid, if any, remains unclear. Any metabolic burden associated with plasmid maintenance could be relieved by generating a cured strain using strategies such as elevated growth temperature and screening procedures, targeting the replication origin through transformation of an incompatible plasmid, and potentially through the application of CRISPR technologies. The application of Cell-Free Protein Synthesis (CFPS) technologies (Silverman et al., 2020) could enable a better understanding of its elements and future systems, and biology studies may shed light on the role of this native *C. autoethanogenum* plasmid. We have shown the utility of the native plasmid origin of replication for isopropanol production. The small size of pCA (5.5 kb), low G + C content, low-copy number, and native origin of replication may facilitate its further optimization as a genetic tool.

## Methods

### Data sources and sequence analysis

DNA, RNA-seq, and chromosome sequence data were downloaded from the National Center for Biotechnology Information databases and accession numbers are provided, along with the data generated in this study (Table 1). Plasmid pCA was assembled from Illumina data using plasmidSPAdes (Antipov et al., 2016), and overlapping contig ends were resolved manually using the Geneious software (version 8.1.6), as described previously (Utturkar et al., 2017). Gene predictions and annotations were conducted using the RAST pipeline (Overbeek et al., 2014), with the Prodigal gene caller option and default settings. Sequence similarity searches were performed using NCBI BLAST and PSI-BLAST tools. Plasmids sequences for pUB110/pMV158 and from this family were retrieved from NCBI and further alignments were conducted using CLUSTALW (https://www.genome.jp/tools-bin/clustalw).

**TABLE 1:** Sequences and chromosome data sources.

Data type	NCBI BioProject/GenBank Accession/Proteomics DB	References
RNA-seq	PRJNA355951	Valgepea et al. (2017)[15]
RNA-seq	PRJNA476240	de Souza Pinto Lemgruber et al. (2019)[14]
DNA sequence	PRJNA219420	Utturkar et al. (2017)
DNA sequence	PRJNA291963	Humphreys et al. (2015)[9]
DNA sequence	PRJNA280340	Martin et al. (2016)[12]
Chromosome sequence	CP012395	Humphreys et al. (2015)[9]
Plasmid sequence	CP097624.1	This study
Raw proteomics	MassIVE: MSV000089452	This study
Searched proteomics	ProteomeXchange: PXD033792	This study

For plasmid coverage estimates, DNA sequencing reads were trimmed using Trimmomatic (Bolger et al., 2014), (adapter removal and minimum quality score 30) and then mapped to the plasmid pCA sequence using the Bowtie2 (Langmead and Salzberg, 2012) (Illumina, Ion-Torrent) and blasr (Chaisson and Tesler, 2012) (PacBio, 454) algorithms with default parameters. Read coverages for every 50 bp intervals were determined using the deepTools-multiBamSummary (Ramírez et al., 2016). Genome overview and heatmap figures were generated using the Circos (Krzywinski et al., 2009) and R-pheatmap package, respectively. RNA-seq reads were trimmed using Trimmomatic to remove sequencing adapters and bases below a quality score of 30. Paired reads were mapped to the pCA plasmid sequence using Bowtie2 with two alignments per read (k). The alignments were scored with maximum and minimum mismatch penalties (mp) of 5 and 1, respectively, and the natural log function (G). Mapped reads were filtered and sorted using SAMtools (Li et al., 2009), and FPKM values were estimated using Cufflinks utility in the Cufflinks package (Trapnell et al., 2010).

### PCR

PCR primers were designed using Primer3 (version 0.4.0), synthesized by Integrated DNA Technologies (IDT) and the sequences are provided (Table 2). PCR reactions were performed using the Q5 High-Fidelity DNA Polymerase under standard conditions (New England Biolabs).

**TABLE 2:** Oligonucleotides used to confirm plasmids *via* overlapping PCR reactions.

Primer	Gene	Product name	Product size (bp)	Sequence
P1_3349	CAETHG_05110	Product 1	1,807	TCG​TTA​AAA​CCA​CTG​CAG​CC
P1_5155	CAETHG_05120	Product 1		ACT​GGC​CTG​ACT​CTT​CGA​AA
P2_2169	CAETHG_05105	Product 2	1,200	TGA​GCC​AGT​CGA​ATA​CCA​CA
P2_3368	CAETHG_05110	Product 2		GGC​TGC​AGT​GGT​TTT​AAC​GA
P3_483	CAETHG_05090	Product 3	1,706	TGT​GTG​CAT​GGA​GAA​GGT​CA
P3_2188	CAETHG_05105	Product 3		TGT​GGT​ATT​CGA​CTG​GCT​CA
P4_3050	CAETHG_05110	Product 4	1,502	TGT​TAA​CTA​CCG​GCG​TGT​CA
P4_4551	CAETHG_05115	Product 4		AGC​AAG​TCC​ACG​TGA​AGC​TA
P5_2162	CAETHG_05105	Product 5	908	TTC​TTC​CTG​AGC​CAG​TCG​AA
P5_3069	CAETHG_05110	Product 5		TGA​CAC​GCC​GGT​AGT​TAA​CA
P6_3580	intergenic	Product 6	1,651	GGA​GCG​AAC​CCT​TGA​CAT​TT
P6_5230	CAETHG_05120	Product 6		AGG​CTC​GGA​AAC​AGG​ACA​AT
P7_4533	CAETHG_05115	Product 7	2,334	AGC​TTC​ACG​TGG​ACT​TGC​TA
P7_1367	CAETHG_05095	Product 7		TGC​GGT​GCT​AAA​CAT​AAT​GAC​A

### Proteomics

*C. autoethanogenum* strain JA1-1 was obtained from the Deutsche Sammlung von Mikroorganismen und Zellkulturen (DSMZ) culture collection (DSM10061). For proteomic samples, cultures were grown at 37°C using gaseous substrates (50% CO, 10% H_2_, 30% CO_2_, 10% N_2_) and a previously described medium (Valgepea et al., 2017). After ∼24 h, the “lag-phase” cultures had cell densities in the range of OD_600_ 0.09–0.15 and after ∼42 h, the “log-phase” cultures had cell densities in the range of OD_600_ 0.2–0.27. Cell pellets (*n* = 3) from both time points were harvested for proteomics by centrifugation, followed by supernatant removal, rapid freezing in liquid nitrogen, and storage at −80°C until analysis. Cells were lysed by bead-beating in Tris-HCl (100 mM at pH 8.0), adjusting the sample to 4% SDS, and heat-treatment (95°C for 10 min). Crude protein was obtained by centrifugation at ×21,000 g for 10 min followed by quantification with a Nanodrop OneC spectrophotometer (Thermo Scientific). Samples were adjusted to 10 mM dithiothreitol (10 min at 95°C) to reduce proteins, then 30 mM iodoacetamide (20 min at room temperature in darkness), and cleaned up *via* protein aggregation capture (Batth et al., 2019). Aggregated protein [on magnetic Sera-Mag (GE Healthcare) beads] was then digested with proteomic-grade trypsin (1:75 w/w; Promega) in 100 mM Tris-HCl, pH 8.0 overnight at 37°C, and again for 3 h at 37°C the following day. Tryptic peptides were then collected, filtered through a 10 kDa MWCO spin filter (Vivaspin 2; Sartorius), and quantified by Nanodrop OneC. Three micrograms of the peptides were then analyzed by 1D LC-MS/MS using a Vanquish uHPLC coupled directly to an Orbitrap Q Exactive mass spectrometer (Thermo Scientific) as previously described (Walker et al., 2020). Peptides were separated by a 180 min organic gradient across an in-house-pulled nanospray emitter packed with 15 cm of 1.7-micron Kinetex reversed-phase resin (Phenomenex). Peptide fragmentation spectra were analyzed/sequenced by the Proteome Discoverer software (Thermo Scientific) and the peptides quantified by chromatographic area-under-the-curve. Peptide abundances were summed to their respective proteins, protein abundances log2 transformed, and normalized with InfernoRDN (Taverner et al., 2012). Statistical analyses were performed with Perseus (Tyanova et al., 2016).

### Strains and growth experiments

Wild-type *C. autoethanogenum* DSM10061 is available from the German Collection of Microorganisms and Cell Cultures GmbH (DSMZ; Braunschweig, Germany). Strain DSM19630 (derived from DSM10061) was used in these studies and plasmid pMTL80000 plasmid has been described previously (Heap et al., 2009). Methods for the heterologous product synthesis, along with anaerobic techniques and media have been described (Liew et al., 2022). Plasmids pIPA-1 and pIPA-2 were constructed using the described methods (Liew et al., 2022). Briefly, the primers NP_replicon_F (GTG​TTC​TTT​CTT​AAC​TTG​AAT​TGG​CGC​GCC) and NP_replicon_R (GTT​TGA​ACC​TTC​TTC​ACA​TTC​AAT​TGG​CCG​GCC) were used to amplify the native plasmid origin, followed by DNA restriction using the AscI and FseI enzymes, gel extraction, and T4 ligation. The Supplementary Material provides Genbank files for the plasmids respectively. For the growth experiments, the strains were cultured in Schott bottles with a medium containing clarithromycin and using a gas blend (50% CO, 10% H_2_, 30% CO_2_, and 10% N_2_; Airgas) at 37°C, shaking at 120 rpm, and isopropanol was measured by high performance liquid chromatography (HPLC), as described previously (Liew et al., 2022).

## Data Availability

*C. autoethanogenum* DSM10061 is available from DSMZ, the German Collection of Microorganisms and Cell Cultures GmbH. Sequence data used in this study have been previously described and are publicly available (Table 1). Raw peptide, raw protein and quantified proteomic data generated as part of this study are presented (Table 1, Supplementary Tables S1–S2). All other data generated or analyzed in this article are presented in this published article.
