# Supplementary material for: Clostridium autoethanogenum isopropanol production via native plasmid pCA replicon
Source: Front Bioeng Biotechnol. 2022 Aug 5;10:932363. doi: 10.3389/fbioe.2022.932363 (PMC9413188; doi:10.3389/fbioe.2022.932363)
Supplement: Supplementary file 1 [file DataSheet4.docx]

LOCUS pIPA-2 8396 bp DNA circular UNA 19-NOV-2021

DEFINITION Concatenation of 2 sequences.

ACCESSION urn.local...304-ejc6cck

VERSION urn.local...304-ejc6cck

KEYWORDS .

SOURCE

ORGANISM .

FEATURES Location/Qualifiers

ligation 1..4

/label="Ligation"

terminator 6..47

/gene="Cpa fdx terminator"

/label="Cpa fdx terminator"

ligation 50..53

/label="Ligation"

CDS 279..1310

/Genetic_code="Standard"

/Reading_frame=1

/created_by="ORF Finder"

/modified_by="User"

/label="Rep9"

ligation 1354..1357

/label="Ligation"

CDS 1468..2205

/gene="ermB"

/label="ermB CDS"

rep_origin 2395..2940

/gene="ColE1 RNA II"

/label="ColE1 RNA II rep origin"

CDS 3341..3781

/gene="traJ"

/label="traJ CDS"

terminator 3924..3977

/gene="CD0164 terminator"

/label="CD0164 terminator"

ligation 3998..4001

/label="Ligation"

primer_bind complement(4237..4863)

/Sequence="ATGATGTCTAGACTTGATAAAAGTAAAGTTATCAATAGTGCATTGGA

ATTACTAAATGAAGTTGGAATAGAAGGACTAACTACAAGAAAGTTAGCTCAGAAACTT

GGAGTAGAACAACCTACGTTGTATTGGCATGTGAAGAATAAGAGAGCTTTACTTGACG

CCTTAGCTATTGAAATGTTAGATAGACATCACACTCACTTTTGTCCTTTAGAAGGTGA

ATCATGGCAAGATTTTCTTAGGAATAACGCTAAATCTTTTCGTTGTGCTCTATTATCA

CATAGAGATGGGGCTAAAGTACATTTAGGAACTAGACCAACTGAAAAACAATATGAAA

CATTAGAAAATCAATTAGCATTCTTATGTCAACAGGGTTTTAGTTTAGAAAATGCCTT

ATACGCACTAAGTGCTGTAGGACATTTTACTTTAGGTTGCGTATTGGAGGATCAAGAA

CATCAAGTTGCAAAAGAGGAAAGAGAAACACCAACAACAGATTCAATGCCTCCATTGT

TAAGACAAGCAATAGAGTTATTTGATCATCAAGGCGCAGAACCAGCATTTCTTTTTGG

ACTTGAATTGATAATCTGTGGTTTAGAGAAACAACTTAAATGTGAATCTGGTAGCTAA

"

/created_by="User"

/label="tetR"

CDS 5052..6230

/Description="Catalyzes the synthesis of acetoacetyl

coenzyme A from two molecules of acetyl coenzyme A. It can

also act as a thiolase, catalyzing the reverse reaction

and generating two-carbon units from the four-carbon

product of fatty acid oxidation; in Rhizobia and Ralstonia

is involved in PHB biosynthesis"

/product="""""acetyl-CoA acetyltransferase"

/EC_number="""""2.3.1.9"

/protein_id="""""NP_349476.1"

/codon_start

/transl_table=1

/db_xref="""""GeneID:1119056"

/locus_tag="""""CAC2873"

/modified_by="User"

/Transferred_From="thlA"

/Transferred_Similarity="100.00%"

/label="thlA"

CDS 6263..6916

/product="""""""""3-oxoacid CoA-transferase, A subunit"

/modified_by="User"

/Transferred_From="ctfA"

/Transferred_Similarity="100.00%"

/label="ctfA"

CDS 6917..7582

/product="""""""""3-oxoacid CoA-transferase, B subunit"

/modified_by="User"

/Transferred_From="ctfB"

/Transferred_Similarity="100.00%"

/label="ctfB"

CDS 7655..8395

/product="""""""""acetoacetate decarboxylase"

/modified_by="User"

/Transferred_From="adc"

/Transferred_Similarity="100.00%"

/label="adc"

ORIGIN

1 ctagcataaa aataagaagc ctgcatttgc aggcttctta tttttatggc gcgccgtgtt

61 ctttcttaac ttgatactat gtgaacaaca gcaaaataat tttatgtaca ataaaccttt

121 taacatcaat acttcagagg gtgttttttg tatctttttt agcatattat tgacataaaa

181 aaaccccaat gttataattt agttgtccaa tccaaaattt aacacagggt tttttatctt

241 ccgtaaggtt tgaaccctaa cgaaaggaag ttttttaatt gtatgttaat aataacaaag

301 atataagaaa aaatcaaatg gaagttttaa ctgataaaac ccaaaaaggt gaagaaagac

361 cttggaggga aaataaatta aaaactttat cacttgctaa aagttatgaa agaatcggta

421 atactcgtaa agctgataga gttaagaatt gcagtagtca gttgatttat aaaaaagaca

481 aaaatacagg tataaagaag ttacatagta tgatttcgtg tcaggtgagg ttatgtccaa

541 tgtgtgcatg gagaaggtca ttaaaaatat tcggtcaaac atctaaaatt atggataaag

601 ctttagaaaa taaagaatat aggtttatat ttttaacttt aacttgtaga aatgtagaag

661 gtaaagaatt gtccaaaatt attgataatt tgttctatgc atttaaaaaa atgatgctaa

721 aaacgaaagt aaagcaaatt gtcaaaggtt ggtttagagc attggaagta actcataatt

781 tagataaaaa atcaaaggat tataatacat atcatccaca tttccatgta atacttatgg

841 tcaataaaag ctactttaca gatacaaagc agtatttatc acagaaagat tggacaagct

901 tatggaaaga ttgtttaagg gttgattata tgcctatagt taacataaaa gcttttaaaa

961 ctaatactaa aaaagaagta gaaaagtctg ttgcagaatc agcaaaatat acagtaaaag

1021 acaatgatta cttaattgta aatgatgaaa aaatgacaga tgaaactgta tcaatacttg

1081 atggagcatt ggcaaatagg aggcttgttg catttggtgg tgaattaaaa aaaatacata

1141 aatctttaaa tcttagtgat atagagaaag ctagtgtaaa tactgatagt gatgatgatg

1201 ttgtacttag agatgacgtt aattatgtat ttgaagttta taattggaac attggttata

1261 acaatggtca gtatttaaaa gttaaagaaa tagataaaaa ggagaattaa aagtgagttg

1321 taatgttaaa gaatgtgaag aaggttcaaa cggccggccg aagcaaactt aagagtgtgt

1381 tgatagtgca gtatcttaaa attttgtata ataggaattg aagttaaatt agatgctaaa

1441 aatttgtaat taagaaggag tgattacatg aacaaaaata taaaatattc tcaaaacttt

1501 ttaacgagtg aaaaagtact caaccaaata ataaaacaat tgaatttaaa agaaaccgat

1561 accgtttacg aaattggaac aggtaaaggg catttaacga cgaaactggc taaaataagt

1621 aaacaggtaa cgtctattga attagacagt catctattca acttatcgtc agaaaaatta

1681 aaactgaata ctcgtgtcac tttaattcac caagatattc tacagtttca attccctaac

1741 aaacagaggt ataaaattgt tgggagtatt ccttaccatt taagcacaca aattattaaa

1801 aaagtggttt ttgaaagcca tgcgtctgac atctatctga ttgttgaaga aggattctac

1861 aagcgtacct tggatattca ccgaacacta gggttgctct tgcacactca agtctcgatt

1921 cagcaattgc ttaagctgcc agcggaatgc tttcatccta aaccaaaagt aaacagtgtc

1981 ttaataaaac ttacccgcca taccacagat gttccagata aatattggaa gctatatacg

2041 tactttgttt caaaatgggt caatcgagaa tatcgtcaac tgtttactaa aaatcagttt

2101 catcaagcaa tgaaacacgc caaagtaaac aatttaagta ccgttactta tgagcaagta

2161 ttgtctattt ttaatagtta tctattattt aacgggagga aataattcta tgagtcgctt

2221 ttgtaaattt ggaaagttac acgttactaa agggaatgtg tttaaactcc tttttgataa

2281 tctcatgacc aaaatccctt aacgtgagtt ttcgttccac tgagcgtcag accccgtaga

2341 aaagatcaaa ggatcttctt gagatccttt ttttctgcgc gtaatctgct gcttgcaaac

2401 aaaaaaacca ccgctaccag cggtggtttg tttgccggat caagagctac caactctttt

2461 tccgaaggta actggcttca gcagagcgca gataccaaat actgttcttc tagtgtagcc

2521 gtagttaggc caccacttca agaactctgt agcaccgcct acatacctcg ctctgctaat

2581 cctgttacca gtggctgctg ccagtggcga taagtcgtgt cttaccgggt tggactcaag

2641 acgatagtta ccggataagg cgcagcggtc gggctgaacg gggggttcgt gcacacagcc

2701 cagcttggag cgaacgacct acaccgaact gagataccta cagcgtgagc tatgagaaag

2761 cgccacgctt cccgaaggga gaaaggcgga caggtatccg gtaagcggca gggtcggaac

2821 aggagagcgc acgagggagc ttccaggggg aaacgcctgg tatctttata gtcctgtcgg

2881 gtttcgccac ctctgacttg agcgtcgatt tttgtgatgc tcgtcagggg ggcggagcct

2941 atggaaaaac gccagcaacg cggccttttt acggttcctg gccttttgct ggccttttgc

3001 tcacatgttc tttcctgcgt tatcccctga ttctgtggat aaccgtatta ccgcctttga

3061 gtgagctgat accgctcgcc gcagccgaac gaccgagcgc agcgagtcag tgagcgagga

3121 agcggaagag cgcccaatac gcagggcccc ctgcttcggg gtcattatag cgattttttc

3181 ggtatatcca tcctttttcg cacgatatac aggattttgc caaagggttc gtgtagactt

3241 tccttggtgt atccaacggc gtcagccggg caggataggt gaagtaggcc cacccgcgag

3301 cgggtgttcc ttcttcactg tcccttattc gcacctggcg gtgctcaacg ggaatcctgc

3361 tctgcgaggc tggccggcta ccgccggcgt aacagatgag ggcaagcgga tggctgatga

3421 aaccaagcca accaggaagg gcagcccacc tatcaaggtg tactgccttc cagacgaacg

3481 aagagcgatt gaggaaaagg cggcggcggc cggcatgagc ctgtcggcct acctgctggc

3541 cgtcggccag ggctacaaaa tcacgggcgt cgtggactat gagcacgtcc gcgagctggc

3601 ccgcatcaat ggcgacctgg gccgcctggg cggcctgctg aaactctggc tcaccgacga

3661 cccgcgcacg gcgcggttcg gtgatgccac gatcctcgcc ctgctggcga agatcgaaga

3721 gaagcaggac gagcttggca aggtcatgat gggcgtggtc cgcccgaggg cagagccatg

3781 acttttttag ccgctaaaac ggccgggggg tgcgcgtgat tgccaagcac gtccccatgc

3841 gctccatcaa gaagagcgac ttcgcggagc tggtgaagta catcaccgac gagcaaggca

3901 agaccgatcg ggccccctgc aggataaaaa aattgtagat aaattttata aaatagtttt

3961 atctacaatt tttttatcag gaaacagcta tgaccgcggc cgcggcgcca agcttagaaa

4021 aatataaata agaagtagct ttaagagaat taaattatta agaaaagcaa aggtgtttaa

4081 aaaataaatt tttaaacacc tttgcttttc ttaaattata aataagataa aaaagaatcc

4141 tgaataaaat aaaaaggggt gtctcaaaat tttattttga gacgacccct ttttattcta

4201 tatgtcgatg ctatagctga gatcgtggaa ttcttgttag ctaccagatt cacatttaag

4261 ttgtttctct aaaccacaga ttatcaattc aagtccaaaa agaaatgctg gttctgcgcc

4321 ttgatgatca aataactcta ttgcttgtct taacaatgga ggcattgaat ctgttgttgg

4381 tgtttctctt tcctcttttg caacttgatg ttcttgatcc tccaatacgc aacctaaagt

4441 aaaatgtcct acagcactta gtgcgtataa ggcattttct aaactaaaac cctgttgaca

4501 taagaatgct aattgatttt ctaatgtttc atattgtttt tcagttggtc tagttcctaa

4561 atgtacttta gccccatctc tatgtgataa tagagcacaa cgaaaagatt tagcgttatt

4621 cctaagaaaa tcttgccatg attcaccttc taaaggacaa aagtgagtgt gatgtctatc

4681 taacatttca atagctaagg cgtcaagtaa agctctctta ttcttcacat gccaatacaa

4741 cgtaggttgt tctactccaa gtttctgagc taactttctt gtagttagtc cttctattcc

4801 aacttcattt agtaattcca atgcactatt gataacttta cttttatcaa gtctagacat

4861 catttaatat cctcctcttc aatatattta agtcgactga tcggatcctg atcggagctc

4921 ccatggcggc cggtcgatat cgatcttaaa agtttgctat taagtattga gcttctatca

4981 ttgataggtt ataatgaaca ttgtagaatt cccataataa agaaagaatt ttaaataaag

5041 gaggaacaca tatgaaagaa gttgtaatag ctagtgcagt aagaacagcg attggatctt

5101 atggaaagtc tcttaaggat gtaccagcag tagatttagg agctacagct ataaaggaag

5161 cagttaaaaa agcaggaata aaaccagagg atgttaatga agtcatttta ggaaatgttc

5221 ttcaagcagg tttaggacag aatccagcaa gacaggcatc ttttaaagca ggattaccag

5281 ttgaaattcc agctatgact attaataagg tttgtggttc aggacttaga acagttagct

5341 tagcagcaca aattataaaa gcaggagatg ctgacgtaat aatagcaggt ggtatggaaa

5401 atatgtctag agctccttac ttagcgaata acgctagatg gggatataga atgggaaacg

5461 ctaaatttgt tgatgaaatg atcactgacg gattgtggga tgcatttaat gattaccaca

5521 tgggaataac agcagaaaac atagctgaga gatggaacat ttcaagagaa gaacaagatg

5581 agtttgctct tgcatcacaa aaaaaagctg aagaagctat aaaatcaggt caatttaaag

5641 atgaaatagt tcctgtagta attaaaggca gaaagggaga aactgtagtt gatacagatg

5701 agcaccctag atttggatca actatagaag gacttgcaaa attaaaacct gccttcaaaa

5761 aagatggaac agttacagct ggtaatgcat caggattaaa tgactgtgca gcagtacttg

5821 taatcatgag tgcagaaaaa gctaaagagc ttggagtaaa accacttgct aagatagttt

5881 cttatggttc agcaggagtt gacccagcaa taatgggata tggacctttc tatgcaacaa

5941 aagcagctat tgaaaaagca ggttggacag ttgatgaatt agatttaata gaatcaaatg

6001 aagcttttgc agctcaaagt ttagcagtag caaaagattt aaaatttgat atgaataaag

6061 taaatgtaaa tggaggagct attgcccttg gtcatccaat tggagcatca ggtgcaagaa

6121 tactcgttac tcttgtacac gcaatgcaaa aaagagatgc aaaaaaaggc ttagcaactt

6181 tatgtatagg tggcggacaa ggaacagcaa tattgctaga aaagtgctag gaattcgagc

6241 tcggtaccag ggagatatta aaatgaataa attagtaaaa ttaacagatt taaagcgcat

6301 tttcaaagat ggcatgacaa ttatggttgg gggtttttta gattgtggaa ctcctgaaaa

6361 tattatagat atgctagttg atttaaatat aaaaaatctg actattataa gcaatgatac

6421 agcttttcct aataaaggaa taggaaaact tattgtaaat ggtcaagttt ctaaagtaat

6481 tgcttcacat attggaacta atcctgaaac tggaaaaaaa atgagctctg gagaacttaa

6541 agttgagctt tccccacaag gaacactgat tgaaagaatt cgtgcagctg gatctggact

6601 cggaggtgta ttaactccaa ctggacttgg aactatcgtt gaagaaggta agaaaaaagt

6661 tactatcgat ggcaaagaat atctattaga acttccttta tctgctgatg tttcattaat

6721 aaaaggtagc attgtagatg aatttggaaa taccttctat agggctgcta ctaaaaattt

6781 caatccatat atggcaatgg ctgcaaaaac agttatagtt gaagcagaaa atttagttaa

6841 atgtgaagat ttaaaaagag atgccataat gactcctggc gtattagtag attatatcgt

6901 taaggaggcg gcttaattga ttgtagataa agttttagca aaagagataa ttgccaaaag

6961 agttgcaaaa gaactaaaaa aagaccaact cgtaaacctt ggaataggac ttccaacttt

7021 agtagcaaat tatgtaccaa aagaaatgaa cattactttt gaatcagaaa atggcatggt

7081 tggtatggca caaatggcat catcaggtga aaatgaccca gatataataa atgctggcgg

7141 ggaatatgta acattattac ctcaaggttc attttttgat agttcaatgt ctttcgcact

7201 aatacgagga ggacatgttg atgttgctgt tcttggtgct ctagaagttg atgaaaaagg

7261 taatttagct aactggattg ttccaaataa aattgtccca ggtatgggtg gcgctatgga

7321 tttagcaata ggcgcaaaaa aaataatagt ggcaatgcaa catacaggaa aaagtaaacc

7381 taaaatcgtt aaaaaatgta ctctcccact tactgctaag gctcaagtgg atttaattgt

7441 cacagaactt tgtgtaattg atgtaacaaa tgacggctta cttttaaaag aaattcataa

7501 agatacaact attgatgaaa ttaaattttt aacagatgca gatttaatta ttccagataa

7561 cttaaagatt atggatatat gaatcattct attttaaata tataacttta aaaatcttat

7621 gtattaaaaa ctaagaaaag aggttgattg ttttatgtta gaaagtgaag tatctaaaca

7681 aattacaact ccacttgctg ctccagcgtt tcctagagga ccatataggt ttcacaatag

7741 agaatatcta aacattattt atcgaactga tttagatgct cttcgaaaaa tagtaccaga

7801 gccacttgaa ttagatagag catatgttag atttgaaatg atggctatgc ctgatacaac

7861 cggactaggc tcatatacag aatgtggtca agctattcca gtaaaatata atggtgttaa

7921 gggtgactac ttgcatatga tgtatctaga taatgaacct gctattgctg ttggaagaga

7981 aagtagcgct tatccaaaaa agcttggcta tccaaagcta tttgttgatt cagatacttt

8041 agttgggaca cttaaatatg gtacattacc agtagctact gcaacaatgg gatataagca

8101 cgagcctcta gatcttaaag aagcctatgc tcaaattgca agacccaatt ttatgctaaa

8161 aatcattcaa ggttacgatg gtaagccaag aatttgtgaa ctaatatgtg cagaaaatac

8221 tgatataact attcacggtg cttggactgg aagtgcacgt ctacaattat ttagccatgc

8281 actagctcct cttgctgatt tacctgtatt agagattgta tcagcatctc atatcctcac

8341 agatttaact cttggaacac ctaaggttgt acatgattat ctttcagtaa aataag

//
