# Supplementary material for: Clostridium autoethanogenum isopropanol production via native plasmid pCA replicon
Source: Front Bioeng Biotechnol. 2022 Aug 5;10:932363. doi: 10.3389/fbioe.2022.932363 (PMC9413188; doi:10.3389/fbioe.2022.932363)
Supplement: Supplementary file 2 [file DataSheet3.docx]

LOCUS pIPA-1 9545 bp DNA circular UNA 19-NOV-2021

DEFINITION Concatenation of 2 sequences (Circularized).

ACCESSION urn.local...ak-4smdg48

VERSION urn.local...ak-4smdg48

KEYWORDS .

SOURCE

ORGANISM .

FEATURES Location/Qualifiers

ligation 1..4

/label="Ligation"

terminator 6..47

/gene="Cpa fdx terminator"

CDS 568..1623

/gene="repA"

CDS complement(1714..2217)

/gene="orf2"

CDS 2563..3300

/gene="ermB"

rep_origin 3490..4035

/gene="ColE1 RNA II"

CDS 4436..4876

/gene="traJ"

terminator 5019..5072

/gene="CD0164 terminator"

ligation 5093..5096

/label="Ligation"

primer_bind complement(5332..5958)

/Sequence="ATGATGTCTAGACTTGATAAAAGTAAAGTTATCAATAGTGCATTGGA

ATTACTAAATGAAGTTGGAATAGAAGGACTAACTACAAGAAAGTTAGCTCAGAAACTT

GGAGTAGAACAACCTACGTTGTATTGGCATGTGAAGAATAAGAGAGCTTTACTTGACG

CCTTAGCTATTGAAATGTTAGATAGACATCACACTCACTTTTGTCCTTTAGAAGGTGA

ATCATGGCAAGATTTTCTTAGGAATAACGCTAAATCTTTTCGTTGTGCTCTATTATCA

CATAGAGATGGGGCTAAAGTACATTTAGGAACTAGACCAACTGAAAAACAATATGAAA

CATTAGAAAATCAATTAGCATTCTTATGTCAACAGGGTTTTAGTTTAGAAAATGCCTT

ATACGCACTAAGTGCTGTAGGACATTTTACTTTAGGTTGCGTATTGGAGGATCAAGAA

CATCAAGTTGCAAAAGAGGAAAGAGAAACACCAACAACAGATTCAATGCCTCCATTGT

TAAGACAAGCAATAGAGTTATTTGATCATCAAGGCGCAGAACCAGCATTTCTTTTTGG

ACTTGAATTGATAATCTGTGGTTTAGAGAAACAACTTAAATGTGAATCTGGTAGCTAA

"

/created_by="User"

/label="tetR"

CDS 6201..7379

/Description="Catalyzes the synthesis of acetoacetyl

coenzyme A from two molecules of acetyl coenzyme A. It can

also act as a thiolase, catalyzing the reverse reaction

and generating two-carbon units from the four-carbon

product of fatty acid oxidation; in Rhizobia and Ralstonia

is involved in PHB biosynthesis"

/product="""""acetyl-CoA acetyltransferase"

/EC_number="""""2.3.1.9"

/protein_id="""""NP_349476.1"

/codon_start

/transl_table=1

/db_xref="""""GeneID:1119056"

/locus_tag="""""CAC2873"

/modified_by="User"

/Transferred_From="thlA"

/Transferred_Similarity="100.00%"

/label="thlA"

CDS 7412..8065

/product="""""""""3-oxoacid CoA-transferase, A subunit"

/modified_by="User"

/Transferred_From="ctfA"

/Transferred_Similarity="100.00%"

/label="ctfA"

CDS 8066..8731

/product="""""""""3-oxoacid CoA-transferase, B subunit"

/modified_by="User"

/Transferred_From="ctfB"

/Transferred_Similarity="100.00%"

/label="ctfB"

CDS 8804..9544

/product="""""""""acetoacetate decarboxylase"

/modified_by="User"

/Transferred_From="adc"

/Transferred_Similarity="100.00%"

/label="adc"

ORIGIN

1 ctagcataaa aataagaagc ctgcatttgc aggcttctta tttttatggc gcgccgttct

61 gaatccttag ctaatggttc aacaggtaac tatgacgaag atagcaccct ggataagtct

121 gtaatggatt ctaaggcatt taatgaagac gtgtatataa aatgtgctaa tgaaaaagaa

181 aatgcgttaa aagagcctaa aatgagttca aatggttttg aaattgattg gtagtttaat

241 ttaatatatt ttttctattg gctatctcga tacctataga atcttctgtt cacttttgtt

301 tttgaaatat aaaaaggggc tttttagccc ctttttttta aaactccgga ggagtttctt

361 cattcttgat actatacgta actattttcg atttgacttc attgtcaatt aagctagtaa

421 aatcaatggt taaaaaacaa aaaacttgca tttttctacc tagtaattta taattttaag

481 tgtcgagttt aaaagtataa tttaccagga aaggagcaag ttttttaata aggaaaaatt

541 tttcctttta aaattctatt tcgttatatg actaattata atcaaaaaaa tgaaaataaa

601 caagaggtaa aaactgcttt agagaaatgt actgataaaa aaagaaaaaa tcctagattt

661 acgtcataca tagcaccttt aactactaag aaaaatattg aaaggacttc cacttgtgga

721 gattatttgt ttatgttgag tgatgcagac ttagaacatt ttaaattaca taaaggtaat

781 ttttgcggta atagattttg tccaatgtgt agttggcgac ttgcttgtaa ggatagttta

841 gaaatatcta ttcttatgga gcatttaaga aaagaagaaa ataaagagtt tatattttta

901 actcttacaa ctccaaatgt aaaaagttat gatcttaatt attctattaa acaatataat

961 aaatctttta aaaaattaat ggagcgtaag gaagttaagg atataactaa aggttatata

1021 agaaaattag aagtaactta ccaaaaggaa aaatacataa caaaggattt atggaaaata

1081 aaaaaagatt attatcaaaa aaaaggactt gaaattggtg atttagaacc taattttgat

1141 acttataatc ctcattttca tgtagttatt gcagttaata aaagttattt tacagataaa

1201 aattattata taaatcgaga aagatggttg gaattatgga agtttgctac taaggatgat

1261 tctataactc aagttgatgt tagaaaagca aaaattaatg attataaaga ggtttacgaa

1321 cttgcgaaat attcagctaa agacactgat tatttaatat cgaggccagt atttgaaatt

1381 ttttataaag cattaaaagg caagcaggta ttagttttta gtggattttt taaagatgca

1441 cacaaattgt acaagcaagg aaaacttgat gtttataaaa agaaagatga aattaaatat

1501 gtctatatag tttattataa ttggtgcaaa aaacaatatg aaaaaactag aataagggaa

1561 cttacggaag atgaaaaaga agaattaaat caagatttaa tagatgaaat agaaatagat

1621 taaagtgtaa ctatacttta tatatatatg attaaaaaaa taaaaaacaa cagcctatta

1681 ggttgttgtt ttttattttc tttattaatt tttttaattt ttagttttta gttctttttt

1741 aaaataagtt tcagcctctt tttcaatatt ttttaaagaa ggagtatttg catgaattgc

1801 cttttttcta acagacttag gaaatatttt aacagtatct tcttgcgccg gtgattttgg

1861 aacttcataa cttactaatt tataattatt attttctttt ttaattgtaa cagttgcaaa

1921 agaagctgaa cctgttcctt caactagttt atcatcttca atataatatt cttgacctat

1981 atagtataaa tatattttta ttatattttt acttttttct gaatctatta ttttataatc

2041 ataaaaagtt ttaccaccaa aagaaggttg tactccttct ggtccaacat atttttttac

2101 tatattatct aaataatttt tgggaactgg tgttgtaatt tgattaatcg aacaaccagt

2161 tatacttaaa ggaattataa ctataaaaat atataggatt atctttttaa atttcattat

2221 tggcctcctt tttattaaat ttatgttacc ataaaaagga cataacggga atatgtagaa

2281 tatttttaat gtagacaaaa ttttacataa atataaagaa aggaagtgtt tgtttaaatt

2341 ttatagcaaa ctatcaaaaa ttagggggat aaaaatttat gaaaaaaagg ttttcgatgt

2401 tatttttatg tttaacttta atagtttgtg gtttatttac aaattcggcc ggccgaagca

2461 aacttaagag tgtgttgata gtgcagtatc ttaaaatttt gtataatagg aattgaagtt

2521 aaattagatg ctaaaaattt gtaattaaga aggagtgatt acatgaacaa aaatataaaa

2581 tattctcaaa actttttaac gagtgaaaaa gtactcaacc aaataataaa acaattgaat

2641 ttaaaagaaa ccgataccgt ttacgaaatt ggaacaggta aagggcattt aacgacgaaa

2701 ctggctaaaa taagtaaaca ggtaacgtct attgaattag acagtcatct attcaactta

2761 tcgtcagaaa aattaaaact gaatactcgt gtcactttaa ttcaccaaga tattctacag

2821 tttcaattcc ctaacaaaca gaggtataaa attgttggga gtattcctta ccatttaagc

2881 acacaaatta ttaaaaaagt ggtttttgaa agccatgcgt ctgacatcta tctgattgtt

2941 gaagaaggat tctacaagcg taccttggat attcaccgaa cactagggtt gctcttgcac

3001 actcaagtct cgattcagca attgcttaag ctgccagcgg aatgctttca tcctaaacca

3061 aaagtaaaca gtgtcttaat aaaacttacc cgccatacca cagatgttcc agataaatat

3121 tggaagctat atacgtactt tgtttcaaaa tgggtcaatc gagaatatcg tcaactgttt

3181 actaaaaatc agtttcatca agcaatgaaa cacgccaaag taaacaattt aagtaccgtt

3241 acttatgagc aagtattgtc tatttttaat agttatctat tatttaacgg gaggaaataa

3301 ttctatgagt cgcttttgta aatttggaaa gttacacgtt actaaaggga atgtgtttaa

3361 actccttttt gataatctca tgaccaaaat cccttaacgt gagttttcgt tccactgagc

3421 gtcagacccc gtagaaaaga tcaaaggatc ttcttgagat cctttttttc tgcgcgtaat

3481 ctgctgcttg caaacaaaaa aaccaccgct accagcggtg gtttgtttgc cggatcaaga

3541 gctaccaact ctttttccga aggtaactgg cttcagcaga gcgcagatac caaatactgt

3601 tcttctagtg tagccgtagt taggccacca cttcaagaac tctgtagcac cgcctacata

3661 cctcgctctg ctaatcctgt taccagtggc tgctgccagt ggcgataagt cgtgtcttac

3721 cgggttggac tcaagacgat agttaccgga taaggcgcag cggtcgggct gaacgggggg

3781 ttcgtgcaca cagcccagct tggagcgaac gacctacacc gaactgagat acctacagcg

3841 tgagctatga gaaagcgcca cgcttcccga agggagaaag gcggacaggt atccggtaag

3901 cggcagggtc ggaacaggag agcgcacgag ggagcttcca gggggaaacg cctggtatct

3961 ttatagtcct gtcgggtttc gccacctctg acttgagcgt cgatttttgt gatgctcgtc

4021 aggggggcgg agcctatgga aaaacgccag caacgcggcc tttttacggt tcctggcctt

4081 ttgctggcct tttgctcaca tgttctttcc tgcgttatcc cctgattctg tggataaccg

4141 tattaccgcc tttgagtgag ctgataccgc tcgccgcagc cgaacgaccg agcgcagcga

4201 gtcagtgagc gaggaagcgg aagagcgccc aatacgcagg gccccctgct tcggggtcat

4261 tatagcgatt ttttcggtat atccatcctt tttcgcacga tatacaggat tttgccaaag

4321 ggttcgtgta gactttcctt ggtgtatcca acggcgtcag ccgggcagga taggtgaagt

4381 aggcccaccc gcgagcgggt gttccttctt cactgtccct tattcgcacc tggcggtgct

4441 caacgggaat cctgctctgc gaggctggcc ggctaccgcc ggcgtaacag atgagggcaa

4501 gcggatggct gatgaaacca agccaaccag gaagggcagc ccacctatca aggtgtactg

4561 ccttccagac gaacgaagag cgattgagga aaaggcggcg gcggccggca tgagcctgtc

4621 ggcctacctg ctggccgtcg gccagggcta caaaatcacg ggcgtcgtgg actatgagca

4681 cgtccgcgag ctggcccgca tcaatggcga cctgggccgc ctgggcggcc tgctgaaact

4741 ctggctcacc gacgacccgc gcacggcgcg gttcggtgat gccacgatcc tcgccctgct

4801 ggcgaagatc gaagagaagc aggacgagct tggcaaggtc atgatgggcg tggtccgccc

4861 gagggcagag ccatgacttt tttagccgct aaaacggccg gggggtgcgc gtgattgcca

4921 agcacgtccc catgcgctcc atcaagaaga gcgacttcgc ggagctggtg aagtacatca

4981 ccgacgagca aggcaagacc gatcgggccc cctgcaggat aaaaaaattg tagataaatt

5041 ttataaaata gttttatcta caattttttt atcaggaaac agctatgacc gcggccgcgg

5101 cgccaagctt agaaaaatat aaataagaag tagctttaag agaattaaat tattaagaaa

5161 agcaaaggtg tttaaaaaat aaatttttaa acacctttgc ttttcttaaa ttataaataa

5221 gataaaaaag aatcctgaat aaaataaaaa ggggtgtctc aaaattttat tttgagacga

5281 ccccttttta ttctatatgt cgatgctata gctgagatcg tggaattctt gttagctacc

5341 agattcacat ttaagttgtt tctctaaacc acagattatc aattcaagtc caaaaagaaa

5401 tgctggttct gcgccttgat gatcaaataa ctctattgct tgtcttaaca atggaggcat

5461 tgaatctgtt gttggtgttt ctctttcctc ttttgcaact tgatgttctt gatcctccaa

5521 tacgcaacct aaagtaaaat gtcctacagc acttagtgcg tataaggcat tttctaaact

5581 aaaaccctgt tgacataaga atgctaattg attttctaat gtttcatatt gtttttcagt

5641 tggtctagtt cctaaatgta ctttagcccc atctctatgt gataatagag cacaacgaaa

5701 agatttagcg ttattcctaa gaaaatcttg ccatgattca ccttctaaag gacaaaagtg

5761 agtgtgatgt ctatctaaca tttcaatagc taaggcgtca agtaaagctc tcttattctt

5821 cacatgccaa tacaacgtag gttgttctac tccaagtttc tgagctaact ttcttgtagt

5881 tagtccttct attccaactt catttagtaa ttccaatgca ctattgataa ctttactttt

5941 atcaagtcta gacatcattt aatatcctcc tcttcaatat atttaagtcg actgatcgga

6001 tccaatttat acgttttctc taacaactta attataccca ctattattat ttttatcaat

6061 atagagctcc catggcggcc ggtcgatatc gatcttaaaa gtttgctatt aagtattgag

6121 cttctatcat tgataggtta taatgaacat tgtagaattc ccataataaa gaaagaattt

6181 taaataaagg aggaacacat atgaaagaag ttgtaatagc tagtgcagta agaacagcga

6241 ttggatctta tggaaagtct cttaaggatg taccagcagt agatttagga gctacagcta

6301 taaaggaagc agttaaaaaa gcaggaataa aaccagagga tgttaatgaa gtcattttag

6361 gaaatgttct tcaagcaggt ttaggacaga atccagcaag acaggcatct tttaaagcag

6421 gattaccagt tgaaattcca gctatgacta ttaataaggt ttgtggttca ggacttagaa

6481 cagttagctt agcagcacaa attataaaag caggagatgc tgacgtaata atagcaggtg

6541 gtatggaaaa tatgtctaga gctccttact tagcgaataa cgctagatgg ggatatagaa

6601 tgggaaacgc taaatttgtt gatgaaatga tcactgacgg attgtgggat gcatttaatg

6661 attaccacat gggaataaca gcagaaaaca tagctgagag atggaacatt tcaagagaag

6721 aacaagatga gtttgctctt gcatcacaaa aaaaagctga agaagctata aaatcaggtc

6781 aatttaaaga tgaaatagtt cctgtagtaa ttaaaggcag aaagggagaa actgtagttg

6841 atacagatga gcaccctaga tttggatcaa ctatagaagg acttgcaaaa ttaaaacctg

6901 ccttcaaaaa agatggaaca gttacagctg gtaatgcatc aggattaaat gactgtgcag

6961 cagtacttgt aatcatgagt gcagaaaaag ctaaagagct tggagtaaaa ccacttgcta

7021 agatagtttc ttatggttca gcaggagttg acccagcaat aatgggatat ggacctttct

7081 atgcaacaaa agcagctatt gaaaaagcag gttggacagt tgatgaatta gatttaatag

7141 aatcaaatga agcttttgca gctcaaagtt tagcagtagc aaaagattta aaatttgata

7201 tgaataaagt aaatgtaaat ggaggagcta ttgcccttgg tcatccaatt ggagcatcag

7261 gtgcaagaat actcgttact cttgtacacg caatgcaaaa aagagatgca aaaaaaggct

7321 tagcaacttt atgtataggt ggcggacaag gaacagcaat attgctagaa aagtgctagg

7381 aattcgagct cggtaccagg gagatattaa aatgaataaa ttagtaaaat taacagattt

7441 aaagcgcatt ttcaaagatg gcatgacaat tatggttggg ggttttttag attgtggaac

7501 tcctgaaaat attatagata tgctagttga tttaaatata aaaaatctga ctattataag

7561 caatgataca gcttttccta ataaaggaat aggaaaactt attgtaaatg gtcaagtttc

7621 taaagtaatt gcttcacata ttggaactaa tcctgaaact ggaaaaaaaa tgagctctgg

7681 agaacttaaa gttgagcttt ccccacaagg aacactgatt gaaagaattc gtgcagctgg

7741 atctggactc ggaggtgtat taactccaac tggacttgga actatcgttg aagaaggtaa

7801 gaaaaaagtt actatcgatg gcaaagaata tctattagaa cttcctttat ctgctgatgt

7861 ttcattaata aaaggtagca ttgtagatga atttggaaat accttctata gggctgctac

7921 taaaaatttc aatccatata tggcaatggc tgcaaaaaca gttatagttg aagcagaaaa

7981 tttagttaaa tgtgaagatt taaaaagaga tgccataatg actcctggcg tattagtaga

8041 ttatatcgtt aaggaggcgg cttaattgat tgtagataaa gttttagcaa aagagataat

8101 tgccaaaaga gttgcaaaag aactaaaaaa agaccaactc gtaaaccttg gaataggact

8161 tccaacttta gtagcaaatt atgtaccaaa agaaatgaac attacttttg aatcagaaaa

8221 tggcatggtt ggtatggcac aaatggcatc atcaggtgaa aatgacccag atataataaa

8281 tgctggcggg gaatatgtaa cattattacc tcaaggttca ttttttgata gttcaatgtc

8341 tttcgcacta atacgaggag gacatgttga tgttgctgtt cttggtgctc tagaagttga

8401 tgaaaaaggt aatttagcta actggattgt tccaaataaa attgtcccag gtatgggtgg

8461 cgctatggat ttagcaatag gcgcaaaaaa aataatagtg gcaatgcaac atacaggaaa

8521 aagtaaacct aaaatcgtta aaaaatgtac tctcccactt actgctaagg ctcaagtgga

8581 tttaattgtc acagaacttt gtgtaattga tgtaacaaat gacggcttac ttttaaaaga

8641 aattcataaa gatacaacta ttgatgaaat taaattttta acagatgcag atttaattat

8701 tccagataac ttaaagatta tggatatatg aatcattcta ttttaaatat ataactttaa

8761 aaatcttatg tattaaaaac taagaaaaga ggttgattgt tttatgttag aaagtgaagt

8821 atctaaacaa attacaactc cacttgctgc tccagcgttt cctagaggac catataggtt

8881 tcacaataga gaatatctaa acattattta tcgaactgat ttagatgctc ttcgaaaaat

8941 agtaccagag ccacttgaat tagatagagc atatgttaga tttgaaatga tggctatgcc

9001 tgatacaacc ggactaggct catatacaga atgtggtcaa gctattccag taaaatataa

9061 tggtgttaag ggtgactact tgcatatgat gtatctagat aatgaacctg ctattgctgt

9121 tggaagagaa agtagcgctt atccaaaaaa gcttggctat ccaaagctat ttgttgattc

9181 agatacttta gttgggacac ttaaatatgg tacattacca gtagctactg caacaatggg

9241 atataagcac gagcctctag atcttaaaga agcctatgct caaattgcaa gacccaattt

9301 tatgctaaaa atcattcaag gttacgatgg taagccaaga atttgtgaac taatatgtgc

9361 agaaaatact gatataacta ttcacggtgc ttggactgga agtgcacgtc tacaattatt

9421 tagccatgca ctagctcctc ttgctgattt acctgtatta gagattgtat cagcatctca

9481 tatcctcaca gatttaactc ttggaacacc taaggttgta catgattatc tttcagtaaa

9541 ataag

//
