# Supplementary material for: Clostridium autoethanogenum isopropanol production via native plasmid pCA replicon
Source: Front Bioeng Biotechnol. 2022 Aug 5;10:932363. doi: 10.3389/fbioe.2022.932363 (PMC9413188; doi:10.3389/fbioe.2022.932363)
Supplement: Supplementary file 3 [file DataSheet1.PDF]

Clostridium autoethanogenum isopropanol production via native plasmid pCA replicon.

Robert Nogle1,¶, Sagar M. Utturkar2,¶, Shilpa Nagaraju1,¶,Richard J. Giannone3,¶, Vinicio Reynoso1, Ching Leang1, Robert L. Hettich3, Wayne P. Mitchell1, Sean D. Simpson1, Michael C. Jewett4,5,6,7,8, Michael Köpke1, and Steven D. Brown1,*

```
pCA_DSO                 TTCTTTCTTAACTTGATACTAT
pC194_NC_002013         TTCTTTCTTATCTTGATAATAA
p1414_AF091592          TCTTTTCTTATCTTGATACTAT
pBC1_BC1REP             ---TTTCTTATCTTGATA----
pUB110_PB0110CG         TTCTTTCTTATCTTGATACATA
pA01_AB040121           TTTTTTCTTATCTTGATCAAGT
                           **************
```

Supplemental Figure S1: pUB110/pMV158-like plus double-strand origin (DSO) of replication conserved in pCA. Multiple sequences were aligned using ClustalW.

```
pUB110_SSOU     ---AAAATA-AAAATTGGCAAGGG---TTTAAAGGTGGAGA-TTTTTTGAGTGATCTTCT
pMV158_SSOU     ---AAAATA-AAAATTGGCAAGGG---TTTAAAGGTGGAGACTTTTTTGAGTGATCTTCT
pCA             GTAAAAATATAAGATGAGCTATTGCCATTTTTAAGTTCAGAATTTAAATATTCAACCCTC
                   ****** ** **  ** *  *   ***  * **  *** ***    * * * *

pUB110_SSOU     CAAAAAATACTACCTGTCCCTTGCTGATTTTTAAACGAGCACGAGAGCAAAACCCCCCTT
pMV158_SSOU     CAAAAAATACTACCTGTCCCTTGCTGATTTTTAAACGAGCACGAGAGCAAAACCCCCCTT
pCA             TTATCAATGCTTAAATTTTTATATTCAGCAGCATCCATTTATAAACACA---CCCCTATA
                  *  *** **     *    *  * *     *  *    *  *   **   ****  *

pUB110_SSOU     TGCTGAGGTGGCAGAGGGCAGGTTTTTTTGTTTCTTTTTTCTCGTAAA
pMV158_SSOU     TGCTGAGGTGGCAGAGGGCAGGTTTTTTTGTTTCTTTTTTCTCGTAAA
pCA             TAACAATATATTTATTACCA--TTTATTTACTTATATGTT--------
                *    *  *         **  *** ***  ** * * **
```

Supplemental Figure S2: Putative pUB110/pMV158 SSOU – like sequence in pCA. Sequences were aligned using ClustalW.

```
pMV158_NC_010096    ACTTTATGAATATAAAGTATAGTGTGTTATACTTTACAT-GGAAG
pUB110_PB0110CG     ACTTTATGAATATAAAGTATAGTGTGTTATACTTTACTTTGGAAG
pCA_OriT            ACTTTATT--TGTAAAGTATACTATGCTATGCTTTACAT-AAAAG
                    *******   * ********* * ** *** ****** *   ***
```

Supplemental Figure S3: pUB110/pMV158-like OriT conserved in pCA. Sequences were aligned using ClustalW. The imperfect PalD palindrome pCA is highlighted.

Supplemental Table S1. Twenty most abundant *Clostridium autoethanogenum* DSM10061 proteins and detected plasmid proteins.

Locus	Product	[a]FDR	[b]Cov(%)	[c]Pep	[d]UqPep	[e]PSM	[f]Lag 1	Lag 2	Lag 3	Log 1	Log 2	Log 3	[g]median abundance
CLAU_1576	Formate-tetrahydrofolate ligase	0.00	61	37	37	5032	40.90	41.06	41.03	40.97	40.96	40.95	40.97
CLAU_2492	Cell wall binding repeat 2-containing protein	0.00	56	58	58	12331	40.42	40.65	40.88	40.68	40.59	40.67	40.66
CLAU_1794	Alcohol dehydrogenase	0.00	68	25	23	4016	39.38	39.51	39.29	40.55	40.42	40.50	39.97
CLAU_1568	CO dehydrogenase/acetyl-CoA synthase delta	0.00	65	33	33	3813	39.92	40.11	39.81	39.95	39.94	39.94	39.94
CLAU_1566	CO dehydrogenase/acetyl-CoA synthase complex	0.00	61	40	40	3277	39.77	39.91	39.81	39.96	39.96	39.97	39.94
CLAU_1571	Dihydrolipoamide dehydrogenase	0.00	63	33	33	4682	39.62	39.90	39.86	39.93	39.93	39.91	39.91
CLAU_1531	Chaperonin	0.00	84	43	43	5458	39.49	39.79	39.68	39.16	39.19	39.50	39.50
CLAU_1567	Dihydropteroate synthase DHPS	0.00	79	22	22	1922	39.33	39.50	39.50	39.33	39.42	39.49	39.46
CLAU_1569	CO dehydrogenase/acetyl-CoA synthase delta	0.00	90	23	23	4465	38.92	39.09	38.98	38.94	39.02	39.01	39.00
CLAU_0099	Aldehyde ferredoxin oxidoreductase	0.00	60	46	40	2499	39.38	39.18	39.10	38.67	38.83	38.60	38.97
CLAU_2523	Cell wall binding repeat 2-containing protein	0.00	61	31	30	1361	38.22	38.29	38.37	38.61	38.53	38.57	38.45
CLAU_1572	Methylenetetrahydrofolate reductase	0.00	55	14	14	1404	38.11	38.20	38.18	38.25	38.29	38.23	38.22
CLAU_0089	Aldehyde ferredoxin oxidoreductase	0.00	57	36	30	2290	37.93	38.04	37.91	38.45	38.61	38.21	38.13
CLAU_1915	Hypothetical protein	0.00	70	23	23	2412	38.03	38.29	38.18	38.08	38.08	38.06	38.08
CLAU_2947	Pyruvate ferredoxin/flavodoxin oxidoreductase	0.00	48	53	52	1721	38.02	38.15	38.01	37.96	38.13	38.03	38.03
CLAU_2718	NADH dehydrogenase (quinone)	0.00	64	34	34	2470	38.11	38.07	37.92	37.78	37.85	37.86	37.89
CLAU_3861	Alcohol dehydrogenase	0.00	35	10	9	336	37.36	36.42	37.06	38.46	38.32	38.37	37.84
CLAU_3829	Deoxyribose-phosphate aldolase	0.00	68	15	15	1435	37.63	37.53	37.70	37.76	37.89	37.87	37.73
CLAU_2721	Fe-only hydrogenase	0.00	65	25	25	920	37.92	37.83	37.72	37.67	37.69	37.67	37.71
CLAU_1574	Bifunctional methylenetetrahydrofolate	0.00	67	20	20	743	37.59	37.67	37.52	37.86	37.72	37.70	37.69
CAETHG_05115	mob protein	0.01	5	2	2	12	28.15	29.50	28.92	27.11	28.17	26.99	28.16
CAETHG_05090	replication protein	0.01	11	3	3	11	26.92	27.41	25.45	30.37	28.52	27.96	27.69

[a]False-discovery rate (proteome filtering via Percolator); [b]Peptide sequence coverage percentage per specific protein; [c]Number of distinct peptide sequences identified, includes shared peptides; [d]Number of peptides uniquely mapping to each protein, non-shared peptides; [e]Peptide spectrum matches (raw number of MS/MS spectra collected for all peptides of a given protein); [f]Protein abundance in Log2 units; [g]Median log2 abundance across all six samples.

Supplemental Table S2. Proteomics analysis of *Clostridium autoethanogenum* DSM10061

		a	b	c	d	e		f	f	f	f	f	f	g	h	i	j	k
**Locus**	**Desc**	**qval**	**SeqCov(%)**	**Pep**	**UqPep**	**SpC**	**amino acid length**	**Lag 1**	**Lag 2**	**Lag 3**	**Log 1**	**Log 2**	**Log 3**	**median abundance**	**BH FDR 0.05 Sig**	**-log10 pval**	**BH05 pval**	**Diff Lag - Log**
CLAU_1576	Formate-tetrahydrofolate ligase	0.000	61	37	37	5032	558	40.90	41.06	41.03	40.97	40.96	40.95	40.97		0.30	0.6442	0.04
CLAU_2492	Cell wall binding repeat 2-containing prc	0.000	56	58	58	12331	1187	40.42	40.65	40.88	40.68	40.59	40.67	40.66		0.01	0.9891	0.00
CLAU_1794	Alcohol dehydrogenase	0.000	68	25	23	4016	397	39.38	39.51	39.29	40.55	40.42	40.50	39.97	+	3.91	0.0107	-1.10
CLAU_1568	CO dehydrogenase/acetyl-CoA synthase	0.000	65	33	33	3813	450	39.92	40.11	39.81	39.95	39.94	39.94	39.94		0.01	0.9829	0.00
CLAU_1566	CO dehydrogenase/acetyl-CoA synthase	0.000	61	40	40	3277	708	39.77	39.91	39.81	39.96	39.96	39.97	39.94		1.48	0.1183	-0.13
CLAU_1571	Dihydrolipoamide dehydrogenase	0.000	63	33	33	4682	477	39.62	39.90	39.86	39.93	39.93	39.91	39.91		0.67	0.3692	-0.13
CLAU_1531	Chaperonin	0.000	84	43	43	5458	544	39.49	39.79	39.68	39.16	39.19	39.50	39.50		1.24	0.1591	0.37
CLAU_1567	Dihydropteroate synthase DHPS	0.000	79	22	22	1922	261	39.33	39.50	39.50	39.33	39.42	39.49	39.46		0.15	0.8121	0.03
CLAU_1569	CO dehydrogenase/acetyl-CoA synthase	0.000	90	23	23	4465	314	38.92	39.09	38.98	38.94	39.02	39.01	39.00		0.04	0.9490	0.01
CLAU_0099	Aldehyde ferredoxin oxidoreductase	0.000	60	46	40	2499	607	39.38	39.18	39.10	38.67	38.83	38.60	38.97		2.07	0.0548	0.52
CLAU_2523	Cell wall binding repeat 2-containing prc	0.000	61	31	30	1361	604	38.22	38.29	38.37	38.61	38.53	38.57	38.45	+	2.31	0.0430	-0.28
CLAU_1572	Methylenetetrahydrofolate reductase	0.000	55	14	14	1404	293	38.11	38.20	38.18	38.25	38.29	38.23	38.22		1.34	0.1403	-0.09
CLAU_0089	Aldehyde ferredoxin oxidoreductase	0.000	57	36	30	2290	607	37.93	38.04	37.91	38.45	38.61	38.21	38.13		1.71	0.0854	-0.46
CLAU_1915	Hypothetical protein	0.000	70	23	23	2412	397	38.03	38.29	38.18	38.08	38.08	38.06	38.08		0.55	0.4494	0.09
CLAU_2947	Pyruvate ferredoxin/flavodoxin oxidore	0.000	48	53	52	1721	1170	38.02	38.15	38.01	37.96	38.13	38.03	38.03		0.11	0.8655	0.02
CLAU_2718	NADH dehydrogenase (quinone)	0.000	64	34	34	2470	599	38.11	38.07	37.92	37.78	37.85	37.86	37.89		1.49	0.1162	0.20
CLAU_3861	Alcohol dehydrogenase	0.000	35	10	9	336	388	37.36	36.42	37.06	38.46	38.32	38.37	37.84		2.16	0.0504	-1.44
CLAU_3829	Deoxyribose-phosphate aldolase	0.000	68	15	15	1435	214	37.63	37.53	37.70	37.76	37.89	37.87	37.73		1.58	0.1022	-0.22
CLAU_2721	Fe-only hydrogenase	0.000	65	25	25	920	462	37.92	37.83	37.72	37.67	37.69	37.67	37.71		1.18	0.1720	0.15
CLAU_1574	Bifunctional methylenetetrahydrofolate	0.000	67	20	20	743	284	37.59	37.67	37.52	37.86	37.72	37.70	37.69		1.18	0.1722	-0.17
CLAU_0370	(2R,3R)-butanediol dehydrogenase	0.000	67	22	22	1068	357	36.96	37.02	37.15	38.15	38.47	38.23	37.65	+	3.43	0.0182	-1.24
CLAU_1579	Carbon-monoxide dehydrogenase (acce	0.000	51	18	18	980	400	37.78	37.83	37.73	37.08	37.11	37.41	37.57	+	2.22	0.0480	0.58
CLAU_3655	Bifunctional acetaldehyde-CoA/alcohol	0.000	64	43	31	1678	870	37.70	37.71	37.63	37.13	37.23	37.36	37.50	+	2.46	0.0389	0.44
CLAU_3828	Cytidine deaminase	0.000	79	9	9	763	132	37.22	37.33	37.23	37.51	37.56	37.68	37.42	+	2.20	0.0485	-0.32
CLAU_1573	Methylene-tetrahydrofolate reductase	0.000	66	14	14	736	216	37.21	37.30	37.12	37.28	37.30	37.26	37.27		0.59	0.4218	-0.07
CLAU_1948	Histone family protein DNA-binding prot	0.000	63	7	7	606	91	36.89	36.75	37.24	37.26	37.38	37.39	37.25		1.19	0.1714	-0.38
CLAU_2829	Pyruvate/phosphate dikinase	0.000	61	46	46	1852	879	36.81	37.06	36.87	37.30	37.27	37.16	37.11		1.72	0.0839	-0.33
CLAU_2174	L-arabinose isomerase	0.000	34	13	13	153	488	36.45	32.47	37.05	37.03	37.03	36.94	36.99		0.51	0.4750	-1.68
CLAU_3832	Pyrimidine-nucleoside phosphorylase	0.000	74	25	25	1991	433	36.82	37.14	36.95	37.06	36.84	36.87	36.91		0.15	0.8143	0.05
CLAU_2291	ATP synthase subunit beta	0.000	68	22	22	1263	464	36.68	36.92	36.84	36.84	37.04	37.02	36.88		0.74	0.3365	-0.15
CLAU_1565	Glycine cleavage system H protein	0.000	47	5	5	280	124	36.59	36.81	37.11	36.92	36.82	36.89	36.86		0.09	0.8843	-0.04
CLAU_2788	Hydroxylamine reductase	0.000	53	22	22	825	521	37.08	37.24	36.88	36.61	36.53	36.81	36.85		1.45	0.1218	0.42
CLAU_2812	Molecular chaperone DnaK	0.000	53	29	29	1070	625	36.75	36.93	36.81	36.47	36.46	36.65	36.70		1.69	0.0862	0.30
CLAU_3275	Acetate kinase	0.000	67	23	23	1602	398	36.30	36.52	36.40	36.80	36.83	36.79	36.66	+	2.46	0.0389	-0.40
CLAU_2713	Formate dehydrogenase alpha subunit	0.000	55	21	21	695	554	36.50	36.50	36.88	37.03	36.68	36.58	36.63		0.30	0.6461	-0.14
CLAU_2983	Flagellin domain protein	0.000	60	23	21	1428	569	35.81	36.36	36.45	37.01	36.75	37.02	36.60		1.52	0.1114	-0.72
CLAU_1976	Glutamine synthetase catalytic region	0.000	43	24	24	684	632	36.95	37.08	36.29	36.57	36.53	36.20	36.55		0.56	0.4433	0.34
CLAU_1581	Dinitrogenase iron-molybdenum cofacto	0.000	83	5	5	303	115	35.64	35.98	36.00	37.02	37.27	37.18	36.51	+	3.13	0.0230	-1.28
CLAU_2289	ATP synthase subunit alpha	0.000	44	24	24	677	504	36.42	36.46	36.42	36.53	36.61	36.59	36.50	+	2.19	0.0495	-0.14
CLAU_2641	ATP-dependent chaperone ClpB	0.000	37	27	25	411	865	36.30	36.34	36.56	36.48	36.44	36.45	36.45		0.28	0.6681	-0.06
CLAU_3339	Glyceraldehyde-3-phosphate dehydroge	0.000	61	19	18	780	351	35.77	35.93	35.61	36.94	36.80	36.76	36.35	+	3.24	0.0206	-1.06

CLAU_0879	Phospho-2-dehydro-3-deoxyheptonate	0.000	55	14	14	778	344	36.27	36.21	36.51	36.28	36.37	36.47	36.33		0.15	0.8133	-0.04
CLAU_1532	Chaperonin	0.000	69	6	6	1027	94	36.31	36.51	36.60	36.14	36.04	36.20	36.26		1.62	0.0972	0.35
CLAU_0906	Aspartate racemase	0.000	83	16	16	1109	230	36.32	36.63	36.48	35.91	35.71	36.16	36.24		1.60	0.1005	0.55
CLAU_1575	Methenyltetrahydrofolate cyclohydrolas	0.000	53	13	13	286	210	36.24	35.90	36.18	36.19	36.19	36.12	36.19		0.22	0.7323	-0.06
CLAU_3144	Electron transport complex protein rnfC	0.000	60	24	24	587	457	36.30	36.31	36.15	36.12	36.19	36.13	36.17		0.88	0.2706	0.11
CLAU_1902	Translation elongation factor G	0.000	61	30	30	1011	689	36.15	36.22	36.09	36.10	36.10	36.07	36.10		0.75	0.3330	0.06
CLAU_0877	3-dehydroquinate synthase	0.000	67	24	24	507	363	36.24	36.28	36.10	35.88	35.90	36.08	36.09		1.41	0.1284	0.25
CLAU_2719	4Fe-4S ferredoxin iron-sulfur binding	0.000	53	15	15	360	251	36.13	36.10	36.09	36.05	35.99	36.00	36.07		1.87	0.0708	0.09
CLAU_3274	Phosphate acetyltransferase	0.000	52	13	13	673	333	35.51	35.75	35.60	36.09	36.14	36.17	35.92	+	2.65	0.0326	-0.51
CLAU_2202	Transcriptional regulator AbrB family	0.000	78	8	8	159	81	35.63	35.88	36.08	35.86	35.70	35.84	35.85		0.17	0.7869	0.06
CLAU_1958	Ribose-phosphate pyrophosphokinase	0.000	53	15	15	643	319	35.45	35.82	35.77	36.07	35.94	35.88	35.85		1.03	0.2170	-0.28
CLAU_3656	Bifunctional acetaldehyde-CoA/alcohol	0.000	22	15	3	652	877	36.06	36.04	35.94	35.43	35.52	35.64	35.79	+	2.61	0.0339	0.48
CLAU_2956	CheA signal transduction histidine kinas	0.000	52	33	33	787	690	35.55	35.73	35.59	35.79	35.77	35.80	35.75		1.38	0.1330	-0.16
CLAU_1539	Glutamate synthase (NADPH) homotetr	0.000	55	31	31	1001	757	35.85	35.96	35.83	35.54	35.51	35.66	35.75		2.15	0.0511	0.31
CLAU_1530	IMP dehydrogenase	0.000	63	25	25	898	484	35.69	35.60	35.52	35.80	35.91	35.87	35.75		1.92	0.0654	-0.26
CLAU_2623	Gamma-glutamyl phosphate reductase	0.000	58	19	19	512	418	35.47	35.58	35.66	35.73	35.68	35.72	35.67		1.15	0.1796	-0.14
CLAU_1583	ParA/MinD ATPase-like protein	0.000	51	11	11	422	276	34.69	35.00	34.85	36.40	36.44	36.19	35.60	+	3.65	0.0140	-1.50
CLAU_1185	Cold-shock DNA-binding domain proteir	0.000	67	3	3	90	67	35.66	35.67	35.83	35.04	35.34	35.47	35.57		1.46	0.1204	0.44
CLAU_1882	Ribosomal protein S5	0.000	67	9	9	354	165	35.64	35.73	35.84	35.49	35.49	35.45	35.57		1.93	0.0654	0.26
CLAU_1908	50S ribosomal protein L7/L12	0.000	54	10	10	589	123	35.46	35.87	35.91	35.58	35.23	35.20	35.52		1.02	0.2187	0.41
CLAU_2683	Argininosuccinate synthase	0.000	46	17	17	743	401	34.90	35.08	35.29	35.77	35.85	35.75	35.52	+	2.41	0.0393	-0.70
CLAU_1732	Rubrerythrin	0.000	53	11	11	541	181	35.93	35.75	35.51	35.16	35.16	35.46	35.49		1.39	0.1302	0.47
CLAU_2322	Fructose-1,6-bisphosphate aldolase clas	0.000	46	13	13	911	310	35.26	35.37	35.37	35.69	35.61	35.56	35.47	+	2.26	0.0459	-0.29
CLAU_2736	Cell shape determining protein MreB/M	0.000	63	17	16	471	336	35.38	35.30	35.25	35.53	35.47	35.60	35.43		1.86	0.0710	-0.22
CLAU_2220	Methionyl-tRNA synthetase	0.000	51	28	28	281	644	35.45	35.54	35.38	35.27	35.47	35.39	35.42		0.47	0.5107	0.08
CLAU_1906	DNA-directed RNA polymerase subunit I	0.000	38	35	35	538	1178	35.29	35.41	35.40	35.23	35.37	35.42	35.39		0.14	0.8198	0.03
CLAU_3831	Phosphopentomutase	0.000	44	17	17	365	393	35.37	35.47	35.23	35.43	35.40	35.37	35.39		0.24	0.7128	-0.04
CLAU_2958	Response regulator receiver protein	0.000	55	6	6	265	120	35.21	35.30	35.47	35.39	35.37	35.47	35.38		0.43	0.5314	-0.08
CLAU_1866	Ribosomal protein L13	0.000	59	7	7	261	150	35.56	35.53	35.56	35.20	35.18	35.22	35.38	+	4.67	0.0046	0.35
CLAU_3314	Translation initiation factor IF-2	0.000	41	20	20	321	700	35.59	35.77	35.90	34.99	35.15	35.11	35.37	+	2.56	0.0349	0.67
CLAU_0949	putative 2,4-dienoyl-CoA reductase (NA	0.000	41	23	23	565	664	35.19	35.18	34.98	35.66	35.61	35.54	35.37	+	2.50	0.0379	-0.49
CLAU_2717	NADH-quinone oxidoreductase E subuni	0.000	67	9	9	212	161	34.86	34.92	35.38	35.40	35.50	35.34	35.36		0.99	0.2278	-0.36
CLAU_0534	NADPH-dependent butanol dehydrogen	0.000	23	8	7	901	388	35.04	35.29	34.30	35.37	35.50	35.86	35.33		0.99	0.2271	-0.70
CLAU_1900	30S ribosomal protein S10	0.000	59	7	7	257	102	35.40	35.27	35.70	35.25	35.30	35.32	35.31		0.58	0.4294	0.17
CLAU_1896	50S Ribosomal protein L2	0.000	40	8	8	497	277	34.48	35.48	35.85	35.92	34.88	35.06	35.27		0.01	0.9859	-0.02
CLAU_1313	Aspartate-semialdehyde dehydrogenase	0.000	68	24	24	553	330	35.31	35.37	35.37	35.00	35.09	35.14	35.23	+	2.41	0.0393	0.27
CLAU_1433	Trigger factor	0.000	43	21	21	534	430	35.06	35.09	35.40	35.20	35.23	35.20	35.20		0.09	0.8926	-0.03
CLAU_0388	Acetolactate synthase biosynthetic type	0.000	39	20	20	597	558	35.41	35.60	35.40	34.97	34.79	34.83	35.19	+	2.69	0.0309	0.61
CLAU_0873	Shikimate dehydrogenase	0.000	76	14	14	385	271	35.42	35.35	35.25	34.98	34.93	35.12	35.19		1.93	0.0654	0.33
CLAU_2677	O-acetylhomoserine/O-acetylserine sulf	0.000	48	17	17	483	427	35.64	35.83	35.36	34.76	34.64	35.00	35.18		2.03	0.0585	0.81
CLAU_1887	Ribosomal protein L5	0.000	60	11	11	399	181	35.50	35.42	35.25	35.04	35.08	35.04	35.17		1.96	0.0631	0.34
CLAU_1578	Carbon-monoxide dehydrogenase (acce	0.000	63	10	10	175	227	37.15	37.38	33.98	34.30	35.78	34.53	35.16		0.47	0.5038	1.30
CLAU_1763	C_GCAxxG_C_C family protein	0.000	68	13	13	306	215	35.02	35.10	35.04	35.20	35.23	35.19	35.15	+	2.33	0.0415	-0.15
CLAU_2205	Phage shock protein A	0.000	55	16	16	360	216	34.89	34.78	34.87	35.28	35.34	35.48	35.09	+	2.80	0.0283	-0.52
CLAU_1154	ATP-dependent Clp protease proteolytic	0.000	30	7	7	132	194	32.75	33.32	32.76	37.13	37.00	36.85	35.09	+	4.41	0.0046	-4.05
CLAU_3333	Aspartate transaminase	0.000	57	18	18	535	397	34.85	34.85	35.09	35.06	35.14	35.15	35.08		1.03	0.2170	-0.19

CLAU_0210	4-hydroxy-3-methylbut-2-enyl diphosph	0.000	40	24	24	475	637	34.97	35.13	35.01	35.07	35.10	35.07	35.07		0.37	0.5866	-0.04
CLAU_1872	DNA-directed RNA polymerase subunit a	0.000	75	22	22	312	315	35.02	35.14	34.88	35.09	35.04	35.08	35.06		0.30	0.6446	-0.06
CLAU_1875	Ribosomal protein S13	0.000	36	6	6	139	125	35.19	35.15	35.24	34.84	34.90	34.91	35.03	+	3.10	0.0231	0.31
CLAU_0117	Ketol-acid reductoisomerase	0.000	64	16	16	350	337	35.21	35.22	35.26	34.59	34.54	34.84	35.03	+	2.44	0.0390	0.57
CLAU_3229	Cell division protein FtsZ	0.000	56	17	17	830	369	34.99	35.00	34.94	34.94	35.06	35.02	35.00		0.31	0.6407	-0.03
CLAU_0911	CoA-disulfide reductase	0.000	46	31	31	759	839	35.34	35.41	35.07	34.81	34.28	34.82	34.95		1.44	0.1245	0.64
CLAU_1844	Thioredoxin-disulfide reductase	0.000	73	19	19	463	285	34.86	35.17	34.80	34.77	34.95	35.07	34.91		0.03	0.9589	0.01
CLAU_0888	Methyl-accepting chemotaxis sensory tr	0.000	27	7	6	259	347	34.87	34.83	34.85	34.95	35.01	34.89	34.88		1.28	0.1512	-0.10
CLAU_0096	Molybdenum co-factor synthesis domai	0.000	43	20	20	621	635	34.50	34.63	34.55	35.28	35.44	34.98	34.81		2.07	0.0554	-0.67
CLAU_0280	Beta-lactamase domain protein	0.000	47	17	17	395	391	35.14	35.31	35.00	34.58	34.53	34.59	34.80	+	2.51	0.0379	0.58
CLAU_1903	Ribosomal protein S7	0.000	60	8	8	225	156	34.92	34.80	34.92	34.60	34.79	34.67	34.80		1.32	0.1439	0.19
CLAU_0569	Ornithine carbamoyltransferase	0.000	44	13	13	246	333	33.83	33.98	34.63	34.89	35.00	34.90	34.76		1.47	0.1202	-0.78
CLAU_0118	Dihydroxy-acid dehydratase	0.000	44	24	24	364	552	34.90	34.98	34.95	34.41	34.29	34.61	34.76	+	2.21	0.0485	0.51
CLAU_1757	Pyridoxal biosynthesis lyase pdxS	0.000	48	13	13	365	289	34.67	34.95	34.82	34.61	34.74	34.73	34.74		0.59	0.4210	0.12
CLAU_2300	S-adenosylmethionine synthase	0.000	43	14	12	357	391	34.90	34.81	34.74	34.48	34.71	34.69	34.73		1.03	0.2185	0.19
CLAU_3301	Ribosomal protein S2	0.000	32	9	9	326	235	34.83	34.77	34.98	34.66	34.66	34.64	34.72		1.52	0.1114	0.21
CLAU_1529	GMP synthase (glutamine-hydrolyzing)	0.000	53	22	22	423	510	34.67	34.67	34.61	34.75	34.81	34.72	34.70		1.53	0.1105	-0.11
CLAU_0054	Chaperone protein htpG	0.000	48	28	28	428	624	34.88	34.83	34.93	34.49	34.47	34.55	34.69	+	3.25	0.0204	0.38
CLAU_1926	ATPase AAA-2 domain protein	0.000	50	34	32	533	813	33.86	34.29	34.03	35.09	35.01	35.03	34.65	+	2.82	0.0283	-0.98
CLAU_1895	Ribosomal protein S19	0.000	54	6	6	204	98	34.75	34.67	34.78	34.37	34.51	34.54	34.61		1.87	0.0708	0.26
CLAU_1714	Glyceraldehyde-3-phosphate dehydroge	0.000	59	16	15	610	335	34.34	34.60	34.61	34.59	34.90	34.82	34.61		0.92	0.2530	-0.25
CLAU_2680	Periplasmic subunit family 3	0.000	50	11	11	337	274	34.34	34.44	34.57	34.61	34.69	34.68	34.59		1.38	0.1331	-0.21
CLAU_1873	Ribosomal protein S4	0.000	55	11	11	372	206	34.71	34.54	34.82	34.47	34.57	34.57	34.57		0.81	0.3052	0.15
CLAU_1871	50S ribosomal protein L17	0.000	45	6	6	170	113	34.44	34.61	34.71	34.50	34.56	34.52	34.54		0.30	0.6442	0.06
CLAU_1893	Ribosomal protein S3	0.000	48	9	9	290	223	34.57	34.67	34.75	34.45	34.49	34.32	34.53		1.54	0.1099	0.24
CLAU_3302	Elongation factor Ts	0.000	43	16	16	286	306	34.19	34.24	34.28	34.99	34.92	34.78	34.53	+	3.22	0.0206	-0.66
CLAU_2288	ATP synthase subunit delta	0.000	35	8	8	208	181	34.47	34.57	34.86	34.57	34.26	34.32	34.52		0.76	0.3269	0.25
CLAU_2976	Flagellin domain protein	0.000	22	7	5	188	570	34.07	34.33	34.36	34.67	34.68	34.85	34.52		1.93	0.0651	-0.48
CLAU_3055	putative signal transduction protein witl	0.000	58	22	22	306	543	34.53	34.49	34.36	34.47	34.57	34.58	34.51		0.57	0.4309	-0.08
CLAU_3760	Dipeptidase	0.000	46	17	17	630	463	34.40	34.46	34.33	34.67	34.69	34.56	34.51		1.94	0.0651	-0.24
CLAU_0308	Phosphoribosylaminoimidazolecarboxar	0.000	59	23	23	253	391	34.39	34.61	34.34	35.06	35.37	34.28	34.50		0.61	0.4046	-0.46
CLAU_1845	Thioredoxin	0.000	39	3	3	153	105	34.40	34.70	34.53	34.44	34.32	34.81	34.49		0.04	0.9506	0.02
CLAU_0231	Arginine biosynthesis bifunctional prote	0.006	20	7	7	21	406	34.49	34.30	34.47	34.47	34.51	34.26	34.47		0.02	0.9696	0.01
CLAU_1647	Aspartate kinase	0.000	37	12	12	295	400	34.52	34.60	34.55	34.28	34.28	34.41	34.47		2.04	0.0567	0.23
CLAU_1710	Enolase	0.000	73	21	21	502	432	34.06	34.31	34.38	34.56	34.67	34.55	34.47		1.52	0.1114	-0.34
CLAU_2493	Cell wall binding repeat 2-containing prc	0.000	42	30	30	463	1103	33.76	34.05	34.47	34.69	34.46	34.47	34.47		0.95	0.2404	-0.45
CLAU_0207	Aspartate transaminase	0.000	52	20	20	372	398	34.63	34.68	34.56	34.07	34.24	34.35	34.46		1.98	0.0613	0.40
CLAU_0940	NADPH-dependent FMN reductase	0.000	34	7	7	139	187	34.44	34.47	34.31	34.25	34.54	34.51	34.46		0.09	0.8862	-0.03
CLAU_1859	Methylaspartate mutase S chain	0.001	25	3	3	36	134	33.53	34.03	34.27	34.88	34.65	34.64	34.46		1.55	0.1079	-0.78
CLAU_2959	CheW protein	0.000	52	6	6	398	133	34.41	34.59	34.48	34.09	34.32	34.43	34.42		0.88	0.2726	0.21
CLAU_1179	Threonine synthase	0.000	50	22	22	390	496	34.42	34.41	34.45	34.37	34.41	34.45	34.42		0.25	0.6916	0.02
CLAU_2378	Pyruvate kinase	0.000	45	20	20	453	585	34.07	34.25	34.28	34.54	34.76	34.64	34.41		2.09	0.0538	-0.45
CLAU_2394	Methyl-accepting chemotaxis sensory tr	0.000	37	22	21	376	570	34.64	34.71	34.25	34.00	34.34	34.42	34.38		0.66	0.3757	0.28
CLAU_2298	Cell shape determining protein MreB/M	0.000	44	12	11	209	343	34.34	34.37	33.98	34.35	34.44	34.37	34.36		0.54	0.4537	-0.16
CLAU_3521	Threonine ammonia-lyase	0.000	45	16	16	416	403	34.63	34.89	34.57	34.13	33.91	34.08	34.35	+	2.28	0.0450	0.66
CLAU_3279	Acyl carrier protein	0.000	31	2	2	120	77	33.99	33.97	34.35	34.53	34.56	34.34	34.35		1.24	0.1593	-0.37

CLAU_0886	UPF0210 protein	0.000	51	17	17	292	452	34.28	34.40	34.62	34.05	34.20	34.39	34.34		0.72	0.3471	0.22
CLAU_2645	Phosphoenolpyruvate carboxykinase (AT	0.000	47	20	20	212	522	34.05	33.97	33.84	34.74	34.58	34.64	34.32	+	3.09	0.0231	-0.70
CLAU_2924	Carbon-monoxide dehydrogenase cataly	0.000	36	19	19	329	626	34.64	34.54	34.75	33.99	33.71	34.06	34.30	+	2.38	0.0407	0.72
CLAU_1432	ATP-dependent Clp protease proteolytic	0.000	37	6	6	219	194	34.16	34.24	34.14	34.34	34.41	34.45	34.29		2.11	0.0531	-0.22
CLAU_0784	Hydroxylamine reductase	0.000	50	21	21	306	567	34.63	34.86	34.31	34.01	33.80	34.26	34.29		1.30	0.1472	0.58
CLAU_0885	ACT domain-containing protein	0.000	40	5	5	164	89	34.28	34.28	34.57	33.87	33.90	34.23	34.26		1.18	0.1731	0.38
CLAU_1312	Dihydrodipicolinate synthase	0.000	38	8	8	145	293	34.24	34.39	34.18	34.25	34.06	34.50	34.25		0.00	1.0000	0.00
CLAU_2236	Isoleucyl-tRNA synthetase	0.000	33	29	29	406	1035	33.97	34.10	34.25	34.51	34.48	34.24	34.25		1.21	0.1649	-0.30
CLAU_2305	Protein translocase subunit secA	0.000	41	31	31	518	837	34.09	34.18	34.08	34.27	34.30	34.28	34.23		2.14	0.0516	-0.17
CLAU_1431	ATP-dependent Clp protease ATP-bindir	0.000	38	15	15	400	429	34.10	34.00	34.19	34.25	34.31	34.58	34.22		1.15	0.1788	-0.28
CLAU_3158	Serine hydroxymethyltransferase	0.000	47	17	17	384	414	34.22	34.22	34.16	34.21	34.33	34.15	34.22		0.20	0.7490	-0.03
CLAU_3368	Transcriptional regulator	0.095	2	1	1	3	365	34.11	34.23	34.42	34.17	34.18	34.27	34.21		0.19	0.7701	0.05
CLAU_2573	Type II secretion system protein E	0.179	2	1	1	1	558	32.53	34.54	33.83	33.97	34.47	34.42	34.20		0.46	0.5111	-0.65
CLAU_3207	Microcompartments protein	0.000	59	5	2	253	98	34.40	34.21	34.40	33.95	33.90	34.12	34.17		1.71	0.0854	0.35
CLAU_3206	Microcompartments protein	0.000	51	4	1	230	96	34.35	34.18	34.38	33.90	33.82	34.09	34.14		1.65	0.0933	0.37
CLAU_1770	Microcompartments protein	0.000	51	4	1	230	96	34.35	34.18	34.38	33.90	33.82	34.09	34.14		1.65	0.0933	0.37
CLAU_2359	1-deoxy-D-xylulose-5-phosphate syntha	0.000	46	13	13	207	313	34.11	34.19	34.05	34.16	34.10	34.06	34.11		0.07	0.9082	0.01
CLAU_1083	Nicotinate-nucleotide-dimethylbenzimic	0.000	36	11	11	258	358	34.08	34.23	34.15	34.00	34.00	34.07	34.08		1.24	0.1593	0.13
CLAU_1553	Pyruvate carboxylase	0.000	30	28	28	445	1145	34.19	34.09	34.02	33.90	34.06	34.09	34.08		0.47	0.5075	0.08
CLAU_1091	Cobyric acid synthase	0.000	46	21	21	378	505	34.00	33.96	34.10	34.05	34.23	34.15	34.08		0.86	0.2807	-0.12
CLAU_2811	Heat shock protein grpE	0.000	59	11	11	228	216	34.14	34.31	34.24	33.73	33.66	33.99	34.07		1.76	0.0805	0.44
CLAU_0874	Prephenate dehydratase	0.000	33	14	14	421	378	34.15	34.11	34.07	33.93	33.78	34.00	34.04		1.40	0.1286	0.21
CLAU_1713	Phosphoglycerate kinase	0.000	54	15	15	296	398	33.73	33.97	34.60	33.91	34.18	34.09	34.03		0.05	0.9343	0.04
CLAU_1880	Ribosomal protein L15	0.000	47	7	7	147	146	34.06	34.08	34.19	33.93	33.94	33.86	34.00		1.86	0.0708	0.20
CLAU_0729	Glutaminyl-tRNA synthetase	0.000	35	16	16	184	553	33.98	33.91	34.03	34.02	33.95	34.00	33.99		0.15	0.8119	-0.02
CLAU_3146	Electron transport complex subunit G	0.000	51	9	9	258	185	34.00	33.98	33.97	33.90	34.06	34.10	33.99		0.23	0.7169	-0.04
CLAU_3910	Deoxyribose-phosphate	0.000	54	11	11	243	294	33.90	33.84	33.69	34.14	34.11	34.07	33.99		1.97	0.0622	-0.30
CLAU_1712	Triosephosphate isomerase	0.000	74	14	14	226	248	33.98	34.00	33.96	33.89	34.00	33.98	33.98		0.26	0.6854	0.02
CLAU_1617	Hypothetical protein	0.019	10	2	2	6	317	34.34	34.26	34.16	33.41	33.43	33.78	33.97	+	2.26	0.0459	0.71
CLAU_1384	Cell wall binding repeat 2-containing prc	0.000	29	35	33	541	2137	34.02	34.05	34.30	33.89	33.87	33.84	33.96		1.34	0.1416	0.26
CLAU_1782	Pyruvate formate-lyase PFL	0.000	35	22	22	501	849	34.49	34.19	33.99	33.69	33.79	33.91	33.95		1.26	0.1552	0.43
CLAU_2304	Sigma-54 modulation protein/ribosoma	0.000	42	7	7	165	175	33.92	33.54	34.03	33.89	33.98	34.08	33.95		0.41	0.5483	-0.15
CLAU_2512	Cell wall binding repeat 2-containing prc	0.000	35	15	15	549	519	33.92	33.74	33.84	33.98	34.03	34.05	33.95		1.54	0.1099	-0.19
CLAU_3923	Response regulator receiver protein	0.000	79	16	16	399	291	33.72	33.84	33.84	34.04	34.08	34.07	33.94	+	2.49	0.0379	-0.26
CLAU_1891	50S ribosomal protein L29	0.000	51	4	4	118	70	33.95	33.95	34.24	33.88	33.88	33.87	33.92		0.81	0.3023	0.17
CLAU_1857	Methylaspartate mutase E subunit	0.000	43	18	18	262	485	33.93	33.94	33.84	33.90	33.86	34.07	33.92		0.22	0.7333	-0.04
CLAU_0328	L-aspartate oxidase	0.000	44	21	21	205	598	34.86	34.75	34.38	33.31	32.07	33.41	33.90		1.72	0.0839	1.73
CLAU_3319	30S ribosomal protein S15	0.000	41	5	5	149	87	33.44	34.02	34.15	33.89	33.88	33.88	33.89		0.02	0.9719	-0.01
CLAU_0392	NADH-dependent butanol dehydrogena	0.000	36	13	13	280	391	33.74	33.58	33.60	34.07	34.16	34.02	33.88	+	2.62	0.0334	-0.44
CLAU_1304	50S ribosomal protein L20	0.000	18	2	2	63	119	33.91	33.99	33.95	33.61	33.84	33.57	33.88		1.47	0.1196	0.28
CLAU_1535	Hydrogenase Fe-only	0.000	38	16	16	269	559	33.77	34.12	34.35	33.97	33.71	33.74	33.87		0.66	0.3757	0.27
CLAU_1935	Transcription elongation factor GreA	0.000	63	9	8	138	159	33.84	33.89	33.85	34.08	33.84	33.17	33.85		0.24	0.7154	0.16
CLAU_1894	Ribosomal protein L22	0.000	45	5	5	335	111	33.75	34.14	34.02	33.92	33.70	33.65	33.84		0.68	0.3644	0.21
CLAU_3291	50S ribosomal protein L19	0.000	40	5	5	218	115	33.80	33.96	34.04	33.69	33.80	33.66	33.80		1.24	0.1599	0.22
CLAU_1907	DNA-directed RNA polymerase subunit I	0.000	36	33	33	365	1242	33.66	33.79	33.84	33.61	33.87	33.80	33.80		0.01	0.9845	0.00
CLAU_2653	Dihydropteroate synthase	0.000	53	12	12	226	272	33.57	33.69	33.72	33.92	33.98	33.86	33.79		1.97	0.0621	-0.26

CLAU_0550	Molybdenum cofactor sulfurase-like pro	0.000	58	6	6	284	143	33.88	33.75	33.79	33.72	33.69	33.86	33.77		0.31	0.6342	0.05
CLAU_1865	Ribosomal protein S9	0.000	52	6	6	70	130	33.80	33.74	34.06	33.76	33.77	33.67	33.77		0.58	0.4294	0.13
CLAU_2868	Phosphoribosylaminoimidazolesuccinoc	0.000	45	11	11	448	235	33.71	33.86	33.90	33.78	33.74	33.58	33.76		0.66	0.3739	0.12
CLAU_1885	Ribosomal protein S8	0.000	61	7	7	160	132	33.49	33.48	33.90	33.76	33.98	33.76	33.76		0.60	0.4125	-0.21
CLAU_3384	Methyl-accepting chemotaxis sensory tr	0.000	35	18	17	265	463	33.62	33.87	33.87	33.96	33.51	33.43	33.75		0.34	0.6085	0.15
CLAU_2919	3-isopropylmalate dehydratase large sul	0.000	33	10	10	230	421	34.07	34.20	34.15	33.02	33.03	33.37	33.72	+	2.93	0.0264	1.00
CLAU_2084	Seryl-tRNA synthetase	0.000	46	17	17	181	426	33.67	33.79	33.72	33.70	33.98	33.72	33.72		0.31	0.6390	-0.07
CLAU_3077	DivIVA domain-containing protein	0.000	53	10	10	238	227	33.67	33.63	33.76	33.66	33.78	33.76	33.72		0.36	0.5902	-0.05
CLAU_2722	4Fe-4S dicluster domain	0.000	35	5	5	82	205	33.68	33.77	33.70	33.69	33.75	33.72	33.71		0.03	0.9557	0.00
CLAU_2716	Protein fdhD	0.000	27	7	7	56	262	33.36	31.35	33.69	34.48	33.97	33.73	33.71		0.76	0.3289	-1.26
CLAU_1564	Ferredoxin	0.000	26	14	14	241	644	33.94	35.60	33.63	33.62	33.70	33.69	33.70		0.52	0.4719	0.72
CLAU_2590	Tubulin-like protein	0.000	38	43	43	372	1135	33.68	33.71	33.31	33.92	33.72	33.68	33.70		0.63	0.3961	-0.21
CLAU_2490	Cell wall binding repeat 2-containing prc	0.000	55	10	10	203	351	33.19	33.51	33.65	33.73	33.74	33.73	33.69		0.97	0.2319	-0.28
CLAU_2287	ATP synthase subunit b	0.000	41	7	7	245	163	33.55	33.70	33.61	33.71	33.67	33.75	33.69		0.85	0.2859	-0.09
CLAU_0035	Aminotransferase	0.000	48	14	14	273	392	33.80	33.92	33.79	33.22	33.27	33.57	33.68		1.84	0.0721	0.48
CLAU_0552	Molybdenum cofactor synthesis domain	0.000	57	9	9	190	162	33.61	33.65	33.43	33.70	33.74	33.77	33.68		1.15	0.1789	-0.17
CLAU_2279	Uracil phosphoribosyl transferase	0.000	70	11	11	204	209	33.08	33.33	33.47	34.09	34.08	33.88	33.68	+	2.26	0.0459	-0.72
CLAU_1536	NADH dehydrogenase (quinone)	0.000	50	26	26	300	624	33.80	34.10	34.06	33.46	33.40	33.53	33.67	+	2.18	0.0499	0.52
CLAU_0567	Carbamoyl-phosphate synthase large su	0.000	28	23	23	324	1068	32.84	33.04	33.45	33.85	33.97	33.86	33.65		1.89	0.0687	-0.78
CLAU_1910	Ribosomal protein L1	0.000	41	9	9	258	229	33.76	33.81	34.01	33.53	33.50	33.49	33.65		1.99	0.0611	0.35
CLAU_0336	Methyl-accepting chemotaxis sensory tr	0.000	39	18	17	316	682	33.62	33.67	33.55	33.62	33.70	33.72	33.65		0.65	0.3806	-0.07
CLAU_2872	Bifunctional purine biosynthesis protein	0.000	38	17	17	384	499	33.63	33.72	33.61	33.54	33.74	33.58	33.62		0.18	0.7756	0.03
CLAU_3162	Phosphoribosylformylglycinamidine syn	0.000	46	49	49	458	1256	33.44	33.45	33.53	33.82	33.85	33.66	33.60		2.01	0.0601	-0.30
CLAU_1562	Hypothetical protein	0.000	40	4	4	135	114	33.20	33.09	33.33	34.04	33.91	33.82	33.58	+	2.79	0.0283	-0.72
CLAU_1392	Nitroreductase	0.000	39	7	7	184	185	33.32	33.05	33.36	33.89	33.77	33.90	33.57	+	2.35	0.0407	-0.61
CLAU_1934	Lysyl-tRNA synthetase	0.000	38	17	17	268	503	33.41	33.38	33.27	33.71	33.78	33.72	33.56	+	2.88	0.0283	-0.38
CLAU_2031	Putrescine carbamoyltransferase	0.000	34	11	11	197	338	30.56	31.84	31.90	35.58	35.55	35.22	33.56	+	3.05	0.0237	-4.02
CLAU_0856	Rubrerythrin	0.000	49	5	5	91	142	33.59	33.60	33.73	33.21	33.17	33.50	33.55		1.42	0.1259	0.35
CLAU_1589	ABC-type transporter related protein	0.000	45	10	10	265	243	32.49	32.97	32.85	34.34	34.42	34.12	33.55	+	3.07	0.0237	-1.52
CLAU_2684	Argininosuccinate lyase	0.000	33	14	14	201	438	33.00	33.05	33.44	35.24	33.62	34.64	33.53		1.27	0.1535	-1.34
CLAU_1984	Asparaginyl-tRNA synthetase	0.000	47	18	18	245	463	33.79	33.37	32.97	33.58	33.60	33.46	33.52		0.28	0.6612	-0.17
CLAU_1226	Aspartyl-tRNA synthetase	0.000	46	23	23	279	590	33.44	33.52	33.49	33.51	33.62	33.40	33.50		0.15	0.8178	-0.03
CLAU_3320	Polynucleotide phosphorylase	0.000	27	18	18	319	714	33.78	33.36	32.78	33.59	33.42	33.58	33.50		0.31	0.6397	-0.22
CLAU_1960	Regulatory protein SpoVG	0.000	70	6	6	213	96	32.66	33.31	33.53	33.60	33.45	33.53	33.49		0.61	0.4058	-0.36
CLAU_2237	Transcriptional regulator LacI family	0.000	48	12	12	291	332	33.59	33.60	33.05	33.40	33.49	33.46	33.48		0.07	0.9082	-0.04
CLAU_3145	Electron transport complex subunit D	0.000	13	3	3	96	331	33.51	33.52	33.36	33.36	33.59	33.44	33.48		0.00	1.0000	0.00
CLAU_3334	Phosphotransferase system phosphocar	0.000	47	4	4	198	86	32.95	33.17	33.84	33.48	33.46	33.49	33.47		0.23	0.7217	-0.16
CLAU_0866	Fructose-1,6-bisphosphatase class 3	0.000	39	22	22	253	666	33.24	33.19	33.17	33.70	33.90	33.77	33.47	+	3.16	0.0223	-0.59
CLAU_0246	CBS domain-containing protein	0.000	73	6	6	105	125	33.44	33.44	33.42	33.50	33.49	33.58	33.47		1.43	0.1252	-0.09
CLAU_2009	Adenylosuccinate synthetase	0.000	45	17	17	170	428	33.27	33.29	33.43	33.67	33.77	33.50	33.47		1.56	0.1062	-0.32
CLAU_0747	ABC-type transporter related protein	0.000	30	7	7	218	243	32.38	32.90	32.78	34.24	34.32	34.03	33.47	+	2.96	0.0257	-1.51
CLAU_1939	Peptidase M41 FtsH domain protein	0.000	30	17	17	365	602	33.43	33.47	33.57	33.50	33.36	33.43	33.45		0.44	0.5257	0.06
CLAU_2308	Glutamate dehydrogenase (NADP(+))	0.000	35	13	13	171	448	33.59	33.78	33.54	33.19	33.33	33.35	33.45		1.76	0.0805	0.35
CLAU_1884	Ribosomal protein L6	0.000	35	6	6	195	214	33.39	33.65	33.86	33.49	33.33	33.33	33.44		0.79	0.3132	0.25
CLAU_0875	Chorismate synthase	0.000	49	14	14	297	360	33.23	33.71	33.76	33.51	33.22	33.36	33.44		0.47	0.5100	0.20
CLAU_2228	ATPase-like ParA/MinD	0.000	46	12	12	200	282	33.53	33.57	33.49	33.32	33.38	33.36	33.44	+	2.43	0.0390	0.18

CLAU_2765	putative cobalamin-binding protein	0.000	40	7	7	140	210	33.51	33.51	33.52	33.35	32.99	33.10	33.43		1.58	0.1029	0.37
CLAU_1307	Threonyl-tRNA synthetase	0.000	32	17	17	203	637	33.46	33.47	33.34	33.39	33.51	33.11	33.43		0.28	0.6690	0.09
CLAU_1173	ABC-type transporter periplasmic subun	0.000	45	8	8	238	268	33.19	33.41	33.43	33.51	33.39	33.53	33.42		0.69	0.3626	-0.13
CLAU_3579	DGC domain protein	0.000	47	5	5	167	133	32.55	32.68	32.77	34.07	34.10	34.14	33.42	+	4.55	0.0046	-1.44
CLAU_1270	UDP-N-acetyl-D-glucosamine dehydroge	0.000	47	15	15	186	438	33.31	33.47	33.45	33.35	33.46	33.36	33.41		0.12	0.8513	0.02
CLAU_3329	Stage V sporulation protein S	0.000	38	2	2	77	86	33.05	33.52	33.56	33.26	33.36	33.44	33.40		0.05	0.9399	0.02
CLAU_3204	Acetaldehyde dehydrogenase (acetylati	0.000	47	14	14	251	505	33.73	33.63	33.48	33.13	33.13	33.30	33.39		2.01	0.0601	0.43
CLAU_1772	Acetaldehyde dehydrogenase (acetylati	0.000	53	16	16	255	517	33.73	33.63	33.48	33.13	33.13	33.30	33.39		2.01	0.0601	0.43
CLAU_1413	Transcription elongation factor GreA	0.000	63	9	8	209	154	33.31	33.25	33.44	33.37	33.36	33.46	33.37		0.42	0.5430	-0.06
CLAU_3839	Xylose isomerase	0.000	30	11	11	153	440	33.39	33.26	33.22	33.34	33.38	33.45	33.36		0.76	0.3285	-0.10
CLAU_1233	Delta(24)-sterol C-methyltransferase	0.013	12	3	3	9	282	33.39	33.21	33.30	33.32	33.55	33.48	33.36		0.81	0.3037	-0.15
CLAU_1856	Methylaspartate ammonia-lyase	0.000	32	13	13	275	413	33.54	33.25	33.32	33.49	33.23	33.38	33.35		0.01	0.9873	0.00
CLAU_2456	Glutamate-1-semialdehyde 2,1-aminom	0.000	27	9	9	257	424	33.23	33.15	33.22	33.49	33.44	33.47	33.34	+	3.11	0.0231	-0.27
CLAU_2178	Alpha-L-arabinofuranosidase domain pr	0.000	37	13	13	148	510	33.26	33.30	33.05	33.36	33.60	33.44	33.33		1.18	0.1722	-0.26
CLAU_1269	Oxidoreductase	0.000	45	13	13	222	367	33.23	33.27	32.87	33.38	33.45	33.45	33.33		1.10	0.1940	-0.30
CLAU_1892	50S ribosomal protein L16	0.000	28	4	4	122	147	33.29	33.50	33.50	33.18	33.34	33.15	33.32		1.06	0.2087	0.21
CLAU_3219	Ribocuclease J	0.000	47	19	19	246	555	33.44	33.51	33.33	33.16	33.30	33.25	33.32		1.34	0.1415	0.19
CLAU_2918	2-isopropylmalate synthase	0.000	48	19	19	307	510	33.47	33.88	33.70	32.75	32.66	33.12	33.30		1.99	0.0611	0.84
CLAU_1899	Ribosomal protein L3	0.000	41	8	8	167	209	33.25	33.53	33.57	33.32	33.05	32.97	33.29		1.08	0.2012	0.34
CLAU_2766	Uroporphyrinogen decarboxylase (URO-	0.000	20	5	1	98	325	33.40	33.43	33.27	33.20	33.08	33.30	33.29		1.01	0.2206	0.17
CLAU_1296	(S)-2-hydroxy-acid oxidase	0.000	34	10	10	244	338	33.05	33.21	33.09	33.35	33.46	33.43	33.28		2.15	0.0505	-0.30
CLAU_0385	Cysteine desulfurase	0.004	11	3	3	31	389	33.28	33.28	33.55	33.10	32.85	33.25	33.27		0.96	0.2362	0.30
CLAU_3582	Hypothetical protein	0.000	34	4	4	115	125	32.04	32.49	32.39	34.40	34.00	34.01	33.25	+	3.19	0.0216	-1.83
CLAU_1638	putative zinc-binding domain-containing	0.000	39	2	2	139	54	32.86	32.95	33.30	33.18	33.41	33.32	33.24		0.82	0.2962	-0.27
CLAU_1878	Adenylate kinase	0.000	54	9	9	150	217	33.25	33.46	33.55	33.22	33.11	33.06	33.24		1.35	0.1396	0.29
CLAU_0119	Acetolactate synthase biosynthetic type	0.000	34	13	13	251	536	33.42	33.61	33.65	33.04	32.72	33.00	33.23	+	2.19	0.0495	0.64
CLAU_0101	UBA/THIF-type NAD/FAD binding protei	0.000	50	7	7	202	228	33.25	33.17	33.19	33.39	33.37	33.07	33.22		0.28	0.6690	-0.07
CLAU_0084	UBA/THIF-type NAD/FAD binding protei	0.000	28	4	4	186	227	33.25	33.17	33.19	33.39	33.37	33.07	33.22		0.28	0.6690	-0.07
CLAU_2484	Methyl-accepting chemotaxis sensory tr	0.000	34	14	13	174	570	33.24	33.34	33.26	33.07	33.05	33.19	33.22		1.53	0.1108	0.18
CLAU_1239	Preprotein translocase subunit YajC	0.000	43	6	6	80	108	33.20	33.30	33.30	33.17	33.19	33.22	33.21		0.94	0.2437	0.07
CLAU_2445	Carbamoyl-phosphate synthase large su	0.000	36	34	34	352	1068	33.16	32.98	33.17	33.25	33.32	33.49	33.21		1.25	0.1591	-0.25
CLAU_1911	Ribosomal protein L11	0.000	46	6	6	124	141	33.22	33.33	33.46	32.97	33.19	33.02	33.21		1.35	0.1398	0.28
CLAU_1933	Glycine-tRNA ligase	0.000	29	14	14	345	463	33.22	33.17	33.44	33.04	33.22	33.19	33.21		0.56	0.4376	0.13
CLAU_1952	Foldase protein prsA	0.000	38	10	10	176	341	33.19	33.14	33.20	33.20	33.28	33.36	33.20		0.97	0.2324	-0.10
CLAU_1883	Ribosomal protein L18	0.000	41	6	6	80	119	33.21	33.36	33.37	32.93	33.15	33.09	33.18		1.43	0.1256	0.26
CLAU_1646	Diaminopimelate decarboxylase	0.000	33	12	12	190	431	33.43	33.41	33.28	32.86	33.02	33.07	33.18		2.11	0.0534	0.39
CLAU_3017	Homoserine dehydrogenase	0.000	39	13	13	202	432	33.00	33.35	33.24	33.11	33.13	33.22	33.18		0.15	0.8156	0.04
CLAU_1086	Uroporphyrin-III C-methyltransferase	0.000	42	15	15	200	486	33.25	33.20	33.38	32.79	33.09	33.14	33.17		1.04	0.2150	0.27
CLAU_1897	Ribosomal protein L25/L23	0.000	56	6	6	118	99	33.00	33.07	33.20	33.20	33.29	33.14	33.17		0.76	0.3302	-0.12
CLAU_1279	UTP-glucose-1-phosphate uridylyltransf	0.000	35	8	7	75	305	33.50	32.87	32.71	33.33	33.13	33.21	33.17		0.33	0.6236	-0.20
CLAU_3108	Elongation factor P	0.000	43	5	5	130	188	33.00	33.12	33.29	33.20	33.28	33.13	33.17		0.28	0.6615	-0.07
CLAU_2817	Histidine triad (HIT) protein	0.000	65	5	5	97	114	32.92	33.10	33.19	33.13	33.38	33.37	33.16		0.92	0.2563	-0.22
CLAU_0353	Nitrogenase component 2 (ATPase)	0.000	34	7	7	95	256	33.06	32.99	33.15	33.18	33.18	33.15	33.15		1.02	0.2187	-0.10
CLAU_0463	NADPH dehydrogenase	0.000	43	11	11	217	331	33.30	33.39	33.52	32.90	32.93	32.97	33.14	+	2.66	0.0321	0.47
CLAU_1570	Cobyrinic acid a,c-diamide synthase	0.000	63	11	11	260	257	32.76	33.17	33.10	33.09	33.17	33.16	33.13		0.43	0.5335	-0.13
CLAU_2676	Isocitrate dehydrogenase (NAD(+))	0.000	35	9	9	64	336	33.50	33.80	32.77	32.08	33.32	32.93	33.13		0.54	0.4560	0.58

CLAU_3107	Peptidase M24	0.000	35	9	9	141	358	32.93	33.09	32.98	33.59	33.49	33.14	33.12		1.32	0.1448	-0.41
CLAU_2707	Band 7 protein	0.000	45	11	11	248	312	32.98	33.10	32.96	33.12	33.26	33.21	33.11		1.42	0.1259	-0.18
CLAU_2720	4Fe-4S dicluster domain	0.000	39	6	6	201	184	33.13	33.27	32.87	33.06	33.22	33.08	33.11		0.08	0.8948	-0.03
CLAU_3210	Cysteine desulfurase	0.000	25	8	8	121	396	33.20	33.10	33.16	32.77	33.06	32.94	33.08		1.21	0.1645	0.23
CLAU_2077	DNA gyrase B subunit	0.000	28	15	15	67	637	32.93	32.87	32.84	33.29	33.23	33.40	33.08	+	2.79	0.0283	-0.43
CLAU_2750	50S ribosomal L21 protein	0.000	48	4	4	214	104	33.08	33.06	33.29	32.97	33.06	33.04	33.06		0.70	0.3581	0.12
CLAU_3327	Protein recA	0.000	39	11	11	154	362	32.76	32.82	32.88	33.27	33.33	33.20	33.04	+	3.03	0.0239	-0.45
CLAU_2044	Heat shock protein Hsp20	0.000	41	5	3	91	148	32.32	33.01	32.55	33.18	33.07	33.78	33.04		1.12	0.1874	-0.72
CLAU_2921	Isocitrate/isopropylmalate dehydrogena	0.000	27	8	8	142	354	33.79	33.37	33.60	32.51	32.41	32.70	33.04	+	2.67	0.0318	1.05
CLAU_3492	Methyl-accepting chemotaxis sensory tr	0.000	22	10	9	173	570	33.10	33.06	33.02	32.95	32.95	33.04	33.03		0.99	0.2271	0.08
CLAU_1551	Peptidase M16C associated domain prot	0.000	33	27	27	300	973	32.96	32.88	32.79	33.17	33.17	33.09	33.03		2.05	0.0565	-0.27
CLAU_1082	Precorrin-8X methylmutase	0.000	38	9	9	166	206	33.00	32.92	33.28	32.81	33.04	33.04	33.02		0.32	0.6322	0.10
CLAU_0148	Methionine synthase	0.000	47	7	2	66	208	33.01	32.87	32.87	33.03	33.26	33.19	33.02		1.38	0.1331	-0.24
CLAU_0145	Methionine synthase	0.000	47	7	2	66	208	33.01	32.87	32.87	33.03	33.26	33.19	33.02		1.38	0.1331	-0.24
CLAU_1889	Ribosomal protein L14	0.000	43	6	6	79	122	33.07	33.17	33.33	32.82	32.94	32.84	33.01		1.73	0.0828	0.32
CLAU_0134	Cobalamin B12-binding domain protein	0.000	36	6	1	59	207	33.00	32.85	32.85	33.01	33.25	33.16	33.01		1.31	0.1466	-0.24
CLAU_0150	putative ferredoxin	0.000	26	15	15	153	617	33.35	33.30	33.18	32.31	32.72	32.80	32.99		1.85	0.0717	0.67
CLAU_2741	Septum site-determining protein MinD	0.000	53	10	10	263	265	32.81	32.91	32.93	33.08	33.10	33.05	32.99		2.08	0.0546	-0.19
CLAU_0213	Transcriptional regulator RpiR family	0.000	40	10	10	163	291	32.84	33.02	32.90	32.98	33.12	32.99	32.99		0.72	0.3444	-0.11
CLAU_1588	SufBD protein	0.000	34	8	8	159	306	32.20	32.37	32.20	33.68	33.75	33.59	32.98	+	4.38	0.0046	-1.42
CLAU_0746	SufBD protein	0.000	34	8	8	159	306	32.20	32.37	32.20	33.68	33.75	33.59	32.98	+	4.38	0.0046	-1.42
CLAU_2934	Lipoate-protein ligase	0.004	20	6	6	18	329	33.06	33.07	33.28	32.90	32.75	32.82	32.98		1.70	0.0862	0.31
CLAU_3758	Glutamate synthase (ferredoxin)	0.000	29	34	34	259	1512	33.17	33.29	33.13	32.70	32.52	32.82	32.98	+	2.18	0.0495	0.52
CLAU_2771	Uroporphyrinogen decarboxylase (URO-	0.000	20	5	1	85	325	33.15	33.09	33.00	32.92	32.73	32.95	32.98		1.23	0.1609	0.21
CLAU_0149	Uroporphyrinogen decarboxylase (URO-	0.000	38	10	10	206	370	32.80	32.97	32.75	32.97	33.21	33.12	32.97		1.26	0.1552	-0.26
CLAU_1959	Bifunctional protein glmU	0.000	35	11	11	135	456	32.62	32.90	32.92	33.24	33.11	33.02	32.97		1.25	0.1572	-0.31
CLAU_2404	Nitrogen regulatory protein P-II	0.001	27	3	3	47	124	32.88	33.10	33.02	32.88	33.05	32.84	32.95		0.35	0.6030	0.08
CLAU_3506	Transcriptional regulator HxlR family	0.112	17	2	2	2	122	33.07	33.17	33.24	32.81	32.58	32.73	32.94	+	2.25	0.0461	0.45
CLAU_1041	NADH:flavin oxidoreductase	0.001	20	10	10	46	657	33.19	33.11	33.18	32.55	32.62	32.75	32.93	+	2.91	0.0271	0.52
CLAU_3166	Phosphoglycerate dehydrogenase	0.000	58	17	17	251	343	32.68	32.60	32.19	33.28	33.28	33.17	32.93		2.07	0.0549	-0.75
CLAU_3381	Methyl-accepting chemotaxis protein	0.000	37	19	19	174	661	33.17	33.29	32.65	32.21	33.45	32.63	32.91		0.26	0.6827	0.27
CLAU_2744	Methylglyoxal synthase	0.000	79	9	9	164	123	32.45	32.71	33.03	32.98	32.82	33.13	32.90		0.58	0.4279	-0.25
CLAU_3496	Thiolase-like enzyme	0.000	31	9	9	150	409	33.41	32.89	32.66	32.81	33.08	32.90	32.90		0.09	0.8936	0.06
CLAU_1325	Valyl-tRNA synthetase	0.000	35	25	25	232	882	32.99	32.99	32.98	32.65	32.75	32.79	32.89	+	2.45	0.0390	0.26
CLAU_2920	3-isopropylmalate dehydratase small su	0.000	54	6	6	103	164	32.98	32.97	33.17	32.78	32.10	32.33	32.88		1.41	0.1281	0.64
CLAU_1651	Prolyl-tRNA synthetase	0.000	39	13	13	170	476	32.87	32.85	32.78	32.85	33.12	32.98	32.86		0.84	0.2872	-0.15
CLAU_3721	Rubrerythrin	0.000	59	10	10	199	177	32.76	32.87	32.82	32.90	32.79	32.86	32.84		0.30	0.6451	-0.03
CLAU_2756	Endoribonuclease L-PSP	0.000	42	4	4	82	126	32.51	32.47	32.82	32.86	32.86	32.87	32.84		1.12	0.1888	-0.26
CLAU_2212	Mannosyl-glycoprotein	0.000	36	12	12	149	498	32.54	32.62	32.81	32.88	32.94	32.84	32.83		1.27	0.1546	-0.23
CLAU_2078	DNA gyrase subunit A	0.000	36	25	25	373	823	32.63	32.74	32.69	32.91	32.96	32.99	32.83	+	2.60	0.0339	-0.27
CLAU_0521	Methyl-accepting chemotaxis sensory tr	0.000	32	16	15	202	664	32.79	32.82	32.77	33.34	32.71	32.91	32.81		0.45	0.5254	-0.19
CLAU_1144	Histidine ammonia-lyase	0.013	8	3	3	10	523	32.80	32.74	32.96	32.80	32.88	32.81	32.81		0.02	0.9793	0.00
CLAU_2333	Hypothetical protein	0.000	25	9	9	58	478	31.07	30.63	32.45	33.17	33.38	33.15	32.80		1.54	0.1092	-1.85
CLAU_1537	NADH dehydrogenase (ubiquinone) subu	0.000	29	5	5	86	158	33.06	33.12	33.26	32.19	32.39	32.53	32.80	+	2.60	0.0339	0.78
CLAU_2728	UPF0735 ACT domain-containing protei	0.000	45	7	7	113	145	32.86	32.84	33.03	32.74	32.63	32.52	32.79		1.48	0.1179	0.28
CLAU_1577	Cobyrinic acid a,c-diamide synthase	0.000	40	10	10	138	260	32.69	32.95	32.95	32.75	32.69	32.83	32.79		0.48	0.4949	0.11

CLAU_3263	Ribulose-phosphate 3-epimerase	0.001	31	5	5	35	216	32.81	32.86	32.98	32.73	32.76	32.49	32.79		1.06	0.2098	0.22
CLAU_2317	Leucine-tRNA ligase	0.000	27	19	19	144	810	32.93	32.90	32.73	32.69	32.76	32.74	32.75		0.87	0.2736	0.12
CLAU_1758	Glutamine amidotransferase subunit pd	0.000	61	7	7	119	188	32.50	32.80	32.88	32.61	32.83	32.70	32.75		0.03	0.9562	0.01
CLAU_3809	Ethanolamine utilization protein EutS	0.000	56	5	5	87	125	32.77	32.78	32.86	32.57	32.61	32.72	32.75		1.48	0.1178	0.17
CLAU_0248	Arginyl-tRNA synthetase	0.000	31	18	18	190	564	32.99	33.00	32.76	32.66	32.66	32.73	32.75		1.33	0.1416	0.23
CLAU_2675	Aconitate hydratase	0.000	37	15	15	237	641	33.11	33.09	33.14	32.07	32.34	32.39	32.74	+	2.96	0.0257	0.85
CLAU_3250	hypothetical protein	0.000	79	6	6	124	91	32.68	32.68	33.02	32.75	32.78	32.72	32.74		0.14	0.8272	0.04
CLAU_1317	2,3,4,5-tetrahydropyridine-2,6-dicarbox	0.000	40	5	5	152	238	32.44	32.79	32.97	32.63	32.83	32.67	32.73		0.05	0.9375	0.02
CLAU_1229	Metallo-beta-lactamase	0.000	45	7	7	105	199	32.65	32.51	32.79	32.64	32.87	32.83	32.72		0.53	0.4581	-0.13
CLAU_0147	Dihydropteroate synthase	0.000	39	8	8	156	265	32.53	32.56	32.58	32.85	32.99	32.85	32.72	+	2.65	0.0326	-0.34
CLAU_3345	Methyl-accepting chemotaxis protein	0.000	32	16	15	243	666	32.78	32.80	32.64	32.65	32.56	32.77	32.71		0.43	0.5314	0.08
CLAU_2024	Agmatine deiminase	0.000	25	9	9	115	365	30.58	30.87	30.71	35.07	34.89	34.54	32.71	+	4.69	0.0046	-4.11
CLAU_2730	Homoserine kinase	0.000	44	9	9	128	299	32.81	33.03	32.93	32.37	32.57	32.40	32.69	+	2.23	0.0474	0.48
CLAU_2081	UPF0182 protein	0.000	27	20	20	236	905	32.59	32.59	32.55	32.92	32.82	32.79	32.69	+	2.52	0.0373	-0.27
CLAU_1435	Hypothetical protein	0.000	45	9	9	148	259	32.72	32.56	32.69	32.76	32.68	32.68	32.69		0.38	0.5806	-0.05
CLAU_1890	Ribosomal protein S17	0.000	32	3	3	161	84	32.94	33.00	32.64	32.34	32.62	32.72	32.68		0.88	0.2727	0.30
CLAU_0205	putative transcriptional regulator GntR f	0.000	51	14	14	125	394	32.98	32.93	32.78	32.04	32.18	32.57	32.68		1.69	0.0862	0.63
CLAU_1874	30S ribosomal protein S11	0.000	27	3	3	141	132	32.58	33.07	33.11	32.77	32.32	32.38	32.68		0.91	0.2607	0.43
CLAU_3131	NUDIX hydrolase	0.000	43	7	7	130	173	32.70	32.78	32.55	32.56	32.64	32.73	32.67		0.15	0.8154	0.03
CLAU_2010	Pyridine nucleotide-disulfide oxidoreduc	0.000	40	16	16	122	467	32.42	32.41	32.28	33.00	33.00	32.91	32.67	+	3.42	0.0182	-0.60
CLAU_1909	50S ribosomal protein L10	0.000	37	6	6	100	167	32.74	32.63	33.02	32.58	32.67	32.53	32.65		0.76	0.3285	0.20
CLAU_1997	3-oxoacyl-(acyl-carrier-protein) reductas	0.000	46	9	9	135	248	32.55	32.75	32.83	32.55	32.48	32.78	32.65		0.36	0.5931	0.11
CLAU_0016	Transcriptional regulator MarR family	0.000	46	7	7	176	180	30.90	31.80	31.58	33.70	33.51	33.49	32.65	+	2.81	0.0283	-2.14
CLAU_2582	Cell wall binding repeat 2-containing prc	0.000	34	13	13	203	463	32.79	32.51	32.85	32.34	32.33	32.78	32.65		0.57	0.4315	0.23
CLAU_3246	GTP-binding protein engA	0.000	35	13	13	117	438	32.53	32.41	33.07	33.88	32.66	32.62	32.64		0.34	0.6076	-0.38
CLAU_1281	Phosphoglucomutase	0.000	26	11	11	181	575	32.24	32.65	32.63	32.72	32.64	32.50	32.64		0.31	0.6362	-0.11
CLAU_1311	Dihydrodipicolinate reductase	0.000	44	8	8	198	250	32.74	32.77	32.92	32.45	32.48	32.50	32.62	+	2.36	0.0407	0.33
CLAU_0752	hypothetical protein	0.000	34	9	9	153	376	32.29	32.15	32.50	32.82	32.72	32.76	32.61		1.89	0.0683	-0.45
CLAU_3304	Ribosome-recycling factor	0.000	36	6	6	94	185	32.20	32.17	32.62	32.58	32.75	32.72	32.60		1.08	0.2036	-0.35
CLAU_1071	Cob(I)alamin adenosyltransferase	0.000	35	7	7	145	171	32.14	32.47	32.37	32.80	32.99	32.73	32.60		1.83	0.0729	-0.51
CLAU_1078	Cobalt-precorrin-2 C(20)-methyltransfer	0.000	17	5	5	89	228	32.85	32.64	32.99	32.47	32.46	32.55	32.60		1.46	0.1203	0.33
CLAU_2818	30S ribosomal protein S21	0.000	21	1	1	47	58	32.87	32.58	33.08	32.41	32.61	32.56	32.60		0.94	0.2436	0.32
CLAU_3311	Transcription elongation factor NusA	0.000	46	16	16	201	354	32.52	32.38	32.57	32.62	32.61	32.65	32.59		1.11	0.1932	-0.14
CLAU_1227	Histidyl-tRNA synthetase	0.000	35	12	12	138	416	32.32	32.48	32.36	32.68	32.74	32.69	32.58	+	2.45	0.0390	-0.32
CLAU_0193	YbaK/prolyl-tRNA synthetase associated	0.001	36	6	6	40	152	33.02	32.12	32.04	32.82	32.58	32.58	32.58		0.34	0.6105	-0.27
CLAU_2617	HlyD family secretion protein	0.000	50	9	9	180	264	32.39	32.54	32.35	32.78	32.71	32.59	32.57		1.54	0.1099	-0.27
CLAU_1848	Phosphoenolpyruvate-protein phosphot	0.000	29	11	11	139	537	32.68	32.75	32.43	32.75	32.07	32.42	32.56		0.40	0.5588	0.21
CLAU_2870	Phosphoribosylformylglycinamidine cycl	0.000	31	11	11	252	331	32.50	32.43	32.66	32.39	32.76	32.61	32.56		0.17	0.7915	-0.06
CLAU_2955	Protein-glutamate O-methyltransferase	0.000	36	9	9	95	255	32.49	32.54	32.63	32.63	32.55	32.49	32.55		0.02	0.9724	0.00
CLAU_3243	putative phosphate transport regulator	0.000	34	9	9	115	207	32.40	32.98	32.57	32.95	32.50	32.48	32.54		0.01	0.9873	0.01
CLAU_2046	ATP-dependent protease Lon family	0.000	37	19	19	127	631	32.21	32.41	32.44	32.81	32.75	32.63	32.54		1.87	0.0708	-0.38
CLAU_0532	NADP-dependent isopropanol dehydrog	0.000	20	5	5	93	351	31.88	31.96	31.87	33.48	33.61	33.11	32.54	+	3.22	0.0206	-1.50
CLAU_0380	Thymidylate synthase family	0.000	24	6	6	78	271	32.87	32.29	32.77	30.80	31.24	33.15	32.53		0.54	0.4512	0.91
CLAU_2986	hypothetical protein	0.000	35	20	20	111	726	32.55	32.48	32.28	32.44	32.67	32.66	32.52		0.63	0.3968	-0.15
CLAU_3577	Hypothetical protein	0.001	17	2	2	46	122	31.80	31.94	32.00	33.03	33.06	33.11	32.52	+	4.26	0.0057	-1.15
CLAU_0891	GCN5-related N-acetyltransferase	0.054	5	1	1	3	139	33.48	33.34	32.93	31.93	30.80	32.08	32.51		1.71	0.0854	1.65

CLAU_3392	Oligopeptide transporter OPT family	0.000	13	7	7	89	640	32.25	32.25	32.17	32.92	33.01	32.74	32.50	+	2.87	0.0283	-0.67
CLAU_2723	Sigma-54 specific transcriptional regulat	0.004	12	5	4	23	471	32.50	32.63	32.56	32.30	32.41	32.48	32.49		1.21	0.1645	0.17
CLAU_2914	hypothetical protein	0.000	44	11	11	162	289	32.54	32.22	32.29	32.42	32.62	32.58	32.48		0.76	0.3281	-0.19
CLAU_1528	Methyl-accepting chemotaxis sensory tr	0.000	31	13	13	124	383	32.31	32.44	32.56	32.60	32.49	32.46	32.48		0.40	0.5533	-0.08
CLAU_0064	Serine dehydratase	0.000	41	12	12	191	421	32.95	33.16	33.19	30.71	30.44	31.98	32.47		1.89	0.0684	2.06
CLAU_1489	Membrane associated chemotaxis sensc	0.000	14	6	5	46	570	32.56	32.56	32.44	32.39	32.37	32.48	32.46		0.95	0.2404	0.11
CLAU_3228	Cell division protein FtsA	0.000	45	15	15	187	451	32.30	32.35	32.33	32.69	32.56	32.62	32.46	+	2.74	0.0285	-0.30
CLAU_0297	Methyl-accepting chemotaxis sensory tr	0.000	6	3	2	55	551	32.53	32.53	32.43	32.36	32.35	32.46	32.45		1.03	0.2170	0.11
CLAU_2691	XshC-Cox1-family protein	0.000	35	11	11	153	369	32.46	32.30	32.31	32.43	32.51	32.52	32.45		1.03	0.2170	-0.13
CLAU_0812	putative signal transduction protein witl	0.004	3	2	2	35	442	32.61	32.51	32.83	32.37	32.31	32.33	32.44		1.51	0.1133	0.31
CLAU_2626	Methyl-accepting chemotaxis sensory tr	0.001	4	2	1	38	567	32.53	32.52	32.42	32.36	32.34	32.45	32.44		1.03	0.2185	0.11
CLAU_3926	Methyl-accepting chemotaxis sensory tr	0.001	5	3	2	34	659	32.52	32.52	32.42	32.36	32.34	32.45	32.44		1.02	0.2187	0.10
CLAU_3172	Methyl-accepting chemotaxis sensory tr	0.003	6	3	2	32	720	32.52	32.52	32.42	32.36	32.34	32.45	32.44		1.02	0.2187	0.10
CLAU_2338	Membrane associated chemotaxis sensc	0.003	2	1	0	28	574	32.52	32.52	32.42	32.36	32.34	32.45	32.44		1.02	0.2187	0.10
CLAU_0339	Methyl-accepting chemotaxis sensory tr	0.003	6	3	2	30	571	32.52	32.52	32.42	32.36	32.34	32.45	32.44		1.02	0.2187	0.10
CLAU_2331	'putative molybdopterin binding domair	0.000	27	5	5	78	257	32.37	32.07	31.00	33.03	32.56	32.46	32.42		0.90	0.2640	-0.87
CLAU_1084	Cobyrinic acid a,c-diamide synthase	0.000	33	14	14	192	444	32.30	32.46	32.47	32.17	32.47	32.35	32.41		0.32	0.6321	0.08
CLAU_1107	Nitroreductase	0.000	57	8	8	162	190	31.95	31.45	32.57	32.24	32.87	32.76	32.41		0.77	0.3220	-0.63
CLAU_1027	3-Phosphoglycerate dehydrogenase	0.000	35	8	8	73	388	32.15	32.71	32.63	32.32	32.38	32.41	32.40		0.29	0.6557	0.13
CLAU_2971	Flagellar hook-associated 2 protein	0.000	29	17	17	459	785	32.36	32.42	32.46	32.49	32.31	32.28	32.39		0.30	0.6442	0.05
CLAU_3909	Periplasmic binding protein domain-con	0.000	29	8	8	150	368	32.33	32.18	31.82	32.76	32.56	32.45	32.39		1.28	0.1533	-0.48
CLAU_3616	Tryptophan synthase beta chain	0.000	19	6	6	117	391	29.84	30.07	30.94	34.16	33.84	34.05	32.39	+	3.37	0.0187	-3.73
CLAU_2892	ABC-type transporter permease	0.142	4	1	1	2	250	32.26	32.09	27.53	33.07	32.52	33.14	32.39		0.66	0.3746	-2.28
CLAU_1982	Hypothetical protein	0.000	19	3	3	49	166	32.39	32.47	32.59	32.25	32.33	32.35	32.37		1.24	0.1593	0.17
CLAU_1563	RNA-binding protein	0.000	47	3	3	50	64	32.02	32.21	32.12	32.53	32.56	32.68	32.37	+	2.57	0.0344	-0.47
CLAU_1837	Glucosamine-fructose-6-phosphate	0.000	26	14	14	172	608	32.47	32.56	32.42	32.16	32.26	32.31	32.37		1.79	0.0778	0.24
CLAU_0722	4Fe-4S ferredoxin iron-sulfur binding	0.000	30	6	6	69	234	32.71	32.59	32.33	32.19	32.11	32.39	32.36		1.05	0.2109	0.31
CLAU_0401	S-adenosylmethionine synthetase	0.000	14	3	1	59	243	32.65	32.51	32.40	31.97	32.25	32.31	32.36		1.27	0.1547	0.34
CLAU_1038	Hypothetical protein	0.000	44	8	8	112	189	32.13	32.12	32.45	32.31	32.55	32.39	32.35		0.64	0.3867	-0.18
CLAU_2358	Transketolase	0.000	28	6	5	89	274	32.34	32.27	32.35	32.39	32.42	32.34	32.35		0.86	0.2816	-0.06
CLAU_0394	S-ribosylhomocysteinase luxS	0.000	65	9	9	74	159	32.08	32.29	32.40	32.41	32.42	32.29	32.35		0.50	0.4869	-0.12
CLAU_2636	hypothetical protein	0.000	30	10	10	115	318	32.19	32.16	32.29	32.43	32.37	32.50	32.33		1.81	0.0758	-0.22
CLAU_2511	Cell wall binding repeat 2-containing prc	0.000	23	16	16	117	1110	32.08	32.09	32.35	32.51	32.53	32.30	32.33		1.12	0.1891	-0.27
CLAU_1924	DNA integrity scanning protein disA	0.000	23	7	7	49	354	31.98	32.65	32.64	32.20	32.27	32.37	32.32		0.25	0.6973	0.14
CLAU_0262	Hemerythrin-like metal-binding protein	0.000	33	5	5	124	143	32.40	32.32	32.32	32.26	32.36	32.31	32.32		0.39	0.5624	0.04
CLAU_0025	Ferritin	0.000	61	9	9	110	173	32.81	32.75	32.51	31.85	32.08	31.93	32.30	+	2.53	0.0365	0.74
CLAU_1108	D-lactate dehydrogenase	0.000	32	10	10	116	326	32.37	32.27	32.24	32.01	32.30	32.47	32.29		0.08	0.8944	0.03
CLAU_2414	Malate dehydrogenase	0.000	44	14	14	180	409	32.27	32.26	32.43	32.18	32.38	32.28	32.28		0.19	0.7641	0.04
CLAU_3019	hypothetical protein	0.000	33	6	6	215	187	32.31	32.14	32.38	32.24	32.18	32.67	32.28		0.20	0.7586	-0.09
CLAU_0100	Thiamine S protein	0.000	78	5	1	84	74	31.30	31.99	32.51	32.86	32.79	32.03	32.27		0.64	0.3845	-0.63
CLAU_1610	Fn3 associated repeat-containing protei	0.002	17	15	15	34	1033	32.31	32.17	32.25	32.28	32.43	32.17	32.27		0.23	0.7218	-0.05
CLAU_2472	Aspartate transaminase	0.000	41	12	12	106	394	32.49	32.67	32.33	32.19	32.07	32.09	32.26		1.65	0.0933	0.38
CLAU_3336	Adenylosuccinate lyase	0.000	43	17	17	169	476	31.99	32.33	32.08	32.26	32.55	32.26	32.26		0.73	0.3419	-0.22
CLAU_1839	Phosphoglucosamine mutase	0.000	44	16	16	202	449	32.98	32.68	32.14	32.01	32.28	32.23	32.26		0.76	0.3298	0.43
CLAU_2147	Nucleoid-associated protein	0.000	75	5	5	216	114	31.94	32.41	32.27	32.15	32.25	32.26	32.26		0.03	0.9589	-0.01
CLAU_2964	Flagellar hook-associated protein FlgK	0.000	36	14	14	164	638	31.49	31.82	32.02	32.63	32.58	32.48	32.25		2.09	0.0538	-0.79

CLAU_1087	Porphobilinogen deaminase	0.000	44	13	13	163	290	32.26	32.41	32.86	31.86	32.23	32.17	32.25		0.93	0.2522	0.42
CLAU_1587	Nitrite and sulfite reductase 4Fe-4S regi	0.000	33	9	9	84	286	32.33	32.35	32.25	32.13	32.23	32.14	32.24		1.50	0.1140	0.14
CLAU_0876	3-phosphoshikimate 1-carboxyvinyltran	0.000	31	13	13	315	426	32.35	32.36	32.39	31.86	31.93	32.11	32.23	+	2.22	0.0480	0.40
CLAU_2522	Hypothetical protein	0.000	18	5	5	121	290	32.46	32.10	32.09	32.22	32.32	32.24	32.23		0.13	0.8451	-0.04
CLAU_1260	Transcriptional regulator BadM/Rrf2 fan	0.000	41	5	5	60	137	31.85	32.38	32.07	31.94	32.38	32.37	32.22		0.24	0.7074	-0.13
CLAU_2267	CTP synthase	0.000	30	13	13	108	534	32.50	32.07	31.89	32.35	32.56	32.08	32.22		0.32	0.6322	-0.18
CLAU_3211	FeS cluster assembly scaffold protein Ni	0.000	73	7	7	115	143	32.53	32.46	32.23	31.96	32.10	31.95	32.17		1.77	0.0799	0.40
CLAU_2712	Molybdopterin oxidoreductase	0.000	41	4	4	62	138	32.44	32.02	31.50	32.14	32.91	32.17	32.16		0.49	0.4876	-0.42
CLAU_2377	6-phosphofructokinase	0.000	40	10	10	106	318	31.61	31.65	31.82	32.54	32.49	32.57	32.16	+	3.60	0.0147	-0.84
CLAU_1962	UDP-N-acetylmuramate-L-alanine ligase	0.000	31	13	13	132	457	31.97	31.94	32.02	32.34	32.46	32.28	32.15	+	2.57	0.0344	-0.38
CLAU_1931	Translation elongation factor G	0.000	36	18	18	199	694	32.97	33.16	32.43	30.58	29.84	31.87	32.15		1.53	0.1112	2.09
CLAU_0707	Hypothetical protein	0.000	32	10	10	171	332	31.98	31.82	32.01	32.32	32.31	32.29	32.15	+	2.46	0.0389	-0.37
CLAU_1436	Orotate phosphoribosyltransferase	0.000	35	6	6	105	190	32.13	32.11	32.37	32.15	32.28	32.00	32.14		0.20	0.7580	0.06
CLAU_3082	Bifunctional protein pyrR	0.000	57	9	9	97	177	31.54	31.89	32.14	32.58	32.31	32.13	32.14		1.04	0.2149	-0.48
CLAU_2725	Methyl-accepting chemotaxis sensory tr	0.000	34	10	9	179	346	32.43	32.36	32.33	31.86	31.84	31.94	32.14	+	3.50	0.0179	0.49
CLAU_1774	Ethanolamine utilization EutQ family prc	0.000	37	6	6	106	229	32.34	32.29	32.22	32.01	31.97	32.05	32.14	+	2.55	0.0353	0.27
CLAU_0354	Hydrogenase expression/formation prot	0.000	30	6	6	75	334	31.70	32.00	32.04	32.23	32.39	32.27	32.14		1.51	0.1133	-0.38
CLAU_2837	RNA polymerase sigma factor	0.000	29	8	8	157	361	31.92	32.13	32.16	32.12	32.12	32.24	32.13		0.45	0.5191	-0.09
CLAU_1560	Formate/nitrate transporter	0.000	19	4	4	79	280	32.05	32.19	32.20	32.27	31.91	31.99	32.12		0.31	0.6407	0.09
CLAU_0522	NADPH-dependent FMN reductase	0.000	51	6	6	155	181	31.80	32.19	32.34	32.04	32.04	32.25	32.12		0.00	1.0000	0.00
CLAU_3617	Tryptophan synthase subunit alpha	0.000	21	4	4	62	261	28.96	29.93	30.24	34.10	34.11	33.98	32.11	+	3.45	0.0182	-4.35
CLAU_1080	Precorrin-6y C5,15-methyltransferase	0.000	34	7	7	80	205	32.13	32.08	32.29	31.81	32.14	32.03	32.11		0.68	0.3662	0.17
CLAU_0724	Methyl-accepting chemotaxis sensory tr	0.000	48	9	9	87	275	31.91	31.93	31.52	32.38	32.26	32.50	32.10		1.77	0.0794	-0.59
CLAU_1200	Molybdenum cofactor biosynthesis prot	0.000	36	12	12	125	322	31.96	31.85	32.10	32.18	32.23	32.07	32.09		1.03	0.2170	-0.19
CLAU_1726	Phospholipid/glycerol acyltransferase	0.000	47	9	9	106	233	31.92	32.03	31.92	32.14	32.35	32.30	32.09		1.86	0.0710	-0.31
CLAU_3300	GTP-sensing pleiotropic transcriptional	0.000	18	5	5	74	258	32.08	32.22	32.16	31.90	32.08	32.05	32.08		0.98	0.2319	0.14
CLAU_0146	Uroporphyrinogen decarboxylase (URO-	0.000	32	10	10	107	370	31.97	31.72	31.70	32.19	32.40	32.30	32.08		2.04	0.0574	-0.50
CLAU_3798	Aldo-ketol reductase (NADPH-depender	0.000	31	9	9	92	280	31.81	31.88	31.66	32.25	32.50	32.36	32.07	+	2.42	0.0391	-0.59
CLAU_1999	Enoyl-(acyl-carrier-protein) reductase II	0.000	23	5	5	111	312	32.03	32.51	31.98	31.81	32.09	32.20	32.06		0.27	0.6718	0.14
CLAU_0083	Thiamine S protein	0.000	78	5	1	66	74	30.40	31.63	32.29	32.77	32.65	31.83	32.06		0.71	0.3521	-0.98
CLAU_0803	HesB-related (seleno) protein	0.001	21	3	3	39	113	32.06	31.81	32.14	31.97	32.05	32.19	32.06		0.22	0.7305	-0.07
CLAU_1550	Carbon starvation protein CstA	0.000	12	5	5	63	596	31.78	31.95	31.54	32.15	32.35	32.17	32.05		1.59	0.1019	-0.47
CLAU_0024	Rubredoxin domain-containing protein	0.000	43	7	7	71	229	32.31	32.27	32.29	31.66	31.60	31.81	32.04	+	3.15	0.0223	0.60
CLAU_3261	Serine/threonine protein kinase with PA	0.000	40	18	18	191	635	31.96	32.03	31.60	32.05	32.23	32.15	32.04		0.91	0.2569	-0.28
CLAU_1024	Methyl-accepting chemotaxis protein	0.000	20	9	8	59	571	32.20	32.04	32.09	31.90	31.94	32.03	32.04		1.18	0.1720	0.15
CLAU_2813	Molecular chaperone DnaJ	0.000	25	9	9	75	382	32.19	32.44	32.01	32.05	31.21	31.65	32.03		0.99	0.2271	0.58
CLAU_3904	Xylulokinase	0.000	33	13	13	146	520	31.94	31.92	31.52	32.30	32.37	32.12	32.03		1.41	0.1285	-0.47
CLAU_2184	Silent information regulator protein Sir2	0.000	36	5	5	89	241	31.12	31.38	31.78	32.47	32.29	32.28	32.03		1.99	0.0611	-0.92
CLAU_1631	Metal dependent phosphohydrolase	0.000	33	11	11	109	436	31.84	31.97	31.77	32.13	32.14	32.06	32.02		1.76	0.0800	-0.25
CLAU_2245	Extracellular solute-binding protein fami	0.013	8	3	3	9	421	32.30	32.06	31.98	31.92	31.84	32.03	32.01		0.76	0.3283	0.18
CLAU_1077	Precorrin-4 C(11)-methyltransferase	0.000	53	9	9	89	258	32.01	32.19	31.82	31.76	32.10	31.98	32.00		0.15	0.8119	0.06
CLAU_0081	Molybdopterin oxidoreductase	0.000	33	14	14	134	562	32.11	31.97	32.00	31.95	32.17	31.96	31.99		0.00	1.0000	0.00
CLAU_3174	ATP phosphoribosyltransferase regulato	0.000	34	11	11	70	392	31.97	32.07	31.70	31.27	32.02	31.97	31.97		0.24	0.7144	0.16
CLAU_2686	Transcriptional regulator AsnC family	0.000	38	6	6	57	159	32.13	31.98	31.78	31.90	31.96	32.00	31.97		0.03	0.9589	0.01
CLAU_2290	ATP synthase gamma chain	0.000	35	6	6	84	283	31.91	31.69	31.91	32.09	32.04	32.02	31.97		1.31	0.1460	-0.21
CLAU_1766	Alcohol dehydrogenase	0.000	39	12	12	139	382	32.43	32.34	31.92	31.72	31.81	32.01	31.97		1.00	0.2227	0.38

CLAU_0459	Aconitase/isopropylmalate dehydratase	0.000	28	16	16	131	764	31.45	31.65	31.96	31.97	32.17	32.07	31.97		1.13	0.1857	-0.38
CLAU_1085	Delta-aminolevulinic acid dehydratase	0.000	35	8	8	106	324	32.21	32.25	32.05	31.43	31.87	31.88	31.97		1.30	0.1489	0.44
CLAU_3284	Cell division transporter substrate-bindi	0.000	50	11	11	93	303	31.87	31.96	31.95	32.07	31.88	31.98	31.96		0.33	0.6161	-0.05
CLAU_3084	Diaminopimelate epimerase	0.000	43	9	9	120	298	31.89	31.99	31.91	31.92	32.25	32.21	31.96		0.84	0.2874	-0.20
CLAU_0878	Prephenate dehydrogenase	0.000	27	6	6	56	288	32.15	32.09	32.09	31.58	31.73	31.81	31.95	+	2.34	0.0411	0.40
CLAU_0447	XshC-Cox1-family protein	0.000	38	12	12	99	352	31.77	31.89	31.80	32.01	32.15	32.16	31.95		2.05	0.0567	-0.29
CLAU_1998	ACP S-malonyltransferase	0.000	44	9	9	86	312	31.90	32.19	32.14	31.98	31.86	31.91	31.95		0.77	0.3251	0.16
CLAU_1310	Aspartate transaminase	0.000	39	11	11	61	383	31.84	32.12	30.54	30.68	32.04	32.22	31.94		0.07	0.9047	-0.15
CLAU_2869	Amidophosphoribosyltransferase	0.000	20	7	7	91	481	32.02	31.94	31.85	32.01	31.93	31.83	31.94		0.06	0.9174	0.01
CLAU_1180	Cysteine desulfurase family protein	0.000	43	15	15	133	383	31.75	31.87	31.62	32.15	32.07	31.99	31.93		1.71	0.0854	-0.32
CLAU_1301	Phenylalanyl-tRNA synthetase subunit b	0.000	32	19	19	150	793	32.04	32.00	32.24	31.83	31.75	31.83	31.92		1.67	0.0895	0.29
CLAU_0325	Elongator protein 3/MiaB/NifB	0.000	31	10	10	73	324	31.26	31.68	30.65	32.15	32.68	32.68	31.92		1.70	0.0854	-1.31
CLAU_1580	UspA domain-containing protein	0.001	45	6	6	35	136	32.55	32.44	32.31	31.48	30.96	31.52	31.92	+	2.35	0.0407	1.11
CLAU_1920	Cysteinyl-tRNA synthetase	0.000	32	14	14	113	465	31.64	31.85	31.76	32.23	32.33	31.96	31.91		1.55	0.1082	-0.42
CLAU_2951	hypothetical protein	0.000	27	14	14	118	650	31.85	31.72	31.38	31.99	32.15	31.95	31.90		1.17	0.1752	-0.38
CLAU_2950	Aminotransferase class IV	0.000	36	10	10	95	275	31.75	31.70	31.66	32.15	32.23	32.05	31.90	+	2.78	0.0283	-0.44
CLAU_3383	Pyridoxamine 5'-phosphate oxidase	0.000	41	3	3	111	131	31.49	31.98	31.95	31.84	31.96	31.62	31.90		0.00	1.0000	0.00
CLAU_3338	Glutamate-tRNA ligase	0.000	40	15	15	135	480	31.87	31.86	31.95	31.84	32.03	31.90	31.89		0.18	0.7756	-0.03
CLAU_2957	Chemotaxis protein CheC	0.000	27	4	4	57	199	31.66	31.85	32.07	31.88	31.88	31.87	31.88		0.05	0.9375	-0.02
CLAU_3247	Glycerol-3-phosphate dehydrogenase [N	0.000	38	11	11	89	332	31.59	31.39	31.66	32.08	32.16	32.08	31.87	+	2.56	0.0349	-0.56
CLAU_2520	ResB family protein	0.000	29	4	4	143	135	31.14	31.28	31.49	32.32	32.42	32.23	31.86	+	3.04	0.0237	-1.02
CLAU_1330	Survival protein SurE-like	0.013	10	2	2	9	249	31.71	32.74	25.99	26.14	32.00	32.83	31.86		0.02	0.9720	-0.18
CLAU_2172	PpiC-type peptidyl-prolyl cis-trans isome	0.000	41	9	9	114	247	31.90	31.84	31.98	31.86	31.74	31.74	31.85		1.04	0.2144	0.13
CLAU_3328	Ribonuclease Y	0.000	26	10	10	101	514	31.82	31.96	31.86	32.10	31.80	31.80	31.84		0.06	0.9177	-0.02
CLAU_1767	Histidine kinase	0.000	32	10	10	151	396	32.03	32.59	32.29	31.64	31.56	31.54	31.84		1.93	0.0651	0.72
CLAU_1643	Peptide chain release factor 3	0.000	25	12	12	168	530	32.11	31.64	31.74	31.70	31.93	32.24	31.84		0.23	0.7154	-0.13
CLAU_1182	Lipoyl synthase	0.000	47	12	12	150	283	31.53	31.71	31.78	32.08	32.09	31.89	31.84		1.61	0.1005	-0.35
CLAU_1525	Pyridoxamine 5-phosphate oxidase-rela	0.000	48	6	6	111	130	31.17	31.21	31.88	31.79	31.90	31.97	31.84		0.92	0.2530	-0.47
CLAU_0249	UDP-glucose 4-epimerase	0.000	33	8	8	76	328	31.60	31.88	31.87	31.71	31.81	31.85	31.83		0.02	0.9696	-0.01
CLAU_0551	Molybdenum cofactor biosynthesis C	0.000	47	9	9	110	158	31.83	31.80	31.77	31.72	31.91	31.86	31.82		0.19	0.7621	-0.03
CLAU_1943	RNA binding S1 domain protein	0.000	19	2	2	85	136	31.56	31.45	31.89	31.82	31.85	31.81	31.82		0.66	0.3757	-0.19
CLAU_1546	hypothetical protein	0.000	69	6	6	101	95	31.72	31.72	31.65	31.93	31.89	32.07	31.81		1.96	0.0631	-0.27
CLAU_0887	putative metal dependent phosphohydr	0.000	51	14	14	138	352	32.03	31.70	31.98	31.71	31.64	31.84	31.78		0.66	0.3739	0.17
CLAU_1441	Aspartate carbamoyltransferase	0.000	37	11	11	99	306	31.78	31.76	31.64	31.93	31.89	31.75	31.77		0.86	0.2785	-0.13
CLAU_3580	Glycine cleavage system H protein	0.000	43	11	11	86	283	30.85	30.76	30.63	32.69	33.16	32.91	31.77	+	3.88	0.0107	-2.17
CLAU_3078	5'-methylthioadenosine nucleosidase /	0.000	17	3	3	60	230	31.79	31.75	31.68	31.74	31.78	31.86	31.77		0.49	0.4943	-0.05
CLAU_2698	Ribonucleotide reductase	0.000	19	16	16	98	1013	31.47	31.77	31.72	31.76	31.91	31.88	31.77		0.89	0.2706	-0.20
CLAU_1881	Ribosomal protein L30	0.003	20	1	1	28	59	31.85	31.85	32.27	31.55	31.59	31.67	31.76		1.26	0.1565	0.39
CLAU_1267	UDP-N-acetylglucosamine 2-epimerase	0.000	45	11	11	116	350	31.67	31.75	31.67	31.85	31.84	31.77	31.76		1.55	0.1079	-0.12
CLAU_3495	Beta-ketoacyl-[acyl-carrier-protein] synt	0.000	27	7	7	84	361	31.37	31.55	31.75	31.75	31.85	31.81	31.75		1.02	0.2188	-0.25
CLAU_2203	Metal dependent phosphohydrolase	0.000	31	14	14	142	466	31.68	31.48	31.63	31.82	31.80	31.90	31.74		1.65	0.0935	-0.24
CLAU_3287	30S ribosomal protein S16	0.001	41	3	3	45	85	31.37	31.76	31.98	31.39	31.89	31.72	31.74		0.05	0.9297	0.04
CLAU_0330	Succinate dehydogenase/fumarate redu	0.004	48	3	3	26	108	32.48	32.36	32.15	31.19	30.67	31.33	31.74	+	2.33	0.0419	1.27
CLAU_1196	Sporulation protein ytfJ	0.000	40	5	5	75	120	31.51	31.65	31.63	31.85	31.82	31.86	31.74	+	2.26	0.0459	-0.25
CLAU_2157	Alanine-glyoxylate transaminase	0.000	23	8	8	68	356	31.74	31.70	31.90	31.72	31.83	31.59	31.73		0.29	0.6530	0.07
CLAU_3214	Alanine-tRNA ligase	0.000	33	22	22	297	879	31.84	31.81	31.56	31.59	31.78	31.67	31.73		0.21	0.7416	0.06

CLAU_1232	Adenine phosphoribosyltransferase	0.000	30	4	4	85	172	31.52	31.58	31.70	31.73	31.85	31.82	31.72		1.45	0.1218	-0.20
CLAU_0300	Response regulator receiver protein	0.068	8	1	1	4	120	31.75	31.44	31.55	31.92	31.68	32.07	31.72		1.00	0.2241	-0.31
CLAU_1251	GTP-binding protein hflX	0.167	2	1	1	1	595	31.78	31.76	32.04	31.66	30.85	30.89	31.71		1.23	0.1614	0.73
CLAU_0120	Acetolactate synthase small subunit	0.000	34	4	4	75	158	31.75	33.02	32.66	31.60	31.44	31.65	31.70		1.12	0.1882	0.91
CLAU_0095	Molybdenum co-factor synthesis domai	0.000	39	10	10	129	410	30.76	31.60	31.46	32.23	32.29	31.79	31.70		1.28	0.1518	-0.83
CLAU_2043	Heat shock protein Hsp20	0.001	29	4	2	34	148	30.88	31.67	31.08	31.85	31.72	32.46	31.70		1.14	0.1820	-0.80
CLAU_2922	Nitrate reductase	0.000	25	9	9	81	420	32.07	32.01	31.83	31.55	31.32	31.44	31.69	+	2.26	0.0459	0.53
CLAU_2244	Radical SAM domain protein	0.000	22	11	11	106	460	31.16	30.73	31.31	32.60	32.21	32.03	31.67		2.13	0.0524	-1.21
CLAU_2819	Yqey-like protein	0.000	36	6	6	47	147	34.47	35.70	30.42	30.10	30.15	32.91	31.67		0.60	0.4124	2.48
CLAU_3187	hypothetical protein	0.000	45	8	8	74	197	31.53	31.54	31.68	31.72	31.74	31.65	31.67		1.01	0.2206	-0.12
CLAU_2273	Peptide chain release factor 1	0.000	39	10	10	91	357	31.81	31.53	31.38	31.59	31.74	31.77	31.67		0.39	0.5687	-0.13
CLAU_3118	hypothetical protein	0.000	58	4	4	126	132	31.33	31.59	31.62	31.69	31.82	31.75	31.66		1.14	0.1852	-0.24
CLAU_0080	4Fe-4S ferredoxin iron-sulpur binding	0.001	17	3	3	39	190	31.76	31.68	31.64	31.62	31.65	31.65	31.65		0.66	0.3757	0.05
CLAU_3549	Nitroreductase	0.000	33	5	5	49	173	31.29	31.76	31.78	31.69	31.55	31.59	31.64		0.00	1.0000	0.00
CLAU_0659	ABC-type spermidine/putrescine transp	0.000	29	8	8	109	352	31.44	31.27	31.37	31.82	31.99	31.93	31.63	+	2.86	0.0283	-0.55
CLAU_0819	RNA binding S1 domain protein	0.000	23	8	8	54	392	31.63	31.63	31.24	31.62	31.67	31.72	31.63		0.57	0.4349	-0.17
CLAU_1092	Cobalt ABC transporter ATPase	0.000	36	8	8	85	241	31.62	31.22	31.63	31.43	31.78	31.66	31.63		0.32	0.6269	-0.13
CLAU_0018	Phenazine biosynthesis protein PhzF fan	0.000	42	8	8	97	260	30.42	30.87	30.65	33.50	33.00	32.38	31.63	+	2.57	0.0344	-2.31
CLAU_0811	Cyclopropane-fatty-acyl-phospholipid sy	0.000	25	8	8	98	394	31.46	31.69	31.78	31.32	31.63	31.61	31.62		0.37	0.5816	0.12
CLAU_2705	NAD(+) synthetase	0.000	18	11	11	108	637	31.72	31.75	31.61	31.59	31.59	31.63	31.62		0.94	0.2436	0.09
CLAU_3256	Methionyl-tRNA formyltransferase	0.000	26	7	7	60	310	32.64	31.70	30.14	31.09	31.64	31.59	31.62		0.02	0.9683	0.05
CLAU_1711	2,3-bisphosphoglycerate-independent	0.000	37	14	14	113	511	31.57	31.49	31.47	31.65	31.78	31.68	31.61		1.75	0.0810	-0.19
CLAU_2984	Glycosyl transferase family 2	0.000	25	19	19	116	697	31.88	31.79	31.73	31.45	31.46	31.40	31.60	+	2.81	0.0283	0.36
CLAU_0199	RNA chaperone hfq	0.004	58	3	3	27	80	31.33	31.61	31.58	31.47	31.61	31.74	31.60		0.35	0.6025	-0.10
CLAU_3611	Anthranilate synthase component I	0.000	34	16	16	97	476	28.63	28.69	29.65	33.56	33.53	33.55	31.59	+	3.79	0.0113	-4.56
CLAU_3267	DAK2 domain fusion protein YloV	0.000	29	14	14	96	554	31.60	31.71	31.55	31.48	31.49	31.69	31.58		0.33	0.6195	0.07
CLAU_3613	Anthranilate phosphoribosyltransferase	0.000	26	8	8	75	337	28.36	28.18	29.93	33.54	33.22	33.47	31.58	+	2.90	0.0271	-4.59
CLAU_3964	Cytosine deaminase	0.000	31	10	10	63	422	31.61	31.53	32.33	31.84	31.13	31.11	31.57		0.59	0.4185	0.46
CLAU_1851	Citrate lyase alpha subunit	0.000	28	13	13	208	513	31.61	31.60	31.59	31.42	31.46	31.55	31.57		1.47	0.1196	0.12
CLAU_2073	DNA polymerase III beta subunit	0.000	34	12	12	60	367	31.53	31.55	31.52	31.61	31.52	31.48	31.53		0.03	0.9625	0.00
CLAU_0355	Hydrogenase expression/formation prot	0.000	22	6	6	59	352	31.38	31.30	31.45	31.60	31.77	31.80	31.53		1.99	0.0611	-0.35
CLAU_1923	Hypothetical protein	0.000	40	4	4	64	134	31.43	31.55	31.49	31.63	31.49	31.74	31.52		0.74	0.3352	-0.13
CLAU_0734	Glutamine-scyllo-inositol transaminase	0.000	37	10	10	104	373	31.48	31.38	31.51	31.51	31.68	31.71	31.51		1.13	0.1867	-0.18
CLAU_0867	Phosphoglucomutase/phosphomannom	0.000	37	15	15	116	504	31.48	31.46	31.28	31.54	31.81	31.66	31.51		1.23	0.1610	-0.26
CLAU_3183	Phosphoribosyl-ATP pyrophosphatase	0.000	36	4	4	94	111	31.80	31.46	31.80	31.43	31.22	31.55	31.51		0.90	0.2640	0.29
CLAU_0390	Amino acid/polyamine transporter I	0.001	3	1	1	37	470	32.47	32.20	32.44	30.40	30.30	30.81	31.51	+	3.33	0.0187	1.87
CLAU_0389	Amino acid/polyamine transporter I	0.001	3	1	1	37	472	32.47	32.20	32.44	30.40	30.30	30.81	31.51	+	3.33	0.0187	1.87
CLAU_2682	ATP-binding domain of ABC transporter	0.000	25	5	5	65	259	31.20	31.01	31.54	31.47	31.64	31.63	31.51		0.94	0.2465	-0.33
CLAU_3255	Peptide deformylase	0.004	25	3	3	23	152	31.08	30.93	31.45	31.71	31.56	31.58	31.51		1.34	0.1405	-0.46
CLAU_2954	Response regulator receiver modulated	0.000	34	11	11	215	367	31.36	31.50	31.38	31.50	31.56	31.62	31.50		1.23	0.1599	-0.15
CLAU_2269	Transcription termination factor Rho	0.000	37	13	13	116	484	31.32	31.43	31.31	31.56	31.64	31.62	31.50	+	2.30	0.0437	-0.25
CLAU_1927	Protein arginine kinase McsB	0.000	44	13	13	130	346	30.85	31.35	31.09	32.05	31.85	31.62	31.49		1.76	0.0805	-0.74
CLAU_2889	Hypothetical protein	0.000	26	4	4	93	185	31.56	31.39	31.38	31.38	31.59	31.62	31.48		0.38	0.5745	-0.09
CLAU_2061	ParB-like partition protein	0.000	41	12	12	83	286	31.29	31.20	30.94	31.70	31.80	31.66	31.48		2.16	0.0504	-0.58
CLAU_3252	DNA-directed RNA polymerase subunit (	0.004	33	2	2	19	72	31.28	31.26	31.64	31.73	31.38	31.56	31.47		0.44	0.5285	-0.16
CLAU_2002	2-nitropropane dioxygenase NPD	0.000	34	9	9	101	355	31.63	31.75	31.87	31.19	31.28	31.30	31.47	+	2.51	0.0376	0.49

CLAU_2047	50S ribosomal protein L9	0.000	31	5	5	57	147	31.31	31.36	31.55	31.38	31.50	31.57	31.44		0.35	0.6059	-0.08
CLAU_1358	CRISPR-associated protein	0.000	26	8	8	91	309	31.30	31.22	31.06	31.69	31.60	31.55	31.43		2.17	0.0500	-0.42
CLAU_2053	30S ribosomal protein S6	0.000	61	4	4	77	95	31.07	31.37	31.46	31.29	31.65	31.59	31.42		0.58	0.4287	-0.21
CLAU_3164	Zinc-containing alcohol dehydrogenase	0.000	33	8	8	73	364	31.23	31.17	31.14	31.82	31.79	31.60	31.42	+	2.78	0.0283	-0.56
CLAU_0818	Diguanylate cyclase/phosphodiesterase	0.013	5	3	3	10	635	31.88	32.08	31.64	31.19	30.90	31.08	31.42	+	2.22	0.0480	0.81
CLAU_1271	Maltose-binding protein	0.000	29	11	11	97	401	31.34	31.37	31.55	31.36	31.46	31.45	31.41		0.02	0.9796	0.00
CLAU_3109	hypothetical protein	0.000	40	4	4	59	133	31.40	31.31	31.35	31.42	31.66	31.78	31.41		1.15	0.1799	-0.27
CLAU_3288	hypothetical protein	0.004	11	1	1	31	75	31.03	31.27	31.66	31.87	30.70	31.55	31.41		0.05	0.9399	-0.05
CLAU_3060	Ribosome-binding ATPase ychF	0.000	25	8	8	69	365	31.23	31.37	31.33	31.43	31.59	31.64	31.40		1.49	0.1172	-0.24
CLAU_3202	Ethanolamine utilization EutQ family pro	0.000	18	3	3	76	223	31.48	31.58	31.48	31.31	31.25	31.26	31.40	+	2.49	0.0379	0.24
CLAU_2842	Pyridoxal-5-phosphate-dependent prote	0.000	29	10	10	155	495	30.98	31.00	31.20	31.89	31.75	31.58	31.39	+	2.40	0.0396	-0.68
CLAU_3771	Methyl-accepting chemotaxis sensory tr	0.000	17	4	4	76	273	31.55	31.52	31.67	31.24	30.73	30.99	31.38		1.74	0.0824	0.59
CLAU_3787	Asparagine synthase (glutamine-hydroly	0.000	29	12	12	101	529	31.55	31.57	31.49	31.07	31.09	31.27	31.38	+	2.35	0.0407	0.39
CLAU_0087	ABC-type transporter periplasmic subun	0.000	41	11	11	105	362	28.93	30.15	29.95	32.83	32.94	32.58	31.37	+	2.86	0.0283	-3.11
CLAU_0872	Shikimate kinase	0.000	25	4	4	68	169	32.16	31.84	32.24	30.49	30.61	30.88	31.36	+	2.97	0.0257	1.42
CLAU_2283	UDP-N-acetylglucosamine 2-epimerase	0.000	23	7	7	91	384	31.13	31.38	31.33	31.08	31.42	31.50	31.36		0.13	0.8384	-0.05
CLAU_2006	CoA-binding domain protein	0.004	40	5	5	25	126	31.88	31.70	32.08	29.62	29.94	31.00	31.35		1.77	0.0795	1.70
CLAU_1912	NusG antitermination factor	0.000	46	5	5	49	173	31.17	31.37	31.56	31.28	31.46	31.30	31.34		0.05	0.9297	0.02
CLAU_1235	Preprotein translocase subunit SecD	0.000	29	10	10	130	424	31.39	31.49	31.26	31.20	31.47	31.26	31.33		0.26	0.6812	0.07
CLAU_2961	Flagellar motor switch protein FliN	0.000	40	11	11	117	401	31.15	31.26	31.38	31.28	31.51	31.32	31.30		0.48	0.5022	-0.11
CLAU_1552	Pyrroline-5-carboxylate reductase	0.000	17	5	5	87	270	31.33	31.58	31.56	31.25	31.27	31.17	31.30		1.41	0.1281	0.26
CLAU_2231	Anaerobic ribonucleoside-triphosphate	0.000	14	9	9	81	701	31.28	31.28	31.11	31.30	31.55	31.46	31.29		1.08	0.2010	-0.21
CLAU_1996	3-oxoacyl-(acyl-carrier-protein) synthase	0.000	39	10	10	77	412	30.72	31.26	31.12	31.33	31.36	31.31	31.29		0.86	0.2814	-0.30
CLAU_3266	hypothetical protein	0.000	35	4	4	85	116	31.27	31.31	31.23	31.36	31.29	31.10	31.28		0.09	0.8911	0.02
CLAU_2360	Cell division ATP-binding protein FtsE	0.000	31	6	6	73	228	30.69	31.09	31.12	31.44	31.53	31.50	31.28		1.68	0.0873	-0.52
CLAU_0726	hypothetical protein	0.000	29	6	6	101	272	31.03	31.21	31.25	31.30	31.31	31.36	31.28		1.07	0.2043	-0.16
CLAU_2985	Glycosyl transferase group 1	0.000	20	8	8	66	450	31.53	31.52	31.30	31.22	31.18	31.21	31.26		1.50	0.1143	0.25
CLAU_2948	hypothetical protein	0.000	31	2	2	49	115	29.58	31.09	31.41	31.17	31.40	31.34	31.26		0.46	0.5111	-0.61
CLAU_0113	D-lactate dehydrogenase (cytochrome)	0.000	35	10	9	164	468	31.59	31.70	31.49	30.86	30.80	31.02	31.26	+	2.84	0.0283	0.70
CLAU_0558	ABC-type transport system ATPase comp	0.001	34	7	7	37	231	30.57	31.41	31.60	31.67	31.09	31.02	31.25		0.06	0.9216	-0.07
CLAU_0027	NUDIX hydrolase	0.000	23	6	6	56	273	31.37	31.28	30.97	31.20	31.21	31.58	31.25		0.29	0.6600	-0.12
CLAU_3215	UPF0297 protein	0.000	51	5	5	54	83	30.72	31.01	31.44	31.36	31.17	31.32	31.25		0.45	0.5225	-0.23
CLAU_2840	NGG1p interacting factor 3 protein	0.000	44	10	10	98	271	31.10	30.96	30.94	31.38	31.41	31.47	31.24	+	2.75	0.0285	-0.42
CLAU_2769	Dihydropteroate synthase DHPS	0.000	32	8	8	55	267	30.25	31.40	31.57	31.61	31.08	30.79	31.24		0.06	0.9197	-0.09
CLAU_2764	Dihydropteroate synthase DHPS	0.000	32	8	8	55	267	30.25	31.40	31.57	31.61	31.08	30.79	31.24		0.06	0.9197	-0.09
CLAU_2804	30S ribosomal protein S20	0.000	19	3	3	78	88	31.54	31.52	31.01	30.75	31.30	31.16	31.23		0.53	0.4631	0.29
CLAU_0004	Glyoxylate reductase	0.001	19	5	5	36	324	31.03	30.98	31.25	31.26	31.22	31.24	31.23		0.85	0.2845	-0.15
CLAU_3924	Chemotaxis phosphatase CheX	0.000	18	2	2	50	154	31.15	31.20	31.16	31.25	31.54	31.46	31.23		1.32	0.1451	-0.25
CLAU_1044	NADPH-dependent FMN reductase	0.001	16	3	3	49	210	31.17	30.81	31.16	31.54	31.26	31.26	31.22		0.95	0.2404	-0.31
CLAU_2603	ABC-type transporter periplasmic subun	0.000	34	7	7	86	316	30.59	30.86	31.12	31.45	31.50	31.31	31.22		1.58	0.1022	-0.56
CLAU_3581	4Fe-4S ferredoxin iron-sulfur binding	0.000	40	3	3	78	93	29.92	30.73	30.47	31.84	31.69	31.87	31.21	+	2.36	0.0407	-1.43
CLAU_1990	Phosphoglycerate dehydrogenase	0.001	29	8	8	43	315	31.29	31.23	31.07	31.15	31.18	31.25	31.21		0.02	0.9796	0.00
CLAU_1898	Ribosomal protein L4/L1e	0.000	45	7	7	174	206	31.54	31.68	31.21	30.55	31.19	31.09	31.20		1.03	0.2170	0.53
CLAU_1992	Acetyl-CoA carboxylase carboxyl transfe	0.000	20	4	4	51	291	30.94	30.84	31.24	31.24	31.15	31.25	31.20		0.77	0.3262	-0.21
CLAU_1221	DNA polymerase I	0.000	31	20	20	119	876	31.12	31.26	30.90	30.92	31.52	31.37	31.19		0.35	0.6024	-0.18
CLAU_1993	Acetyl-CoA/biotin carboxylase	0.000	40	14	14	78	447	31.61	31.70	30.00	30.79	30.55	31.59	31.19		0.07	0.9082	0.13

CLAU_3888	Methyl-accepting chemotaxis sensory tr	0.000	28	15	14	116	688	31.17	30.91	30.73	31.20	31.38	31.53	31.19		1.28	0.1534	-0.43
CLAU_2332	4Fe-4S ferredoxin iron-sulfur binding	0.000	35	8	8	66	205	30.97	30.86	30.84	31.77	31.83	31.39	31.18	+	2.24	0.0471	-0.77
CLAU_2448	Carboxyvinyl-carboxyphosphonate	0.000	33	9	9	75	298	29.15	29.44	29.40	33.27	32.96	32.92	31.18	+	4.89	0.0046	-3.72
CLAU_0725	hypothetical protein	0.000	32	12	12	116	459	31.11	31.14	31.09	31.42	31.35	31.21	31.18		1.55	0.1079	-0.21
CLAU_2347	Diacylglycerol kinase catalytic	0.000	41	11	11	78	298	31.24	31.20	30.77	31.06	31.33	31.15	31.18		0.26	0.6873	-0.11
CLAU_1022	Aldo/keto reductase	0.000	25	9	9	104	335	31.16	30.54	31.17	31.04	31.44	31.40	31.17		0.62	0.4000	-0.34
CLAU_3951	Cobalamin B12-binding domain protein	0.000	23	4	4	69	218	31.16	31.16	31.07	30.74	31.23	31.53	31.16		0.05	0.9297	-0.04
CLAU_3221	GTP-binding protein TypA	0.000	21	10	10	109	607	31.12	31.17	31.18	31.14	31.20	31.03	31.16		0.25	0.6996	0.03
CLAU_3903	Cupin 2 conserved barrel domain protei	0.000	51	5	5	70	116	30.88	31.09	31.10	31.32	31.52	31.21	31.16		1.32	0.1440	-0.33
CLAU_0360	Nitrogenase component 2 NifH ATPase	0.000	36	8	8	148	253	30.50	30.91	30.67	31.44	31.41	31.40	31.16	+	2.42	0.0390	-0.72
CLAU_0102	Sigma-54 specific transcriptional regulat	0.000	21	11	9	84	631	31.39	31.03	31.32	30.90	31.27	29.98	31.15		0.59	0.4177	0.53
CLAU_2175	L-ribulose-5-phosphate 4-epimerase	0.000	35	6	6	49	233	31.43	31.39	31.31	30.37	30.63	30.97	31.14		1.81	0.0752	0.72
CLAU_0441	Adenine deaminase	0.019	5	2	2	6	569	27.31	31.18	31.39	31.34	31.10	31.04	31.14		0.38	0.5768	-1.20
CLAU_2613	FAD-linked oxidase domain protein	0.000	46	14	13	157	460	31.47	31.44	31.43	30.69	30.57	30.83	31.13	+	3.23	0.0206	0.75
CLAU_1855	Fumarate hydratase	0.000	21	5	5	60	280	31.10	31.13	31.23	30.97	31.03	31.27	31.12		0.25	0.6948	0.06
CLAU_3123	1-deoxy-D-xylulose-5-phosphate syntha	0.000	20	10	10	67	624	31.02	30.93	30.92	31.32	31.29	31.21	31.12	+	2.64	0.0329	-0.32
CLAU_3685	Phage terminase-like protein small subu	0.353	7	1	1	1	161	31.37	31.37	31.10	30.94	31.08	31.12	31.11		1.04	0.2150	0.23
CLAU_2888	RNA methyltransferase TrmA family	0.000	37	13	13	94	450	31.10	30.98	31.15	30.92	31.16	31.11	31.11		0.05	0.9328	0.01
CLAU_3587	Serpin domain-containing protein	0.000	30	14	14	49	523	30.55	30.84	30.53	31.37	31.64	31.53	31.11	+	2.63	0.0333	-0.87
CLAU_3906	Monosaccharide-transporting ATPase	0.000	23	10	10	75	511	31.25	31.13	31.28	31.06	30.80	30.51	31.10		1.22	0.1622	0.43
CLAU_1649	Helicase superfamily 1 UvrD-related pro	0.000	26	18	18	165	709	30.82	31.07	31.09	31.10	31.36	31.58	31.10		1.01	0.2206	-0.35
CLAU_2521	KWG Leptospira repeat protein	0.000	14	8	8	97	585	31.05	31.13	31.17	31.05	31.13	31.04	31.09		0.41	0.5530	0.04
CLAU_3021	Hypothetical protein	0.004	29	3	3	24	124	31.12	31.17	31.22	30.90	31.06	30.98	31.09		1.60	0.1005	0.19
CLAU_2133	2-hydroxy-3-oxopropionate reductase	0.000	35	7	7	85	297	30.34	29.81	30.16	32.13	31.83	32.08	31.09	+	3.34	0.0187	-1.91
CLAU_1771	Propanediol utilization protein	0.000	44	6	6	54	216	31.64	31.20	31.30	30.90	30.96	30.91	31.08		1.56	0.1059	0.46
CLAU_2501	DNA-binding domain protein excisionas	0.000	34	9	9	67	319	31.17	29.99	31.72	31.01	30.96	31.15	31.08		0.05	0.9308	-0.08
CLAU_2337	Dihydrodipicolinate synthase	0.000	21	4	4	55	293	30.70	30.88	30.81	31.32	31.28	31.29	31.08	+	3.13	0.0230	-0.50
CLAU_1635	Tyrosyl-tRNA synthetase	0.000	18	6	6	77	406	31.23	31.31	30.66	30.89	31.11	31.04	31.08		0.09	0.8903	0.05
CLAU_2281	CMP/dCMP deaminase zinc-binding pro	0.008	20	3	3	12	136	30.66	30.97	31.54	26.98	31.18	31.95	31.08		0.26	0.6860	1.02
CLAU_1099	NLPA lipoprotein	0.000	47	11	11	106	273	31.34	31.25	31.29	30.59	30.49	30.89	31.07	+	2.18	0.0498	0.64
CLAU_1231	(p)ppGpp synthetase I	0.000	29	20	20	152	725	31.24	31.04	30.90	31.26	31.10	30.92	31.07		0.08	0.8940	-0.03
CLAU_3811	Molybdopterin-binding domain-containi	0.000	23	7	7	47	339	29.67	29.68	29.51	32.50	32.45	32.59	31.07	+	5.72	0.0030	-2.89
CLAU_1697	3-hydroxybutyrate dehydrogenase	0.000	33	7	7	80	254	31.57	31.15	31.42	30.98	30.76	30.91	31.07		1.63	0.0959	0.50
CLAU_0154	Translation initiation factor IF-3	0.001	31	5	5	40	172	31.06	31.04	31.34	31.25	31.07	31.03	31.07		0.09	0.8870	0.03
CLAU_1854	Hydro-lyase Fe-S type tartrate/fumarate	0.001	34	4	4	41	188	31.04	31.07	31.44	31.13	30.99	31.05	31.06		0.40	0.5604	0.13
CLAU_1815	Flavoprotein	0.000	40	6	6	60	174	30.86	30.90	30.99	31.88	31.11	31.14	31.05		0.84	0.2894	-0.46
CLAU_3180	Histidinol-phosphate aminotransferase	0.000	39	11	11	84	357	30.99	31.04	31.03	31.08	31.13	31.04	31.04		0.98	0.2292	-0.06
CLAU_2278	Sugar-phosphate isomerase RpiB/LacA/I	0.000	22	4	4	57	149	30.60	30.83	31.02	31.57	31.48	31.06	31.04		1.31	0.1466	-0.55
CLAU_2867	N5-carboxyaminoimidazole ribonucleoti	0.004	8	1	1	23	159	31.16	31.12	31.43	30.72	30.94	30.94	31.03		1.41	0.1279	0.37
CLAU_2170	Serine-pyruvate transaminase	0.000	20	6	6	51	381	30.96	31.16	31.20	31.02	31.03	31.01	31.03		0.51	0.4760	0.09
CLAU_3015	Ig domain-containing protein	0.000	19	19	19	149	1597	30.44	30.17	30.73	31.85	31.37	31.30	31.02		1.96	0.0636	-1.06
CLAU_3810	Xanthine dehydrogenase accessory prot	0.000	31	11	11	47	359	28.28	29.71	28.90	32.58	32.32	32.49	31.02	+	2.94	0.0260	-3.50
CLAU_0805	Cysteine desulfurase	0.006	23	6	6	16	378	31.04	30.95	30.94	30.99	31.27	31.21	31.02		0.93	0.2522	-0.18
CLAU_1074	Sirohydrochlorin cobaltochelatase	0.000	29	6	6	68	274	31.17	31.34	31.51	30.59	30.85	30.66	31.01		2.16	0.0504	0.64
CLAU_0216	Selenium metabolism protein YedF	0.000	31	5	5	48	193	30.56	30.89	30.74	31.21	31.12	31.18	31.01		1.95	0.0646	-0.44
CLAU_2008	Acyl-ACP thioesterase	0.000	35	8	8	109	251	31.08	30.92	30.84	30.79	31.80	31.67	31.00		0.66	0.3757	-0.47

CLAU_0711	Glycerol dehydrogenase	0.001	29	7	7	34	363	31.17	31.37	31.29	30.55	30.83	30.72	31.00	+	2.35	0.0407	0.58
CLAU_3065	UDP-N-acetylmuramoyl-L-alanyl-D-gluta	0.000	31	12	12	90	482	30.75	30.80	30.92	31.05	31.11	31.05	30.99		1.98	0.0614	-0.25
CLAU_2159	SpoOJ/ParA/ParB/repB family protein	0.000	26	9	9	53	416	30.35	30.56	31.34	31.71	30.96	30.99	30.98		0.53	0.4578	-0.47
CLAU_1835	Endoribonuclease L-PSP	0.004	18	3	3	19	124	29.21	30.12	29.20	32.47	32.03	31.81	30.97	+	2.70	0.0309	-2.59
CLAU_0802	hypothetical protein	0.000	26	10	10	80	390	30.85	30.76	30.65	31.08	31.17	31.07	30.96	+	2.23	0.0474	-0.35
CLAU_3073	hypothetical protein	0.000	33	8	8	66	222	31.01	30.83	31.02	30.90	31.03	30.89	30.96		0.06	0.9223	0.01
CLAU_3249	hypothetical protein	0.001	30	9	9	36	305	30.07	29.48	31.96	30.89	31.00	31.42	30.95		0.32	0.6276	-0.60
CLAU_2013	(FeFe)-hydrogenase H-cluster maturatio	0.001	26	8	8	44	411	30.77	30.77	30.86	31.60	31.46	31.02	30.94		1.47	0.1202	-0.56
CLAU_3759	Glutamate synthase NADH/NADPH sma	0.000	27	11	11	94	477	31.41	31.20	31.31	30.16	30.66	30.33	30.93	+	2.36	0.0407	0.92
CLAU_2204	Hypothetical protein	0.000	30	5	5	63	217	31.01	30.84	30.94	30.64	30.92	30.98	30.93		0.29	0.6538	0.08
CLAU_1540	Redox-sensing transcriptional repressor	0.000	35	7	7	57	210	30.67	30.87	30.92	31.05	30.98	30.94	30.93		0.96	0.2378	-0.17
CLAU_2755	Metal dependent phosphohydrolase	0.000	32	5	5	50	191	30.99	30.62	30.96	30.59	30.89	31.24	30.93		0.08	0.8983	-0.05
CLAU_3074	Cell division protein sepF	0.000	44	5	5	106	154	30.47	31.05	31.28	29.97	30.93	30.90	30.92		0.35	0.6042	0.33
CLAU_3710	PemK family protein	0.000	46	6	6	79	133	30.68	30.53	30.71	31.12	31.33	31.11	30.91	+	2.42	0.0393	-0.55
CLAU_1151	Hypothetical protein	0.009	19	5	5	11	319	30.66	31.33	31.44	31.10	30.53	29.99	30.88		0.68	0.3655	0.60
CLAU_3011	Zinc-ribbon domain-containing protein	0.000	29	11	11	47	483	30.85	30.90	31.00	31.08	30.66	30.81	30.88		0.20	0.7586	0.07
CLAU_2960	Flagellar motor switch protein FliM	0.000	15	4	4	63	331	30.76	30.64	30.58	30.99	31.12	31.06	30.88	+	2.44	0.0390	-0.40
CLAU_1695	4-hydroxybutyrate dehydrogenase	0.000	28	8	8	58	371	30.63	30.56	30.69	31.22	31.13	31.06	30.88	+	2.99	0.0254	-0.51
CLAU_1212	Hydrolase ycdX	0.000	46	8	8	98	241	30.68	30.49	31.09	31.03	30.81	30.93	30.87		0.38	0.5765	-0.17
CLAU_1113	Phosphoesterase PA-phosphatase	0.014	3	1	1	15	186	29.55	29.85	30.66	31.34	31.32	31.07	30.87		1.63	0.0961	-1.22
CLAU_1957	Two component transcriptional regulatc	0.000	31	6	6	113	228	30.75	30.47	30.59	30.97	31.18	31.00	30.86		1.89	0.0683	-0.45
CLAU_1255	RNA polymerase sigma-70 factor	0.011	4	1	1	19	251	31.00	30.91	31.00	30.74	30.67	30.81	30.86		1.99	0.0611	0.23
CLAU_2365	UvrABC system protein A	0.000	21	16	16	64	940	30.67	30.73	30.77	31.02	30.94	30.93	30.85	+	2.38	0.0407	-0.24
CLAU_0203	(Dimethylallyl)adenosine tRNA	0.000	29	11	11	80	447	31.09	31.18	30.85	30.81	30.76	30.84	30.85		1.10	0.1951	0.24
CLAU_2028	Amidohydrolase 2	0.000	39	10	10	54	289	29.19	28.91	29.00	32.69	32.54	32.49	30.84	+	5.38	0.0030	-3.54
CLAU_0359	Oxidoreductase/nitrogenase componen	0.000	14	5	5	94	434	30.62	30.60	30.81	30.87	31.16	31.16	30.84		1.52	0.1114	-0.39
CLAU_2639	HTH domain-containing protein	0.001	26	9	9	41	435	30.93	31.00	30.86	30.75	30.82	30.77	30.84		1.52	0.1114	0.15
CLAU_2371	UDP-N-acetylenolpyruvoylglucosamine ı	0.000	26	7	7	81	304	30.85	30.82	31.01	30.70	30.77	30.89	30.84		0.59	0.4210	0.11
CLAU_1081	Cobalt-precorrin-6A synthase	0.000	25	8	8	69	360	30.86	31.02	30.89	30.46	30.79	30.55	30.83		1.37	0.1341	0.32
CLAU_0098	Sigma-54 specific transcriptional regulat	0.000	35	11	11	89	341	30.37	30.36	30.43	31.49	31.65	31.21	30.82	+	2.91	0.0271	-1.06
CLAU_1840	HI0933 family protein	0.000	19	10	10	72	532	30.84	30.86	30.57	31.14	30.42	30.77	30.81		0.03	0.9605	-0.02
CLAU_3057	hypothetical protein	0.000	36	4	4	60	134	30.71	31.02	30.69	30.75	30.90	30.86	30.81		0.09	0.8849	-0.03
CLAU_0568	Carbamoyl-phosphate synthase small su	0.000	31	7	7	55	354	30.01	29.87	30.53	31.11	31.36	31.08	30.81		2.05	0.0565	-1.05
CLAU_2525	hypothetical protein	0.001	16	6	6	42	348	30.75	30.81	30.80	30.83	30.85	30.80	30.81		0.78	0.3174	-0.04
CLAU_3578	DGC domain protein	0.000	53	5	5	62	137	29.31	29.92	30.24	31.34	31.55	31.46	30.79	+	2.36	0.0407	-1.63
CLAU_1205	Phosphoglycerate dehydrogenase	0.003	19	5	5	34	324	30.40	30.94	30.64	30.35	31.22	31.31	30.79		0.36	0.5902	-0.30
CLAU_1729	Cysteine synthase A	0.000	38	9	9	78	303	30.84	30.86	30.86	30.48	30.73	30.44	30.79		1.54	0.1099	0.30
CLAU_1762	Basic membrane lipoprotein	0.000	39	8	8	73	368	30.63	30.69	30.77	30.80	31.02	31.00	30.79		1.40	0.1286	-0.24
CLAU_0792	Spermidine synthase / Polyamine aminc	0.001	32	7	7	43	290	30.66	30.61	30.67	30.89	31.03	30.89	30.78	+	2.35	0.0407	-0.29
CLAU_2454	Hypothetical protein	0.000	7	5	5	49	735	31.36	31.14	30.48	30.58	30.52	30.96	30.77		0.44	0.5275	0.31
CLAU_2065	TRNA uridine 5-carboxymethylaminome	0.001	21	10	10	40	628	30.73	30.86	31.02	30.54	30.78	30.61	30.76		0.96	0.2362	0.23
CLAU_3755	Amidohydrolase	0.000	25	9	9	79	390	30.85	30.95	30.56	30.69	30.76	30.74	30.75		0.18	0.7756	0.06
CLAU_2753	GTPase obg	0.001	26	8	8	37	424	30.66	30.77	30.82	30.94	30.73	30.48	30.75		0.08	0.8945	0.03
CLAU_1302	Phenylalanyl-tRNA synthetase alpha sub	0.001	21	7	7	44	337	30.82	30.54	30.87	30.66	30.73	30.77	30.75		0.08	0.9024	0.02
CLAU_0304	Molybdenum ABC transporter periplasm	0.002	18	4	4	31	273	30.68	30.80	30.89	30.67	30.70	30.80	30.75		0.39	0.5683	0.07
CLAU_2865	Hydrolase	0.004	21	1	1	27	63	30.76	31.81	31.09	30.69	30.67	30.74	30.75		0.77	0.3229	0.52

CLAU_2016	Aspartyl aminopeptidase	0.000	18	6	6	69	434	30.79	30.76	30.61	30.72	30.77	30.71	30.74		0.08	0.8983	-0.01
CLAU_3709	Hypothetical protein	0.001	30	2	2	37	76	30.72	30.52	30.76	30.53	30.97	30.98	30.74		0.41	0.5499	-0.16
CLAU_2524	Cell wall binding repeat 2-containing pro	0.000	31	8	8	82	442	30.38	30.30	30.60	31.03	30.87	30.89	30.74		2.09	0.0538	-0.50
CLAU_2953	Chemotaxis protein CheD	0.000	51	7	7	131	162	30.67	30.84	30.81	30.51	30.59	30.79	30.73		0.66	0.3757	0.14
CLAU_1079	Precorrin-6Y C5,15-methyltransferase	0.001	29	5	5	37	188	31.24	31.04	31.38	29.65	30.41	30.42	30.73		1.75	0.0813	1.06
CLAU_2654	GTP cyclohydrolase 1	0.000	26	4	4	51	185	29.88	30.68	29.84	31.32	31.28	30.77	30.73		1.41	0.1278	-0.99
CLAU_2681	ABC-type transporter permease protein	0.000	26	5	5	52	216	29.53	29.78	30.17	31.57	31.71	31.28	30.73	+	2.78	0.0283	-1.69
CLAU_2747	Radical SAM protein	0.008	8	5	5	13	617	30.03	30.40	29.98	31.62	31.38	31.05	30.73	+	2.34	0.0411	-1.21
CLAU_0111	Electron transfer flavoprotein	0.000	35	7	7	60	267	30.94	30.86	30.93	30.50	30.55	30.57	30.72	+	3.46	0.0182	0.37
CLAU_2528	Capsular exopolysaccharide family	0.001	34	7	7	42	239	30.69	30.50	30.73	30.54	31.04	30.94	30.71		0.52	0.4670	-0.20
CLAU_2132	Type III effector Hrp-dependent outer pr	0.000	28	10	10	46	477	30.08	30.12	29.78	31.53	31.28	31.42	30.70	+	3.40	0.0184	-1.42
CLAU_1210	Methyl-accepting chemotaxis sensory tr	0.000	20	10	10	69	564	30.44	30.39	30.66	30.90	30.77	30.73	30.70		1.45	0.1227	-0.30
CLAU_3181	Imidazole glycerol phosphate synthase s	0.001	28	6	6	44	253	30.68	30.44	30.88	30.82	30.69	30.58	30.69		0.07	0.9072	-0.03
CLAU_2118	Hypothetical protein	0.000	28	9	9	50	369	30.78	30.57	30.23	30.78	30.80	30.54	30.68		0.43	0.5369	-0.18
CLAU_1100	D-alanine-D-alanine ligase	0.000	35	9	9	67	296	31.15	31.11	30.82	30.23	30.28	30.52	30.67		2.12	0.0529	0.68
CLAU_0539	KWG Leptospira repeat protein	0.001	11	3	3	44	302	31.04	28.56	30.90	30.74	30.60	30.44	30.67		0.20	0.7508	-0.43
CLAU_1979	UDP-N-acetylglucosamine	0.000	30	8	8	47	420	30.09	31.06	30.88	30.53	30.60	30.73	30.67		0.06	0.9174	0.06
CLAU_2227	Hypothetical protein	0.000	33	8	8	75	285	30.54	30.58	30.65	30.69	30.79	30.67	30.66		1.21	0.1649	-0.13
CLAU_3286	Signal recognition protein	0.000	24	9	9	73	449	30.62	30.70	30.56	30.60	31.17	31.04	30.66		0.81	0.3037	-0.31
CLAU_2458	UTP-glucose-1-phosphate uridylyltransfe	0.005	11	3	2	20	289	30.73	30.63	30.46	30.64	30.65	30.86	30.65		0.44	0.5262	-0.11
CLAU_3119	N utilization substance protein B	0.000	58	7	7	54	158	30.00	30.30	30.83	30.58	30.85	30.70	30.64		0.58	0.4246	-0.33
CLAU_2186	Peptidase U61 LD-carboxypeptidase A	0.000	34	8	8	74	306	30.25	30.51	30.56	30.71	30.98	30.87	30.64		1.54	0.1099	-0.41
CLAU_3179	1-(5-phosphoribosyl)-5-(5-	0.004	28	6	6	27	237	30.79	30.82	30.86	30.48	30.23	30.10	30.64		2.09	0.0538	0.55
CLAU_2361	FtsX-like permease family	0.000	20	5	5	62	300	30.42	30.18	30.46	30.79	30.81	30.84	30.63	+	2.18	0.0495	-0.46
CLAU_0795	Dihydrodipicolinate synthase	0.000	14	4	4	83	298	30.62	30.62	30.70	30.63	30.31	30.07	30.62		0.88	0.2726	0.31
CLAU_1073	Cobalt-precorrin-6A reductase	0.001	37	7	7	43	258	30.74	30.47	30.85	30.43	30.81	30.50	30.62		0.26	0.6838	0.11
CLAU_0429	putative selenate reductase YgfK	0.000	16	14	14	85	1005	30.58	30.79	30.17	30.69	30.64	30.57	30.61		0.26	0.6872	-0.12
CLAU_2912	Transglutaminase domain-containing pr	0.000	35	12	12	96	475	30.22	30.08	30.40	30.98	30.82	30.84	30.61	+	2.45	0.0390	-0.65
CLAU_3864	Methyl-accepting chemotaxis sensory tr	0.000	10	4	2	59	382	30.49	30.56	30.89	30.82	30.64	30.32	30.60		0.10	0.8754	0.05
CLAU_0483	Methyl-accepting chemotaxis sensory tr	0.000	10	3	1	60	384	30.50	30.57	30.88	30.86	30.62	30.32	30.60		0.09	0.8860	0.05
CLAU_0114	2-dehydropantoate 2-reductase	0.001	17	6	6	46	302	30.77	30.79	30.78	30.06	30.42	30.39	30.60		1.88	0.0697	0.49
CLAU_1138	Phosphoglycerate dehydrogenase	0.001	39	10	10	42	313	30.21	30.17	30.89	31.06	30.70	30.49	30.60		0.50	0.4867	-0.33
CLAU_2179	Sugar (Glycoside-Pentoside-Hexuronide)	0.004	5	2	2	19	457	30.81	30.57	30.50	30.61	30.57	30.73	30.59		0.03	0.9589	-0.01
CLAU_1545	3-hydroxybutyryl-CoA dehydrogenase	0.000	34	7	7	53	314	30.17	30.59	30.34	30.57	30.79	30.74	30.58		1.13	0.1867	-0.33
CLAU_0229	Acetylornithine/succinyldiaminopimelat	0.000	32	8	8	54	390	30.14	30.32	30.51	30.63	31.12	30.87	30.57		1.44	0.1231	-0.55
CLAU_1430	ATP-dependent protease La	0.000	20	14	14	60	774	30.36	30.32	30.76	30.52	30.84	30.61	30.57		0.45	0.5240	-0.18
CLAU_0547	ABC-type transport system periplasmic c	0.000	23	4	4	48	263	30.07	30.00	30.47	30.65	30.77	30.74	30.56		1.64	0.0955	-0.54
CLAU_1463	Germination protein Ger(x)C family	0.013	4	1	1	13	397	30.49	30.76	30.85	30.47	30.48	30.63	30.56		0.65	0.3784	0.17
CLAU_2873	Phosphoribosylamine-glycine ligase	0.000	29	8	8	57	413	30.39	30.54	31.29	30.38	30.75	30.57	30.56		0.23	0.7229	0.17
CLAU_2168	Cellulase	0.000	29	8	8	123	319	30.60	30.27	30.50	30.46	30.67	30.62	30.55		0.47	0.5063	-0.13
CLAU_1995	Acetyl-CoA carboxylase/biotin carboxyl c	0.003	22	2	2	29	170	30.01	30.55	30.55	30.48	30.78	30.70	30.55		0.64	0.3904	-0.28
CLAU_3489	Phospho-2-dehydro-3-deoxyheptonate a	0.000	30	8	8	64	342	30.59	30.50	30.58	30.20	30.52	30.61	30.55		0.37	0.5839	0.11
CLAU_0427	putative selenium metabolism hydrolase	0.000	29	8	8	58	402	30.28	30.52	30.68	30.43	30.68	30.58	30.55		0.20	0.7586	-0.07
CLAU_1561	hypothetical protein	0.000	30	5	5	65	211	30.70	30.64	30.90	30.45	30.29	30.23	30.55		1.84	0.0721	0.42
CLAU_3171	2-dehydro-3-deoxyphosphogluconate	0.000	53	8	8	70	215	30.18	29.95	30.23	30.96	30.95	30.86	30.55	+	3.03	0.0239	-0.80
CLAU_0442	Xanthine/uracil/vitamin C permease	0.000	7	3	3	63	474	30.22	30.55	30.55	30.69	30.27	30.53	30.54		0.13	0.8451	-0.06

CLAU_1248	Hypothetical protein	0.000	39	7	7	55	220	30.34	30.02	30.13	30.95	30.74	30.96	30.54	+	2.44	0.0390	-0.72
CLAU_1975	Glutamate racemase	0.002	26	5	5	30	261	30.19	30.57	30.56	30.50	30.71	30.52	30.54		0.41	0.5499	-0.14
CLAU_1749	Two component transcriptional regulato	0.004	19	4	4	23	232	30.08	30.23	30.08	30.85	31.57	31.17	30.54		2.12	0.0529	-1.07
CLAU_3573	Transcriptional regulator ArsR family	0.083	19	2	2	3	108	29.32	30.19	29.55	31.35	31.41	30.89	30.54		2.12	0.0530	-1.53
CLAU_3264	Thiamine pyrophosphokinase	0.000	39	7	7	59	211	30.39	30.55	29.94	30.63	30.52	30.58	30.54		0.70	0.3581	-0.28
CLAU_1316	Chaperonin	0.001	27	7	7	41	296	30.20	30.56	30.52	30.79	30.54	30.50	30.53		0.56	0.4409	-0.18
CLAU_1104	Metal-dependent RNase	0.011	2	1	1	18	825	30.73	30.44	29.48	30.51	31.03	30.55	30.53		0.51	0.4763	-0.48
CLAU_0276	HPr kinase	0.001	29	7	7	34	306	30.41	30.48	30.34	30.63	30.90	30.57	30.53		1.25	0.1590	-0.29
CLAU_1497	Methyladenine glycosylase	0.028	6	1	1	8	294	30.97	29.42	30.65	30.20	30.37	30.70	30.51		0.05	0.9310	-0.08
CLAU_2478	Hypothetical protein	0.001	36	3	3	33	129	30.55	30.31	30.56	30.46	30.43	30.56	30.51		0.04	0.9525	-0.01
CLAU_0157	Sodium:dicarboxylate symporter	0.016	3	1	1	7	438	31.06	30.77	30.45	30.56	30.37	30.44	30.51		0.75	0.3302	0.30
CLAU_2965	Flagellar hook-associated protein	0.000	37	12	12	102	468	30.48	30.57	30.52	30.47	30.60	30.45	30.50		0.11	0.8603	0.02
CLAU_2618	'HlyD family secretion protein	0.000	23	3	3	55	223	30.44	30.01	30.92	30.51	30.49	30.51	30.50		0.06	0.9213	-0.05
CLAU_0351	Oxidoreductase/nitrogenase componen	0.001	20	5	5	42	380	30.57	30.39	30.37	30.49	30.71	30.51	30.50		0.60	0.4138	-0.13
CLAU_0352	Oxidoreductase/nitrogenase componen	0.005	18	6	6	20	442	30.40	30.49	30.26	30.51	30.73	30.62	30.50		1.21	0.1664	-0.24
CLAU_0226	Urocanate hydratase	0.000	17	9	9	49	675	30.44	30.84	30.80	30.55	29.20	29.58	30.50		1.02	0.2187	0.92
CLAU_3614	Indole-3-glycerol phosphate synthase	0.001	12	3	3	35	258	27.50	27.65	29.00	32.10	31.98	32.14	30.49	+	2.96	0.0257	-4.02
CLAU_3024	Methyl-accepting chemotaxis sensory tr	0.005	5	3	2	16	669	30.39	30.56	30.58	30.47	30.43	30.50	30.49		0.27	0.6725	0.04
CLAU_2589	Hypothetical protein	0.000	27	11	11	62	484	30.43	30.46	30.20	30.56	30.70	30.50	30.48		1.04	0.2170	-0.22
CLAU_1969	2-phosphosulfolactate phosphatase	0.007	24	5	5	15	236	30.33	30.43	30.42	30.54	30.53	30.74	30.48		1.30	0.1466	-0.21
CLAU_3016	hypothetical protein	0.000	39	6	6	51	250	30.21	30.38	30.69	30.66	30.49	30.47	30.48		0.30	0.6442	-0.11
CLAU_1089	Threonine-phosphate decarboxylase	0.001	17	7	7	37	412	30.66	30.56	30.50	30.16	30.45	30.23	30.48		1.38	0.1327	0.29
CLAU_0356	Hydrogenase assembly chaperone hypC	0.000	41	2	2	100	68	29.14	30.39	30.29	30.55	30.58	30.73	30.47		0.77	0.3213	-0.68
CLAU_1852	Citryl-CoA lyase	0.001	28	8	8	40	290	30.51	30.42	30.36	30.39	30.54	30.58	30.47		0.43	0.5314	-0.07
CLAU_2176	Carbohydrate kinase FGGY	0.000	18	9	9	68	534	30.48	30.60	30.44	30.43	30.24	30.44	30.44		0.78	0.3191	0.14
CLAU_0461	Sorbitol operon regulator	0.000	26	7	7	60	327	30.47	30.29	30.36	30.41	30.57	30.50	30.44		0.79	0.3126	-0.12
CLAU_0430	Carbamoyltransferase YgeW	0.001	14	5	5	42	398	30.25	29.94	30.41	30.56	30.45	30.49	30.43		0.99	0.2267	-0.30
CLAU_0131	hypothetical protein	0.007	17	3	3	14	172	30.19	30.12	30.37	30.77	30.46	30.47	30.42		1.27	0.1547	-0.34
CLAU_1275	Glycosyl transferase family 2	0.017	15	4	4	8	333	29.37	29.34	29.92	31.41	31.12	30.91	30.42	+	2.60	0.0339	-1.60
CLAU_2018	Cupin 2 conserved barrel domain protei	0.000	32	3	3	51	111	30.81	30.82	30.98	29.77	29.50	30.01	30.41	+	2.67	0.0318	1.11
CLAU_2941	Ornithine carbamoyltransferase	0.000	38	10	10	58	333	30.28	30.92	30.87	30.53	29.22	29.34	30.41		1.00	0.2241	0.99
CLAU_0160	Hypothetical protein	0.004	5	1	1	30	129	30.73	30.52	29.82	30.38	30.41	30.40	30.41		0.05	0.9350	-0.04
CLAU_0444	Transcriptional regulator with sigma-54	0.000	14	8	5	88	642	30.76	30.38	30.34	30.37	30.93	30.42	30.40		0.13	0.8375	-0.08
CLAU_1506	Ribonuclease PH	0.002	41	8	8	31	248	30.42	30.38	30.30	31.96	29.97	30.42	30.40		0.28	0.6690	-0.42
CLAU_0051	putative signal transduction protein	0.064	2	1	1	4	448	30.29	30.46	30.50	30.24	30.54	30.33	30.40		0.16	0.8035	0.05
CLAU_2547	DTDP-glucose 4,6-dehydratase	0.001	23	7	5	35	351	29.75	30.31	30.25	30.46	30.59	30.62	30.39		1.16	0.1780	-0.45
CLAU_0367	FAD-binding oxidoreductase	0.000	35	12	11	126	460	30.63	30.76	30.61	28.75	29.86	30.14	30.38		1.19	0.1703	1.08
CLAU_1026	Methyl-accepting chemotaxis protein	0.001	25	5	5	44	277	30.54	30.41	30.33	30.12	30.47	30.04	30.37		0.68	0.3676	0.22
CLAU_2808	GTP-binding protein lepA	0.001	18	8	8	35	602	30.55	30.14	30.45	30.32	30.41	30.33	30.37		0.07	0.9059	0.03
CLAU_0437	Molybdopterin-family oxidoreductase	0.000	17	9	9	60	704	30.71	30.68	30.25	30.15	30.41	30.32	30.37		0.69	0.3605	0.25
CLAU_2903	Cardiolipin synthase	0.201	1	1	1	1	507	30.15	30.29	30.42	30.83	30.46	30.31	30.37		0.64	0.3845	-0.25
CLAU_1094	NUDIX hydrolase	0.000	43	6	6	49	182	29.93	29.99	30.40	30.31	30.42	30.46	30.36		0.87	0.2736	-0.29
CLAU_3226	Twitching motility protein	0.000	39	12	12	66	351	30.58	30.53	30.25	30.45	30.18	30.20	30.35		0.59	0.4222	0.18
CLAU_0862	CBS domain-containing membrane prot	0.000	39	5	5	53	144	30.54	30.39	29.95	29.89	30.30	30.51	30.35		0.08	0.8945	0.06
CLAU_3271	hypothetical protein	0.001	39	7	7	40	174	30.32	29.96	29.97	30.55	30.37	30.68	30.35		1.41	0.1281	-0.45
CLAU_0358	Oxidoreductase/nitrogenase componen	0.000	30	8	8	63	353	29.93	29.27	30.22	30.88	30.45	30.84	30.34		1.37	0.1347	-0.92

CLAU_3167	6-phosphogluconate dehydrogenase	0.001	31	7	7	45	298	30.06	30.10	29.86	30.82	30.57	30.63	30.34	+	2.49	0.0379	-0.67
CLAU_2270	50S ribosomal protein L31	0.007	20	1	1	14	70	29.42	30.50	30.63	30.31	30.26	30.36	30.34		0.12	0.8505	-0.13
CLAU_3122	Polyprenyl synthetase / Geranyltranstra	0.000	23	6	6	55	293	30.29	30.36	30.05	30.45	30.47	30.23	30.33		0.55	0.4491	-0.15
CLAU_2572	Type IV pilin N-term methylation site GF	0.033	13	2	2	5	175	30.59	30.56	30.63	29.83	29.87	30.09	30.33	+	2.87	0.0283	0.66
CLAU_3260	Protein serine/threonine phosphatase	0.001	39	7	7	41	239	30.22	30.15	30.16	30.42	30.67	30.55	30.32		2.10	0.0537	-0.37
CLAU_1719	Cell wall hydrolase/autolysin	0.158	4	1	1	2	238	29.25	28.08	29.88	30.75	31.62	31.49	30.32		1.70	0.0862	-2.22
CLAU_1101	Alanine racemase	0.000	26	7	7	142	388	30.28	30.34	30.60	30.05	30.18	30.36	30.31		0.72	0.3456	0.21
CLAU_2444	Carbamoyl phosphate synthase small su	0.001	10	3	3	37	349	30.21	30.27	30.15	30.35	30.66	30.42	30.31		1.25	0.1582	-0.27
CLAU_3612	Glutamine amidotransferase of anthrani	0.001	38	7	7	38	201	27.75	26.53	28.46	32.41	32.16	32.43	30.31	+	2.95	0.0260	-4.75
CLAU_2544	Glycosyl transferase group 1	0.004	10	7	7	28	784	29.13	29.77	29.46	30.84	30.89	30.93	30.31	+	2.81	0.0283	-1.43
CLAU_0733	DegT/DnrJ/EryC1/StrS aminotransferase	0.001	23	7	7	39	392	30.00	30.28	30.32	30.21	30.45	30.46	30.30		0.60	0.4138	-0.17
CLAU_0379	hypothetical protein	0.000	18	13	13	82	805	30.15	30.12	29.81	30.53	30.49	30.45	30.30		1.85	0.0717	-0.46
CLAU_2814	Ribosomal protein L11 methyltransferas	0.002	21	5	5	34	313	30.61	30.60	30.33	29.97	30.26	30.21	30.30		1.34	0.1413	0.37
CLAU_2072	Chromosomal replication initiator prote	0.000	22	9	9	63	450	30.28	30.70	30.12	30.38	30.26	30.30	30.29		0.11	0.8641	0.05
CLAU_3435	hypothetical protein	0.000	23	6	6	47	366	29.79	30.12	30.10	30.64	30.74	30.45	30.29		1.95	0.0646	-0.61
CLAU_0091	Sigma-54 specific transcriptional regulat	0.000	16	9	4	74	632	30.14	30.02	29.85	30.56	31.17	30.43	30.29		1.38	0.1331	-0.72
CLAU_0425	Dihydropyrimidinase	0.000	25	9	9	91	470	30.12	29.99	30.20	30.43	30.75	30.36	30.28		1.42	0.1275	-0.41
CLAU_2622	Gamma-glutamyl kinase	0.001	36	6	6	42	266	29.99	30.52	30.48	30.30	30.26	30.21	30.28		0.16	0.8035	0.07
CLAU_2855	Serine protease with trypsin-like peptid	0.000	27	8	8	56	412	30.14	30.41	29.94	30.07	30.69	30.82	30.28		0.61	0.4079	-0.36
CLAU_3325	Ribosomal protein S12 methylthiotransf	0.000	29	11	11	60	440	30.42	30.11	30.63	30.26	30.28	29.98	30.27		0.52	0.4665	0.21
CLAU_1487	Hypothetical protein	0.000	17	8	8	48	569	30.41	30.02	30.09	30.22	30.31	30.33	30.27		0.38	0.5744	-0.11
CLAU_3342	Nicotinate phosphoribosyltransferase	0.000	16	7	7	51	493	29.99	30.68	29.18	30.62	30.53	30.00	30.27		0.38	0.5725	-0.43
CLAU_0337	Cyclophilin-type peptidyl-prolyl cis-trans	0.000	39	5	5	54	174	30.03	30.21	30.64	30.20	30.31	30.31	30.26		0.04	0.9527	0.02
CLAU_0217	Omega-amidase	0.000	23	6	6	62	277	29.98	30.28	30.04	30.23	30.39	30.31	30.26		0.96	0.2386	-0.21
CLAU_3538	Nitroreductase	0.000	19	3	3	54	177	30.33	30.12	30.14	30.18	30.46	30.53	30.26		0.70	0.3577	-0.19
CLAU_2739	Penicillin-binding Protein dimerization d	0.000	19	12	12	50	895	30.18	30.45	30.43	30.32	30.06	30.18	30.25		0.66	0.3762	0.17
CLAU_1724	Competence-damaged protein	0.000	24	7	7	54	412	30.22	30.37	30.37	29.80	30.27	30.18	30.25		0.71	0.3534	0.24
CLAU_0224	Histidine ammonia-lyase	0.000	16	5	5	55	507	31.23	30.62	30.60	29.22	29.53	29.86	30.23		2.00	0.0605	1.28
CLAU_1949	MazG family protein	0.000	21	10	10	60	481	30.36	29.55	30.17	30.37	30.26	29.75	30.22		0.12	0.8536	-0.10
CLAU_1008	Methyl-accepting chemotaxis sensory tr	0.002	19	5	5	30	325	31.09	30.30	30.42	29.74	30.13	29.89	30.22		1.19	0.1719	0.68
CLAU_2628	Peptidase family S41 protein	0.009	17	7	7	13	534	30.08	29.80	30.15	30.29	30.33	30.28	30.22		1.26	0.1559	-0.29
CLAU_3592	Diguanylate cyclase with PAS/PAC sensc	0.000	22	15	14	62	725	30.22	30.20	30.26	30.10	30.08	30.30	30.21		0.39	0.5683	0.07
CLAU_0153	Purine nucleoside phosphorylase I inosir	0.000	39	6	6	52	281	30.25	29.98	30.26	30.33	30.16	30.13	30.21		0.15	0.8194	-0.04
CLAU_1776	Respiratory-chain NADH dehydrogenase	0.000	26	9	9	58	442	30.43	30.34	30.54	29.80	29.64	30.06	30.20		1.95	0.0643	0.60
CLAU_3955	Hypothetical protein	0.000	21	5	5	46	281	30.09	30.30	30.25	29.71	30.15	30.23	30.19		0.46	0.5180	0.18
CLAU_1278	Polysaccharide biosynthesis protein Cap	0.000	21	9	9	59	612	29.65	30.19	29.52	30.16	30.35	30.22	30.18		1.01	0.2220	-0.46
CLAU_1991	Acetyl-CoA carboxylase carboxyl transfe	0.003	13	4	4	30	281	30.06	30.18	30.17	29.94	30.26	30.35	30.18		0.13	0.8375	-0.05
CLAU_2062	Cobyrinic acid a,c-diamide synthase	0.000	22	4	4	55	255	29.60	29.20	29.89	30.79	30.44	30.66	30.17		2.05	0.0567	-1.07
CLAU_0034	Cold-shock DNA-binding domain proteir	0.001	55	3	3	41	65	30.67	30.89	30.93	29.11	29.47	29.66	30.17	+	2.85	0.0283	1.42
CLAU_2687	TRNA synthetase class II (D K and N)	0.004	17	5	5	25	368	30.43	30.48	30.60	29.39	29.71	29.90	30.17	+	2.22	0.0479	0.84
CLAU_3776	Flavodoxin	0.014	15	2	2	8	149	28.49	28.84	28.80	31.77	31.64	31.49	30.17	+	4.54	0.0046	-2.92
CLAU_0282	Peptide deformylase	0.001	42	5	5	38	156	30.14	30.15	30.12	30.16	30.40	30.39	30.16		1.07	0.2043	-0.18
CLAU_1040	Alcohol dehydrogenase	0.004	15	4	4	23	389	29.97	30.08	30.11	30.20	30.24	30.46	30.16		1.27	0.1546	-0.25
CLAU_0453	Transcriptional regulator with sigma-54	0.000	15	7	1	82	632	30.24	29.72	29.96	30.20	30.85	30.10	30.15		0.67	0.3730	-0.41
CLAU_3189	Histidinol phosphate phosphatase HisJ f	0.001	26	5	5	43	257	29.88	30.18	30.28	30.03	30.10	30.18	30.14		0.03	0.9653	0.01
CLAU_1932	UDP-N-acetylmuramoylalanine-D-glutar	0.000	33	12	12	76	458	30.07	29.95	29.86	30.19	30.68	30.36	30.13		1.35	0.1393	-0.45

CLAU_0472	Homoserine O-succinyltransferase	0.002	28	7	7	31	305	30.51	30.46	30.74	28.88	29.80	29.34	30.13		1.93	0.0651	1.23
CLAU_1439	Orotidine 5'-phosphate decarboxylase	0.002	18	5	5	31	289	29.99	30.21	30.10	29.48	30.29	30.15	30.13		0.19	0.7683	0.13
CLAU_0375	RND-type efflux system membrane fusic	0.000	38	11	11	59	421	30.01	29.64	29.91	30.35	30.60	30.23	30.12		1.60	0.1005	-0.54
CLAU_3837	Hypothetical protein	0.001	20	5	5	39	249	29.88	29.69	29.92	30.58	30.47	30.31	30.12	+	2.38	0.0407	-0.62
CLAU_3705	Hypothetical protein	0.153	2	2	2	2	1230	29.87	30.16	30.07	30.19	29.64	30.31	30.12		0.02	0.9720	-0.01
CLAU_2935	DJ-1 family protein	0.000	34	6	6	46	184	30.10	29.51	30.12	30.03	30.27	30.25	30.11		0.57	0.4350	-0.27
CLAU_0373	ABC-type transport system ATPase com	0.001	15	3	3	33	241	30.04	30.11	30.07	30.09	30.11	30.13	30.10		0.72	0.3471	-0.04
CLAU_2674	(R)-citramalate synthase	0.000	25	10	10	73	453	30.25	30.34	30.42	29.66	29.92	29.59	30.09	+	2.27	0.0459	0.61
CLAU_0793	Aldo/keto reductase / Methylglyoxal rec	0.002	26	6	6	34	329	30.05	29.98	30.44	29.96	30.12	30.12	30.09		0.23	0.7194	0.09
CLAU_0905	Transcriptional regulator GntR family wi	0.001	11	6	6	35	484	30.56	30.37	30.20	29.45	29.00	29.96	30.08		1.42	0.1259	0.91
CLAU_1214	UDP-galactopyranose mutase	0.001	32	10	10	36	403	30.13	30.02	29.25	31.03	29.61	31.41	30.08		0.65	0.3806	-0.88
CLAU_0446	hypothetical protein	0.000	26	9	9	78	368	29.98	29.42	30.31	30.00	30.25	30.13	30.07		0.34	0.6086	-0.22
CLAU_3276	hypothetical protein	0.004	26	4	4	23	167	29.59	30.17	29.59	30.14	30.36	29.92	30.03		0.70	0.3561	-0.36
CLAU_0090	PAS modulated sigma54 specific transcr	0.000	17	7	7	50	446	29.29	29.50	29.29	31.31	31.58	30.54	30.02	+	2.30	0.0437	-1.78
CLAU_0376	RND-type efflux system membrane perr	0.000	15	11	11	51	1046	29.93	29.84	29.97	30.07	30.05	30.25	30.01		1.32	0.1439	-0.21
CLAU_2679	Hydrolase containing phosphate-binding	0.001	20	4	4	47	228	30.07	29.93	30.03	29.99	29.99	30.06	30.01		0.02	0.9688	0.00
CLAU_0465	Cyclase family protein	0.001	45	8	8	44	244	30.76	31.27	30.31	29.53	29.00	29.69	30.00		1.78	0.0792	1.37
CLAU_1527	Glucose-6-phosphate isomerase	0.001	17	6	6	33	450	30.12	29.88	29.04	28.88	30.38	30.17	30.00		0.08	0.8983	-0.13
CLAU_2067	Single-stranded nucleic acid binding R3H	0.001	32	6	6	37	208	29.84	29.90	29.96	30.03	30.43	30.12	30.00		1.09	0.1968	-0.29
CLAU_0225	Imidazolonepropionase	0.009	18	5	5	13	417	30.28	30.29	30.12	29.66	29.78	29.87	30.00	+	2.30	0.0435	0.46
CLAU_1961	Purine operon repressor PurR	0.001	24	6	6	42	275	29.91	29.83	29.73	30.10	30.26	30.08	30.00		1.86	0.0710	-0.32
CLAU_2372	UPF0042 nucleotide-binding protein	0.004	19	4	4	22	294	29.43	29.41	29.88	30.61	30.51	30.11	30.00		1.74	0.0817	-0.84
CLAU_3455	AAA-ATPase-like protein	0.000	16	10	10	52	558	29.61	29.67	29.85	30.38	30.32	30.12	29.99	+	2.21	0.0483	-0.56
CLAU_0822	Hypothetical protein	0.015	7	4	4	8	505	29.76	25.75	29.30	30.26	30.21	30.52	29.99		0.74	0.3352	-2.06
CLAU_1230	D-tyrosyl-tRNA(Tyr) deacylase	0.002	24	3	3	31	149	29.62	29.73	29.70	30.40	30.39	30.23	29.98	+	3.29	0.0191	-0.66
CLAU_1834	Threonine synthase	0.004	34	10	10	28	419	27.86	28.51	28.74	31.69	31.51	31.22	29.98	+	3.32	0.0187	-3.10
CLAU_1765	C_GCAxxG_C_C family protein	0.004	24	5	5	22	305	30.02	29.27	29.93	30.22	29.91	30.65	29.98		0.75	0.3330	-0.52
CLAU_0607	Alpha/beta hydrolase-like family proteir	0.003	25	7	7	30	398	30.16	29.81	29.72	29.74	30.42	30.12	29.97		0.34	0.6099	-0.20
CLAU_2310	Tex-like protein	0.000	29	15	15	61	721	30.34	30.28	29.27	29.41	29.80	30.12	29.96		0.18	0.7846	0.19
CLAU_2060	Hypothetical protein	0.001	22	3	3	36	167	29.82	29.76	30.06	30.02	30.09	29.90	29.96		0.50	0.4813	-0.12
CLAU_3344	Response regulator	0.003	25	3	3	29	125	29.76	29.68	29.95	29.97	29.98	29.97	29.96		1.04	0.2170	-0.18
CLAU_0326	Transcriptional regulator PadR-like famil	0.004	55	5	5	24	127	28.84	29.50	28.23	30.42	30.82	30.63	29.96		2.00	0.0606	-1.77
CLAU_1582	UPF0251 protein	0.004	24	4	4	25	157	28.46	28.40	28.00	31.66	31.42	31.59	29.94	+	4.46	0.0046	-3.27
CLAU_2656	2-amino-4-hydroxy-6-	0.004	13	3	3	21	275	29.85	30.01	29.81	29.86	30.21	30.19	29.94		0.70	0.3586	-0.20
CLAU_3812	Diguanylate cyclase (GGDEF) domain pr	0.004	9	6	6	18	767	29.68	29.81	29.42	30.52	30.05	30.30	29.93		1.67	0.0895	-0.65
CLAU_2136	Transcriptional regulator TetR family	0.012	20	3	3	10	192	29.46	29.89	29.93	29.93	29.99	29.99	29.93		0.62	0.3982	-0.21
CLAU_1276	Glycosyl transferase	0.002	31	10	10	35	370	29.74	29.24	29.50	30.19	30.57	30.11	29.93		1.77	0.0796	-0.80
CLAU_1418	hypothetical protein	0.004	14	1	1	24	64	29.30	29.98	29.86	30.27	29.80	30.02	29.92		0.56	0.4376	-0.32
CLAU_1306	Translation initiation factor IF-3	0.000	25	4	4	52	173	29.98	29.85	30.50	29.84	30.16	29.82	29.92		0.30	0.6426	0.17
CLAU_2396	6-pyruvoyl tetrahydrobiopterin synthase	0.001	11	11	11	43	1018	30.24	30.07	30.21	29.38	29.54	29.76	29.92		2.13	0.0522	0.61
CLAU_3129	Sporulation transcriptional activator Spc	0.001	37	9	9	34	271	29.87	29.70	29.08	29.95	30.08	29.95	29.91		0.84	0.2871	-0.44
CLAU_0519	Cell wall binding repeat 2-containing prc	0.000	31	12	12	50	572	30.21	29.50	29.60	29.48	30.53	32.49	29.91		0.51	0.4742	-1.06
CLAU_1234	Preprotein translocase subunit SecF	0.001	25	5	5	42	291	29.80	29.90	29.89	29.84	30.09	30.03	29.90		0.69	0.3626	-0.12
CLAU_1633	Universal protein YeaZ	0.004	19	4	4	28	237	29.58	29.56	29.20	30.21	30.22	30.27	29.90	+	2.49	0.0379	-0.79
CLAU_0720	BirA bifunctional protein biotin operon	0.000	29	8	8	50	329	29.62	29.56	29.83	30.11	30.15	29.95	29.89		1.76	0.0801	-0.40
CLAU_0264	Hypothetical protein	0.001	42	4	4	36	108	29.98	30.06	30.54	29.65	29.80	29.71	29.89		1.23	0.1601	0.47

CLAU_2153	Hypothetical protein	0.001	47	7	7	39	227	29.87	29.89	29.68	29.64	30.13	29.94	29.88		0.22	0.7262	-0.09
CLAU_0860	ABC-type transporter periplasmic subun	0.009	18	3	3	11	282	29.43	29.92	29.87	30.21	29.84	29.89	29.88		0.55	0.4491	-0.24
CLAU_3802	Response regulator receiver protein	0.002	36	7	7	31	262	29.97	30.07	30.02	29.50	29.77	29.71	29.87		1.85	0.0721	0.36
CLAU_2909	Methyl-accepting chemotaxis sensory tr	0.000	15	6	6	69	572	30.12	30.16	29.53	29.63	29.73	30.00	29.87		0.26	0.6872	0.15
CLAU_3220	hypothetical protein	0.001	19	2	2	44	119	30.14	29.70	30.01	29.70	29.85	29.88	29.87		0.42	0.5408	0.14
CLAU_2285	ATP synthase subunit a	0.001	10	3	3	38	227	29.59	30.12	29.67	29.84	29.87	30.22	29.86		0.37	0.5810	-0.18
CLAU_2051	30S ribosomal protein S18	0.001	28	3	3	54	85	30.62	30.10	29.14	29.29	30.27	29.60	29.85		0.17	0.7902	0.23
CLAU_2080	3H domain-containing protein	0.002	33	4	4	31	171	29.12	29.54	29.80	30.20	30.02	29.90	29.85		1.20	0.1675	-0.55
CLAU_0033	Flavodoxin	0.007	13	2	2	13	161	29.90	29.74	29.68	29.85	29.84	29.92	29.85		0.62	0.4010	-0.10
CLAU_3497	Rubredoxin-like zinc ribbon protein	0.001	27	3	3	43	146	29.48	29.21	29.68	30.01	30.11	30.00	29.84		1.85	0.0721	-0.58
CLAU_1742	Butyryl-CoA dehydrogenase	0.004	14	5	5	28	390	29.85	29.57	29.49	29.83	29.88	29.95	29.84		1.02	0.2187	-0.25
CLAU_3823	Yip1 domain-containing protein	0.007	4	1	1	14	227	29.69	29.77	29.78	30.09	30.00	29.90	29.84		1.81	0.0758	-0.25
CLAU_2624	Transcriptional Coactivator p15	0.001	39	3	3	45	74	29.42	29.73	30.42	29.98	29.75	29.90	29.83		0.02	0.9696	-0.02
CLAU_1556	hypothetical protein	0.004	23	4	4	25	194	29.85	30.83	29.64	29.79	29.83	29.82	29.83		0.33	0.6204	0.29
CLAU_0038	Vesicle tethering protein	0.001	50	5	5	44	109	29.27	29.48	29.81	30.05	29.81	30.30	29.81		1.19	0.1719	-0.53
CLAU_1298	DNA mismatch repair protein mutS2	0.002	12	8	8	31	786	29.90	29.41	29.72	29.49	30.49	30.13	29.81		0.48	0.4985	-0.36
CLAU_0074	Methyl-accepting chemotaxis sensory tr	0.021	6	3	3	6	668	29.50	29.59	29.42	30.02	30.17	30.13	29.81	+	3.09	0.0231	-0.60
CLAU_3307	1-deoxy-D-xylulose 5-phosphate reducta	0.000	21	6	6	49	385	29.55	29.75	29.85	29.97	29.95	29.75	29.80		0.70	0.3567	-0.17
CLAU_2714	Molybdenum cofactor biosynthesis	0.001	18	5	5	43	399	29.67	30.05	29.53	29.53	29.94	29.92	29.80		0.08	0.8983	-0.05
CLAU_3170	Phosphoglycerate dehydrogenase	0.001	28	7	7	34	307	28.75	28.78	30.01	29.84	29.75	29.84	29.80		0.69	0.3614	-0.63
CLAU_1606	putative transcriptional regulator	0.000	18	8	8	46	480	29.41	29.79	29.40	29.89	29.92	29.79	29.79		1.17	0.1753	-0.33
CLAU_2166	Cellulase	0.004	16	5	5	23	343	29.44	29.33	29.82	29.89	29.76	29.91	29.79		0.97	0.2324	-0.32
CLAU_1504	Phosphodiesterase MJ0936 family	0.012	19	3	3	11	156	29.55	29.79	29.89	29.96	29.61	29.79	29.79		0.11	0.8639	-0.04
CLAU_3050	hypothetical protein	0.004	34	4	4	26	160	30.30	29.91	30.06	29.22	29.27	29.66	29.79		1.77	0.0796	0.71
CLAU_2923	Nitrate reductase subunit beta	0.004	27	4	4	20	151	30.24	30.22	30.00	29.16	29.31	29.56	29.78	+	2.36	0.0407	0.81
CLAU_2761	L-seryl-tRNA(Sec) selenium transferase	0.001	30	11	11	36	460	29.18	29.48	29.27	30.06	30.09	30.10	29.77	+	3.00	0.0249	-0.77
CLAU_1558	Pyridine nucleotide-disulfide oxidoreduc	0.003	15	7	7	29	421	29.54	29.46	29.65	29.92	29.88	29.88	29.77	+	2.43	0.0390	-0.34
CLAU_0155	Response regulator receiver protein	0.001	16	4	4	33	308	29.09	29.43	29.53	29.99	30.32	30.09	29.76		2.04	0.0567	-0.78
CLAU_0112	Electron transfer flavoprotein	0.001	33	8	8	35	332	30.32	29.87	30.10	29.64	29.56	29.40	29.76		1.72	0.0839	0.56
CLAU_3407	putative methyl-accepting chemotaxis p	0.004	6	3	2	26	594	29.77	29.73	29.60	29.50	29.86	29.94	29.75		0.17	0.7850	-0.07
CLAU_0965	putative basic membrane protein	0.000	37	10	10	66	375	30.87	29.53	30.39	29.63	29.39	29.86	29.75		0.70	0.3571	0.64
CLAU_1507	AIR synthase related protein domain pro	0.000	22	8	8	64	329	29.72	29.33	29.76	29.67	29.94	30.39	29.74		0.72	0.3444	-0.40
CLAU_3265	50S ribosomal protein L28	0.001	33	2	2	42	63	28.76	29.08	29.96	29.83	29.85	29.64	29.74		0.62	0.3968	-0.51
CLAU_2586	Hypothetical protein	0.004	30	7	7	19	347	29.17	29.82	30.51	30.37	29.45	29.62	29.72		0.01	0.9816	0.02
CLAU_2917	FeS-binding protein	0.003	20	6	6	31	317	30.10	30.02	30.10	29.28	29.41	29.27	29.72	+	3.87	0.0107	0.75
CLAU_1694	Acetolactate synthase	0.000	25	9	9	52	556	29.59	29.38	29.24	29.83	30.01	29.89	29.71		1.94	0.0651	-0.51
CLAU_0804	putative tRNA sulfurtransferase	0.001	10	5	5	42	380	29.89	29.70	29.70	29.66	29.72	29.81	29.71		0.16	0.7989	0.03
CLAU_2963	FlgN family protein	0.004	21	3	3	24	135	29.16	28.89	29.74	29.79	29.68	29.92	29.71		0.96	0.2378	-0.53
CLAU_1994	(3R)-hydroxymyristoyl-(acyl-carrier-prot	0.004	11	2	2	28	151	29.26	29.91	28.33	29.92	29.68	29.74	29.71		0.59	0.4197	-0.61
CLAU_1034	Diguanylate cyclase	0.003	25	5	5	28	302	29.51	29.47	29.32	30.07	29.88	29.91	29.70	+	2.49	0.0379	-0.52
CLAU_3569	Arsenite methyltransferase	0.004	26	6	6	26	272	29.11	29.66	29.77	29.73	29.56	29.82	29.70		0.36	0.5905	-0.19
CLAU_2030	Carbamate kinase	0.000	30	7	7	54	312	26.02	27.39	26.56	32.58	32.42	31.96	29.68	+	3.68	0.0138	-5.66
CLAU_2335	Transcriptional regulator IclR family	0.000	25	6	6	98	259	29.78	29.27	29.56	29.66	29.89	29.68	29.67		0.56	0.4434	-0.21
CLAU_2742	Cell division topological specificity facto	0.003	39	3	3	29	88	28.99	29.59	29.76	29.73	29.64	29.70	29.67		0.44	0.5254	-0.24
CLAU_1650	Peptidase M41 FtsH domain protein	0.000	26	12	12	49	617	30.12	29.94	29.61	29.32	29.72	29.54	29.67		0.90	0.2646	0.36
CLAU_0415	Transcriptional regulator PucR family	0.149	2	1	1	2	560	29.99	28.88	29.71	29.58	29.88	29.62	29.67		0.18	0.7736	-0.17

CLAU_0535	Copper ion binding protein	0.000	32	2	2	73	71	29.74	29.51	29.71	29.20	29.75	29.61	29.66		0.30	0.6443	0.13
CLAU_1075	Cobalt-precorrin-3B C17-methyltransfer	0.001	13	2	2	38	240	29.23	29.41	29.66	29.65	30.11	29.84	29.66		1.12	0.1895	-0.43
CLAU_1547	Two component transcriptional regulato	0.004	11	3	2	30	256	29.63	29.67	29.21	30.47	29.50	30.00	29.65		0.70	0.3566	-0.49
CLAU_0421	Anaerobic sulfite reductase subunit C	0.004	15	5	5	28	315	29.91	30.33	30.10	29.38	28.88	29.22	29.65		2.12	0.0528	0.95
CLAU_2542	DegT/DnrJ/EryC1/StrS aminotransferase	0.001	29	8	8	35	365	29.09	28.99	29.42	29.85	30.17	29.92	29.64		2.13	0.0524	-0.81
CLAU_1366	Polyketide cyclase/dehydrase	0.006	5	1	1	30	132	30.43	28.39	30.57	29.69	29.44	29.58	29.64		0.12	0.8549	0.23
CLAU_2124	Transcriptional regulator IclR family	0.008	22	5	5	12	249	28.91	29.44	29.18	29.81	29.91	29.85	29.63		1.92	0.0654	-0.68
CLAU_2321	Adenylate cyclase	0.002	21	4	4	30	176	30.38	29.33	30.16	29.45	29.67	29.54	29.61		0.55	0.4491	0.40
CLAU_2200	NLP/P60 protein	0.000	20	6	5	56	345	30.04	30.02	30.00	29.20	28.71	29.09	29.60	+	2.62	0.0333	1.02
CLAU_2966	Flagellar assembly factor fliW	0.000	31	4	4	73	143	29.78	29.54	29.62	29.99	29.36	29.57	29.60		0.01	0.9852	0.01
CLAU_1176	Methyl-accepting chemotaxis sensory tr	0.001	14	9	9	35	570	30.06	29.69	28.90	29.15	29.50	30.00	29.60		0.00	1.0000	0.00
CLAU_2040	Nitrogen regulatory protein P-II	0.004	9	1	1	18	121	29.43	30.25	30.23	29.69	28.89	29.49	29.59		0.78	0.3174	0.61
CLAU_2946	UDP-N-acetylglucosamine transferase	0.003	30	8	8	28	354	29.65	29.73	29.09	29.36	29.54	29.63	29.59		0.03	0.9589	-0.02
CLAU_0331	Transcriptional regulator MarR family	0.004	26	3	3	21	145	29.86	29.79	30.03	29.38	28.88	29.38	29.59		1.70	0.0856	0.68
CLAU_1775	Microcompartments protein	0.001	36	5	5	34	182	29.74	29.51	30.08	29.11	29.42	29.65	29.58		0.78	0.3206	0.38
CLAU_0798	Diguanylate cyclase	0.001	23	5	5	42	247	29.86	29.62	28.93	28.91	29.54	29.61	29.58		0.12	0.8508	0.12
CLAU_0142	Cobalamin synthesis protein P47K	0.001	26	4	4	34	202	29.56	29.26	29.58	29.17	29.59	29.78	29.57		0.08	0.8983	-0.05
CLAU_3842	Sugar transporter	0.004	7	4	4	17	455	29.04	28.55	28.90	30.15	30.17	30.10	29.57	+	3.06	0.0237	-1.31
CLAU_1793	Transcriptional regulator TetR family	0.003	41	7	7	28	183	29.45	29.26	29.57	29.87	29.56	29.92	29.57		1.16	0.1765	-0.36
CLAU_2446	Amidohydrolase	0.001	24	7	7	36	389	29.56	29.39	29.55	29.38	29.76	29.78	29.56		0.42	0.5390	-0.14
CLAU_0393	DNA topoisomerase III	0.001	15	9	9	43	734	29.65	29.66	29.50	29.31	29.39	29.60	29.55		0.78	0.3197	0.17
CLAU_2418	HpcH/HpaI aldolase/citrate lyase family	0.005	16	5	5	21	290	29.50	29.59	29.40	29.51	29.61	29.73	29.55		0.65	0.3841	-0.12
CLAU_2633	HD family phosphohydrolase	0.001	30	10	10	41	352	29.90	29.60	29.49	29.68	29.10	28.99	29.55		0.76	0.3289	0.41
CLAU_3949	Uroporphyrinogen decarboxylase (URO-	0.007	17	4	4	15	289	28.05	28.55	29.60	30.12	29.81	29.49	29.55		1.03	0.2187	-1.07
CLAU_3048	Type IV pilus assembly pilZ	0.002	21	5	5	31	223	29.01	29.60	29.48	29.76	31.15	29.33	29.54		0.55	0.4486	-0.72
CLAU_2064	Ribosomal RNA small subunit methyltra	0.007	20	5	5	15	237	29.55	29.36	29.15	29.57	29.64	29.53	29.54		0.88	0.2724	-0.23
CLAU_2082	putative FAD-dependent oxidoreductase	0.001	14	7	7	38	463	29.20	29.29	29.61	29.46	30.57	30.40	29.54		0.99	0.2267	-0.78
CLAU_1023	Acetyltransferase	0.149	6	1	1	2	188	*23.07*	29.61	30.13	29.46	29.81	29.46	29.54		0.36	0.5929	-1.97
CLAU_0357	(NiFe) hydrogenase maturation protein	0.003	8	5	5	29	799	29.78	28.47	28.73	29.27	30.30	29.92	29.53		0.77	0.3237	-0.84
CLAU_2530	hypothetical protein	0.054	38	2	2	3	72	29.89	29.51	29.54	30.32	29.45	28.74	29.53		0.11	0.8639	0.14
CLAU_1402	CoA-substrate-specific enzyme activase	0.005	9	10	10	22	1431	31.25	30.15	29.57	28.01	28.67	29.45	29.51		1.18	0.1731	1.61
CLAU_1440	Aspartate carbamoyltransferase regulat	0.001	23	3	3	38	145	29.51	30.14	29.45	30.22	29.49	29.18	29.50		0.06	0.9177	0.07
CLAU_1253	Sporulation protein YunB	0.339	3	1	1	1	237	29.02	29.00	29.64	29.72	29.76	29.36	29.50		0.73	0.3392	-0.39
CLAU_2207	hypothetical protein	0.001	41	6	6	32	197	29.20	29.43	29.82	29.62	29.50	29.49	29.50		0.10	0.8716	-0.05
CLAU_3316	3'(2'),5'-bisphosphate nucleotidase	0.001	13	4	4	34	320	29.75	29.90	29.47	29.33	29.51	29.42	29.49		0.99	0.2288	0.29
CLAU_0291	Ferritin-like protein	0.000	45	9	9	52	187	29.49	29.35	29.66	28.38	29.81	29.49	29.49		0.24	0.7058	0.27
CLAU_3191	Pyruvate formate-lyase	0.000	19	12	12	47	854	29.64	29.15	29.33	30.30	29.99	29.26	29.49		0.63	0.3921	-0.48
CLAU_1918	RNA methyltransferase TrmH family gro	0.001	18	4	4	37	266	29.23	28.98	29.07	29.74	29.85	29.80	29.49	+	3.04	0.0237	-0.70
CLAU_0820	Peptidase S58 DmpA	0.018	12	3	3	10	319	29.64	29.44	29.52	29.50	29.45	29.47	29.49		0.43	0.5343	0.06
CLAU_2423	Methyl-accepting chemotaxis signaling (	0.001	7	2	1	35	382	29.45	29.96	29.78	29.50	29.04	29.01	29.48		1.18	0.1722	0.55
CLAU_0435	Molybdopterin-family oxidoreductase F/	0.003	15	4	4	29	272	29.54	29.06	29.32	29.41	29.77	29.78	29.48		0.87	0.2736	-0.35
CLAU_2370	UvrABC system protein C	0.018	5	3	3	7	622	29.83	30.08	29.80	29.04	28.27	29.15	29.48		1.69	0.0864	1.08
CLAU_3234	Transcriptional repressor NrdR	0.001	18	3	3	37	151	29.72	29.65	29.61	29.20	29.30	29.33	29.47	+	2.78	0.0283	0.38
CLAU_2364	UvrABC system protein B	0.002	19	12	12	31	660	29.28	30.19	29.22	29.92	29.65	29.04	29.47		0.02	0.9696	0.03
CLAU_2066	TRNA modification GTPase mnmE	0.004	17	6	6	21	460	29.49	29.77	29.43	28.89	29.54	28.85	29.46		0.89	0.2703	0.47
CLAU_0918	Transcriptional regulator ArsR family	0.005	43	3	3	20	97	28.69	29.03	29.03	30.06	29.96	29.89	29.46	+	2.98	0.0254	-1.05

CLAU_0227	Transcriptional regulator	0.000	14	13	13	50	1010	29.24	28.73	28.44	29.73	29.91	29.66	29.45		1.77	0.0799	-0.96
CLAU_2330	Molybdopterin-binding domain-containi	0.004	27	7	7	24	293	29.04	28.92	28.76	30.00	29.90	29.86	29.45	+	3.43	0.0182	-1.01
CLAU_3046	Flagellar biosynthetic protein flhF	0.004	25	8	8	27	397	28.79	29.02	28.51	29.93	30.12	29.88	29.45	+	2.73	0.0290	-1.20
CLAU_3425	putative permease	0.259	3	1	1	1	747	29.45	29.50	29.45	29.18	29.55	29.43	29.45		0.29	0.6508	0.08
CLAU_2504	Nitrogen regulatory protein P-II	0.021	5	1	1	10	130	28.36	29.10	29.09	30.04	29.91	29.78	29.44		1.84	0.0721	-1.06
CLAU_1262	Undecaprenyl-phosphate	0.066	11	2	2	2	217	29.74	29.49	28.94	28.56	29.39	29.52	29.44		0.24	0.7101	0.23
CLAU_2076	Hypothetical protein	0.007	43	4	4	15	87	29.51	29.40	29.68	29.28	29.44	29.43	29.44		0.69	0.3604	0.15
CLAU_2952	CheW protein	0.003	51	5	5	29	148	29.67	29.65	29.09	28.31	29.47	29.40	29.44		0.41	0.5459	0.41
CLAU_1002	Cell wall binding repeat 2-containing prc	0.000	6	3	1	109	1708	29.54	29.76	29.94	29.32	29.30	29.31	29.43		1.71	0.0854	0.44
CLAU_3727	NADP-dependent oxidoreductase doma	0.001	25	7	7	39	324	28.77	30.72	30.12	29.74	29.11	28.85	29.43		0.43	0.5341	0.64
CLAU_0606	Antibiotic biosynthesis monooxygenase-	0.004	29	2	2	24	95	29.41	29.22	29.85	29.40	29.42	29.46	29.42		0.13	0.8384	0.07
CLAU_2336	NAD synthase	0.004	25	6	6	26	265	29.37	29.38	29.03	29.51	29.66	29.44	29.41		0.98	0.2296	-0.28
CLAU_0275	5-formyltetrahydrofolate cyclo-ligase	0.006	9	2	2	16	181	29.11	29.17	28.14	29.72	29.85	29.65	29.41		1.29	0.1501	-0.93
CLAU_2277	Protein tyrosine phosphatase	0.004	30	4	4	25	152	29.19	29.21	29.41	29.63	29.39	29.45	29.40		1.03	0.2185	-0.22
CLAU_3038	Flagellar hook-basal body protein	0.001	31	6	6	32	347	29.23	29.42	29.59	28.83	29.52	29.37	29.40		0.30	0.6442	0.17
CLAU_2368	Peptidoglycan glycosyltransferase	0.000	23	8	8	52	475	29.38	29.14	29.41	29.49	29.72	29.33	29.40		0.65	0.3827	-0.20
CLAU_3168	Gluconate transporter	0.000	5	2	2	66	441	28.94	28.71	28.99	30.00	30.24	29.80	29.40	+	2.74	0.0285	-1.13
CLAU_0454	Glycine cleavage system glycine dehydr	0.000	21	8	8	57	485	29.85	29.88	29.63	29.11	28.87	28.91	29.37	+	2.79	0.0283	0.82
CLAU_1541	ABC-type transporter related protein	0.001	20	9	9	38	637	29.32	29.57	28.62	29.36	29.38	29.37	29.37		0.28	0.6619	-0.20
CLAU_1904	Ribosomal protein S12	0.004	37	3	3	22	125	29.80	29.37	28.60	28.86	30.09	29.33	29.35		0.12	0.8465	-0.17
CLAU_1922	PilT domain protein	0.004	6	2	2	22	375	29.02	29.80	29.21	30.21	29.06	29.47	29.34		0.23	0.7243	-0.24
CLAU_3945	Oligoendopeptidase F	0.001	16	9	9	49	595	29.35	29.31	29.17	29.34	29.45	29.23	29.33		0.31	0.6397	-0.06
CLAU_0082	Nitrate reductase	0.001	30	3	3	38	134	29.44	28.42	28.62	29.29	29.36	29.49	29.33		0.81	0.3052	-0.55
CLAU_0017	Ferric uptake regulator Fur family	0.013	28	4	4	10	152	27.15	28.66	27.63	29.98	29.98	30.00	29.32		2.09	0.0538	-2.17
CLAU_1219	Lytic transglycosylase	0.112	9	2	2	2	195	30.39	29.44	29.50	29.18	28.97	28.86	29.31		1.13	0.1857	0.77
CLAU_0092	PBP domain-containing protein	0.003	18	4	4	28	281	29.02	28.68	28.81	30.33	29.98	29.59	29.31		2.06	0.0556	-1.13
CLAU_2737	Rod shape-determining protein MreC	0.004	14	4	4	22	287	29.21	29.40	29.46	29.42	29.19	29.20	29.31		0.34	0.6128	0.09
CLAU_2614	Malate dehydrogenase	0.000	18	5	5	106	365	29.87	29.48	29.64	28.53	28.87	29.08	29.28		1.89	0.0687	0.84
CLAU_2672	Beta-lactamase domain protein	0.000	28	7	7	46	286	29.06	28.99	28.69	30.13	29.54	29.49	29.28		1.58	0.1031	-0.81
CLAU_0570	Ureidoglycolate lyase	0.004	21	5	5	21	300	29.10	28.88	28.71	29.43	29.58	29.54	29.27		2.16	0.0504	-0.62
CLAU_2098	Hypothetical protein	0.001	33	4	4	39	120	29.64	29.01	29.84	29.13	28.92	29.39	29.26		0.54	0.4512	0.35
CLAU_2429	HAD-superfamily hydrolase subfamily IA	0.004	14	3	3	22	216	28.76	28.85	29.42	29.18	29.34	29.55	29.26		0.68	0.3676	-0.35
CLAU_0663	Threonine aldolase	0.001	25	6	6	33	345	29.15	29.63	29.34	29.06	29.44	28.89	29.25		0.50	0.4869	0.24
CLAU_0026	Cold-shock DNA-binding domain proteir	0.004	17	1	1	24	65	29.81	29.95	29.99	28.26	28.42	28.68	29.25	+	3.40	0.0184	1.46
CLAU_2319	Heat shock protein DnaJ domain proteir	0.005	13	2	2	19	193	29.00	29.10	29.18	29.37	29.30	29.35	29.24		1.93	0.0651	-0.25
CLAU_2071	50S ribosomal protein L34	0.009	18	1	1	14	44	27.55	28.02	29.29	29.58	29.19	30.73	29.24		1.04	0.2144	-1.55
CLAU_2734	Septum formation protein Maf	0.003	10	2	2	29	192	29.18	29.24	29.23	29.44	29.27	29.24	29.24		0.70	0.3566	-0.10
CLAU_0599	putative secreted protein	0.000	33	3	3	71	82	29.60	29.74	28.86	28.58	28.99	29.46	29.23		0.45	0.5225	0.39
CLAU_3253	Phosphopantothenoylcysteine	0.005	21	6	6	19	396	28.37	28.76	29.29	29.16	29.46	29.38	29.23		0.87	0.2752	-0.53
CLAU_0046	Rubrerythrin	0.117	5	1	1	2	174	28.80	*21.26*	27.18	29.86	30.23	29.64	29.22		0.84	0.2882	-4.16
CLAU_0824	Cobalt ABC transporter ATPase subunit	0.000	28	6	6	49	278	30.17	29.30	30.40	28.53	28.65	29.13	29.22		1.44	0.1228	1.19
CLAU_0422	Sulfite reductase subunit B	0.001	35	8	8	34	263	29.65	30.39	29.81	28.77	28.43	28.60	29.21	+	2.27	0.0457	1.35
CLAU_2548	Glucose-1-phosphate thymidylyltransfer	0.003	23	6	6	29	292	28.79	29.26	28.84	29.16	29.78	29.46	29.21		1.01	0.2206	-0.50
CLAU_3433	hypothetical protein	0.050	3	2	2	4	966	29.27	29.34	29.61	28.88	28.72	29.15	29.21		1.40	0.1286	0.49
CLAU_1318	Single-strand binding protein	0.004	13	3	3	23	242	28.58	29.06	28.34	29.37	29.62	29.33	29.20		1.56	0.1062	-0.78
CLAU_2301	Helicase RecD/TraA family	0.028	7	4	4	5	745	28.72	29.17	29.10	29.22	29.42	29.33	29.20		1.01	0.2206	-0.33

CLAU_0125	Urea carboxylase	0.004	19	6	6	23	327	29.40	29.41	29.40	28.90	28.93	28.97	29.19	+	4.66	0.0046	0.47
CLAU_0163	Cysteinyl-tRNA ligase	0.002	10	5	5	31	471	29.32	29.31	29.12	29.22	29.14	28.95	29.18		0.64	0.3856	0.15
CLAU_0471	Amino acid permease	0.003	10	4	4	30	450	29.34	29.53	28.86	29.25	29.11	29.09	29.18		0.17	0.7869	0.09
CLAU_1097	Phage infection protein	0.156	4	2	2	2	719	29.16	29.22	29.32	28.78	29.20	29.01	29.18		0.84	0.2867	0.24
CLAU_0473	Methyl-accepting chemotaxis protein	0.013	5	2	1	10	511	29.14	29.09	28.89	29.27	29.21	29.32	29.18		1.28	0.1512	-0.23
CLAU_3193	Two component transcriptional regulatc	0.001	40	7	7	43	248	29.14	29.34	29.65	29.00	29.19	29.15	29.17		0.76	0.3283	0.26
CLAU_0260	Amino acid permease-associated region	0.007	5	2	2	15	487	29.33	29.00	28.96	29.48	29.77	28.95	29.17		0.50	0.4869	-0.30
CLAU_0097	MOSC domain-containing protein	0.005	29	3	3	19	143	28.97	29.14	29.28	29.18	29.67	29.04	29.16		0.32	0.6252	-0.17
CLAU_1072	GHMP kinase	0.004	16	5	5	26	285	29.52	29.51	29.15	29.15	29.16	29.09	29.16		0.99	0.2288	0.26
CLAU_0863	Hypothetical protein	0.004	31	3	3	21	59	28.92	29.34	29.20	29.10	29.20	28.98	29.15		0.16	0.7996	0.06
CLAU_0426	Carbamate kinase	0.004	20	5	5	18	316	29.16	28.79	29.14	29.10	29.28	29.39	29.15		0.70	0.3559	-0.23
CLAU_2970	Flagellar biosynthesis protein FliS	0.004	33	3	3	24	128	28.91	29.09	29.10	29.30	29.19	29.21	29.15		1.33	0.1426	-0.20
CLAU_3374	putative methyl-accepting chemotaxis p	0.002	12	7	7	33	677	29.20	28.92	29.08	29.18	29.11	29.25	29.15		0.55	0.4437	-0.11
CLAU_0610	Transcriptional regulator MerR family	0.013	22	4	4	10	131	28.87	28.53	29.13	29.16	29.18	29.18	29.15		0.88	0.2706	-0.33
CLAU_3066	UDP-N-acetylmuramoyl-tripeptide-D-ala	0.001	24	9	9	40	464	28.81	29.24	29.31	29.37	29.04	28.89	29.14		0.03	0.9589	0.02
CLAU_3363	putative dioxygenase	0.013	11	4	4	9	466	29.60	29.03	28.70	29.70	28.98	29.25	29.14		0.23	0.7169	-0.20
CLAU_3124	Hemolysin A	0.001	16	4	4	35	274	28.91	29.12	29.09	29.16	29.22	29.15	29.14		0.92	0.2530	-0.14
CLAU_1847	Hypothetical protein	0.004	12	3	3	25	252	28.98	29.07	28.94	29.39	29.20	29.40	29.14		1.94	0.0651	-0.33
CLAU_1477	Hemerythrin-like metal-binding protein	0.004	22	2	2	17	136	29.68	29.35	29.45	28.76	28.91	28.90	29.13	+	2.37	0.0407	0.64
CLAU_0758	Radical SAM domain protein	0.004	10	5	5	27	460	29.29	28.82	29.54	29.52	28.96	28.67	29.13		0.20	0.7586	0.17
CLAU_3324	Cell division FtsK/SpoIIIE	0.007	11	7	7	16	752	28.44	28.36	28.97	29.28	29.35	29.47	29.13		1.75	0.0806	-0.78
CLAU_2587	Hypothetical protein	0.003	18	6	6	29	392	28.78	28.89	29.35	28.11	30.31	30.14	29.12		0.28	0.6615	-0.51
CLAU_2192	DNA polymerase III gamma subunit dor	0.006	17	6	6	16	314	28.83	29.07	29.07	29.34	29.31	29.13	29.10		1.23	0.1614	-0.27
CLAU_3003	Glycosyl transferase family 2	0.017	3	1	1	13	349	28.93	27.47	30.85	28.71	29.29	29.27	29.10		0.00	0.9990	-0.01
CLAU_3176	Histidinol dehydrogenase	0.007	19	7	7	16	434	28.54	28.79	29.40	29.68	28.27	30.07	29.10		0.29	0.6575	-0.43
CLAU_2467	Methionine import ATP-binding protein	0.003	22	6	6	31	318	29.53	29.70	29.49	28.09	28.52	28.69	29.09	+	2.41	0.0393	1.14
CLAU_1415	Di-trans,poly-cis-decaprenylcistransfera	0.003	22	4	4	28	214	29.07	29.15	28.84	28.94	29.11	29.13	29.09		0.13	0.8371	-0.04
CLAU_1785	Microcompartments protein	0.007	13	1	1	15	98	29.44	29.39	29.56	28.51	28.31	28.79	29.09	+	2.48	0.0381	0.93
CLAU_1183	Lipoyltransferase and lipoate-protein lig	0.004	19	6	6	25	327	29.18	28.88	28.81	29.43	29.13	29.04	29.09		0.68	0.3676	-0.24
CLAU_1417	hypothetical protein	0.004	26	2	2	18	66	29.66	28.98	29.35	27.16	28.72	29.17	29.08		0.70	0.3580	0.98
CLAU_3417	Regulatory protein MerR	0.009	45	6	6	12	143	29.28	29.52	29.74	28.87	28.79	28.51	29.08		2.00	0.0606	0.79
CLAU_3814	putative Fe-S cluster protein	0.001	16	5	5	34	344	28.81	29.09	29.06	29.51	29.25	28.98	29.08		0.67	0.3730	-0.26
CLAU_1335	Amidohydrolase	0.001	18	4	4	32	378	28.87	28.66	28.79	29.26	29.42	29.41	29.07	+	2.74	0.0285	-0.59
CLAU_2637	Ribonuclease H	0.013	21	5	5	9	252	28.76	28.68	30.14	29.67	27.75	29.37	29.07		0.13	0.8451	0.26
CLAU_2258	4-diphosphocytidyl-2-C-methyl-D-erythr	0.003	19	4	4	29	280	29.11	28.19	29.06	28.97	29.07	29.10	29.07		0.36	0.5951	-0.26
CLAU_1067	Hypothetical protein	0.004	13	2	2	18	120	29.11	29.01	29.26	29.01	29.19	29.02	29.07		0.22	0.7262	0.05
CLAU_0165	HutD-family protein	0.006	25	4	4	16	210	29.11	29.03	29.26	28.92	29.01	29.09	29.06		0.69	0.3604	0.13
CLAU_2395	Glycosyl transferase family 2	0.003	22	5	5	29	312	29.06	29.04	29.05	28.01	29.14	29.30	29.06		0.22	0.7251	0.23
CLAU_1250	Nucleotidyltransferase	0.007	9	3	3	14	298	29.59	29.56	29.42	28.66	28.62	28.69	29.06	+	3.99	0.0097	0.87
CLAU_3175	ATP phosphoribosyltransferase	0.007	16	4	4	13	219	28.73	28.08	29.44	28.91	29.33	29.20	29.06		0.41	0.5499	-0.40
CLAU_3299	DNA topoisomerase I	0.001	15	9	9	33	702	28.36	28.62	28.90	29.45	29.68	29.19	29.05		1.74	0.0817	-0.81
CLAU_2798	Histidine kinase	0.275	2	1	1	1	482	28.75	29.20	29.26	29.05	28.39	29.04	29.05		0.38	0.5799	0.24
CLAU_1869	Cobalt ABC transporter ATPase subunit	0.004	19	6	6	22	284	28.92	28.79	29.01	29.06	29.16	29.14	29.04		1.40	0.1286	-0.21
CLAU_2000	3-oxoacyl-(acyl-carrier-protein) synthase	0.012	8	2	2	10	326	28.77	28.51	29.02	29.05	29.10	29.24	29.04		1.08	0.2015	-0.36
CLAU_3628	Transcriptional regulator MarR family	0.013	38	5	5	12	148	29.34	29.08	29.39	28.87	28.92	28.97	29.03		1.60	0.1005	0.35
CLAU_2497	Hypothetical protein	0.004	45	3	3	21	86	28.22	27.91	28.66	29.66	29.75	29.38	29.02	+	2.26	0.0459	-1.33

CLAU_1268	Transferase hexapeptide repeat-contain	0.001	14	3	3	36	249	28.94	29.33	28.81	27.85	29.09	29.44	29.02		0.17	0.7850	0.23
CLAU_1043	Transcriptional regulator CdaR family	0.003	17	7	7	30	521	29.30	29.30	29.13	28.66	28.90	28.68	29.02	+	2.19	0.0495	0.50
CLAU_0372	ClC-type chloride channel EriC	0.112	3	1	1	2	435	29.09	28.93	28.86	29.24	29.22	28.79	29.01		0.31	0.6377	-0.12
CLAU_1329	Uroporphyrinogen decarboxylase (URO-	0.002	28	8	8	32	368	29.01	29.00	28.98	28.85	29.23	29.18	29.01		0.31	0.6407	-0.09
CLAU_3606	putative Mg(2+) transporter	0.297	2	1	1	1	408	26.55	27.45	28.16	29.95	29.99	29.85	29.01	+	2.26	0.0459	-2.54
CLAU_1208	Peptidase M20	0.004	9	4	4	24	438	28.94	28.86	28.63	29.08	29.06	29.23	29.00		1.36	0.1356	-0.31
CLAU_3036	Flagellar hook capping protein	0.004	21	4	4	22	242	29.27	29.15	29.37	28.82	28.47	28.83	28.99		1.84	0.0721	0.56
CLAU_2729	Homoserine dehydrogenase	0.001	23	7	7	40	414	29.09	29.52	29.39	28.60	28.88	28.05	28.99		1.40	0.1292	0.82
CLAU_1940	Hypoxanthine phosphoribosyltransferas	0.004	20	4	4	27	181	29.02	28.94	29.03	28.79	29.18	28.77	28.98		0.24	0.7101	0.08
CLAU_3640	Response regulator	0.150	5	1	1	2	239	29.80	27.76	27.44	29.21	28.83	29.12	28.98		0.41	0.5499	-0.72
CLAU_1446	Methionine aminopeptidase type I	0.001	31	5	5	32	251	28.83	28.82	29.09	28.84	29.31	29.11	28.97		0.46	0.5125	-0.17
CLAU_2880	Fumarate reductase	0.004	14	7	7	20	486	28.91	29.02	27.56	28.64	29.48	29.58	28.97		0.59	0.4185	-0.74
CLAU_3824	Efflux transporter RND family MFP subu	0.001	31	9	9	41	361	28.98	29.50	28.60	28.73	29.45	28.94	28.96		0.01	0.9828	-0.01
CLAU_3052	Flagellar hook-basal body protein	0.002	39	6	6	31	255	27.72	28.59	28.47	29.31	29.39	29.32	28.95		1.77	0.0794	-1.08
CLAU_0228	Glutamate	0.001	30	7	7	36	298	29.58	30.00	29.46	27.73	27.85	28.42	28.94	+	2.48	0.0381	1.68
CLAU_2054	hypothetical protein	0.001	34	2	2	35	70	28.55	28.90	29.31	28.81	29.06	28.97	28.94		0.04	0.9510	-0.03
CLAU_1590	MOSC domain-containing protein	0.004	20	3	3	17	138	28.63	28.90	29.16	28.60	28.96	29.09	28.93		0.02	0.9710	0.01
CLAU_2452	Glycosyl transferase family 2	0.004	10	3	3	21	316	29.43	29.09	27.94	28.76	28.77	29.22	28.93		0.07	0.9072	-0.10
CLAU_2972	Flagellar protein FliT	0.004	29	3	3	25	114	28.89	28.80	29.39	28.70	28.94	29.03	28.92		0.26	0.6838	0.14
CLAU_3594	Peptidase M29 aminopeptidase	0.004	15	5	5	20	409	29.59	28.30	28.40	28.83	29.21	29.00	28.92		0.23	0.7222	-0.25
CLAU_2068	Membrane protein insertase YidC/Oxa1	0.013	6	1	1	9	218	28.93	29.16	28.91	28.64	28.92	28.90	28.92		0.68	0.3673	0.18
CLAU_2715	Molybdopterin-guanine dinucleotide bic	0.002	24	4	4	31	189	28.71	28.55	28.84	28.95	29.07	29.03	28.90		1.60	0.1005	-0.32
CLAU_1636	Hypothetical protein	0.004	26	3	3	18	136	29.15	29.05	28.56	28.74	28.50	29.10	28.90		0.22	0.7344	0.14
CLAU_0934	Hypothetical protein	0.014	11	3	3	8	341	28.84	29.36	28.95	27.95	27.98	29.18	28.90		0.71	0.3493	0.68
CLAU_1397	ABC-type transporter related protein	0.006	14	3	3	16	285	28.60	28.73	28.95	29.13	28.97	28.83	28.89		0.74	0.3359	-0.22
CLAU_2276	Threonylcarbamoyl-AMP synthase	0.004	26	7	7	26	350	28.91	28.59	28.73	28.99	28.98	28.86	28.89		0.92	0.2545	-0.20
CLAU_2375	putative signal transduction protein witl	0.004	19	6	6	24	434	28.91	28.95	29.01	28.49	28.67	28.85	28.88		1.25	0.1589	0.29
CLAU_1241	S-adenosylmethionine:tRNA	0.003	17	5	5	29	341	29.27	30.07	29.17	28.26	28.50	28.58	28.88		1.61	0.1001	1.06
CLAU_1518	ATP-dependent DNA helicase PcrA	0.004	14	10	10	27	746	28.25	28.58	28.47	29.15	29.26	29.21	28.87	+	2.79	0.0283	-0.77
CLAU_3340	Serine-type D-Ala-D-Ala carboxypeptida	0.001	11	5	5	35	413	28.84	28.82	29.07	28.35	28.88	28.97	28.86		0.35	0.6030	0.18
CLAU_3615	N-(5'-phosphoribosyl)anthranilate isome	0.012	27	4	4	10	209	27.20	23.67	28.78	28.93	29.36	29.32	28.86		0.81	0.3037	-2.65
CLAU_3524	Xanthine phosphoribosyltransferase	0.003	19	3	3	28	190	28.81	28.90	28.68	28.66	29.24	29.20	28.86		0.53	0.4631	-0.24
CLAU_2673	1-acyl-sn-glycerol-3-phosphate acyltran:	0.009	17	4	4	11	241	28.65	28.90	28.68	28.81	29.27	29.03	28.86		0.88	0.2706	-0.29
CLAU_0197	2,4-dienoyl-CoA reductase (NADPH)	0.000	19	10	10	51	673	28.34	28.79	28.90	28.63	28.93	28.91	28.85		0.30	0.6437	-0.15
CLAU_2860	Xanthine/uracil/vitamin C permease	0.004	9	3	3	23	478	28.81	28.76	28.29	28.88	29.13	29.02	28.85		1.01	0.2206	-0.39
CLAU_0220	Formimidoylglutamase	0.005	11	3	3	22	339	29.46	28.70	26.95	29.52	28.99	28.28	28.85		0.27	0.6742	-0.56
CLAU_0517	ATP-dependent Clp protease ATP-bindir	0.000	16	11	11	56	746	28.68	28.61	28.82	29.04	28.94	28.85	28.84		1.36	0.1370	-0.24
CLAU_1888	Ribosomal protein L24	0.000	16	2	2	129	105	29.24	29.20	28.49	28.06	28.92	28.73	28.83		0.50	0.4867	0.41
CLAU_2699	Transferase hexapeptide repeat-contain	0.004	19	3	3	24	168	28.41	28.48	28.43	29.55	29.24	29.17	28.83	+	2.75	0.0285	-0.88
CLAU_0062	Glyoxalase-like protein	0.007	41	5	5	15	155	29.27	28.89	29.24	28.76	28.74	28.65	28.83		1.52	0.1114	0.42
CLAU_2419	Citrate lyase alpha subunit	0.007	2	1	1	14	513	28.83	28.83	28.87	28.82	28.13	28.77	28.83		0.53	0.4567	0.27
CLAU_1186	Iron-sulfur-dependent L-serine dehydrat	0.004	32	7	7	22	290	28.47	28.21	28.86	28.98	28.91	28.76	28.81		0.86	0.2801	-0.37
CLAU_1956	Integral membrane sensor signal transd	0.005	17	6	6	20	471	28.88	28.74	28.69	28.63	29.52	29.40	28.81		0.66	0.3761	-0.41
CLAU_1324	FolC bifunctional protein	0.000	22	9	9	51	448	28.63	28.09	28.92	28.78	29.07	28.81	28.80		0.58	0.4246	-0.34
CLAU_0566	Aldo-keto reductase family NADP-deper	0.004	12	4	4	29	378	29.26	29.35	29.06	28.46	28.24	28.53	28.80	+	2.57	0.0344	0.81
CLAU_3236	Two component transcriptional regulatc	0.005	17	4	4	24	231	29.15	28.81	28.71	28.53	28.78	28.82	28.80		0.49	0.4943	0.18

CLAU_1254	Penicillin-binding protein transpeptidase	0.002	23	11	11	31	783	29.06	28.71	28.62	28.87	29.03	28.32	28.79		0.08	0.8986	0.06
CLAU_3754	Cell division protein FtsA	0.004	13	7	7	26	701	28.83	28.81	28.75	28.66	30.59	28.72	28.78		0.34	0.6082	-0.53
CLAU_1858	GlmL	0.005	17	6	6	17	470	28.10	27.61	27.68	29.52	29.46	29.63	28.78	+	3.38	0.0186	-1.74
CLAU_1841	YbbR family protein	0.002	17	6	6	31	418	28.91	29.60	28.37	28.64	28.87	28.65	28.76		0.26	0.6830	0.24
CLAU_3303	Uridylate kinase	0.003	14	4	4	31	237	28.58	28.74	28.46	29.06	29.12	28.77	28.76		1.35	0.1392	-0.39
CLAU_2455	Glutamyl-tRNA reductase	0.004	13	5	5	18	398	28.32	28.74	27.69	29.15	28.77	28.79	28.76		0.93	0.2517	-0.65
CLAU_2810	Heat-inducible transcription repressor H	0.004	38	9	9	23	346	29.80	29.89	29.41	27.36	27.84	28.07	28.74	+	2.79	0.0283	1.94
CLAU_3516	putative NUDIX hydrolase	0.004	17	5	5	18	299	28.58	28.51	28.73	28.75	29.05	28.94	28.74		1.32	0.1451	-0.31
CLAU_1928	UvrB/UvrC protein	0.064	11	2	2	2	182	27.97	28.76	28.39	28.72	29.17	29.02	28.74		1.06	0.2080	-0.60
CLAU_1236	Radical SAM protein	0.005	11	5	5	16	455	28.80	28.53	28.74	28.90	28.74	28.70	28.74		0.37	0.5866	-0.09
CLAU_3208	MgsA AAA-ATPase domain-containing p	0.004	27	9	9	19	438	28.65	28.69	28.67	28.78	28.96	28.95	28.74		1.72	0.0839	-0.23
CLAU_0705	ABC-type transporter / Fe(3+)-transport	0.005	27	5	5	21	204	27.54	28.66	28.50	28.81	29.25	28.98	28.74		0.98	0.2296	-0.78
CLAU_2643	CobB/CobQ domain protein glutamine	0.007	21	4	4	13	244	28.42	29.15	28.76	27.63	28.70	29.00	28.73		0.29	0.6562	0.33
CLAU_1877	Translation initiation factor IF-1	0.018	17	1	1	6	72	28.38	29.90	30.14	28.82	28.18	28.62	28.72		0.73	0.3392	0.93
CLAU_2012	Fumarate hydratase	0.005	19	6	6	20	465	28.60	27.96	28.55	29.01	28.93	28.83	28.72		1.23	0.1614	-0.55
CLAU_2883	Transcriptional regulator MerR family	0.004	18	4	4	27	279	28.58	28.71	28.70	28.80	28.68	28.87	28.71		0.80	0.3092	-0.12
CLAU_2549	DTDP-4-dehydrorhamnose reductase	0.012	19	5	5	11	295	28.46	28.50	28.02	28.97	29.15	28.91	28.71		1.80	0.0765	-0.68
CLAU_2568	DTDP-4-dehydrorhamnose reductase	0.013	14	4	4	10	291	28.46	28.50	28.02	28.97	29.15	28.91	28.71		1.80	0.0765	-0.68
CLAU_1941	TRNA(Ile)-lysidine synthase	0.002	24	10	10	31	468	27.97	28.13	28.71	29.23	28.67	28.75	28.69		1.01	0.2213	-0.61
CLAU_1036	Peptidase S51 dipeptidase E	0.004	30	5	5	25	220	28.40	28.12	28.80	28.65	28.73	28.76	28.69		0.61	0.4040	-0.27
CLAU_3025	hypothetical protein	0.009	11	1	1	11	112	28.06	28.34	29.07	28.82	28.66	28.72	28.69		0.33	0.6205	-0.24
CLAU_3804	DNA alkylation repair enzyme	0.009	20	4	4	12	233	28.86	28.35	27.69	28.73	29.00	28.64	28.69		0.62	0.4001	-0.49
CLAU_1810	Transcriptional regulator MerR family	0.005	12	2	2	21	253	28.70	28.55	28.62	28.66	29.10	28.87	28.68		0.88	0.2726	-0.25
CLAU_2982	Carbon storage regulator	0.013	22	1	1	8	58	29.08	28.70	28.97	28.41	28.44	28.66	28.68		1.40	0.1286	0.41
CLAU_0958	putative oxidoreductase iron-sulfur bind	0.001	4	1	1	43	161	28.21	28.32	29.08	29.20	28.82	28.53	28.68		0.39	0.5619	-0.31
CLAU_0436	Molybdopterin-family oxidoreductase 2	0.001	4	1	1	43	155	28.21	28.32	29.08	29.20	28.82	28.53	28.68		0.39	0.5619	-0.31
CLAU_0909	SEC-C motif domain protein	0.004	12	4	4	24	403	29.20	29.39	29.28	27.75	27.47	28.12	28.66	+	2.81	0.0283	1.51
CLAU_3040	Flagellar basal body-associated protein f	0.000	17	3	3	48	179	28.39	29.24	28.87	28.91	28.44	28.42	28.66		0.34	0.6086	0.24
CLAU_3000	putative nucleotidyltransferase	0.006	27	4	4	17	100	28.40	28.45	28.89	28.84	28.47	28.84	28.66		0.28	0.6690	-0.14
CLAU_1474	putative secreted protein	0.013	8	2	2	9	315	28.84	28.32	28.54	28.80	28.64	28.67	28.66		0.36	0.5952	-0.14
CLAU_2292	ATP synthase epsilon chain	0.004	30	3	3	20	132	28.10	29.42	29.43	29.15	28.15	28.00	28.65		0.41	0.5499	0.55
CLAU_1252	Hypoxanthine phosphoribosyltransferas	0.007	12	2	2	15	173	28.54	29.00	29.22	28.68	28.46	28.62	28.65		0.72	0.3444	0.33
CLAU_2334	hypothetical protein	0.003	17	5	5	28	399	28.19	28.60	28.31	29.37	28.93	28.69	28.65		1.27	0.1546	-0.63
CLAU_1057	Hypothetical protein	0.002	15	5	5	33	301	28.30	27.43	28.41	28.88	29.16	28.93	28.65		1.37	0.1347	-0.94
CLAU_3803	Methylated-DNA-protein-cysteine	0.004	44	6	6	25	156	28.43	27.97	28.29	28.85	29.05	28.97	28.64		2.10	0.0537	-0.73
CLAU_3943	Gamma-glutamyltransferase	0.007	12	5	5	16	535	29.29	27.40	30.14	28.43	28.55	28.73	28.64		0.17	0.7853	0.37
CLAU_3494	Transcriptional regulator LclR family	0.033	14	3	3	5	260	28.32	29.19	29.27	28.54	28.60	28.66	28.63		0.46	0.5126	0.33
CLAU_0717	hypothetical protein	0.004	11	4	4	24	389	28.59	28.65	28.45	28.76	28.69	28.59	28.62		0.69	0.3614	-0.12
CLAU_2836	DNA primase	0.004	12	7	7	18	590	29.12	28.59	28.70	28.44	28.64	28.26	28.62		0.85	0.2857	0.36
CLAU_1870	Phosphonate-transporting ATPase	0.004	19	5	5	20	281	28.46	28.28	28.41	28.76	29.23	28.87	28.61		1.70	0.0854	-0.57
CLAU_3257	Peptidase membrane zinc metallopeptic	0.013	9	2	2	9	226	28.16	28.15	27.96	29.06	29.21	29.07	28.61	+	3.64	0.0140	-1.02
CLAU_1718	S-adenosyl-L-methionine dependent tRN	0.004	44	7	7	24	155	28.42	27.99	28.47	28.74	29.28	28.95	28.61		1.48	0.1191	-0.70
CLAU_1249	Transcriptional regulator GntR family	0.239	2	1	1	1	473	29.31	28.65	28.70	28.50	28.51	28.56	28.61		0.79	0.3151	0.36
CLAU_0198	Methionine gamma-lyase	0.004	10	4	4	19	425	28.40	28.52	28.61	28.65	28.75	28.59	28.60		0.94	0.2479	-0.15
CLAU_3475	putative protein UPF0597 family	0.044	4	2	2	4	435	28.55	28.28	28.51	28.65	29.01	28.82	28.60		1.33	0.1426	-0.38
CLAU_3609	NADPH-dependent FMN reductase	0.005	9	2	2	16	233	28.92	28.55	28.58	28.61	28.51	28.74	28.60		0.18	0.7830	0.06

CLAU_0433	N-acyl-D-amino-acid deacylase	0.055	1	1	1	4	534	25.12	22.79	32.73	32.68	*20.34*	32.04	28.58		0.11	0.8678	-1.47
CLAU_0201	DNA mismatch repair protein mutL	0.004	15	8	8	23	609	28.30	28.31	28.54	28.61	28.66	28.64	28.58		1.47	0.1193	-0.25
CLAU_2678	Homocysteine Methyltransferase	0.004	9	5	5	19	799	28.59	28.66	28.65	28.54	28.38	28.44	28.57		1.60	0.1005	0.18
CLAU_3775	putative amidotransferase	0.263	11	1	1	1	194	28.84	28.61	*22.35*	28.75	28.49	28.47	28.55		0.39	0.5658	-1.97
CLAU_1538	DNA (apurinic or apyrimidinic site) lyase	0.001	27	8	8	47	302	28.63	28.41	27.86	28.70	29.10	28.43	28.53		0.67	0.3718	-0.44
CLAU_3584	Transcriptional regulator ArsR family	0.009	13	1	1	12	102	28.18	28.12	28.34	28.79	29.02	28.72	28.53	+	2.31	0.0430	-0.63
CLAU_0233	SNF2 helicase associated domain protei	0.023	3	2	2	8	1080	28.16	27.41	28.44	28.67	28.60	28.74	28.52		1.01	0.2217	-0.67
CLAU_0900	Transcriptional regulator IclR family	0.004	26	5	5	25	261	27.82	28.10	28.29	28.76	28.75	28.81	28.52		2.16	0.0504	-0.70
CLAU_3194	Histidine kinase	0.004	15	4	4	25	403	28.59	28.73	28.21	28.73	28.44	27.35	28.52		0.31	0.6409	0.34
CLAU_0240	Regulatory protein GntR with HTH doma	0.004	17	3	3	22	232	28.27	28.46	28.35	28.57	28.60	28.62	28.52		1.85	0.0721	-0.24
CLAU_1985	(FeFe)-hydrogenase system regulator	0.004	65	3	3	20	81	26.65	28.44	28.59	28.91	28.86	28.08	28.52		0.46	0.5128	-0.72
CLAU_2967	Carbon storage regulator	0.033	15	1	1	4	71	28.27	28.40	28.14	28.65	28.61	28.73	28.51		2.04	0.0567	-0.39
CLAU_3205	Propanediol utilization protein	0.004	29	4	4	21	216	29.89	29.18	28.63	28.14	28.14	28.36	28.50		1.29	0.1512	1.02
CLAU_1860	Transcriptional regulator GntR family	0.004	22	6	6	17	325	28.67	28.45	28.54	28.23	28.55	28.39	28.50		0.66	0.3757	0.16
CLAU_1483	4-hydroxybenzoyl-CoA thioesterase	0.007	22	3	3	15	137	28.27	28.21	28.69	28.55	28.52	28.47	28.50		0.33	0.6163	-0.12
CLAU_2749	Ribonuclease	0.004	17	7	7	24	479	28.82	29.53	24.59	28.16	29.07	28.03	28.49		0.19	0.7683	-0.77
CLAU_1731	Ig domain protein	0.004	12	3	3	19	424	27.81	28.59	28.15	29.20	29.13	28.38	28.49		0.97	0.2319	-0.72
CLAU_2936	Peptidase M16 domain protein	0.054	5	2	2	3	410	27.95	*20.77*	28.09	28.88	29.39	29.35	28.49		0.68	0.3676	-3.60
CLAU_3498	Major facilitator superfamily MFS_1	0.007	12	4	4	15	429	28.50	27.52	28.15	28.46	29.32	28.53	28.48		0.83	0.2920	-0.71
CLAU_2039	Methyl-accepting chemotaxis sensory tr	0.013	4	1	0	8	278	28.93	28.74	28.55	28.19	28.40	28.31	28.48		1.61	0.1005	0.44
CLAU_3008	hypothetical protein	0.101	19	2	2	3	118	27.80	27.04	28.99	28.22	28.72	28.90	28.47		0.48	0.4964	-0.67
CLAU_2938	Arginine repressor	0.004	14	2	2	25	151	28.26	27.40	28.49	28.43	28.80	28.63	28.46		0.75	0.3322	-0.57
CLAU_1163	Universal stress protein	0.013	7	1	1	10	137	28.17	26.27	*21.10*	29.18	29.11	28.73	28.45		0.84	0.2892	-3.83
CLAU_3178	Imidazole glycerol phosphate synthase s	0.004	18	3	3	17	202	29.51	28.57	28.22	26.73	29.29	28.33	28.45		0.32	0.6323	0.65
CLAU_3464	Transcriptional regulator XRE family	0.002	55	5	5	31	94	28.42	28.67	28.93	28.44	28.28	28.45	28.45		0.84	0.2900	0.28
CLAU_3270	Phosphopantetheine adenylyltransferas	0.005	31	4	4	16	164	28.65	28.05	27.58	28.28	28.60	28.92	28.44		0.63	0.3917	-0.51
CLAU_1191	Transcriptional regulator LysR family	0.013	15	3	2	9	290	28.49	28.30	28.45	28.07	28.42	28.54	28.44		0.17	0.7850	0.07
CLAU_3591	Peptidoglycan binding domain-containir	0.004	7	3	3	20	470	28.22	28.48	28.38	28.47	28.54	28.22	28.43		0.15	0.8133	-0.05
CLAU_1360	CRISPR-associated protein	0.004	12	3	3	22	236	28.59	28.53	28.32	28.38	28.46	28.35	28.42		0.40	0.5587	0.08
CLAU_2592	Von Willebrand factor type A	0.004	13	6	6	25	419	28.27	28.28	27.97	28.55	28.68	28.83	28.42		1.77	0.0794	-0.51
CLAU_3416	Cupin 2 conserved barrel domain protei	0.004	39	3	3	21	114	28.45	27.87	28.42	28.09	28.56	28.40	28.41		0.17	0.7930	-0.10
CLAU_1368	Nitroreductase	0.002	19	3	3	31	180	28.49	28.44	28.34	28.47	28.36	28.37	28.41		0.15	0.8109	0.02
CLAU_1708	Ribonuclease R	0.007	6	5	5	14	712	27.85	28.14	28.32	28.49	28.69	28.83	28.41		1.55	0.1082	-0.57
CLAU_2828	Transcriptional regulator containing CBS	0.013	11	2	2	9	214	29.23	29.00	28.15	28.43	28.38	28.36	28.41		0.54	0.4527	0.40
CLAU_0030	Alcohol dehydrogenase	0.055	6	2	2	4	378	28.18	25.69	28.37	28.52	28.46	28.42	28.40		0.54	0.4546	-1.05
CLAU_3963	Nucleoside transporter	0.004	5	2	2	22	461	28.77	28.69	27.40	28.23	28.41	28.37	28.39		0.04	0.9525	-0.05
CLAU_2871	Phosphoribosylglycinamide formyltransf	0.004	24	3	3	21	204	28.36	28.38	27.18	28.02	28.96	28.55	28.37		0.49	0.4948	-0.54
CLAU_1246	Hypothetical protein	0.005	14	3	3	19	272	28.90	28.52	28.78	28.21	28.22	28.09	28.37		2.02	0.0588	0.56
CLAU_0638	hypothetical protein	0.045	9	1	1	3	75	28.74	28.49	28.06	28.25	28.18	28.69	28.37		0.08	0.8990	0.06
CLAU_2019	Transcriptional regulator LysR family	0.017	16	3	2	8	287	28.35	28.08	28.51	28.13	28.38	28.38	28.37		0.04	0.9525	0.02
CLAU_3321	Processing peptidase	0.019	4	2	2	6	432	28.07	28.73	27.90	29.94	28.37	28.36	28.37		0.49	0.4907	-0.66
CLAU_2238	Alkaline phosphatase / SNARE associate	0.008	8	2	2	12	202	28.23	28.33	28.32	28.40	28.67	28.67	28.37		1.40	0.1286	-0.29
CLAU_3950	Uroporphyrinogen decarboxylase (URO-	0.002	15	3	3	30	356	27.61	27.79	28.35	28.57	28.37	28.84	28.36		1.22	0.1637	-0.68
CLAU_2710	Hypothetical protein	0.017	5	2	2	8	481	28.97	28.50	27.88	28.21	28.19	28.54	28.36		0.15	0.8131	0.14
CLAU_3326	CDP-diacylglycerol/glycerol-3-phosphat	0.023	8	2	2	8	195	29.03	29.37	26.61	26.73	28.15	28.55	28.35		0.20	0.7586	0.53
CLAU_0202	DNA mismatch repair protein mutS	0.004	10	8	8	17	891	28.62	28.07	28.69	27.43	27.54	28.78	28.35		0.50	0.4853	0.54

CLAU_2823	Heat-shock protein	0.016	20	3	3	7	164	29.03	28.78	28.53	27.92	27.90	28.13	28.33		2.10	0.0537	0.80
CLAU_1076	Cobalamin synthesis protein cbiG	0.019	12	4	4	8	350	28.44	28.52	28.35	26.65	28.28	28.30	28.33		0.56	0.4395	0.69
CLAU_2193	PSP1 domain protein	0.001	10	3	3	41	301	28.16	28.43	28.39	28.31	28.33	28.11	28.32		0.28	0.6642	0.08
CLAU_1152	Hypothetical protein	0.005	25	5	5	27	218	28.43	28.09	27.94	28.21	28.71	28.72	28.32		0.82	0.2986	-0.39
CLAU_3357	Transcriptional regulator IclR family	0.088	11	2	2	3	263	26.48	27.65	27.42	29.36	29.57	28.99	28.32	+	2.23	0.0474	-2.12
CLAU_3897	Hypothetical protein	0.155	4	1	1	2	245	27.49	26.36	28.44	28.79	28.20	28.46	28.32		0.78	0.3200	-1.05
CLAU_0969	Response regulator receiver protein	0.004	36	4	4	19	120	29.47	28.11	29.16	28.19	28.08	28.44	28.32		0.73	0.3419	0.68
CLAU_0428	Selenium metabolism protein SsnA	0.016	8	3	3	7	442	27.56	27.09	28.27	28.63	28.36	28.62	28.32		1.19	0.1711	-0.90
CLAU_1225	Hypothetical protein	0.004	32	6	6	26	222	28.36	28.33	28.27	27.97	28.66	28.28	28.31		0.03	0.9628	0.02
CLAU_1285	Hypothetical protein	0.054	6	2	2	3	327	25.52	22.00	30.53	29.11	28.13	28.48	28.31		0.44	0.5282	-2.56
CLAU_1093	Cobalt permease	0.005	9	3	3	20	268	28.03	28.36	28.14	28.32	28.78	28.28	28.30		0.69	0.3616	-0.28
CLAU_2875	Hypothetical protein	0.017	9	3	3	7	350	27.99	28.48	28.45	28.34	28.17	28.26	28.30		0.11	0.8644	0.05
CLAU_2366	FHA domain-containing protein	0.004	24	3	3	21	146	26.88	27.50	28.15	28.61	28.61	28.44	28.30		1.32	0.1451	-1.04
CLAU_2519	Outer membrane efflux protein	0.017	2	1	1	7	380	27.56	27.65	27.27	29.31	28.93	29.19	28.29	+	3.30	0.0191	-1.65
CLAU_2644	UDP-N-acetlymuramyl peptide synthase	0.003	16	6	6	27	452	28.11	27.50	27.95	28.47	28.46	28.50	28.29		1.57	0.1052	-0.62
CLAU_2803	DNA polymerase III delta subunit	0.276	6	1	1	1	341	27.34	29.03	28.81	28.27	28.30	27.44	28.29		0.26	0.6867	0.39
CLAU_0644	Glycyl-radical enzyme pyruvate formate	0.140	1	1	1	2	784	28.73	28.51	*21.44*	26.22	28.19	28.36	28.28		0.21	0.7390	-1.36
CLAU_3014	Cell wall binding repeat 2-containing pro	0.000	16	10	10	64	749	26.48	26.36	27.78	29.53	29.43	28.76	28.27		2.00	0.0606	-2.37
CLAU_3125	NAD kinase	0.007	13	3	3	13	285	27.71	28.33	28.20	28.43	28.36	28.21	28.27		0.56	0.4376	-0.25
CLAU_3218	Ferric uptake regulator Fur family	0.005	25	3	3	16	158	28.18	28.13	28.25	28.26	28.43	28.35	28.26		1.25	0.1588	-0.16
CLAU_1017	Citrate lyase acyl carrier protein	0.009	19	2	2	12	98	28.30	28.44	28.95	28.21	28.08	28.09	28.26		1.02	0.2206	0.44
CLAU_0580	Citrate lyase gamma subunit	0.009	19	2	2	12	98	28.30	28.44	28.95	28.21	28.08	28.09	28.26		1.02	0.2206	0.44
CLAU_2167	Cellulase	0.002	30	7	7	35	335	27.64	28.23	28.22	28.46	28.34	28.27	28.25		0.74	0.3371	-0.33
CLAU_3259	putative dual-specificity RNA methyltrar	0.008	10	2	2	14	345	27.31	*23.00*	28.84	27.82	28.68	28.71	28.25		0.50	0.4867	-2.02
CLAU_0065	Transcriptional regulator GntR family	0.013	16	3	3	10	208	28.40	28.27	26.93	27.51	28.22	28.50	28.25		0.14	0.8271	-0.21
CLAU_2908	Periplasmic binding protein domain-con	0.002	24	5	5	31	336	28.03	28.23	28.25	28.18	28.35	28.24	28.24		0.43	0.5335	-0.09
CLAU_3859	Spore germination protein	0.143	3	1	1	2	364	*22.85*	27.07	27.84	29.50	28.75	28.63	28.24		0.90	0.2633	-3.04
CLAU_2827	hypothetical protein	0.004	12	2	2	21	201	27.80	28.98	27.51	28.84	28.66	27.53	28.23		0.15	0.8133	-0.25
CLAU_1725	Endonuclease	0.004	18	3	3	23	214	28.18	28.05	28.06	28.28	28.70	28.46	28.23		1.39	0.1305	-0.38
CLAU_2362	Carboxyl-terminal protease	0.004	18	5	5	21	391	27.50	28.01	27.70	28.83	28.69	28.45	28.23		2.12	0.0530	-0.92
CLAU_0008	Amino acid permease	0.019	2	1	1	6	501	28.27	28.17	27.61	27.60	28.44	28.27	28.22		0.09	0.8826	-0.09
CLAU_2405	putative RNA methylase family protein	0.004	9	3	3	26	376	27.93	27.23	28.41	28.67	28.44	28.02	28.22		0.59	0.4181	-0.52
CLAU_3120	Exodeoxyribonuclease 7 large subunit	0.004	23	5	5	17	398	27.41	28.27	28.00	28.15	28.42	28.38	28.21		0.72	0.3444	-0.42
CLAU_2265	hypothetical protein	0.009	20	2	2	12	138	27.79	27.77	29.03	28.62	26.66	28.92	28.21		0.05	0.9297	0.13
CLAU_0151	putative transcriptional regulator PucR f	0.012	8	4	4	13	517	27.48	27.50	28.26	28.15	30.48	29.81	28.21		1.10	0.1940	-1.73
CLAU_0232	N-acetyl-gamma-glutamyl-phosphate re	0.004	15	4	4	24	344	27.76	27.92	28.24	28.16	28.41	28.27	28.20		0.90	0.2626	-0.31
CLAU_1348	hypothetical protein	0.001	13	3	3	38	242	27.53	27.29	27.88	28.50	28.75	28.72	28.19	+	2.35	0.0407	-1.09
CLAU_1177	ATP-dependent helicase	0.004	9	9	9	24	1232	28.04	27.68	27.77	28.33	28.56	30.05	28.19		0.98	0.2299	-1.15
CLAU_2052	Single-strand binding protein	0.004	17	2	2	20	146	28.29	28.36	28.27	15.99	28.08	27.96	28.18		0.46	0.5111	4.30
CLAU_1717	DegV family protein	0.004	16	4	4	22	279	27.92	27.56	28.42	28.14	28.20	28.68	28.17		0.55	0.4491	-0.37
CLAU_1737	Long-chain-fatty-acid-CoA ligase	0.335	2	1	1	1	571	27.08	*20.16*	27.88	28.89	28.45	29.21	28.17		0.71	0.3545	-3.81
CAETHG_05115	mob protein	0.009	5	2	2	12	368	28.15	29.50	28.92	27.11	28.17	26.99	28.16		1.24	0.1593	1.43
CLAU_2340	Methyl-accepting chemotaxis sensory tr	0.004	12	6	6	27	662	28.90	28.17	28.14	27.53	28.53	27.99	28.16		0.43	0.5314	0.39
CLAU_3646	Beta-lactamase A	0.004	20	5	5	21	257	28.44	28.52	27.83	27.73	28.16	28.15	28.16		0.41	0.5500	0.25
CLAU_2357	Transcriptional modulator of MazE/toxir	0.013	22	2	2	9	116	27.70	27.97	28.35	28.34	27.63	28.35	28.16		0.12	0.8505	-0.10
CLAU_0218	UBA/THIF-type NAD/FAD binding protei	0.014	8	2	2	8	251	28.36	27.49	27.76	28.01	28.38	28.30	28.16		0.57	0.4320	-0.36

CLAU_3199	Ethanolamine utilization protein EutJ far	0.019	8	2	2	6	280	28.55	28.21	28.09	27.51	27.93	28.39	28.15		0.52	0.4719	0.34
CLAU_2776	hypothetical protein	0.009	13	3	3	11	288	28.49	28.17	27.95	28.25	28.11	28.07	28.14		0.13	0.8371	0.06
CLAU_2327	1-(5-phosphoribosyl)-5-amino-4-imidazo	0.007	14	3	3	14	248	27.71	28.04	27.87	28.29	28.56	28.23	28.14		1.60	0.1005	-0.49
CLAU_0853	DegS sensor signal transduction histidin	0.065	14	3	3	4	393	28.63	28.34	26.50	28.05	27.82	28.20	28.13		0.11	0.8672	-0.20
CLAU_0542	DegV family protein	0.016	7	2	2	7	283	28.00	27.74	27.99	28.24	28.37	28.50	28.12		1.81	0.0754	-0.46
CLAU_3269	Methyltransferase	0.007	32	5	5	15	186	28.06	27.77	28.16	29.10	27.88	28.33	28.11		0.51	0.4725	-0.44
CLAU_0936	putative protein YlaK family	0.022	12	5	5	6	460	27.92	27.30	26.57	28.30	28.45	28.51	28.11		1.37	0.1349	-1.16
CLAU_3070	hypothetical protein	0.004	15	3	3	20	237	28.08	27.85	28.13	28.00	28.71	28.37	28.11		0.70	0.3581	-0.34
CLAU_2325	Phosphoribulokinase/uridine kinase fam	0.004	20	9	9	26	571	27.79	27.88	27.62	29.10	28.98	28.32	28.10		1.82	0.0744	-1.04
CLAU_0984	Hypothetical protein	0.044	8	4	4	5	636	28.20	28.56	27.04	26.77	28.07	28.13	28.10		0.16	0.7989	0.28
CLAU_1632	ATPase putative protein family UPF0079	0.017	5	1	1	7	151	28.18	27.95	27.85	28.19	28.47	28.01	28.10		0.62	0.3968	-0.23
CLAU_3315	Ribosome-binding factor A	0.000	16	2	2	85	121	28.13	28.24	28.35	27.65	27.72	28.05	28.09		1.45	0.1218	0.43
CLAU_2591	Hypothetical protein	0.004	7	6	6	17	781	27.86	27.69	27.69	28.32	28.87	28.69	28.09		2.17	0.0504	-0.88
CLAU_0223	Amino acid permease	0.009	5	2	2	12	467	28.28	28.67	27.56	28.02	27.92	28.16	28.09		0.15	0.8119	0.14
CLAU_0596	Prephenate dehydratase	0.018	16	3	3	6	276	27.77	28.00	28.03	28.15	28.35	28.29	28.09		1.51	0.1133	-0.33
CLAU_0242	HAD-superfamily subfamily IB hydrolase	0.004	18	4	4	19	241	27.91	27.97	27.96	28.51	28.20	28.27	28.09		1.78	0.0786	-0.38
CLAU_1300	Cell division protein ZapA	0.020	10	2	2	6	199	27.87	27.63	28.41	28.92	28.13	28.04	28.09		0.47	0.5071	-0.39
CLAU_0980	hypothetical protein	0.117	2	1	1	2	660	28.44	24.92	27.73	28.59	30.04	23.97	28.09		0.08	0.8945	-0.50
CLAU_1863	Peptidase C26	0.012	20	4	4	11	264	25.77	26.87	24.91	29.54	29.29	29.72	28.08	+	2.49	0.0379	-3.67
CLAU_2657	ATPase associated with various cellular	0.028	5	2	2	5	371	28.02	26.03	24.35	29.30	28.14	28.64	28.08		1.08	0.2015	-2.56
CLAU_2048	Phosphoesterase RecJ domain protein	0.005	7	6	6	16	661	27.42	27.25	27.97	28.40	28.18	28.39	28.08		1.56	0.1059	-0.78
CLAU_1938	putative transcriptional acitvator Baf far	0.004	34	7	7	17	259	27.76	28.18	28.07	27.17	28.06	28.21	28.07		0.21	0.7395	0.19
CLAU_2650	NLPA lipoprotein	0.044	4	1	1	3	279	28.21	27.90	28.02	28.14	28.09	27.93	28.06		0.03	0.9594	-0.01
CLAU_2146	DNA polymerase III subunits gamma and	0.013	7	4	4	11	559	27.82	27.21	27.71	28.35	28.41	28.28	28.05		1.79	0.0769	-0.77
CLAU_0753	Transcriptional regulator MerR family	0.142	13	1	1	1	71	28.39	28.18	28.41	27.71	27.90	27.62	28.04	+	2.21	0.0483	0.58
CLAU_0889	Hypothetical protein	0.004	26	5	5	20	175	27.43	27.27	27.91	28.16	28.54	28.32	28.04		1.65	0.0930	-0.80
CLAU_2208	2-C-methyl-D-erythritol 2,4-cyclodiphos	0.004	30	4	4	25	159	28.00	28.07	27.82	28.12	28.06	27.79	28.03		0.07	0.9053	-0.03
CLAU_2743	putative rod shape-determining protein	0.003	6	2	2	29	373	27.88	28.00	27.96	28.05	28.42	28.24	28.03		1.21	0.1649	-0.29
CLAU_3476	Diguanylate cyclase/phosphodiesterase	0.004	12	7	7	24	657	28.37	27.97	28.41	27.62	28.00	28.05	28.03		0.86	0.2822	0.36
CLAU_2127	Gluconate transporter	0.000	2	1	1	58	449	27.19	26.47	27.71	28.93	29.36	28.32	28.02		1.69	0.0866	-1.75
CLAU_0105	Deoxyribonuclease IV (phage-T(4)-induc	0.004	7	2	2	25	298	27.94	27.50	28.47	28.35	28.09	27.65	28.02		0.06	0.9229	-0.06
CLAU_0915	Regulatory protein ArsR	0.005	27	2	2	16	95	27.13	27.56	27.69	28.34	28.58	28.41	28.02	+	2.23	0.0474	-0.98
CLAU_3953	2Fe-2S iron-sulfur cluster binding domai	0.007	4	2	2	15	597	27.17	27.46	28.16	28.05	28.65	27.98	28.02		0.80	0.3063	-0.63
CLAU_2541	WxcM-like domain-containing protein	0.094	6	1	1	2	135	27.88	28.03	28.16	28.00	27.98	28.20	28.02		0.13	0.8456	-0.04
CLAU_3371	Arsenate reductase-like protein	0.004	17	2	2	18	112	28.52	28.45	28.85	26.87	26.95	27.57	28.01	+	2.37	0.0407	1.48
CLAU_3305	Undecaprenyl pyrophosphate synthase	0.004	28	6	6	30	255	28.25	28.52	27.08	27.26	28.32	27.76	28.01		0.11	0.8569	0.17
CLAU_2003	Primosome DnaD subunit	0.007	15	5	5	15	332	27.78	27.87	27.81	28.14	28.73	28.68	28.01		1.66	0.0912	-0.70
CLAU_1280	Hypothetical protein	0.004	17	6	6	24	360	28.63	27.90	27.65	27.82	28.19	28.10	28.00		0.02	0.9674	0.02
CLAU_2219	Phage SPO1 DNA polymerase-related pr	0.007	17	3	3	13	192	27.94	28.16	28.40	28.05	27.37	27.17	28.00		1.00	0.2228	0.64
CLAU_2316	RelA/SpoT domain protein	0.000	27	7	7	95	262	23.10	28.06	28.86	28.25	27.92	26.49	27.99		0.18	0.7816	-0.88
CLAU_0458	Pyridine nucleotide-disulfide oxidoreduc	0.004	23	7	7	20	422	28.20	28.21	28.03	27.94	26.39	27.26	27.99		0.98	0.2292	0.95
CLAU_3209	Rrf2 family protein	0.007	18	2	2	15	148	27.77	27.97	28.14	28.42	27.99	27.98	27.99		0.40	0.5588	-0.17
CLAU_3808	Ethanolamine utilization protein EutP	0.009	19	3	3	18	161	28.94	28.81	28.96	25.51	25.67	27.16	27.99	+	2.21	0.0483	2.79
CLAU_3047	Site-determining protein	0.017	9	3	3	7	292	26.93	26.71	28.48	28.51	28.06	27.91	27.99		0.60	0.4122	-0.79
CLAU_1299	Peptidase U32	0.004	9	7	7	18	789	27.91	27.77	27.70	28.10	28.39	28.05	27.98		1.46	0.1203	-0.39
CLAU_0440	Transcriptional regulator TetR family	0.018	9	2	2	7	195	27.98	26.67	27.39	27.95	28.33	28.09	27.97		0.92	0.2545	-0.78

CLAU_1352	Beta-lactamase domain protein	0.004	19	4	4	18	268	26.91	28.26	27.85	28.07	27.26	28.49	27.96		0.19	0.7675	-0.27
CLAU_0152	Methionine synthase	0.007	10	2	2	13	211	28.20	27.87	27.58	26.43	28.03	28.06	27.95		0.27	0.6812	0.38
CLAU_0222	Formiminotransferase-cyclodeaminase	0.006	21	4	4	16	212	28.07	28.81	28.21	27.27	27.29	27.83	27.95		1.43	0.1245	0.90
CLAU_1242	Holliday junction DNA helicase ruvB	0.007	5	2	2	14	348	28.24	27.77	27.73	27.91	27.97	29.79	27.94		0.43	0.5335	-0.64
CLAU_1584	Hypothetical protein	0.028	40	3	3	5	73	24.02	25.82	28.96	28.54	28.85	27.34	27.94		0.58	0.4249	-1.98
CLAU_0788	Gluconate transporter	0.004	9	3	3	17	441	28.58	28.97	29.24	27.28	26.95	26.64	27.93	+	2.75	0.0285	1.97
CLAU_2566	DTDP-glucose 4,6-dehydratase	0.012	13	3	1	12	351	27.17	28.10	27.84	27.73	28.13	28.02	27.93		0.35	0.6012	-0.26
CLAU_0266	PHP domain protein	0.013	14	4	4	9	279	27.34	27.64	27.70	28.31	28.42	28.16	27.93	+	2.27	0.0459	-0.74
CLAU_1266	Hypothetical protein	0.001	5	2	2	37	419	27.83	27.22	26.93	28.02	28.36	28.32	27.93		1.47	0.1196	-0.91
CLAU_1476	hypothetical protein	0.004	22	4	4	23	215	27.84	27.30	27.97	27.88	28.13	28.12	27.93		0.70	0.3566	-0.34
CLAU_3056	Calcium-translocating P-type ATPase	0.260	1	1	1	1	846	24.81	23.16	28.41	28.21	27.86	27.99	27.93		0.76	0.3289	-2.56
CLAU_2119	Virulence protein RhuM	0.013	6	2	2	9	337	28.20	28.35	28.02	27.39	27.78	27.82	27.92		1.46	0.1203	0.53
CLAU_1781	Glycyl-radical enzyme activating protein	0.013	9	3	3	13	316	28.48	28.22	27.21	27.83	27.58	28.01	27.92		0.15	0.8150	0.16
CLAU_3081	Uracil-xanthine permease	0.064	4	2	2	2	432	26.01	*20.76*	27.84	28.23	28.66	28.00	27.92		0.74	0.3377	-3.43
CLAU_3026	Flagellin domain protein	0.001	22	5	5	35	282	26.80	26.86	26.58	29.16	29.16	28.96	27.91	+	4.57	0.0046	-2.35
CLAU_2711	ATP-dependent helicase/deoxyribonucl	0.004	8	8	8	19	1150	27.83	28.09	27.42	27.48	28.05	27.99	27.91		0.08	0.8983	-0.06
CLAU_3520	ABC-type transporter related protein	0.006	20	3	3	17	243	27.93	27.32	28.01	27.81	27.88	28.35	27.91		0.40	0.5592	-0.26
CLAU_0935	hypothetical protein	0.013	11	3	3	10	336	28.65	28.37	28.03	27.21	27.19	27.78	27.91		1.65	0.0930	0.96
CLAU_1213	Glycosyl transferase family 2	0.013	12	3	3	9	339	26.99	28.31	27.84	27.97	27.97	26.74	27.91		0.10	0.8785	0.15
CLAU_3149	Electron transport complex subunit B	0.001	45	8	8	35	284	27.94	27.72	27.95	27.06	27.86	28.63	27.90		0.01	0.9808	0.02
CLAU_1423	Ferric uptake regulator Fur family	0.016	6	1	1	7	140	27.97	27.95	27.90	27.90	27.78	27.70	27.90		1.12	0.1891	0.15
CLAU_0902	Nitroreductase	0.007	8	2	2	13	272	28.18	27.89	24.44	27.88	28.40	27.89	27.89		0.43	0.5335	-1.22
CLAU_3245	hypothetical protein	0.001	18	6	6	40	444	27.75	28.29	27.74	27.41	28.23	28.01	27.88		0.05	0.9370	0.04
CLAU_3127	DNA repair protein RecN	0.006	10	6	6	17	565	27.92	27.77	27.12	27.84	28.40	28.32	27.88		0.90	0.2625	-0.58
CLAU_1174	Polar amino acid ABC transporter inner	0.033	11	2	2	4	212	27.69	27.64	27.55	28.12	28.07	28.08	27.88	+	3.35	0.0187	-0.46
CLAU_3121	Exodeoxyribonuclease 7 small subunit	0.000	58	3	3	60	74	27.12	26.83	28.00	28.33	28.36	27.74	27.87		0.95	0.2404	-0.83
CLAU_0238	hypothetical protein	0.004	19	6	6	19	424	27.01	27.33	27.14	28.41	28.87	28.63	27.87	+	3.09	0.0231	-1.48
CLAU_0077	FMN-dependent nitroreductase	0.007	10	3	3	14	177	27.47	27.20	27.43	28.25	28.60	28.32	27.86	+	2.78	0.0283	-1.02
CLAU_1378	TfoX domain-containing protein	0.013	13	1	1	9	84	29.16	27.81	27.58	27.29	27.89	28.23	27.85		0.27	0.6765	0.38
CLAU_0727	Asparagine synthase (glutamine-hydroly	0.028	6	3	3	5	613	27.15	28.80	26.89	24.98	28.80	28.54	27.85		0.04	0.9456	0.17
CLAU_0209	S-adenosylmethionine decarboxylase pr	0.023	24	3	3	6	126	27.31	28.51	27.76	27.87	28.18	27.78	27.83		0.08	0.8983	-0.08
CLAU_0488	Dihydrofolate reductase	0.013	14	2	2	8	159	27.69	27.39	27.75	28.54	28.13	27.87	27.81		1.19	0.1703	-0.57
CLAU_0489	putative membrane protein DUF2207	0.035	5	2	2	4	601	27.81	27.20	27.81	27.94	28.02	27.53	27.81		0.37	0.5866	-0.22
CLAU_1842	hypothetical protein	0.005	8	2	2	16	278	27.63	27.63	28.39	28.50	27.63	27.98	27.81		0.16	0.8019	-0.15
CLAU_2703	DNA-binding protein	0.018	3	1	1	6	290	27.79	27.45	27.86	27.49	27.97	27.82	27.81		0.11	0.8569	-0.06
CLAU_3195	Microcompartments protein	0.023	7	2	2	8	287	28.00	27.96	27.92	27.21	27.39	27.68	27.80		1.73	0.0827	0.53
CLAU_0182	Transcriptional regulator AsnC family	0.007	27	4	4	17	150	26.41	*21.81*	*22.19*	29.69	29.20	29.18	27.80		1.78	0.0786	-5.88
CLAU_3186	Integral membrane sensor signal transd	0.036	1	1	1	5	503	27.00	26.06	27.53	28.25	28.42	28.06	27.80		1.45	0.1219	-1.38
CLAU_0950	PadR family transcriptional regulator	0.037	16	3	3	7	183	26.07	28.78	29.06	28.57	25.83	27.02	27.80		0.27	0.6783	0.83
CLAU_2706	NfeD-like protein	0.013	6	1	1	9	141	27.72	28.17	27.77	27.67	27.81	27.94	27.79		0.19	0.7683	0.08
CLAU_1015	Citrate lyase alpha subunit	0.009	8	3	3	11	513	27.30	26.77	29.49	27.85	30.56	27.72	27.79		0.28	0.6690	-0.86
CLAU_0578	Citrate lyase alpha subunit	0.009	8	3	3	11	513	27.30	26.77	29.49	27.85	30.56	27.72	27.79		0.28	0.6690	-0.86
CLAU_3083	Fibronectin-binding A domain protein	0.004	14	8	8	20	578	28.32	28.23	25.95	26.88	27.96	27.60	27.78		0.01	0.9891	0.02
CLAU_2726	Amino acid permease	0.004	9	4	4	22	613	27.56	27.96	27.71	27.36	27.85	27.82	27.77		0.12	0.8465	0.07
CLAU_2015	ApbE family lipoprotein	0.013	7	2	2	9	359	26.90	25.50	24.70	29.18	28.63	29.33	27.77		2.11	0.0534	-3.35
CLAU_1716	RNA polymerase sigma-54 subunit	0.009	15	6	6	12	455	28.28	27.56	27.71	27.81	27.59	28.03	27.76		0.05	0.9297	0.04

CLAU_1641	Phosphoesterase PA-phosphatase relate	0.029	10	3	3	5	328	28.73	28.24	27.36	27.39	27.71	27.80	27.76		0.50	0.4869	0.48
CLAU_3545	Allantoinase	0.076	2	1	1	3	455	27.95	27.56	27.39	27.45	27.97	28.02	27.76		0.30	0.6481	-0.18
CLAU_0174	hypothetical protein	0.011	11	1	1	11	70	29.07	29.13	28.77	26.02	26.32	26.73	27.75	+	3.45	0.0182	2.63
CLAU_1511	Aspartyl/glutamyl-tRNA(Asn/Gln) amidc	0.029	6	2	2	7	476	26.09	27.82	27.98	26.77	27.99	27.62	27.72		0.08	0.8971	-0.16
CLAU_1498	Hypothetical protein	0.004	22	7	7	21	379	27.24	26.58	26.50	28.30	28.17	28.43	27.71	+	2.46	0.0389	-1.53
CLAU_0289	Phospholipase C/P1 nuclease	0.017	15	3	3	7	234	27.07	27.37	25.63	28.16	28.57	28.04	27.71		1.31	0.1462	-1.57
CLAU_1955	HtrA2 peptidase	0.009	9	3	3	11	392	26.61	27.10	27.20	28.37	28.52	28.18	27.69	+	2.59	0.0341	-1.39
CLAU_2907	Formate dehydrogenase	0.009	4	3	3	12	714	27.27	27.22	26.38	28.10	28.32	28.11	27.69		1.83	0.0733	-1.22
CAETHG_05090	protein rep	0.009	11	3	3	11	343	26.92	27.41	25.45	30.37	28.52	27.96	27.69		1.18	0.1720	-2.36
CLAU_2408	Acriflavin resistance protein	0.007	7	6	6	16	1018	27.72	27.65	27.01	27.26	28.04	27.92	27.69		0.35	0.6029	-0.28
CLAU_2762	Translation elongation factor	0.007	5	3	3	14	635	28.04	27.30	27.16	27.29	28.81	28.68	27.67		0.61	0.4051	-0.76
CLAU_1016	Citrate lyase beta subunit	0.007	11	3	3	13	300	27.26	27.68	27.98	27.58	27.89	27.65	27.67		0.10	0.8693	-0.07
CLAU_2876	Beta-lactamase domain protein	0.009	17	3	3	12	297	27.61	27.51	26.99	27.83	27.88	27.71	27.66		1.03	0.2170	-0.44
CLAU_1974	Helix-turn-helix domain protein	0.014	9	2	2	8	222	29.11	29.52	27.59	27.25	27.72	22.07	27.66		0.74	0.3377	3.06
CLAU_3800	Peptide deformylase	0.013	23	3	3	10	160	27.87	27.93	27.66	27.09	27.47	27.64	27.65		1.08	0.2010	0.42
CLAU_3130	hypothetical protein	0.013	16	1	1	10	56	24.40	27.72	28.54	28.24	27.58	27.00	27.65		0.21	0.7390	-0.72
CLAU_0230	Acetylglutamate kinase	0.007	7	2	2	15	284	26.53	26.97	27.72	27.71	27.72	27.56	27.64		0.77	0.3213	-0.59
CLAU_2534	Glycosyl transferase group 1	0.007	20	6	6	14	372	26.83	27.62	25.94	27.89	28.23	27.65	27.64		1.03	0.2173	-1.13
CLAU_3668	Hypothetical protein	0.033	13	2	2	4	162	27.59	28.80	27.17	27.68	27.56	28.15	27.64		0.04	0.9527	0.06
CLAU_2879	D-glucuronyl C5-epimerase domain prot	0.007	7	4	4	14	737	27.39	26.68	27.25	28.36	27.87	28.32	27.63		1.80	0.0767	-1.08
CLAU_0137	PTS system fructose subfamily IIC subun	0.002	9	5	5	32	632	31.23	29.14	26.02	27.31	27.52	27.72	27.62		0.35	0.6030	1.28
CLAU_2851	Alpha-acetolactate decarboxylase	0.004	20	4	4	22	239	26.48	25.52	28.06	27.56	27.68	28.43	27.62		0.69	0.3586	-1.20
CLAU_0676	hypothetical protein	0.020	6	2	2	6	333	26.41	27.87	26.62	27.70	27.66	27.58	27.62		0.68	0.3676	-0.68
CLAU_2037	Transcriptional regulator DeoR family	0.007	15	4	4	17	252	27.33	27.26	27.39	27.97	27.98	27.84	27.62	+	3.30	0.0191	-0.60
CLAU_0622	Signal transduction histidine kinase cont	0.033	11	3	3	6	350	27.87	27.61	27.40	26.97	27.68	27.59	27.60		0.34	0.6128	0.21
CLAU_2272	Protein-(glutamine-N5) methyltransfera	0.086	9	2	2	2	287	27.44	27.32	26.85	27.87	27.93	27.76	27.60		1.60	0.1005	-0.65
CLAU_2546	Mannose-1-phosphate	0.007	11	4	3	14	459	27.68	28.04	27.70	27.30	26.87	27.51	27.60		1.23	0.1609	0.58
CLAU_2243	UPF0272 protein	0.028	11	4	4	6	425	27.64	27.54	27.23	28.03	28.07	27.16	27.59		0.37	0.5866	-0.28
CLAU_1544	Dihydroorotase multifunctional comple›	0.007	16	5	5	15	429	27.53	27.64	26.39	26.89	27.93	27.75	27.59		0.26	0.6838	-0.34
CLAU_1520	Pseudouridine synthase Rsu	0.012	11	3	3	10	238	27.56	27.47	27.61	27.41	27.81	27.83	27.59		0.41	0.5526	-0.14
CLAU_0629	Transketolase	0.044	7	2	2	6	288	28.22	28.52	25.97	26.74	27.46	27.71	27.59		0.11	0.8596	0.27
CLAU_0374	putative membrane protein	0.004	8	4	4	18	554	27.51	27.83	24.81	27.35	27.65	27.78	27.58		0.38	0.5745	-0.88
CLAU_1786	Hypothetical protein	0.004	28	2	2	25	88	27.65	27.48	27.81	27.16	26.84	27.65	27.57		0.78	0.3183	0.43
CLAU_1517	DNA ligase	0.012	12	6	6	11	664	27.43	27.70	27.42	26.59	27.91	28.53	27.57		0.10	0.8763	-0.16
CLAU_2180	Periplasmic binding protein domain-con	0.013	8	2	2	9	363	26.48	26.72	27.34	28.30	28.13	27.78	27.56		1.83	0.0733	-1.22
CLAU_0136	Uroporphyrinogen decarboxylase (URO-	0.005	19	6	6	18	380	28.01	27.55	26.81	27.56	27.21	27.80	27.56		0.06	0.9237	-0.07
CLAU_3049	RNA polymerase sigma factor	0.007	12	3	3	18	241	28.23	27.52	27.59	27.99	27.09	27.37	27.56		0.35	0.6012	0.30
CLAU_3309	4-hydroxy-3-methylbut-2-en-1-yl diphos	0.013	11	3	3	9	349	27.63	27.30	26.73	27.55	27.56	27.69	27.56		0.64	0.3845	-0.38
CLAU_2599	Hypothetical protein	0.004	20	6	6	17	424	27.41	27.30	27.44	27.66	27.83	28.48	27.55		1.13	0.1872	-0.61
CLAU_0184	Uroporphyrinogen decarboxylase (URO-	0.070	7	2	2	3	358	27.31	27.14	26.82	28.15	28.22	27.78	27.55		2.08	0.0546	-0.96
CLAU_1420	Adenosylcobinamide-phosphate	0.007	4	1	1	14	182	27.20	27.43	27.53	27.55	27.69	27.64	27.54		1.06	0.2075	-0.24
CLAU_0834	Hypothetical protein	0.013	7	2	2	10	323	27.70	27.87	27.63	27.25	27.44	27.28	27.54		1.94	0.0647	0.41
CLAU_2608	hypothetical protein	0.006	7	2	2	16	316	27.20	27.33	27.21	28.36	28.13	27.72	27.53		1.90	0.0683	-0.82
CLAU_2190	Thymidylate kinase	0.007	19	4	4	13	227	27.50	27.32	27.47	27.55	27.84	27.87	27.53		1.30	0.1468	-0.32
CLAU_3518	Peptidase M42 family protein	0.013	14	4	4	9	346	27.68	27.79	27.34	27.35	27.77	26.68	27.52		0.42	0.5453	0.34
CLAU_3537	Transcriptional regulator CdaR family	0.051	4	2	2	5	557	26.71	26.86	*20.75*	29.02	28.16	28.24	27.51		0.85	0.2862	-3.70

CLAU_2234	Beta-lactamase	0.018	14	2	2	6	210	26.65	27.12	27.60	27.80	27.76	27.38	27.49	0.79	0.3127	-0.52
CLAU_0007	Peptidase T	0.007	20	5	5	15	408	27.83	27.27	27.45	26.91	27.52	27.64	27.49	0.22	0.7262	0.16
CLAU_0188	2-hydroxyglutaryl-CoA dehydratase D-cc	0.012	13	4	4	11	382	27.21	27.04	25.45	27.76	28.13	27.94	27.49	1.13	0.1852	-1.38
CLAU_2315	hypothetical protein	0.006	19	3	3	16	267	27.19	27.54	27.44	27.52	27.75	27.29	27.48	0.32	0.6341	-0.13
CLAU_2697	Glycosyl transferase group 1	0.009	20	6	6	13	406	26.60	27.24	26.74	27.77	27.88	27.72	27.48	2.02	0.0598	-0.93
CLAU_0912	Methyltransferase type 11	0.012	11	2	2	10	186	29.33	29.11	28.84	*21.11*	25.98	26.11	27.48	1.33	0.1426	4.69
CLAU_0423	Sulfite reductase subunit A	0.023	7	3	3	7	333	27.68	27.99	28.44	27.26	26.58	26.82	27.47	1.74	0.0816	1.15
CLAU_1622	Hypothetical protein	0.009	11	3	3	11	292	26.87	27.42	27.27	27.67	27.73	27.50	27.46	1.18	0.1722	-0.45
CLAU_1437	Dihydroorotate dehydrogenase	0.023	3	1	1	5	296	27.56	27.31	27.42	27.34	27.53	27.50	27.46	0.10	0.8722	-0.03
CLAU_2373	putative protein family UPF0052	0.007	10	4	4	14	442	27.26	27.34	27.03	27.86	27.57	27.64	27.46	1.71	0.0854	-0.48
CLAU_2131	Ketose-bisphosphate aldolase	0.009	31	5	5	13	278	25.02	26.46	26.57	29.48	29.19	28.33	27.45	2.10	0.0537	-2.98
CLAU_2451	Two component transcriptional regulatc	0.013	9	2	2	9	225	26.66	26.14	27.23	27.66	27.66	27.66	27.45	1.45	0.1218	-0.98
CLAU_1648	(FeFe)-hydrogenase maturation hydE	0.049	8	2	2	3	346	27.35	27.30	27.32	27.56	27.53	27.92	27.44	1.29	0.1505	-0.35
CLAU_2197	Motility protein B	0.005	10	2	2	19	283	27.98	27.85	27.39	27.14	26.98	27.48	27.44	1.09	0.1968	0.54
CLAU_0211	Cytidylate kinase	0.007	20	3	3	16	221	27.45	27.94	27.79	27.42	26.73	26.92	27.44	1.31	0.1462	0.70
CLAU_1748	3-isopropylmalate dehydrogenase	0.005	13	4	4	20	360	27.60	27.06	27.80	26.98	27.25	27.60	27.43	0.30	0.6451	0.21
CLAU_2754	putative nicotinate-nucleotide	0.018	12	2	2	6	203	26.89	25.93	27.34	27.74	27.76	27.51	27.43	1.05	0.2109	-0.95
CLAU_2173	Aldose 1-epimerase	0.006	15	4	4	16	344	27.38	27.88	27.46	27.14	27.34	27.53	27.42	0.55	0.4491	0.24
CLAU_3797	Cof-like hydrolase	0.005	14	3	3	16	293	27.26	27.33	27.01	27.56	27.65	27.50	27.42	1.59	0.1006	-0.37
CLAU_0432	Diaminopropionate ammonia-lyase	0.011	17	4	4	11	393	27.31	26.85	27.03	27.59	27.53	27.52	27.42	1.63	0.0964	-0.48
CLAU_3223	Peptidase U32	0.004	24	8	8	19	408	26.35	27.76	27.84	27.46	27.36	27.03	27.41	0.02	0.9696	0.03
CLAU_3289	Ribosome maturation factor rimM	0.007	40	5	5	13	162	27.80	27.74	28.52	27.07	27.02	26.87	27.41	1.80	0.0768	1.03
CLAU_2581	MgtC/SapB transporter	0.007	11	2	2	13	220	27.19	27.64	28.12	27.34	27.28	27.45	27.40	0.46	0.5111	0.29
CLAU_2670	Two component transcriptional regulatc	0.014	3	1	1	12	229	27.69	26.88	27.61	27.37	27.40	27.39	27.40	0.01	0.9891	0.01
CLAU_1035	DNA polymerase III polC-type	0.004	8	10	10	19	1449	27.20	27.34	27.35	27.56	27.91	27.43	27.39	1.05	0.2144	-0.34
CLAU_3595	Transcriptional regulator DeoR family	0.007	15	3	3	13	253	27.07	27.28	27.27	27.62	27.52	27.50	27.39	1.92	0.0654	-0.34
CLAU_0835	Major facilitator superfamily MFS_1	0.007	4	2	2	13	436	27.27	26.42	27.37	27.40	27.68	27.88	27.39	0.89	0.2692	-0.63
CLAU_3251	Guanylate kinase	0.037	3	1	1	4	205	27.68	27.39	27.35	27.34	27.43	27.30	27.37	0.45	0.5193	0.12
CLAU_3165	Xylulokinase	0.056	4	2	2	3	512	27.12	27.08	27.33	27.45	27.66	27.39	27.36	1.34	0.1405	-0.32
CLAU_0417	Molybdopterin-family oxidoreductase b	0.139	6	2	2	2	407	27.26	27.45	28.10	27.27	27.18	27.53	27.36	0.43	0.5335	0.28
CLAU_2201	NLP/P60 protein	0.007	12	3	2	14	348	27.30	27.73	27.40	26.12	26.57	27.46	27.35	0.85	0.2845	0.76
CLAU_1953	Transcription-repair coupling factor	0.007	9	9	9	16	1173	27.89	26.69	27.52	27.29	27.41	27.06	27.35	0.11	0.8619	0.11
CLAU_2932	DNA gyrase subunit B	0.049	5	2	2	5	649	27.52	26.85	27.77	26.92	27.69	27.17	27.35	0.12	0.8474	0.12
CLAU_3278	Phosphate acyltransferase	0.015	13	4	4	8	337	25.44	25.72	26.86	27.88	27.98	27.82	27.34	1.90	0.0674	-1.89
CLAU_0416	Pyridoxamine 5-phosphate oxidase-rela	0.013	19	2	2	9	133	27.49	27.34	27.33	27.09	27.26	27.48	27.34	0.37	0.5850	0.11
CLAU_3030	Flagellar M-ring protein	0.009	10	4	4	12	532	27.84	28.64	27.26	25.76	27.33	27.31	27.32	0.78	0.3174	1.11
CLAU_3190	Flavin reductase domain protein	0.016	13	2	2	7	164	24.16	22.06	29.48	29.83	27.59	27.05	27.32	0.55	0.4491	-2.92
CLAU_3053	Flagellar basal body rod protein	0.005	24	4	4	16	262	27.17	27.46	26.97	27.55	27.84	26.43	27.32	0.06	0.9297	-0.07
CLAU_2199	Ribosomal RNA small subunit methyltra	0.012	10	3	3	10	279	27.48	27.23	27.14	26.97	27.42	27.39	27.31	0.04	0.9422	0.02
CLAU_1836	PAS modulated sigma54 specific transcr	0.013	3	2	2	9	605	27.87	27.55	26.91	27.22	27.36	26.99	27.29	0.35	0.6046	0.25
CLAU_2981	hypothetical protein	0.007	5	2	2	14	361	27.26	27.46	27.30	27.32	27.16	27.25	27.28	0.56	0.4402	0.10
CLAU_0660	ABC-type spermidine/putrescine transp	0.018	12	3	3	7	353	24.63	27.11	27.45	30.04	26.05	28.88	27.28	0.58	0.4266	-1.93
CLAU_0537	Copper-sensing transcriptional represso	0.035	19	2	2	4	88	27.00	26.93	27.29	27.27	27.43	27.50	27.28	1.19	0.1719	-0.33
CLAU_1755	Hypothetical protein	0.009	2	1	1	12	618	27.04	27.47	27.31	26.52	27.34	27.24	27.28	0.35	0.6059	0.24
CLAU_2878	Vitamin B12 dependent methionine syn	0.013	15	3	3	8	211	26.48	23.86	28.11	27.25	27.71	27.29	27.27	0.44	0.5314	-1.27
CLAU_0650	NADP-binding oxido reductase	0.126	2	1	1	2	325	27.42	27.22	26.96	26.70	27.43	27.31	27.27	0.07	0.9072	0.05

CLAU_2468	Methionine ABC transporter permease	0.033	5	1	1	4	218	27.77	27.42	27.61	26.58	26.87	27.09	27.26		1.87	0.0708	0.75
CLAU_3627	Peptidase S41	0.307	3	1	1	1	430	27.49	28.37	22.93	27.94	27.01	26.83	27.25		0.23	0.7229	-1.00
CLAU_0028	Hypothetical protein	0.207	16	1	1	1	77	27.24	25.98	27.68	27.05	27.23	27.39	27.24		0.19	0.7675	-0.26
CLAU_2187	Mg2 transporter protein CorA family pro	0.034	5	2	2	4	313	27.34	26.46	27.18	27.02	27.63	27.28	27.23		0.42	0.5435	-0.32
CLAU_0817	putative radical SAM protein	0.004	16	4	4	17	233	27.22	25.77	25.92	27.24	27.57	27.47	27.23		1.12	0.1880	-1.12
CLAU_3009	putative NAD dependent epimerase/del	0.005	20	5	5	19	336	26.76	26.80	26.27	27.65	28.57	28.20	27.23		2.07	0.0549	-1.53
CLAU_0667	4Fe4S-binding SPASM domain-containir	0.009	14	5	5	12	445	27.28	26.55	27.03	27.18	27.26	27.44	27.22		0.68	0.3666	-0.34
CLAU_3757	Polysaccharide biosynthesis protein	0.013	10	4	4	9	535	27.13	26.86	27.14	27.29	27.79	27.75	27.22		1.43	0.1258	-0.57
CLAU_2339	Anti-sigma-factor antagonist	0.075	9	1	1	2	94	27.60	27.38	27.72	26.73	26.84	27.05	27.22		2.15	0.0512	0.69
CLAU_1919	Mini-ribonuclease 3	0.004	24	4	4	26	141	27.07	27.09	26.91	27.82	27.88	27.31	27.20		1.57	0.1051	-0.65
CLAU_2392	hypothetical protein	0.046	41	3	3	3	109	26.34	26.39	27.18	27.31	27.32	27.22	27.20		1.11	0.1917	-0.65
CLAU_3281	Radical SAM domain protein	0.018	5	2	2	8	355	27.16	27.41	26.60	26.60	27.27	27.22	27.19		0.03	0.9628	0.03
CLAU_2312	Hypothetical protein	0.012	4	3	3	10	612	27.32	26.34	27.46	27.10	27.04	27.27	27.19		0.10	0.8798	-0.10
CLAU_2353	Holo-(acyl-carrier-protein) synthase	0.018	17	2	2	6	134	27.00	27.32	27.15	27.06	27.47	27.21	27.18		0.23	0.7169	-0.09
CLAU_0273	Transcriptional regulator PucR family	0.014	4	3	3	8	542	27.00	27.06	27.46	27.50	27.17	27.18	27.18		0.24	0.7103	-0.11
CLAU_1591	Hypothetical protein	0.054	13	2	2	4	272	24.70	26.60	25.81	28.23	27.97	27.75	27.18		1.80	0.0768	-2.28
CLAU_3952	Methyltransferase MtaA/CmuA family	0.013	14	4	4	10	343	26.75	26.66	26.91	27.53	27.80	27.43	27.17	+	2.45	0.0390	-0.81
CLAU_2550	DTDP-4-dehydrorhamnose 3,5-epimera:	0.019	11	2	2	6	185	27.09	26.96	26.98	27.73	27.42	27.25	27.17		1.45	0.1218	-0.46
CLAU_0642	Fructose-6-phosphate aldolase	0.009	27	4	4	12	225	26.72	26.23	27.10	27.20	27.58	27.43	27.15		1.23	0.1609	-0.72
CLAU_0185	Methionine synthase	0.013	12	2	2	10	219	26.86	27.03	26.20	27.39	27.26	27.51	27.15		1.23	0.1608	-0.69
CLAU_0593	Anaerobic sulfite reductase subunit A	0.013	17	6	6	10	352	27.37	27.91	26.91	26.41	27.91	26.56	27.14		0.32	0.6279	0.44
CLAU_2517	Hypothetical protein	0.007	18	5	5	15	417	26.83	26.04	27.30	27.40	26.97	27.43	27.14		0.62	0.4028	-0.54
CLAU_1359	Hypothetical protein	0.013	10	6	6	9	584	26.97	27.01	27.09	27.96	27.18	27.23	27.14		0.79	0.3161	-0.43
CLAU_3756	Pseudouridine synthase RluA family	0.090	6	2	2	2	319	27.41	27.16	27.24	25.58	27.11	26.61	27.14		0.85	0.2845	0.84
CLAU_3893	Hypothetical protein	0.343	7	1	1	1	237	26.92	27.04	27.29	27.33	27.17	26.97	27.11		0.19	0.7706	-0.07
CLAU_1181	Cobalamin (vitamin B12) biosynthesis Cl	0.008	2	1	1	12	332	27.47	27.42	27.19	26.94	26.91	26.99	27.09		2.01	0.0601	0.41
CLAU_2413	Transcriptional regulator AsnC family	0.033	13	2	2	4	142	27.11	26.52	26.82	27.05	27.14	27.45	27.08		0.88	0.2706	-0.40
CLAU_0970	Multi-sensor hybrid histidine kinase	0.143	1	1	1	1	949	27.64	*23.05*	*22.76*	27.78	26.52	28.22	27.08		0.85	0.2866	-3.02
CLAU_3795	Hypothetical protein	0.012	30	2	2	10	92	27.05	27.32	27.07	26.85	27.08	27.30	27.08		0.17	0.7902	0.07
CLAU_1642	Cof-like hydrolase	0.112	11	2	2	2	272	27.16	27.56	26.89	27.33	26.90	26.97	27.07		0.23	0.7229	0.14
CLAU_1354	CRISPR-associated protein Cas1	0.013	7	3	3	10	334	26.65	26.84	26.36	27.26	27.28	27.54	27.05		1.96	0.0637	-0.74
CLAU_0395	RRNA methylase	0.217	14	1	1	1	188	25.67	26.85	27.25	27.49	*21.10*	27.40	27.05		0.23	0.7229	1.26
CLAU_3318	Bifunctional riboflavin kinase/FMN	0.013	21	6	6	10	312	27.09	27.03	27.21	27.06	25.90	26.54	27.05		0.83	0.2916	0.61
CLAU_3501	Diguanylate cyclase with extracellular sc	0.007	8	6	5	13	826	27.31	26.77	27.33	26.76	26.68	27.71	27.04		0.08	0.8983	0.09
CLAU_2625	Peptidoglycan glycosyltransferase	0.059	3	2	2	3	671	25.23	25.88	26.94	27.14	27.46	27.35	27.04		1.20	0.1665	-1.30
CLAU_2045	Replicative DNA helicase	0.064	2	1	1	2	444	27.00	26.56	26.87	27.24	27.07	27.26	27.04		1.24	0.1593	-0.38
CLAU_1147	Na(+)/solute symporter	0.014	7	3	3	8	467	26.30	26.07	25.99	28.38	27.95	27.73	27.02	+	3.06	0.0237	-1.90
CLAU_0277	UPF0291 protein	0.018	10	1	1	8	60	27.69	27.47	27.97	23.91	26.56	26.00	27.02		1.27	0.1537	2.22
CLAU_1019	GntR family transcriptional regulator	0.016	14	3	3	7	224	24.43	26.74	27.27	27.30	27.91	25.85	27.01		0.34	0.6111	-0.87
CLAU_0060	YbaK/prolyl-tRNA synthetase associated	0.021	15	3	3	6	185	26.83	24.84	27.33	27.26	26.53	27.17	27.00		0.34	0.6105	-0.65
CLAU_0122	Biotin carboxylase	0.013	11	6	6	9	653	27.38	27.25	27.73	26.74	26.61	26.56	27.00	+	2.23	0.0477	0.82
CLAU_2640	HTH domain-containing protein	0.017	9	4	4	7	420	27.00	27.35	27.46	24.94	26.90	26.98	26.99		0.66	0.3746	1.00
CLAU_0296	Riboflavin biosynthesis protein RibD	0.017	5	2	2	11	372	25.84	27.95	24.80	28.14	27.60	26.38	26.99		0.48	0.4982	-1.18
CLAU_0274	Mg2 transporter protein CorA family pro	0.338	3	1	1	1	312	27.11	27.21	27.80	26.87	26.79	26.73	26.99		1.24	0.1598	0.58
CLAU_1704	Metal dependent phosphohydrolase	0.067	9	2	2	3	300	25.02	26.95	27.00	26.24	27.56	27.10	26.98		0.35	0.6018	-0.64
CLAU_0933	Peptidase M50	0.044	5	1	1	3	214	26.65	27.06	26.95	27.01	26.82	26.99	26.97		0.15	0.8194	-0.05

CLAU_0700	Transcriptional regulator LytTR family	0.190	6	1	1	1	180	27.38	27.43	*22.15*	26.89	26.83	27.05	26.97	0.29	0.6511	-1.27
CLAU_1597	putative secreted protein	0.004	12	3	3	17	410	25.43	25.31	26.50	28.45	27.43	28.89	26.97	1.92	0.0654	-2.51
CLAU_1379	GCN5-related N-acetyltransferase	0.020	6	1	1	6	140	27.08	27.06	27.26	26.37	26.87	26.64	26.97	1.49	0.1172	0.51
CLAU_3484	putative hydrolase	0.028	12	2	2	9	327	27.91	28.64	26.45	25.74	27.47	*20.33*	26.96	0.63	0.3917	3.15
CLAU_2004	IstB domain protein ATP-binding proteir	0.008	16	5	5	12	328	26.70	27.02	26.91	27.00	27.04	26.24	26.96	0.16	0.8053	0.12
CLAU_2607	Ig domain protein	0.009	5	2	2	12	506	27.64	26.47	26.04	26.97	28.22	26.94	26.96	0.44	0.5254	-0.66
CLAU_1419	ApbE family lipoprotein	0.014	18	5	5	8	348	26.58	26.78	26.94	26.95	27.22	27.21	26.95	1.24	0.1595	-0.36
CLAU_3360	putative kinase	0.078	8	1	1	3	191	27.37	26.56	26.12	25.81	27.56	27.33	26.95	0.12	0.8505	-0.22
CLAU_1351	3-oxoacyl-(acyl-carrier-protein) reductas	0.013	10	2	2	9	250	26.63	24.58	26.16	27.25	27.84	27.66	26.94	1.30	0.1466	-1.79
CLAU_0571	Methyltransferase type 11	0.009	13	2	2	11	207	26.98	27.74	26.88	23.06	25.94	27.28	26.93	0.63	0.3961	1.77
CLAU_3001	Nucleotidyltransferase substrate binding	0.013	8	1	1	9	128	26.62	26.86	27.20	27.09	26.99	26.81	26.93	0.14	0.8298	-0.07
CLAU_0078	Hypothetical protein	0.054	15	2	2	3	185	25.83	26.02	26.96	27.33	26.88	26.99	26.92	1.00	0.2250	-0.80
CLAU_1783	Acetaldehyde dehydrogenase (acetylatii	0.007	11	4	4	15	498	26.95	26.43	26.84	*20.50*	27.48	27.07	26.90	0.31	0.6383	1.72
CLAU_1408	Regulatory protein recX	0.014	12	3	3	9	283	26.69	27.24	27.09	25.26	26.57	27.19	26.89	0.49	0.4907	0.67
CLAU_3427	Two component transcriptional regulatc	0.021	8	2	2	6	224	25.84	26.20	26.73	27.09	27.24	27.05	26.89	1.52	0.1114	-0.87
CLAU_3283	Chromosome segregation protein SMC	0.004	13	13	13	27	1187	26.38	26.76	26.51	26.98	27.49	26.97	26.87	1.36	0.1360	-0.60
CLAU_1660	Transcriptional regulatory protein MalR	0.013	11	3	3	9	242	26.91	25.79	26.82	26.76	27.06	26.98	26.87	0.50	0.4811	-0.43
CLAU_3436	hypothetical protein	0.064	4	1	1	2	260	26.21	26.55	26.72	27.24	27.01	27.09	26.87	1.71	0.0854	-0.62
CLAU_0728	Polysaccharide deacetylase	0.005	23	5	5	17	285	28.31	27.22	26.69	27.03	26.49	26.54	26.86	0.64	0.3860	0.72
CLAU_2407	HlyD family secretion protein	0.005	14	4	4	19	424	25.85	26.72	26.23	26.98	27.24	27.12	26.85	1.49	0.1162	-0.85
CLAU_3365	putative transcriptional regulator	0.267	7	1	1	1	190	26.23	25.30	27.35	27.17	27.03	26.65	26.84	0.46	0.5111	-0.66
CLAU_3826	Peptide transport system permease con	0.013	5	2	2	8	403	26.60	27.07	27.32	24.71	26.39	27.29	26.84	0.48	0.4991	0.87
CLAU_1548	Signal transduction histidine kinase LytS	0.009	4	2	2	11	562	27.27	27.01	25.26	26.51	26.84	26.82	26.83	0.12	0.8505	-0.21
CLAU_1321	Hypothetical protein	0.012	33	6	6	12	175	26.60	26.74	26.83	26.83	27.82	27.77	26.83	1.07	0.2043	-0.75
CLAU_3514	Alcohol dehydrogenase	0.128	3	1	1	2	382	25.50	24.39	28.64	28.13	28.75	24.77	26.82	0.23	0.7213	-1.04
CLAU_1171	Transcriptional regulator MarR family	0.153	15	2	2	2	164	21.99	26.48	28.81	28.09	27.10	22.54	26.79	0.02	0.9725	-0.15
CLAU_2655	Metal dependent phosphohydrolase	0.013	7	1	1	9	161	25.65	25.65	26.62	27.27	26.94	27.05	26.78	1.52	0.1114	-1.11
CLAU_1823	OsmC family protein	0.073	5	1	1	3	147	27.04	26.55	27.21	26.63	26.87	26.67	26.77	0.42	0.5379	0.21
CLAU_1222	Hypothetical protein	0.307	3	1	1	1	216	27.65	26.10	27.09	27.29	26.44	24.95	26.77	0.37	0.5879	0.72
CLAU_0916	hypothetical protein	0.044	3	1	1	3	322	*21.44*	25.82	*22.48*	27.69	28.17	28.22	26.76	1.64	0.0951	-4.78
CLAU_0721	Isoaspartyl dipeptidase	0.012	8	3	3	10	388	26.77	26.81	26.66	26.73	26.70	27.00	26.75	0.24	0.7144	-0.06
CLAU_0839	NADPH/dihydrolipoyl dehydrogenase	0.009	6	3	3	13	665	25.84	25.04	27.19	26.70	26.79	27.05	26.75	0.58	0.4287	-0.82
CLAU_1178	Diguanylate cyclase	0.013	6	2	2	10	354	26.54	27.42	26.39	26.32	26.95	26.94	26.74	0.04	0.9477	0.05
CLAU_0745	hypothetical protein	0.016	17	3	3	7	201	26.82	28.33	24.54	24.51	27.37	26.66	26.74	0.10	0.8773	0.38
CLAU_2816	TRNA modifying enzyme	0.014	11	4	4	8	463	27.01	26.80	26.81	*22.61*	26.58	26.65	26.73	0.52	0.4657	1.59
CLAU_0583	2-(5''-triphosphoribosyl)-3'-dephosphoc	0.199	6	1	1	1	290	27.49	26.80	27.06	24.39	26.25	26.65	26.73	0.87	0.2766	1.35
CLAU_1607	SNF2-related protein	0.043	4	3	3	5	898	26.48	26.84	26.60	*21.30*	27.43	27.14	26.72	0.27	0.6759	1.35
CLAU_2822	7TM receptor with intracellular metal de	0.065	2	1	1	2	701	26.90	26.19	26.74	26.70	26.43	26.79	26.72	0.04	0.9466	-0.03
CLAU_2526	Capsular biosynthesis protein	0.004	34	5	5	22	227	25.89	27.15	27.23	27.03	26.38	26.16	26.71	0.17	0.7850	0.23
CLAU_1787	Hypothetical protein	0.013	2	1	1	10	362	27.73	27.19	26.64	26.62	26.62	26.77	26.71	0.74	0.3352	0.52
CLAU_2169	Uracil-DNA glycosylase superfamily	0.013	10	2	2	10	198	26.46	26.66	24.05	26.80	26.75	26.91	26.71	0.58	0.4249	-1.10
CLAU_0837	2,4-dienoyl-CoA reductase (NADPH)	0.023	1	1	1	5	640	27.18	26.83	26.79	26.44	26.58	26.62	26.71	1.34	0.1415	0.39
CLAU_2249	Spore coat protein CotS-like protein	0.037	4	1	1	6	255	26.92	27.01	26.05	26.36	26.52	26.87	26.70	0.08	0.8983	0.08
CLAU_2748	hypothetical protein	0.007	4	1	1	13	238	26.47	26.60	26.85	26.68	27.01	26.70	26.69	0.43	0.5314	-0.16
CLAU_1088	Precorrin-2 dehydrogenase	0.013	11	2	2	9	220	27.29	27.53	27.12	26.25	25.18	25.53	26.69	2.10	0.0537	1.66
CLAU_3822	TrkA-C domain protein	0.013	17	3	3	9	210	27.99	27.69	27.01	26.36	26.13	25.69	26.69	1.90	0.0683	1.50

CLAU_1764	Alcohol dehydrogenase	0.012	23	2	2	10	119	27.59	24.99	26.66	26.47	26.69	27.89	26.68		0.28	0.6700	-0.60
CLAU_1788	Hypothetical protein	0.016	11	1	1	7	87	26.59	26.76	26.96	26.42	25.50	26.83	26.68		0.57	0.4349	0.52
CLAU_2263	Monogalactosyldiacylglycerol synthase	0.007	11	4	4	15	412	25.39	25.77	23.09	27.56	27.95	27.62	26.67		1.60	0.1005	-2.96
CLAU_1434	Xylose isomerase domain-containing prc	0.013	8	2	2	8	261	27.37	26.70	26.12	26.41	26.63	27.45	26.67		0.07	0.9071	-0.10
CLAU_1512	Glutamyl-tRNA(Gln) amidotransferase sı	0.084	7	3	3	3	485	27.05	25.50	26.97	26.35	26.08	27.44	26.66		0.06	0.9210	-0.12
CLAU_0115	Hydrogenase large subunit domain prot	0.030	1	1	1	7	638	27.84	27.46	28.12	24.95	24.05	25.85	26.66	+	2.17	0.0500	2.86
CLAU_1879	Protein translocase subunit SecY	0.034	10	3	3	5	426	26.26	26.99	24.09	26.62	26.94	26.68	26.65		0.48	0.4994	-0.97
CLAU_1868	ECF-type transporter transmembrane pı	0.017	10	2	2	7	269	26.75	26.91	26.59	26.51	26.69	26.51	26.64		0.75	0.3324	0.18
CLAU_1750	Osmosensitive K(+) channel signal transı	0.012	5	5	5	11	911	25.95	26.74	26.24	26.86	26.53	27.32	26.64		0.85	0.2859	-0.59
CLAU_2442	Hemolysin D related protein	0.018	18	3	3	6	217	26.63	26.33	26.54	26.64	26.79	26.78	26.64		1.10	0.1951	-0.24
CLAU_1501	Cell wall binding repeat 2-containing prc	0.033	4	2	2	4	509	27.28	26.04	26.28	25.91	27.17	26.98	26.63		0.10	0.8754	-0.15
CLAU_3965	Hypothetical protein	0.013	28	3	3	9	129	26.81	26.44	26.86	*20.04*	25.39	27.48	26.63		0.47	0.5086	2.40
CLAU_1135	Fe-S oxidoreductase	0.019	6	2	2	7	343	26.83	26.35	26.53	25.51	26.70	26.74	26.62		0.23	0.7170	0.25
CLAU_1343	Monosaccharide-transporting ATPase	0.054	5	2	2	4	509	26.12	25.96	27.09	26.67	26.55	27.00	26.61		0.39	0.5658	-0.35
CLAU_2760	Selenide/water dikinase	0.013	9	2	2	8	311	26.30	26.74	26.46	26.49	26.71	26.84	26.60		0.48	0.5023	-0.18
CLAU_3237	Multi-sensor signal transduction histidin	0.016	8	3	3	7	577	26.65	26.03	26.59	26.39	26.59	27.05	26.59		0.38	0.5727	-0.25
CLAU_2815	RNA methyltransferase	0.033	10	2	2	4	251	26.72	26.92	25.40	26.80	26.30	26.45	26.59		0.12	0.8461	-0.17
CLAU_0686	Phosphoglycerate mutase	0.014	8	2	2	8	237	26.55	26.47	*20.94*	27.05	26.86	26.61	26.58		0.52	0.4721	-2.19
CLAU_3907	Ribose/xylose/arabinose/galactoside AB	0.028	4	1	1	9	347	27.66	29.08	27.22	25.93	25.77	23.09	26.58		1.33	0.1435	3.06
CLAU_2086	Helix-turn-helix domain protein	0.016	10	2	2	7	187	26.64	26.31	26.50	26.48	26.81	26.65	26.57		0.53	0.4578	-0.16
CLAU_2945	Transcriptional regulator TrmB family	0.024	9	3	3	6	267	25.86	25.83	25.90	27.34	28.03	27.24	26.57	+	2.59	0.0340	-1.67
CLAU_3134	Tyrosine recombinase xerC	0.143	3	1	1	1	292	25.60	23.38	26.70	26.75	26.70	26.44	26.57		0.65	0.3839	-1.40
CLAU_3519	ABC-2 family transporter protein	0.014	12	4	4	8	394	26.74	27.07	26.10	25.25	26.46	26.67	26.57		0.41	0.5483	0.51
CLAU_2154	4Fe-4S ferredoxin iron-sulpur binding	0.044	8	2	2	3	260	25.67	26.36	26.58	26.54	26.65	26.56	26.55		0.62	0.4009	-0.38
CLAU_0324	putative transcriptional regulator PucR f	0.145	6	2	2	2	518	26.66	23.14	26.43	23.18	27.69	27.35	26.55		0.13	0.8374	-0.66
CLAU_1429	GTP-binding protein engB	0.007	34	6	6	13	196	27.18	26.51	26.04	26.56	26.86	26.12	26.54		0.06	0.9297	0.06
CLAU_0180	SOS-response transcriptional repressor I	0.019	3	1	1	6	205	26.51	26.56	26.53	*21.19*	26.61	26.37	26.52		0.44	0.5285	1.81
CLAU_3361	Peptide deformylase	0.013	16	1	1	8	150	26.27	26.21	26.45	26.56	26.77	27.27	26.51		1.18	0.1731	-0.56
CLAU_0251	Bacterial extracellular solute-binding prc	0.009	12	4	4	12	358	26.24	26.21	26.76	26.48	26.52	26.63	26.50		0.31	0.6383	-0.14
CLAU_2382	Alcohol dehydrogenase	0.067	4	1	1	4	386	26.46	27.28	24.99	27.17	26.54	*21.57*	26.50		0.24	0.7112	1.15
CLAU_2518	Outer membrane efflux protein	0.012	14	4	4	12	378	26.66	23.48	25.54	27.76	28.28	26.33	26.50		0.95	0.2414	-2.23
CLAU_0572	ATP-dependent DNA helicase RecQ	0.009	12	7	7	14	722	24.42	26.68	26.57	26.38	27.20	24.92	26.48		0.10	0.8754	-0.28
CLAU_3310	Ribosome maturation factor RimP	0.012	19	3	3	10	154	26.35	27.09	27.13	26.39	*22.87*	26.56	26.48		0.57	0.4307	1.58
CLAU_1303	RRNA methyltransferase (SpoU)	0.315	3	1	1	1	260	*21.57*	*21.64*	26.65	26.82	27.23	26.29	26.47		0.96	0.2378	-3.49
CLAU_0364	Glyoxalase-like protein	0.023	19	2	2	5	121	26.36	25.09	26.68	26.51	26.41	26.69	26.46		0.43	0.5343	-0.49
CLAU_2731	Acetyltransferase (GNAT) domain	0.028	20	3	3	5	165	26.70	26.34	27.43	26.19	25.93	26.57	26.46		0.73	0.3392	0.59
CLAU_3766	Hypothetical protein	0.036	7	3	3	4	497	25.57	26.39	26.11	26.51	27.08	27.15	26.45		1.32	0.1439	-0.89
CLAU_3522	Hypothetical protein	0.139	6	1	1	2	213	26.48	27.35	26.42	24.87	27.05	24.46	26.45		0.68	0.3641	1.29
CLAU_0072	hypothetical protein	0.009	13	3	3	11	295	26.55	26.41	26.28	26.21	26.47	26.62	26.44		0.05	0.9375	-0.02
CLAU_0892	UDP-glucuronosyl/UDP-glucosyltransfer	0.009	8	4	4	13	442	25.97	26.19	26.12	26.79	26.81	26.63	26.41	+	2.78	0.0283	-0.65
CLAU_1735	Transcriptional regulator LysR family	0.018	6	2	2	6	299	26.20	26.87	26.78	26.59	26.22	25.71	26.41		0.60	0.4124	0.44
CLAU_2050	MazG-like domain-containing protein	0.175	10	1	1	1	105	26.16	26.36	26.42	26.36	26.55	26.59	26.39		0.82	0.3010	-0.19
CLAU_1274	Hypothetical protein	0.023	3	1	1	5	429	23.96	25.58	26.53	27.08	27.09	26.24	26.39		0.84	0.2894	-1.45
CLAU_2515	ABC-2 family transporter protein	0.171	4	1	1	1	250	25.68	26.62	25.86	27.39	26.13	26.72	26.38		0.68	0.3666	-0.69
CLAU_2536	Hypothetical protein	0.018	3	2	2	8	437	26.47	26.26	25.24	25.12	26.78	26.80	26.37		0.13	0.8371	-0.24
CLAU_0896	Pyruvate ferredoxin/flavodoxin oxidoreı	0.016	6	5	4	12	1174	24.27	24.34	26.31	26.86	26.41	26.53	26.36		1.12	0.1882	-1.63

CLAU_1467	Aminotransferase class IV	0.033	13	3	3	6	251	27.02	25.83	26.05	26.48	26.48	26.22	26.35		0.09	0.8903	-0.09
CLAU_3660	putative transcriptional regulator	0.189	17	1	1	1	70	26.07	26.43	26.27	26.22	26.83	26.69	26.35		0.70	0.3586	-0.32
CLAU_3491	Diguanylate cyclase/phosphodiesterase	0.033	3	2	2	4	587	26.19	26.47	26.38	26.56	26.31	26.29	26.35		0.12	0.8479	-0.04
CLAU_3147	Electron transport complex subunit E	0.033	3	1	1	4	213	24.83	26.33	26.36	27.16	28.56	25.82	26.35		0.64	0.3841	-1.34
CLAU_0269	AroM family protein	0.016	10	2	2	7	224	27.11	26.14	26.96	25.88	26.13	26.54	26.34		0.71	0.3545	0.55
CLAU_0377	Transcriptional regulator MarR family	0.023	6	1	1	5	152	26.64	26.19	26.21	26.19	26.47	26.61	26.34		0.15	0.8154	-0.08
CLAU_0913	Hypothetical protein	0.014	34	2	2	8	35	28.77	28.73	29.00	*22.59*	23.23	23.93	26.33	+	3.84	0.0107	5.58
CLAU_0903	Hypothetical protein	0.013	7	2	2	12	177	26.62	26.26	26.41	21.82	26.39	25.80	26.33		0.54	0.4536	1.76
CLAU_3791	Hypothetical protein	0.033	6	2	2	4	390	25.97	24.87	24.87	26.67	27.29	26.92	26.32		1.87	0.0708	-1.72
CLAU_0971	Metal dependent phosphohydrolase	0.013	9	3	3	10	356	26.92	27.19	27.21	25.51	24.08	25.69	26.31		1.75	0.0809	2.01
CLAU_3385	putative sodium:solute symporter	0.009	3	1	1	12	469	26.57	27.12	27.99	23.32	26.01	23.14	26.29		1.41	0.1284	3.07
CLAU_0536	Copper-translocating P-type ATPase	0.016	4	3	3	8	816	25.93	26.32	26.07	26.73	26.30	26.26	26.28		0.79	0.3132	-0.32
CLAU_2196	Chemotaxis protein MotA	0.016	11	2	2	7	264	25.52	27.04	22.82	27.69	*21.94*	27.88	26.28		0.11	0.8619	-0.71
CLAU_2469	hypothetical protein	0.007	16	5	5	15	424	27.75	27.36	27.61	25.16	*20.43*	25.19	26.28		1.18	0.1722	3.98
CLAU_2161	Metal-dependent phosphohydrolase HD	0.033	16	2	2	4	164	26.20	26.48	26.89	26.32	26.18	26.23	26.28		0.61	0.4030	0.28
CLAU_1095	GntR family transcriptional regulator	0.023	3	2	2	5	482	26.34	25.93	25.88	26.20	26.58	26.81	26.27		0.98	0.2306	-0.48
CLAU_1687	GerA spore germination protein	0.269	2	1	1	1	499	26.88	*22.09*	22.12	27.42	25.66	27.76	26.27		0.88	0.2724	-3.25
CLAU_0840	Shikimate dehydrogenase	0.033	3	1	1	4	291	26.42	26.37	26.06	26.06	26.29	26.24	26.27		0.26	0.6849	0.09
CLAU_0640	Chloramphenicol O-acetyltransferase-lik	0.033	5	1	1	4	225	25.88	25.59	24.14	26.63	26.82	26.63	26.26		1.29	0.1505	-1.49
CLAU_0350	AraC family transcriptional regulator wit	0.068	3	1	1	4	415	25.74	27.38	26.69	27.41	25.20	24.83	26.22		0.35	0.6029	0.79
CLAU_0608	Transcriptional regulator TetR family	0.074	10	2	2	2	213	26.23	26.19	25.97	26.36	26.13	26.28	26.21		0.53	0.4605	-0.13
CLAU_3262	Ribosome biogenesis GTPase RsgA	0.013	7	2	2	8	287	26.52	27.01	26.45	25.66	25.53	25.96	26.21		1.91	0.0665	0.94
CLAU_2535	Glycosyl transferase family 4	0.013	11	4	4	9	370	25.65	25.91	26.02	27.15	27.11	26.38	26.20		1.69	0.0862	-1.02
CLAU_3730	ABC-type transporter periplasmic bindin	0.028	9	3	3	5	361	26.51	24.57	24.70	25.88	29.86	29.29	26.20		1.04	0.2150	-3.08
CLAU_2740	Septum site-determining protein minC	0.013	10	2	2	9	210	25.90	26.14	26.14	26.24	27.52	26.37	26.19		0.72	0.3481	-0.65
CLAU_0431	Xanthine permease	0.013	6	3	3	8	451	26.02	24.92	*21.96*	26.46	26.45	26.36	26.19		0.81	0.3037	-2.12
CLAU_0270	hypothetical protein	0.016	8	2	2	7	310	27.14	27.72	27.11	25.25	24.60	*21.71*	26.18		1.46	0.1208	3.47
CLAU_2101	Hypothetical protein	0.207	7	1	1	1	121	23.95	*21.06*	*22.03*	28.40	28.89	28.85	26.18	+	2.75	0.0285	-6.36
CLAU_2055	MscS Mechanosensitive ion channel	0.016	14	3	3	7	296	26.70	24.80	23.39	26.50	26.04	26.29	26.17		0.61	0.4070	-1.31
CLAU_3317	TRNA pseudouridine synthase B	0.056	9	2	2	4	293	24.36	25.68	27.57	27.83	26.64	22.55	26.16		0.04	0.9538	0.20
CLAU_1277	Glycosyl transferase group 1	0.044	3	2	2	3	393	26.35	25.59	25.95	24.15	26.64	26.38	26.15		0.11	0.8687	0.24
CLAU_0294	3,4-dihydroxy-2-butanone 4-phosphate	0.058	3	1	1	4	401	30.79	26.42	25.96	25.00	26.32	*20.04*	26.14		0.74	0.3390	3.94
CLAU_3632	Methyl-accepting chemotaxis sensory tr	0.013	22	3	3	13	177	26.67	25.70	25.98	25.00	28.02	26.28	26.13		0.13	0.8451	-0.32
CLAU_3351	putative glycosyl hydrolase	0.016	9	4	4	7	658	26.60	23.66	26.07	22.71	26.76	26.18	26.13		0.05	0.9350	0.23
CLAU_0135	Uroporphyrinogen decarboxylase (URO-	0.044	6	2	2	3	369	24.87	24.62	26.03	26.20	26.21	26.66	26.12		1.21	0.1655	-1.18
CLAU_1422	Alpha-ribazole phosphatase	0.017	4	1	1	7	197	25.32	25.64	26.10	26.28	26.38	26.12	26.11		1.13	0.1866	-0.57
CLAU_0212	HI0933 family protein	0.013	7	2	2	9	408	24.85	25.16	25.64	26.57	29.06	29.91	26.11		1.49	0.1173	-3.30
CLAU_1228	Coproporphyrinogen III oxidase	0.014	7	3	3	8	482	26.23	26.54	26.72	25.04	25.96	25.10	26.10		1.57	0.1039	1.13
CLAU_2363	PDZ/DHR/GLGF domain protein	0.044	2	1	1	3	433	25.90	26.16	25.96	26.03	26.42	26.30	26.10		0.81	0.3052	-0.24
CLAU_0443	Guanine deaminase	0.073	3	1	1	3	427	26.00	26.78	*22.19*	24.89	26.50	26.19	26.10		0.23	0.7229	-0.87
CLAU_3794	HAD-superfamily hydrolase subfamily IA	0.020	11	2	2	6	215	24.65	25.68	26.85	27.25	26.48	25.47	26.08		0.34	0.6105	-0.67
CLAU_3619	Alcohol dehydrogenase	0.064	7	2	2	2	364	25.90	26.06	25.32	26.26	26.45	26.10	26.08		0.97	0.2340	-0.51
CLAU_2177	Transcriptional regulator GntR family	0.009	10	3	3	13	361	26.09	25.50	26.71	25.46	26.06	26.17	26.08		0.19	0.7683	0.20
CLAU_1905	Ribosomal protein L7Ae/L30e/S12e/Gad	0.015	21	1	1	7	80	25.95	25.91	26.63	25.60	26.42	26.20	26.08		0.10	0.8815	0.09
CLAU_0704	Hypothetical protein	0.141	3	1	1	2	283	26.66	*21.75*	25.77	27.37	26.27	25.85	26.06		0.49	0.4923	-1.77
CLAU_3515	Transcriptional regulator PucR family	0.141	3	1	1	2	522	26.36	26.43	23.24	25.70	26.08	26.04	26.06		0.22	0.7305	-0.60

CLAU_1722	Hypothetical protein	0.013	18	2	2	10	131	25.60	24.67	24.05	26.49	26.49	28.94	26.05		1.27	0.1534	-2.53
CLAU_1265	Glycosyl transferase	0.016	7	2	2	7	367	23.24	25.40	25.43	27.67	26.96	26.65	26.04		1.42	0.1259	-2.40
CLAU_2893	NMT1/THI5-like protein	0.013	14	3	3	8	334	26.72	26.10	25.95	23.86	*21.25*	26.19	26.03		0.80	0.3108	2.49
CLAU_2158	Phosphoglycerate dehydrogenase	0.013	10	3	3	9	308	25.77	25.83	25.96	26.34	27.10	26.09	26.03		1.00	0.2250	-0.66
CLAU_2097	Hypothetical protein	0.033	18	1	1	4	82	26.45	*21.70*	26.30	22.96	26.18	25.87	26.03		0.03	0.9562	-0.19
CLAU_2115	Hypothetical protein	0.145	8	1	1	1	101	25.37	25.90	26.57	26.13	25.85	26.26	26.02		0.13	0.8371	-0.13
CLAU_0641	Rubrerythrin	0.020	18	3	3	7	184	25.64	24.87	25.86	26.13	26.41	26.38	26.00		1.27	0.1534	-0.85
CLAU_0438	Selenium-dependent molybdopterin oxi	0.019	4	3	3	6	854	25.78	25.87	25.44	26.10	26.13	26.60	25.99		1.31	0.1466	-0.58
CLAU_0457	Glycine cleavage system aminomethyltr	0.011	10	3	3	11	368	27.98	27.64	26.20	25.57	25.76	25.47	25.98		1.41	0.1281	1.67
CLAU_0815	Signal transduction histidine kinase regu	0.013	5	2	2	10	422	25.84	26.32	25.82	25.38	26.12	26.17	25.98		0.12	0.8461	0.10
CLAU_1773	Microcompartments protein	0.045	3	1	1	4	248	25.30	26.79	26.65	27.18	25.02	25.09	25.98		0.22	0.7289	0.48
CLAU_2329	Quinolinate phosphoribosyl transferase	0.044	7	2	2	3	269	25.83	25.83	25.66	27.04	26.10	27.00	25.97		1.40	0.1286	-0.94
CLAU_1438	Dihydroorotate dehydrogenase electron	0.064	3	1	1	2	250	25.28	25.50	26.57	26.41	26.51	25.14	25.96		0.15	0.8156	-0.24
CLAU_0613	Signal transduction histidine kinase cont	0.103	2	1	1	2	487	22.11	*20.05*	25.57	26.33	26.48	26.98	25.95		1.17	0.1759	-4.02
CLAU_2499	Phosphodiesterase MJ0936 family	0.014	19	3	3	8	230	25.16	25.94	26.46	26.37	25.95	25.67	25.95		0.12	0.8479	-0.14
CLAU_1947	RNA-binding S4 domain protein	0.044	15	1	1	3	82	26.30	27.55	22.79	*21.08*	27.80	25.58	25.94		0.11	0.8661	0.73
CLAU_0474	Hypothetical protein	0.013	16	3	3	8	266	28.63	28.70	27.68	*19.80*	*24.19*	*21.66*	25.94		2.10	0.0537	6.45
CLAU_2825	GTPase Era	0.044	12	3	3	3	293	26.08	26.89	27.84	25.79	24.66	24.92	25.94		1.38	0.1328	1.81
CLAU_3160	Transcriptional regulator MerR family	0.169	8	1	1	1	133	24.63	22.56	23.48	27.36	27.24	27.32	25.94	+	2.48	0.0381	-3.75
CLAU_2516	ABC-type transporter related protein	0.018	9	2	2	7	241	26.14	24.89	25.91	24.23	25.95	26.49	25.93		0.04	0.9510	0.09
CLAU_2130	Cof-like hydrolase	0.029	8	2	2	5	274	26.36	25.79	*21.49*	25.71	26.05	27.36	25.92		0.49	0.4895	-1.83
CLAU_1256	Anti-sigma F factor	0.043	13	1	1	3	142	25.09	26.08	26.44	25.74	26.15	25.57	25.91		0.04	0.9513	0.05
CLAU_1509	ABC-type transporter periplasmic subun	0.303	4	1	1	1	538	25.88	26.29	25.93	24.99	25.94	25.61	25.91		0.78	0.3180	0.52
CLAU_0545	Uroporphyrinogen-III decarboxylase-like	0.076	2	1	1	3	370	25.33	23.55	26.26	26.24	26.26	25.56	25.90		0.52	0.4719	-0.97
CLAU_1001	Uroporphyrinogen decarboxylase (URO-	0.160	3	1	1	2	351	27.93	26.51	25.29	*21.83*	26.54	*22.29*	25.90		0.83	0.2915	3.02
CLAU_3424	LL-diaminopimelate aminotransferase	0.184	2	1	1	1	404	27.24	27.00	*21.67*	24.07	25.70	26.10	25.90		0.00	0.9990	0.01
CLAU_0675	Hypothetical protein	0.171	13	1	1	1	82	26.26	25.78	26.10	25.62	26.01	24.19	25.90		0.61	0.4077	0.77
CLAU_0307	ABC-type metal ion transporter periplas	0.101	9	2	2	2	310	27.41	25.61	*21.26*	23.73	26.18	26.89	25.90		0.15	0.8131	-0.84
CLAU_0556	Response regulators consisting of a Che'	0.023	24	3	3	5	227	25.52	25.78	*21.74*	26.21	26.17	26.00	25.89		0.61	0.4051	-1.78
CLAU_1426	WYL domain-containing protein	0.033	11	3	3	4	317	26.35	25.41	26.84	26.37	*19.51*	*22.89*	25.88		0.74	0.3354	3.28
CLAU_1977	Hypothetical protein	0.016	6	1	1	7	182	26.29	25.94	25.50	25.54	26.06	25.81	25.88		0.14	0.8194	0.11
CLAU_2035	L-rhamnose isomerase	0.013	7	3	3	8	419	25.85	25.88	25.04	23.66	26.91	26.72	25.87		0.05	0.9297	-0.17
CLAU_0371	Transcriptional regulator containing GAF	0.033	2	1	1	4	646	24.15	*21.89*	25.80	25.91	26.00	25.92	25.86		0.82	0.3011	-2.00
CLAU_1967	HAD-superfamily hydrolase subfamily IA	0.259	3	1	1	1	217	26.02	*22.34*	25.69	25.36	26.13	26.09	25.86		0.42	0.5436	-1.18
CLAU_3062	Ribosomal RNA small subunit methyltra	0.018	15	3	3	6	309	24.29	26.71	26.83	26.62	24.48	25.06	25.84		0.21	0.7483	0.56
CLAU_3313	putative ribosomal protein	0.017	18	2	2	7	105	26.04	25.86	25.81	*21.18*	26.08	25.19	25.84		0.51	0.4763	1.75
CLAU_3746	ABC-type transporter periplasmic bindin	0.007	26	6	6	14	363	25.79	*21.84*	27.05	24.86	25.87	26.57	25.83		0.20	0.7490	-0.87
CLAU_2664	hypothetical protein	0.017	6	6	6	8	1189	25.77	25.93	25.89	25.89	23.47	25.58	25.83		0.51	0.4773	0.88
CLAU_0559	Transcriptional regulator GntR family	0.045	9	1	1	3	122	25.57	26.09	24.75	*22.98*	26.64	26.19	25.83		0.06	0.9291	0.20
CLAU_3095	Signal transduction histidine kinase regu	0.143	3	1	1	1	396	25.61	26.14	25.68	25.38	25.96	26.02	25.82		0.03	0.9603	0.02
CLAU_3478	VanW family protein	0.187	2	1	1	1	437	25.78	25.89	25.64	25.48	25.84	25.87	25.81		0.10	0.8763	0.04
CLAU_3080	Pseudouridine synthase	0.019	6	2	2	6	302	25.69	25.65	25.79	25.92	25.82	26.01	25.81		1.40	0.1286	-0.21
CLAU_2354	ADP/ATP-dependent (S)-NAD(P)H-hydra	0.027	14	6	6	6	499	25.61	25.11	32.26	25.70	25.91	26.31	25.81		0.30	0.6483	1.69
CLAU_0320	Cell wall binding repeat 2-containing prc	0.037	3	3	2	6	969	25.58	28.82	28.30	25.25	25.00	26.03	25.81		0.95	0.2404	2.14
CLAU_0833	Hypothetical protein	0.016	5	1	1	7	305	25.19	26.64	26.73	25.72	25.88	25.72	25.80		0.34	0.6105	0.41
CLAU_3258	Sun protein	0.009	7	3	3	12	443	25.62	25.99	26.28	25.43	25.97	25.58	25.80		0.54	0.4564	0.30

CLAU_3035	Flagellar hook-length control protein-lik	0.037	5	2	2	4	513	24.87	24.63	22.67	26.89	27.09	26.71	25.79		1.80	0.0765	-2.84
CLAU_2557	Asparagine synthase	0.254	2	1	1	1	598	25.18	25.89	26.40	25.66	25.13	25.91	25.78		0.24	0.7106	0.26
CLAU_3346	putative transcriptional regulator	0.035	9	1	1	4	123	25.94	25.87	26.12	25.57	25.67	25.36	25.77		1.70	0.0854	0.44
CLAU_0236	Electron transfer flavoprotein	0.263	4	1	1	1	402	26.33	25.20	26.91	*20.01*	*20.56*	27.16	25.77		0.69	0.3604	3.57
CLAU_2529	hypothetical protein	0.064	17	1	1	2	75	*21.15*	*20.41*	26.11	27.13	27.36	25.37	25.74		1.01	0.2227	-4.06
CLAU_3843	Sugar kinase ROK family	0.064	4	2	2	2	392	24.73	24.57	25.78	25.68	26.19	25.87	25.73		1.02	0.2187	-0.89
CLAU_3143	Positive regulator of sigma E RseC/MucC	0.053	22	2	2	3	138	25.06	25.58	23.53	26.23	25.88	26.23	25.73		1.04	0.2149	-1.39
CLAU_1332	Phosphoglycerate dehydrogenase	0.026	6	2	2	5	339	26.23	25.50	25.69	25.29	25.75	26.39	25.72		0.00	0.9990	0.00
CLAU_0917	Redox-active disulfide protein 2	0.016	11	1	1	7	79	25.72	25.71	24.56	24.89	26.56	26.51	25.72		0.42	0.5435	-0.66
CLAU_0899	AAA-ATPase	0.028	2	1	1	5	249	25.63	24.79	25.76	*22.15*	26.45	26.50	25.70		0.09	0.8926	0.36
CLAU_2583	Beta-lactamase domain protein	0.044	10	3	3	4	308	26.12	23.75	23.30	27.69	25.27	26.86	25.70		0.92	0.2545	-2.22
CLAU_2215	Hypothetical protein	0.016	15	1	1	7	61	23.42	*21.57*	*21.64*	27.94	28.04	27.98	25.68	+	3.17	0.0222	-5.77
CLAU_2229	Ferredoxin	0.022	24	1	1	5	62	25.60	25.74	25.31	25.16	26.05	25.91	25.67		0.20	0.7580	-0.16
CLAU_0931	TRNA (guanine-N(7)-)-methyltransferase	0.274	5	1	1	1	211	24.55	22.24	*21.02*	26.88	27.04	26.78	25.67		1.84	0.0721	-4.30
CLAU_2033	Major facilitator superfamily MFS_1	0.019	2	1	1	6	421	23.19	24.80	24.95	26.43	26.36	26.52	25.66		1.70	0.0854	-2.12
CLAU_3465	UV-endonuclease UvdE	0.143	4	1	1	1	418	25.03	26.76	25.26	25.74	25.70	25.61	25.66		0.00	1.0000	0.00
CLAU_1937	TIM-barrel protein nifR3 family	0.013	11	3	3	8	320	25.47	25.83	27.00	26.59	25.38	24.44	25.65		0.34	0.6136	0.63
CLAU_0977	Glyoxylate reductase (NADP(+))	0.013	8	2	2	8	322	25.11	25.11	24.68	26.73	26.78	26.19	25.65	+	2.60	0.0339	-1.60
CLAU_3382	Hypothetical protein	0.183	7	1	1	1	244	25.81	25.84	25.81	25.25	25.44	25.49	25.65	+	2.35	0.0407	0.43
CLAU_0267	Peptidase M18 aminopeptidase I	0.045	3	2	2	3	466	26.78	26.07	25.22	26.34	23.21	22.78	25.65		0.72	0.3444	1.91
CLAU_0235	D-lactate dehydrogenase (cytochrome)	0.044	5	2	2	3	470	24.48	25.73	25.56	25.72	26.38	24.55	25.64		0.17	0.7930	-0.29
CLAU_2349	UPF0313 protein	0.016	3	2	2	7	614	24.90	25.86	25.98	25.39	25.87	24.96	25.63		0.15	0.8150	0.17
CLAU_3962	4Fe-4S ferredoxin iron-sulpur binding	0.013	9	2	2	10	235	26.25	25.45	24.72	26.92	25.55	25.69	25.62		0.40	0.5619	-0.58
CLAU_0237	Electron transfer flavoprotein	0.050	10	2	2	3	262	25.61	25.06	25.49	25.57	26.33	25.80	25.59		0.85	0.2845	-0.51
CLAU_0800	hypothetical protein	0.044	28	3	3	3	127	25.23	26.47	25.93	*20.64*	27.10	24.23	25.58		0.42	0.5390	1.89
CLAU_1407	Membrane-bound protein with a transg	0.054	5	2	2	3	395	26.62	*22.21*	28.05	24.53	24.14	26.67	25.58		0.10	0.8807	0.51
CLAU_0751	Sua5/YciO/YrdC/YwlC family protein	0.023	3	1	1	5	349	25.66	25.71	23.70	25.38	25.93	25.48	25.57		0.35	0.6045	-0.57
CLAU_1676	Penicillinase repressor	0.034	16	2	2	4	127	24.23	25.22	26.58	*20.85*	25.92	26.14	25.57		0.22	0.7319	1.04
CLAU_3341	NUDIX hydrolase	0.044	5	1	1	3	202	23.13	24.58	25.95	25.53	25.60	25.96	25.57		0.62	0.3975	-1.14
CLAU_3768	4Fe-4S ferredoxin iron-sulfur binding	0.321	5	1	1	1	234	*22.03*	22.06	25.47	27.44	25.61	25.75	25.54		1.13	0.1867	-3.08
CLAU_0445	Alpha-ribazole phosphatase	0.033	14	2	2	4	195	26.46	27.94	28.51	*20.62*	24.40	24.59	25.53		1.44	0.1234	4.43
CLAU_0774	Response regulator	0.045	3	1	1	3	228	23.31	24.95	25.67	26.09	25.68	25.36	25.52		0.66	0.3746	-1.07
CLAU_0789	Glucarate dehydratase	0.011	11	3	3	11	448	26.71	25.34	25.61	22.32	26.31	25.38	25.50		0.40	0.5537	1.22
CLAU_0625	6-phosphofructokinase	0.019	6	2	2	8	316	25.81	25.16	26.01	25.11	25.05	26.29	25.49		0.14	0.8330	0.18
CLAU_0797	Adenosine deaminase	0.075	6	2	2	2	341	25.83	25.88	24.75	23.88	25.80	25.16	25.48		0.33	0.6197	0.54
CLAU_0732	putative transcriptional regulator MerR	0.315	3	1	1	1	399	22.74	25.48	*21.89*	25.48	26.02	25.82	25.48		1.03	0.2171	-2.40
CLAU_3593	Macrocin-O-methyltransferase	0.029	7	2	2	5	281	25.60	25.46	25.29	25.18	25.64	25.49	25.48		0.03	0.9629	0.01
CLAU_0398	vnfN-like nitrogenase	0.340	5	1	1	1	506	25.67	25.24	28.31	*20.32*	*20.80*	25.85	25.46		0.95	0.2414	4.08
CLAU_2088	Phage regulatory protein Rha family	0.180	3	1	1	1	264	25.13	*21.30*	*20.20*	25.76	26.14	25.86	25.45		1.16	0.1760	-3.71
CLAU_2025	Hypothetical protein	0.018	2	1	1	6	459	*20.99*	*21.76*	*21.36*	29.36	29.29	29.11	25.43	+	5.34	0.0030	-7.88
CLAU_3154	Cof-like hydrolase	0.054	8	2	2	3	273	24.29	23.55	25.70	25.78	25.13	25.97	25.42		0.75	0.3318	-1.11
CLAU_1684	Peptidoglycan glycosyltransferase	0.013	9	4	4	11	592	24.20	25.72	25.22	24.39	25.66	25.60	25.41		0.10	0.8754	-0.17
CLAU_2508	Nitrogenase cofactor biosynthesis prote	0.218	1	1	1	1	893	24.12	24.28	27.59	27.83	26.54	*23.30*	25.41		0.12	0.8566	-0.56
CLAU_0138	1-phosphofructokinase	0.007	23	6	6	14	301	29.01	27.23	24.22	24.67	25.38	25.43	25.41		0.51	0.4742	1.66
CLAU_1153	Transcriptional regulator PadR-like famil	0.019	8	1	1	6	113	25.38	25.42	26.01	25.92	25.39	25.28	25.41		0.09	0.8849	0.07
CLAU_3308	Membrane-associated metalloprotease	0.029	15	4	4	5	336	25.38	25.42	26.44	25.58	24.94	25.20	25.40		0.57	0.4307	0.51

CLAU_2799	hypothetical protein	0.033	5	2	2	4	368	22.69	24.47	*21.57*	26.30	27.18	27.05	25.39		1.94	0.0647	-3.93
CLAU_0040	Two component transcriptional regulato	0.102	6	1	1	2	230	25.06	22.87	*20.67*	25.70	27.15	26.41	25.38		1.25	0.1586	-3.55
CLAU_2852	ABC-type transporter ATP-binding prote	0.021	8	4	4	6	635	26.17	25.18	25.17	25.21	25.53	26.63	25.37		0.20	0.7546	-0.28
CLAU_3841	Aldose 1-epimerase	0.054	9	2	2	3	353	25.34	25.06	26.37	25.32	25.13	25.37	25.33		0.32	0.6282	0.32
CLAU_1768	Two component transcriptional regulato	0.011	20	4	4	11	256	25.72	24.96	24.68	25.50	25.59	25.09	25.30		0.32	0.6259	-0.27
CLAU_1954	Peptidyl-tRNA hydrolase	0.013	13	2	2	8	191	25.40	25.41	23.44	23.45	25.73	25.18	25.29		0.01	0.9829	-0.04
CLAU_0128	LamB/YcsF family protein	0.019	4	1	1	6	255	25.79	25.60	25.55	23.67	*22.24*	25.02	25.29		1.17	0.1753	2.00
CLAU_0005	Peptidase T	0.064	6	2	2	2	408	25.40	25.16	24.63	27.84	25.43	23.74	25.28		0.19	0.7641	-0.61
CLAU_0754	hypothetical protein	0.020	2	1	1	11	320	25.06	24.20	23.38	25.48	26.97	27.51	25.27		1.46	0.1208	-2.44
CLAU_0995	Hypothetical protein	0.012	19	4	4	12	262	26.17	26.37	26.22	24.15	24.29	24.35	25.26	+	4.72	0.0046	1.99
CLAU_2433	Transcriptional regulator AraC family	0.069	7	2	2	3	283	25.87	26.62	25.91	24.64	24.54	24.22	25.26	+	2.43	0.0390	1.67
CLAU_2584	MacB-like periplasmic core domain cont	0.033	9	3	3	4	384	25.50	24.99	23.99	23.94	25.68	25.72	25.25		0.14	0.8194	-0.29
CLAU_0693	Transcription elongation factor GreA	0.012	20	3	3	10	154	25.32	25.15	27.74	27.87	24.98	23.14	25.24		0.17	0.7850	0.74
CLAU_1513	Aspartyl/glutamyl-tRNA(Asn/Gln) amidc	0.062	9	1	1	4	95	25.70	*21.14*	*22.30*	26.29	25.26	25.20	25.23		0.83	0.2916	-2.54
CLAU_0868	Heavy metal translocating P-type ATPas	0.142	1	1	1	2	721	24.00	*21.67*	26.02	24.66	26.26	25.77	25.22		0.55	0.4484	-1.67
CLAU_3541	Aspartate carbamoyltransferase	0.155	6	2	2	2	337	25.05	*20.30*	24.97	25.69	25.89	25.38	25.22		0.63	0.3920	-2.21
CLAU_0968	putative amidohydrolase	0.091	2	1	1	3	389	24.01	*21.80*	25.26	25.15	25.72	25.90	25.21		0.85	0.2845	-1.90
CLAU_0321	Ig domain protein group 2 domain prote	0.058	3	2	2	3	1008	24.88	25.19	25.65	24.84	25.85	25.20	25.20		0.05	0.9316	-0.06
CLAU_0110	putative ABC transporter	0.012	4	4	4	10	1164	25.21	24.93	25.16	25.60	26.23	23.80	25.19		0.05	0.9328	-0.11
CLAU_2023	Methyl-accepting chemotaxis sensory tr	0.006	11	8	8	17	697	*22.23*	*22.13*	*21.15*	28.93	28.46	28.10	25.17	+	4.03	0.0092	-6.66
CLAU_2383	Dihydrodipicolinate synthase	0.044	3	1	1	3	299	25.17	23.16	*21.11*	25.87	25.34	25.15	25.16		0.90	0.2625	-2.31
CLAU_1459	Acetylornithine transaminase	0.018	5	2	2	10	455	24.98	24.89	25.29	24.58	27.54	28.15	25.14		0.70	0.3571	-1.70
CLAU_0036	Transcriptional regulator GntR family wi	0.012	15	5	5	10	507	25.63	27.28	26.09	*21.66*	24.63	24.62	25.13		1.15	0.1801	2.70
CLAU_3830	Na(+) dependent nucleoside transporter	0.029	13	4	4	5	394	25.92	24.81	24.60	24.76	25.42	25.85	25.12		0.17	0.7890	-0.23
CLAU_2826	DNA repair protein recO	0.337	3	1	1	1	257	25.07	23.84	25.14	25.34	25.58	24.68	25.11		0.44	0.5262	-0.52
CLAU_2774	Acriflavin resistance protein	0.098	1	1	1	3	1013	23.92	22.47	*18.77*	26.73	26.46	26.27	25.10		1.44	0.1236	-4.77
CLAU_2588	Hypothetical protein	0.142	1	1	1	1	868	25.18	23.64	25.01	24.51	25.78	25.61	25.10		0.48	0.5023	-0.69
CLAU_0923	hypothetical protein	0.064	7	1	1	2	150	25.06	24.90	24.94	25.09	26.26	26.06	25.08		1.08	0.2025	-0.84
CLAU_0594	Anaerobic sulfite reductase subunit B	0.146	8	2	2	2	262	25.04	25.06	24.74	24.27	26.50	26.07	25.05		0.41	0.5499	-0.67
CLAU_2391	Methyl-accepting chemotaxis sensory tr	0.033	4	2	2	4	570	25.92	24.16	23.48	24.81	27.68	25.28	25.05		0.54	0.4537	-1.40
CLAU_3273	hypothetical protein	0.033	5	2	2	4	409	22.49	23.40	24.67	25.36	25.59	25.43	25.02		1.42	0.1266	-1.94
CLAU_1801	putative amidase domain-containing prc	0.145	8	2	2	2	383	*20.96*	*23.14*	25.22	25.57	25.44	24.80	25.01		0.80	0.3093	-2.17
CLAU_0870	ErfK/YbiS/YcfS/YnhG family protein	0.143	3	1	1	1	222	23.21	23.30	*22.36*	27.10	26.97	26.69	25.00	+	3.60	0.0147	-3.96
CLAU_2805	Germination protease	0.177	3	1	1	1	324	26.33	24.62	23.27	23.38	26.46	25.37	25.00		0.09	0.8843	-0.33
CLAU_0460	Hypothetical protein	0.007	11	2	2	14	277	22.71	24.89	25.72	24.66	25.42	25.04	24.97		0.26	0.6867	-0.60
CLAU_2320	Hypothetical protein	0.044	4	1	1	3	292	25.51	25.37	24.31	24.65	25.10	24.82	24.96		0.20	0.7580	0.21
CLAU_2121	Coat F domain protein	0.141	30	1	1	2	97	25.07	26.92	23.51	24.82	22.64	25.76	24.95		0.22	0.7305	0.76
CLAU_2129	Transcriptional activator AraC family	0.036	4	1	1	4	294	24.64	24.38	25.22	23.42	25.63	26.25	24.93		0.15	0.8175	-0.35
CLAU_0926	hypothetical protein	0.141	9	1	1	1	141	22.67	*20.99*	24.44	25.60	25.51	25.39	24.92		1.31	0.1453	-2.80
CLAU_2224	Ribosomal RNA small subunit methyltra	0.047	11	3	3	4	280	24.69	24.98	26.28	23.43	24.84	25.55	24.91		0.38	0.5799	0.71
CLAU_0348	putative TIM-barrel enzyme, putative	0.013	11	2	2	8	276	24.84	*21.07*	*20.25*	24.95	25.41	25.57	24.90		1.07	0.2041	-3.26
CLAU_2926	Magnesium and cobalt transport protei	0.143	3	1	1	1	315	23.95	*20.24*	24.88	25.28	24.90	25.38	24.89		0.69	0.3604	-2.16
CLAU_3788	Transcriptional regulator LysR family	0.033	4	1	1	4	287	24.88	24.99	24.56	25.20	24.89	24.86	24.89		0.44	0.5276	-0.17
CLAU_3735	ABC-type transporter periplasmic subun	0.044	4	1	1	3	346	24.71	24.49	24.90	25.31	24.87	25.19	24.89		1.13	0.1872	-0.42
CLAU_3702	Hypothetical protein	0.276	6	1	1	1	154	*20.99*	*21.23*	23.99	26.53	26.40	25.78	24.89		1.87	0.0708	-4.16
CLAU_1409	Processing peptidase	0.033	3	1	1	4	416	24.02	24.37	25.31	25.09	24.69	25.07	24.88		0.40	0.5588	-0.38

CLAU_3033	Flagellar protein export ATPase fliI	0.029	7	3	3	5	436	29.24	23.21	*21.18*	26.09	23.65	26.55	24.87		0.13	0.8451	-0.89
CLAU_0130	Sodium/proline symporter	0.033	2	1	1	4	490	24.51	24.02	24.61	25.19	25.61	25.12	24.87		1.75	0.0807	-0.93
CLAU_2271	hypothetical protein	0.146	2	1	1	1	350	23.71	25.07	25.47	25.28	24.66	*21.42*	24.87		0.30	0.6458	0.96
CLAU_0256	Membrane-flanked domain DUF304	0.023	9	1	1	5	161	24.99	25.02	24.47	23.53	24.73	25.17	24.86		0.27	0.6774	0.35
CLAU_0410	Acyl-CoA N-acyltransferase	0.049	4	1	1	3	227	22.09	24.78	24.99	25.39	24.94	24.47	24.86		0.43	0.5329	-0.98
CLAU_2821	Sporulation protein YqfD	0.108	5	2	2	3	366	26.21	25.77	23.32	23.46	24.25	25.44	24.85		0.27	0.6774	0.72
CLAU_2235	Cell wall hydrolase/autolysin	0.045	4	2	2	3	603	24.73	24.79	26.28	22.93	25.65	24.88	24.84		0.34	0.6128	0.78
CLAU_2350	MgtE intracellular region	0.033	3	1	1	4	420	25.61	*20.42*	24.12	24.66	25.39	25.00	24.83		0.45	0.5210	-1.63
CLAU_1986	DUTPase	0.019	5	1	1	6	154	25.40	25.41	24.50	24.13	24.59	25.06	24.83		0.56	0.4395	0.51
CLAU_1672	Beta-lactamase	0.323	2	1	1	1	665	24.59	25.07	25.06	25.22	23.71	24.40	24.83		0.43	0.5361	0.46
CLAU_3563	Molybdopterin-binding oxidoreductase	0.014	9	4	4	8	645	26.85	24.29	*20.89*	26.54	25.20	24.44	24.82		0.31	0.6404	-1.38
CLAU_3216	Holliday junction resolvase	0.017	7	1	1	7	137	26.35	27.09	27.67	*20.90*	*21.64*	*23.29*	24.82	+	2.50	0.0379	5.09
CLAU_3352	putative sugar:sodium symporter	0.036	3	1	1	4	463	24.69	24.85	24.90	*21.37*	24.82	24.77	24.80		0.43	0.5314	1.16
CLAU_0841	3-dehydroquinate dehydratase	0.018	10	2	2	6	270	24.10	*21.01*	24.49	25.07	25.69	25.63	24.78		0.95	0.2425	-2.26
CLAU_0287	Radical SAM protein	0.202	2	1	1	1	570	23.14	23.38	25.87	24.55	25.60	24.96	24.76		0.42	0.5435	-0.91
CLAU_1148	Sigma-54 specific transcriptional regulat	0.018	6	3	3	7	547	24.59	24.76	24.71	24.73	25.37	25.31	24.75		1.00	0.2227	-0.45
CLAU_0904	Transcriptional regulator TetR family	0.145	6	1	1	2	207	23.99	24.90	*20.41*	24.50	28.25	29.26	24.70		1.00	0.2251	-4.24
CLAU_0689	NADPH-dependent FMN reductase	0.037	13	3	3	4	215	*21.45*	23.22	24.99	24.39	25.66	25.50	24.69		0.83	0.2929	-1.96
CLAU_0191	Hypothetical protein	0.119	8	1	1	2	205	25.19	25.27	25.78	*21.78*	24.16	23.33	24.68		1.49	0.1162	2.32
CLAU_1155	Transcriptional regulator AraC family	0.022	6	1	1	7	116	25.08	24.36	22.65	24.52	25.05	24.77	24.65		0.44	0.5314	-0.75
CLAU_1273	Acyltransferase 3	0.065	3	1	1	2	326	25.59	22.78	25.21	22.86	25.77	24.07	24.64		0.09	0.8936	0.29
CLAU_2882	Pyridine nucleotide-disulfide oxidoreduc	0.317	2	1	1	1	704	24.11	21.64	24.87	24.67	24.76	24.61	24.64		0.51	0.4744	-1.14
CLAU_0322	Cell wall binding repeat 2-containing prc	0.045	3	2	1	5	654	25.59	24.84	23.73	24.43	25.00	24.08	24.64		0.13	0.8375	0.22
CLAU_1864	N-acetylmuramoyl-L-alanine amidase C\	0.262	3	1	1	1	230	24.55	*22.30*	24.72	24.35	28.05	27.03	24.64		0.90	0.2619	-2.62
CLAU_0574	Hypothetical protein	0.054	36	3	3	3	98	*22.02*	*22.15*	24.63	25.58	24.61	24.72	24.62		1.06	0.2090	-2.04
CLAU_1971	Hypothetical protein	0.150	3	1	1	2	367	25.79	25.30	24.35	24.60	24.59	24.06	24.60		0.73	0.3419	0.73
CLAU_2593	Hypothetical protein	0.023	12	2	2	5	215	24.60	22.90	25.02	24.24	25.13	24.53	24.57		0.26	0.6838	-0.46
CLAU_0893	hypothetical protein	0.228	9	1	1	1	140	23.74	*21.57*	26.95	25.60	24.92	24.21	24.57		0.20	0.7586	-0.82
CLAU_3620	Oxidoreductase	0.034	4	3	3	4	647	25.67	24.51	26.07	23.26	22.23	24.60	24.56		1.16	0.1765	2.05
CLAU_0094	Phosphate-transporting ATPase	0.037	8	2	2	4	234	23.99	23.21	22.78	26.58	26.68	25.12	24.56		1.98	0.0614	-2.80
CLAU_1400	Resolvase domain-containing protein	0.146	2	1	1	2	499	21.81	*21.39*	25.00	24.11	25.44	25.52	24.56		0.87	0.2771	-2.29
CLAU_3004	AAA-ATPase-like protein	0.064	5	2	2	2	530	24.56	24.39	24.49	25.69	24.91	24.52	24.54		0.74	0.3371	-0.56
CLAU_3570	Hypothetical protein	0.150	6	1	1	1	166	25.68	25.21	25.92	*21.48*	*21.70*	23.87	24.54		1.83	0.0729	3.26
CLAU_3293	Ribosome biogenesis GTP-binding prote	0.110	4	1	1	2	284	23.22	24.32	25.06	24.75	23.22	25.04	24.54		0.06	0.9217	-0.14
CLAU_1833	Na(+)/H(+) antiporter NhaC-like protein	0.023	4	2	2	5	485	*20.88*	*21.57*	*21.74*	27.92	27.76	27.32	24.53	+	4.40	0.0046	-6.27
CLAU_3813	Transcription activator/effector binding	0.027	14	4	4	5	279	23.48	24.84	25.62	24.22	25.59	*21.74*	24.53		0.24	0.7058	0.80
CLAU_2560	DegT/DnrJ/EryC1/StrS aminotransferase	0.275	3	1	1	1	396	24.29	24.70	25.70	23.11	24.36	26.22	24.53		0.12	0.8479	0.33
CLAU_0573	Hypothetical protein	0.033	17	3	3	5	152	24.98	25.88	23.77	24.26	24.46	24.55	24.51		0.30	0.6461	0.45
CLAU_3280	Ribonuclease 3	0.016	6	1	1	7	235	25.05	24.38	24.61	23.45	25.21	21.18	24.50		0.52	0.4689	1.40
CLAU_2029	Hypothetical protein	0.081	1	1	1	2	480	24.15	24.71	24.14	24.78	24.63	24.33	24.48		0.46	0.5111	-0.25
CLAU_2616	Appr-1-p processing domain protein	0.265	5	1	1	1	180	23.70	*22.14*	24.44	25.45	24.46	26.34	24.45		1.08	0.2032	-1.99
CLAU_2733	hypothetical protein	0.018	29	2	2	6	86	22.24	24.17	*21.33*	25.74	26.15	24.72	24.45		1.46	0.1204	-2.96
CLAU_0553	putative membrane protein	0.143	12	1	1	1	89	24.53	24.79	23.91	*21.07*	24.16	24.58	24.35		0.43	0.5343	1.14
CLAU_3689	ClpP family serine protease	0.258	4	1	1	1	265	23.16	24.55	23.29	24.78	24.60	24.14	24.35		0.81	0.3054	-0.84
CLAU_1069	Metal ABC-type transporter ATP-binding	0.054	8	1	1	3	221	24.53	24.59	24.20	24.46	23.60	23.50	24.33		0.83	0.2929	0.59
CLAU_2239	Hypothetical protein	0.315	1	1	1	1	1361	24.96	27.76	26.33	*21.53*	23.63	23.48	24.30		1.52	0.1114	3.47

CLAU_1505	Nucleoside-triphosphatase rdgB	0.007	23	4	4	13	202	23.54	*21.43*	26.37	24.54	26.63	24.03	24.29		0.32	0.6269	-1.29
CLAU_2306	Peptide chain release factor 2	0.013	10	3	3	8	331	24.37	24.81	24.19	22.98	25.04	23.68	24.28		0.37	0.5866	0.56
CLAU_1637	putative transcriptional regulator	0.045	2	1	1	3	382	23.71	23.84	23.53	25.15	24.72	24.75	24.28	+	2.69	0.0309	-1.18
CLAU_1490	Nitroreductase	0.044	4	1	1	3	199	23.31	23.18	23.24	25.42	25.59	25.23	24.27	+	4.40	0.0046	-2.17
CLAU_0654	Transcriptional regulator DeoR family	0.018	9	2	2	6	254	24.43	24.10	24.05	23.09	25.69	24.91	24.27		0.18	0.7769	-0.37
CLAU_1356	CRISPR-associated helicase Cas3	0.058	5	3	3	4	855	24.37	*20.38*	24.16	23.91	26.09	25.96	24.27		0.73	0.3419	-2.35
CLAU_3742	Cobalamin synthesis protein P47K	0.325	10	1	1	1	231	27.07	25.04	22.83	26.84	23.49	*22.27*	24.27		0.16	0.8036	0.78
CLAU_2252	Spore coat protein CotS family	0.324	5	1	1	1	333	24.33	25.79	24.45	23.14	*22.12*	24.19	24.26		1.06	0.2100	1.71
CLAU_3891	Hypothetical protein	0.064	3	1	1	2	324	22.39	*22.28*	*21.97*	26.28	26.11	26.16	24.25	+	5.09	0.0040	-3.97
CLAU_1461	Transcriptional regulator	0.148	8	2	2	2	401	*21.21*	*22.89*	*22.62*	26.25	26.30	25.61	24.25	+	2.60	0.0339	-3.81
CLAU_3817	Transcriptional regulator HxlR family	0.145	6	1	1	1	120	23.93	24.29	24.77	22.52	24.14	24.37	24.22		0.44	0.5254	0.65
CLAU_1670	ATP-binding region ATPase domain prot	0.036	7	2	2	4	353	24.36	24.45	23.34	24.27	24.13	24.11	24.20		0.12	0.8479	-0.12
CLAU_0587	Thiaminase II	0.049	5	1	1	3	219	23.48	*21.25*	22.52	24.91	26.23	25.78	24.20		1.89	0.0683	-3.22
CLAU_2181	Ribose import ATP-binding protein rbsA	0.136	2	1	1	2	499	23.03	20.81	25.51	24.29	25.55	24.06	24.18		0.46	0.5175	-1.52
CLAU_2085	Integrase family protein	0.035	5	2	2	4	391	24.18	23.14	*21.47*	24.16	24.40	24.83	24.17		0.88	0.2726	-1.53
CLAU_2482	Hypothetical protein	0.044	3	1	1	3	467	23.43	22.97	23.58	24.98	25.32	24.72	24.15	+	2.58	0.0344	-1.68
CLAU_2502	Nitrogenase iron protein	0.172	5	1	1	1	272	24.38	23.86	24.50	*22.24*	24.76	*22.98*	24.12		0.52	0.4663	0.92
CLAU_2940	Arginine deiminase	0.142	3	1	1	1	426	25.15	24.70	22.60	24.42	23.79	22.85	24.11		0.20	0.7586	0.46
CLAU_2116	Hypothetical protein	0.143	4	1	1	1	184	22.87	22.23	*21.21*	25.33	25.70	26.82	24.10	+	2.37	0.0407	-3.85
CLAU_0998	DNA modification/repair radical SAM pr	0.073	1	1	1	3	445	25.26	25.36	22.80	22.46	24.07	24.09	24.08		0.40	0.5615	0.93
CLAU_2476	Transcriptional regulator MarR family	0.216	7	1	1	1	149	23.84	24.02	24.19	25.50	23.32	24.11	24.07		0.17	0.7869	-0.29
CLAU_0329	L-2-amino-thiazoline-4-carboxylic acid	0.139	5	1	1	2	153	24.65	24.63	23.54	21.48	22.56	24.55	24.05		0.66	0.3761	1.41
CLAU_2152	NUDIX hydrolase	0.062	17	2	2	2	183	23.93	22.79	*21.33*	24.10	25.71	25.34	24.02		1.24	0.1593	-2.37
CLAU_2434	Dihydrodipicolinate synthase	0.045	3	1	1	3	293	25.05	25.48	22.21	23.94	23.16	24.07	24.01		0.19	0.7683	0.52
CLAU_1346	Flavodoxin domain-containing protein	0.023	20	3	3	5	180	24.00	*21.82*	23.04	23.98	26.58	26.56	23.99		1.21	0.1652	-2.75
CLAU_0268	Peptidase M29 aminopeptidase II	0.047	4	1	1	5	313	22.71	*20.27*	23.52	25.39	24.65	24.42	23.97		1.22	0.1619	-2.65
CLAU_1156	Integral membrane sensor signal transd	0.089	4	2	2	2	487	26.58	27.66	27.16	*21.20*	*21.31*	*20.35*	23.94	+	3.85	0.0107	6.18
CLAU_3840	Xylulokinase	0.143	2	1	1	1	506	*21.20*	*21.01*	*23.49*	24.47	25.01	24.39	23.94		1.53	0.1105	-2.72
CLAU_3028	Flagellar basal body rod protein flgC	0.254	11	1	1	1	143	23.37	*21.80*	24.83	22.06	25.23	24.48	23.93		0.17	0.7869	-0.59
CLAU_1700	Germination protein Ger(x)C family	0.102	5	1	1	2	386	*20.72*	*22.76*	*21.92*	25.30	25.08	25.40	23.92	+	2.35	0.0407	-3.46
CLAU_0315	Cell wall binding repeat 2-containing prc	0.040	3	2	1	5	629	23.58	23.74	25.26	25.08	23.73	24.08	23.91		0.05	0.9312	-0.10
CLAU_0838	Xylose isomerase domain-containing prc	0.065	9	2	2	2	290	24.46	24.42	22.87	22.74	23.59	24.09	23.84		0.27	0.6751	0.44
CLAU_1445	Drug resistance transporter Bcr/CflA sub	0.043	5	1	1	3	420	24.13	25.02	24.73	*21.51*	23.53	23.16	23.83		1.32	0.1451	1.89
CLAU_0058	Two component transcriptional regulatc	0.172	4	1	1	1	217	22.77	22.52	24.86	*20.57*	27.54	26.08	23.82		0.23	0.7154	-1.35
CLAU_0627	Threonine dehydrogenase	0.064	5	1	1	2	338	22.77	*22.31*	22.51	25.24	24.80	24.93	23.79	+	3.72	0.0129	-2.46
CLAU_2443	Efflux transporter RND family MFP subu	0.142	4	1	1	1	322	23.23	22.33	22.25	25.22	25.06	24.26	23.75	+	2.18	0.0495	-2.24
CLAU_0141	hypothetical protein	0.141	7	1	1	1	226	23.27	24.03	23.44	*20.23*	24.71	24.79	23.74		0.08	0.8994	0.34
CLAU_0618	Response regulators consisting of a Che'	0.139	8	1	1	1	227	25.31	23.03	23.25	22.79	24.59	24.19	23.72		0.00	0.9990	0.01
CLAU_0530	Transcriptional regulator PadR-like famil	0.064	8	1	1	2	172	23.79	*20.28*	23.62	23.86	23.94	22.93	23.71		0.35	0.6002	-1.01
CLAU_2401	6-pyruvoyl tetrahydropterin synthase-re	0.037	5	1	1	4	163	24.23	22.51	26.02	24.59	*23.09*	*22.11*	23.66		0.33	0.6209	0.99
CLAU_2801	Cation-transporting ATPase	0.040	4	2	2	4	862	23.37	23.80	23.81	23.61	23.63	23.48	23.62		0.22	0.7279	0.09
CLAU_3100	hypothetical protein	0.122	10	1	1	2	150	22.47	23.76	24.59	*20.67*	23.47	27.43	23.62		0.04	0.9477	-0.25
CLAU_0666	SNF2 family protein	0.073	2	2	2	3	891	26.56	*21.32*	*23.18*	24.04	24.20	23.16	23.61		0.02	0.9681	-0.11
CLAU_3139	Sporulation protein YtfJ	0.160	20	2	2	2	146	25.45	23.92	23.13	25.90	23.30	23.10	23.61		0.02	0.9720	0.07
CLAU_1264	Integral membrane protein MviN	0.112	1	1	1	2	516	*22.34*	*19.90*	22.75	24.84	25.62	24.43	23.59		1.59	0.1022	-3.30
CLAU_2768	putative transcriptional regulator PucR f	0.140	2	1	1	2	521	22.69	22.95	24.18	20.83	25.48	26.39	23.57		0.21	0.7440	-0.96

CLAU_1398	Transcriptional regulator GntR family	0.143	7	1	1	1	124	24.00	23.11	23.53	23.51	24.14	22.84	23.52		0.04	0.9525	0.05
CLAU_3468	Histidine triad (HIT) protein	0.142	7	1	1	1	141	22.86	*21.29*	23.20	23.81	24.33	23.79	23.50		1.17	0.1752	-1.53
CLAU_2123	Transcriptional regulator MarR family w	0.143	2	1	1	1	331	23.20	21.75	23.11	24.33	23.78	24.11	23.49		1.31	0.1460	-1.39
CLAU_0930	TesA-like protease	0.143	6	1	1	1	192	23.57	22.64	24.06	24.77	22.28	23.40	23.49		0.02	0.9681	-0.06
CLAU_0305	DNA mismatch repair protein MutS dor	0.023	3	1	1	5	560	22.76	23.62	23.57	*21.36*	24.96	23.33	23.45		0.03	0.9589	0.10
CLAU_2565	Mannose-1-phosphate	0.054	4	2	1	3	455	23.54	25.68	23.34	*21.03*	22.80	23.91	23.44		0.65	0.3841	1.61
CLAU_3749	Fe-only hydrogenase	0.122	1	1	1	2	457	22.44	23.82	23.65	23.40	22.93	23.44	23.42		0.03	0.9562	0.05
CLAU_1675	putative membrane protein with peptid	0.333	2	1	1	1	748	22.87	24.44	23.87	27.00	*21.26*	*21.79*	23.37		0.07	0.9082	0.38
CLAU_0664	Peptidase M42 family protein	0.028	4	1	1	7	325	24.85	24.22	26.32	*20.67*	*22.46*	22.45	23.34		1.72	0.0839	3.27
CLAU_1867	TRNA pseudouridine synthase A	0.207	2	1	1	1	244	23.58	23.43	23.36	22.96	23.30	23.21	23.33		1.17	0.1752	0.30
CLAU_3405	putative glycosyltransferase	0.064	4	1	1	2	405	*21.05*	*19.80*	*22.16*	24.42	25.41	24.74	23.29	+	2.19	0.0495	-3.85
CLAU_1715	Transcriptional regulator DeoR family	0.034	14	4	4	4	341	26.22	23.26	26.81	*20.44*	23.30	*20.59*	23.28		1.30	0.1479	3.99
CLAU_0714	Purine or other phosphorylase family 1	0.075	5	1	1	3	277	23.05	*20.04*	23.78	24.12	23.49	22.83	23.27		0.42	0.5399	-1.19
CLAU_2933	DNA topoisomerase IV subunit A	0.114	2	2	2	2	954	24.49	25.00	24.04	22.46	21.17	19.81	23.25		1.84	0.0721	3.36
CLAU_3444	hypothetical protein	0.273	6	1	1	1	144	23.51	23.86	20.37	*23.60*	*22.97*	21.47	23.24		0.03	0.9660	-0.10
CLAU_0409	Acetyl-CoA acetyltransferase	0.110	4	1	1	3	399	24.44	24.80	25.76	*20.60*	*21.90*	*22.02*	23.23	+	2.36	0.0407	3.50
CLAU_1030	Integral membrane sensor signal transd	0.086	5	2	2	2	409	22.73	*19.70*	23.71	24.34	22.12	24.47	23.22		0.49	0.4943	-1.60
CLAU_1187	Iron-sulfur-dependent L-serine dehydrat	0.143	5	1	1	1	227	*20.00*	*21.36*	22.32	25.36	25.40	24.04	23.18		2.00	0.0606	-3.71
CLAU_3224	Penicillin-binding protein transpeptidase	0.143	4	2	2	2	554	*21.70*	*21.14*	*21.16*	26.54	26.55	24.63	23.17	+	2.63	0.0333	-4.57
CLAU_0932	Hypothetical protein	0.258	3	1	1	1	382	22.97	*23.34*	22.41	23.41	22.13	23.56	23.15		0.09	0.8936	-0.13
CLAU_1284	Hypothetical protein	0.279	4	1	1	1	433	25.40	24.20	25.12	*21.61*	*22.08*	*21.42*	23.14	+	2.83	0.0283	3.20
CLAU_2533	Exopolysaccharide biosynthesis polyprei	0.143	5	1	1	1	222	23.42	24.15	23.31	*21.97*	*21.42*	22.95	23.13		1.36	0.1358	1.51
CLAU_3772	Transcriptional regulator LysR family	0.078	2	1	1	2	283	*22.22*	*21.71*	23.57	24.49	24.11	22.63	23.10		0.72	0.3484	-1.24
CLAU_0106	Penicillin-binding protein transpeptidase	0.111	1	1	1	2	881	25.05	24.94	24.86	*21.29*	*20.33*	*20.62*	23.08	+	3.88	0.0107	4.20
CLAU_1247	Transcriptional regulator	0.058	12	2	2	3	246	24.91	24.61	22.89	*20.16*	22.65	23.26	23.08		0.86	0.2788	2.11
CLAU_3819	Cell wall binding repeat 2-containing prc	0.054	4	3	3	3	747	*21.20*	*23.18*	25.31	25.64	22.68	22.95	23.07		0.13	0.8451	-0.52
CLAU_1698	Hypothetical protein	0.049	2	1	1	3	444	24.56	25.90	25.94	*21.33*	*20.96*	*21.32*	22.95	+	3.09	0.0231	4.26
CLAU_3408	putative transcriptional regulator	0.047	7	2	2	3	386	23.08	22.32	23.28	*22.55*	*22.41*	23.15	22.82		0.20	0.7586	0.19
CLAU_2646	UDP pyrophosphate phosphatase	0.035	8	2	2	4	277	*22.71*	*20.12*	*20.07*	28.22	24.90	22.92	22.81		1.16	0.1768	-4.38
CLAU_1164	Phosphomethylpyrimidine kinase	0.338	5	1	1	1	267	22.84	19.11	23.46	22.73	*20.26*	22.85	22.79		0.03	0.9594	-0.14
CLAU_3110	Stage III sporulation protein AA	0.169	2	1	1	1	308	21.63	22.71	22.30	22.96	22.69	22.95	22.70		0.93	0.2481	-0.65
CLAU_1862	putative sigma54 specific transcriptiona	0.297	1	1	1	1	580	22.40	22.10	22.58	22.80	23.52	23.76	22.69		1.45	0.1219	-1.00
CLAU_0658	Adenine deaminase	0.028	6	3	3	5	588	*21.44*	*22.54*	*20.63*	23.19	23.73	22.77	22.66		1.28	0.1512	-1.69
CLAU_0955	Adenine deaminase	0.097	4	2	2	2	595	24.24	22.87	*21.05*	23.33	22.39	22.37	22.63		0.01	0.9898	0.02
CLAU_0806	Polysaccharide deacetylase	0.201	6	1	1	1	239	*20.55*	*21.24*	21.85	23.69	23.38	23.88	22.62	+	2.43	0.0390	-2.43
CLAU_0592	TorD-like chaperone	0.255	5	1	1	1	222	24.13	23.87	22.30	*22.88*	20.93	20.95	22.59		1.00	0.2227	1.85
CLAU_0959	putative xanthine dehydrogenase	0.094	5	2	2	3	700	22.90	25.87	22.96	*22.23*	*21.40*	*21.18*	22.57		1.05	0.2122	2.31
CLAU_2056	hypothetical protein	0.141	20	1	1	1	83	24.25	25.97	25.18	*19.71*	*20.83*	*20.74*	22.54	+	2.81	0.0283	4.71
CLAU_0799	Serine phosphatase	0.037	6	3	3	4	613	24.65	24.47	22.57	*21.19*	*21.59*	22.00	22.29		1.51	0.1129	2.30
CLAU_0250	N-acyl-D-glutamate deacylase	0.242	3	1	1	1	456	*22.27*	*20.19*	*21.43*	27.41	22.29	27.47	22.28		1.14	0.1820	-4.42
CLAU_0302	Fe(3+)-transporting ATPase	0.146	3	1	1	2	354	23.86	24.73	22.24	21.12	22.23	21.34	22.24		1.20	0.1696	2.05
CLAU_3585	VanZ family protein	0.176	4	1	1	1	326	*21.13*	*20.55*	*21.83*	23.30	22.64	24.15	22.23		1.73	0.0828	-2.19
CLAU_0234	Hemerythrin HHE cation binding domair	0.348	6	1	1	1	184	*21.63*	*22.31*	*20.74*	26.49	22.64	22.13	22.22		0.69	0.3614	-2.19
CLAU_3268	ATP-dependent DNA helicase RecG	0.143	2	1	1	1	679	23.21	23.45	22.13	22.13	*21.34*	21.00	22.13		1.28	0.1512	1.44
CLAU_3389	GntR domain protein	0.348	3	1	1	1	233	*21.36*	*20.39*	*21.07*	23.31	27.16	22.21	21.79		1.01	0.2217	-3.29
CLAU_2091	Hypothetical protein	0.311	5	1	1	1	135	21.62	22.36	22.15	21.60	19.98	21.19	21.61		0.98	0.2292	1.12

CLAU_3235	Multi-copper polyphenol oxidoreductas	0.044	10	2	2	3	236	23.66	24.40	19.65	*20.07*	*21.65*	*21.27*	21.46		0.43	0.5314	1.57
CLAU_3277	50S ribosomal protein L32	0.022	39	2	2	6	61	23.18	25.29	21.80	20.89	20.89	21.06	21.43		1.15	0.1814	2.48
CLAU_3126	Arginine repressor	0.004	21	2	2	17	150	21.51	18.57	21.86	*22.89*	20.46	21.12	21.32		0.26	0.6812	-0.84
CLAU_1807	ABC-type transporter related protein	0.141	4	2	2	2	514	23.51	23.86	20.37	*19.02*	*20.93*	21.47	21.20		0.72	0.3457	2.11
CLAU_1305	50S ribosomal protein L35	0.033	15	1	1	6	65	21.22	21.61	22.11	20.62	21.00	21.17	21.20		1.11	0.1932	0.72
CLAU_0167	hypothetical protein	0.080	13	1	1	3	68	20.97	17.32	22.42	17.84	21.05	21.68	21.01		0.01	0.9891	0.05
CLAU_2447	Proprionate catabolism activator Fis fan	0.321	1	1	1	1	647	20.27	20.17	*20.84*	20.37	21.19	20.56	20.47		0.36	0.5975	-0.28
CLAU_0546	putative transcriptional regulator PucR f	0.295	2	1	1	1	521	22.54	18.65	21.11	*21.25*	*19.67*	19.49	20.39		0.19	0.7653	0.63
CLAU_2559	Polysaccharide biosynthesis protein	0.299	2	1	1	1	352	20.18	22.31	19.73	*21.64*	20.35	19.36	20.27		0.10	0.8754	0.29
CLAU_1593	putative secreted protein	0.058	3	1	1	4	484	19.90	19.96	19.76	19.53	18.68	18.99	19.65		1.47	0.1202	0.81
CLAU_2561	Cytidylyltransferase family protein	0.300	3	1	1	1	394	21.06	19.68	20.23	19.48	19.22	19.54	19.61		1.03	0.2170	0.91

[a]False-discovery rate (proteome filtering via Percolator)

[b]Peptide sequence coverage percentage per specific protein

[c]Number of distinct peptide sequences identified, includes shared peptides

[d]Number of peptides uniquely mapping to each protein, non-shared peptides

[e]Peptide spectrum matches (raw number of MS/MS spectra collected for all peptides of a given protein)

[f]Protein abundance in Log2 units

[g]Median log2 abundance across all six samples

[h]Benjamini-Hochberg determined significance (significant = '+')

[i]p-value comparing Lag samples vs. log samples, expressed in -log10 of the pvalue.

[j]Benjamini-Hochberg corrected p-value, assessed at an FDR of < 0.05.
