# Supplementary material for: Clostridium autoethanogenum isopropanol production via native plasmid pCA replicon
Source: Front Bioeng Biotechnol. 2022 Aug 5;10:932363. doi: 10.3389/fbioe.2022.932363 (PMC9413188; doi:10.3389/fbioe.2022.932363)
Supplement: Supplementary file 4 [file DataSheet2.docx]

LOCUS pCA1 5499 bp dna circular UNK 17-JUN-2022

DEFINITION .

ACCESSION Native_Plasmid

KEYWORDS .

SOURCE null

ORGANISM .

FEATURES Location/Qualifiers

rep_origin 1..22

/created_by="User"

/modified_by="User"

/label="DSO"

source <1..3

/organism="Clostridium Native Plasmid"

/db_xref="taxon:1485"

/mol_type="genomic DNA"

/note="1485.252"

source 4..>5499

/organism="Clostridium Native Plasmid"

/db_xref="taxon:1485"

/mol_type="genomic DNA"

/note="1485.252"

repeat_region 120..128

/%_Identity=100

/Motif="aaaaaaccc"

/annotation_group="New_P_3: 120 -> 128"

/modified_by="User"

/label="IR_3"

repeat_region complement(169..177)

/%_Identity=100

/Motif="aaaaaaccc"

/annotation_group="New_P_3: 169 <- 177"

/modified_by="User"

/label="IR_3"

CDS 221..1252

/translation="MYVNNNKDIRKNQMEVLTDKTQKGEERPWRENKLKTLSLAKSYE

RIGNTRKADRVKNCSSQLIYKKDKNTGIKKLHSMISCQVRLCPMCAWRRSLKIFGQTS

KIMDKALENKEYRFIFLTLTCRNVEGKELSKIIDNLFYAFKKMMLKTKVKQIVKGWFR

ALEVTHNLDKKSKDYNTYHPHFHVILMVNKSYFTDTKQYLSQKDWTSLWKDCLRVDYM

PIVNIKAFKTNTKKEVEKSVAESAKYTVKDNDYLIVNDEKMTDETVSILDGALANRRL

VAFGGELKKIHKSLNLSDIEKASVNTDSDDDVVLRDDVNYVFEVYNWNIGYNNGQYLK

VKEIDKKEN"

/note="rasttk_feature_creation_tool=prodigal"

/note="rasttk_feature_annotation_tool=annotate_proteins_si

milarity"

/transl_table=11

/db_xref="RAST2:fig|1485.252.peg.1"

/product="replication protein"

/modified_by="User"

/modified_by="Steve.Brown"

/label="CAETHG_05090; rep"

DNA_probe_bind 483..2188

/created_by="Steve.Brown"

/modified_by="User"

/label="PCR product 3 (1,706 bp)"

CDS 1255..1515

/product="hypothetical protein"

/note="rasttk_feature_creation_tool=prodigal"

/note="rasttk_feature_annotation_tool=vr1087@patricbrc.org

"

/transl_table=11

/translation="MSCNVKECEEGSNVIYCPNRFTFDEMCNIKMSLCLAPQVIKGFI

ESVEDYESLSKEEKAMFDNGKSLIKQMGKLEKKVNRLVGYEK"

/db_xref="RAST2:fig|1485.252.peg.2"

/modified_by="User"

/modified_by="Steve.Brown"

/label="CAETHG_05095"

CDS complement(1710..1964)

/db_xref="RAST2:fig|1485.252.peg.3"

/translation="MSKKTIDVVKEYLRDRVIDDKNEHGGLYDNTKIKHLVEIVHHTD

EDLAICMENELVQDIYNIAIEFYDIGFKDGKHLLRELLKD"

/transl_table=11

/note="rasttk_feature_creation_tool=prodigal"

/note="rasttk_feature_annotation_tool=vr1087@patricbrc.org

"

/product="hypothetical protein"

/modified_by="User"

/modified_by="Steve.Brown"

/label="CAETHG_05100"

CDS complement(2079..2927)

/translation="MGNLLNQYLTSCAQSVLSGSFDLAKKLIFTPQGLPSFANTLYNI

FLGIGATLMTVIVGAKLVNLMFDISNNCADYSVGELITRTIKSSAMIVICPFLIKIVV

GEIAFPLGNWIFSQMSANASTLLDGYVKSSGISSISTGFFLFLIVGFVAASCFCFFLK

MCVYQVDLIFLQIYSVPVSISMISDNFNYMDTWWRELISQVVTIITQLICMLGITWAL

SNKFTWYNFMILFGCCINLIKGPSFLRDMWYSTGSGRNAMHMGSKIATRVMMIKNLGG

RSYLQD"

/note="rasttk_feature_creation_tool=prodigal"

/note="rasttk_feature_annotation_tool=vr1087@patricbrc.org

"

/transl_table=11

/db_xref="RAST2:fig|1485.252.peg.4"

/product="hypothetical protein"

/modified_by="User"

/modified_by="Steve.Brown"

/label="CAETHG_05105; traL-like"

DNA_probe_bind 2162..3069

/created_by="Steve.Brown"

/Sequence="TTCTTCCTGAGCCAGTCGAATACCACATATCACGTAAAAAGCTTGGA

CCTTTAATTAGATTAATACAACATCCAAATAAAATCATGAAATTATACCAGGTGAATT

TGTTACTTAAAGCCCATGTAATACCCAACATACATATCAATTGAGTTATTATGGTTAC

TACTTGACTTATAAGCTCTCTCCACCATGTATCCATGTAATTAAAGTTATCTGAAATC

ATTGAAATGGATACTGGAACTGAATATATCTGTAAAAATATTAAATCTACCTGATAAA

CACACATTTTTAGAAAAAAACAAAAGCATGAAGCTGCAACAAAACCAACTATTAAGAA

TAAGAAAAAACCTGTAGATATTGAACTAATACCACTAGATTTCACATAACCATCGAGT

AAGGTTGATGCATTTGCAGACATTTGGCTAAAAATCCAATTACCTAAAGGAAACGCAA

TTTCTCCAACTACAATTTTTATAAGAAACGGGCATATAACTATCATTGCACTTGATTT

TATAGTTCTAGTTATTAATTCACCCACTGAATAATCAGCACAGTTATTACTTATATCA

AACATTAAATTTACAAGTTTAGCACCAACTATTACAGTCATTAAGGTTGCTCCAATAC

CTAAAAATATATTGTATAAAGTATTAGCGAAGCTAGGTAAGCCTTGGGGTGTAAAAAT

CAATTTTTTTGCTAAGTCAAATGAGCCACTTAAGACACTTTGAGCACAAGATGTAAGA

TATTGATTTAATAAATTACCCATAAAAAAACTTCCTTTCTATTGAACTTTATTGATTT

TCCATTTACTATCTTCTTGCTTCATCTGCACTTTATAAAACAATAAATTGTCTGAATT

AATTTCATAAGTAACTGTCTTGTCATCTTTGTTAACTACCGGCGTGTCA"

/Tm="81.0"

/%GC="30.3"

/modified_by="User"

/label="PCR product 5 (908 bp)"

DNA_probe_bind 2169..3368

/created_by="Steve.Brown"

/modified_by="User"

/label="PCR product 2 (1,200 bp)"

CDS complement(2943..3425)

/note="rasttk_feature_creation_tool=prodigal"

/note="rasttk_feature_annotation_tool=vr1087@patricbrc.org

"

/transl_table=11

/translation="MKKVLMIFTSMVLLIFFITGCSGFNETTAKTDSKQALQQFFKTH

KNDEVLVSNDQNREKVKEYVNTNFKDYFTKDFLTTTTNYIENGLSMNPDIFYLNDAVS

NNNAISNKITFKNDFKIDTPVVNKDDKTVTYEINSDNLLFYKVQMKQEDSKWKINKVQ

"

/db_xref="RAST2:fig|1485.252.peg.5"

/product="hypothetical protein"

/modified_by="User"

/modified_by="Steve.Brown"

/label="CAETHG_05110"

DNA_probe_bind 3050..4551

/created_by="Steve.Brown"

/modified_by="User"

/label="PCR product 4 (1,502 bp)"

primer_bind 3349..3368

/Mismatches=0

/created_by="primer3"

/annotation_group="6038086843035009e61cd59-f8d7-4d2f-be40-

c2f12e876d16"

/%GC="50.0"

/Tm="59.1"

/Hairpin_Tm="None"

/Self_Dimer_Tm="None"

/Pair_Dimer_Tm="None"

/Sequence="TCGTTAAAACCACTGCAGCC"

/Product_Size=1807

/label="P1_3349"

DNA_probe_bind 3349..5155

/created_by="User"

/label="PCR Product 1 (1,807bp)"

DNA_probe_bind 3580..5230

/created_by="Steve.Brown"

/modified_by="User"

/label="PCR product 6 (1,651 bp)"

regulatory 3700..3741

/created_by="User"

/modified_by="User"

/label="OriT"

CDS 3769..4875

/product="hypothetical protein"

/db_xref="RAST2:fig|1485.252.peg.6"

/transl_table=11

/note="rasttk_feature_creation_tool=prodigal"

/note="rasttk_feature_annotation_tool=annotate_proteins_km

er_v2"

/translation="MSFLVCHIEKYKRGNLYGLQKHEQRENKNYSNKSVDLQRTKFNY

DLANNKKINYLNRADEIINSQRVSKKAVRKDAVIAIGVIVSSDKDFFDKLDKTKQDKF

FRDSLDYFKENFSDKNIISANIHLDESTPHMHLNFVPMTSDGCLSAKKVITKAKLREL

QRGLPAYLKTKGFDIERGVENNQAKHIEITELKNQTFKELSKEYDVKLNALKKAIDVS

KNDEKILSDLKSIKTKKSILGGNISLNEGDYNKIVDLASRGLAREKEISNLKEKIKAL

ENTNCELKNKNSSLNFDKNNLNKRVDELKELVKVAESKHKKVSDSLHLATRFIKSVQV

TLNEHDLMNEAKKLFEKDELERNRKLNRNKNIGL"

/modified_by="User"

/modified_by="Steve.Brown"

/label="CAETHG_05115; mob-like"

DNA_probe_bind complement(4533..1367)

/created_by="Steve.Brown"

/modified_by="User"

/label="PCR product 7 (2,334 bp)"

CDS 5024..5449

/note="rasttk_feature_creation_tool=prodigal"

/note="rasttk_feature_annotation_tool=vr1087@patricbrc.org

"

/transl_table=11

/translation="MDAAEYKNLSIDKRVEYLNSELKNGNSSSYIFTKILGISKSQAS

IIKKNGYVLKDNQYIKAEDCPVSEPKTETKTKSVGKSVGRPPSDTEKTSANLILNKRE

YKIMQVYALLHDTNVSEIVNNFIIKFVKDENLDLNIYKK"

/db_xref="RAST2:fig|1485.252.peg.7"

/product="hypothetical protein"

/modified_by="User"

/modified_by="Steve.Brown"

/label="CAETHG_05120"

misc_feature complement(5136..5155)

/modified_by="User"

/Mismatches=0

/created_by="primer3"

/annotation_group="6038086843035009e61cd59-f8d7-4d2f-be40-

c2f12e876d16"

/%GC="50.0"

/Tm="59.0"

/Hairpin_Tm="None"

/Self_Dimer_Tm="17.6"

/Pair_Dimer_Tm="None"

/Sequence="ACTGGCCTGACTCTTCGAAA"

/Product_Size=1807

/label="P1_5155"

/note="Geneious type: primer_bind_reverse"

repeat_region 5441..5450

/%_Identity=100

/Motif="aaaaaatagg"

/annotation_group="New_P1: 5,441 -> 5,450"

/modified_by="User"

/label="IR_1"

repeat_region complement(5464..5473)

/%_Identity=100

/Motif="aaaaaatagg"

/annotation_group="New_P1: 5,464 <- 5,473"

/modified_by="User"

/label="IR_1"

repeat_region 5475..5484

/%_Identity=100

/Motif="acgatttttt"

/annotation_group="New_P_2: 5,475 -> 5,484"

/modified_by="User"

/label="IR_2"

repeat_region complement(5489..5498)

/%_Identity=100

/Motif="acgatttttt"

/annotation_group="New_P_2: 5,489 <- 5,498"

/modified_by="User"

/label="IR_2"

ORIGIN

1 ttctttctta acttgatact atgtgaacaa cagcaaaata attttatgta caataaacct

61 tttaacatca atacttcaga gggtgttttt tgtatctttt ttagcatatt attgacataa

121 aaaaacccca atgttataat ttagttgtcc aatccaaaat ttaacacagg gttttttatc

181 ttccgtaagg tttgaaccct aacgaaagga agttttttaa ttgtatgtta ataataacaa

241 agatataaga aaaaatcaaa tggaagtttt aactgataaa acccaaaaag gtgaagaaag

301 accttggagg gaaaataaat taaaaacttt atcacttgct aaaagttatg aaagaatcgg

361 taatactcgt aaagctgata gagttaagaa ttgcagtagt cagttgattt ataaaaaaga

421 caaaaataca ggtataaaga agttacatag tatgatttcg tgtcaggtga ggttatgtcc

481 aatgtgtgca tggagaaggt cattaaaaat attcggtcaa acatctaaaa ttatggataa

541 agctttagaa aataaagaat ataggtttat atttttaact ttaacttgta gaaatgtaga

601 aggtaaagaa ttgtccaaaa ttattgataa tttgttctat gcatttaaaa aaatgatgct

661 aaaaacgaaa gtaaagcaaa ttgtcaaagg ttggtttaga gcattggaag taactcataa

721 tttagataaa aaatcaaagg attataatac atatcatcca catttccatg taatacttat

781 ggtcaataaa agctacttta cagatacaaa gcagtattta tcacagaaag attggacaag

841 cttatggaaa gattgtttaa gggttgatta tatgcctata gttaacataa aagcttttaa

901 aactaatact aaaaaagaag tagaaaagtc tgttgcagaa tcagcaaaat atacagtaaa

961 agacaatgat tacttaattg taaatgatga aaaaatgaca gatgaaactg tatcaatact

1021 tgatggagca ttggcaaata ggaggcttgt tgcatttggt ggtgaattaa aaaaaataca

1081 taaatcttta aatcttagtg atatagagaa agctagtgta aatactgata gtgatgatga

1141 tgttgtactt agagatgacg ttaattatgt atttgaagtt tataattgga acattggtta

1201 taacaatggt cagtatttaa aagttaaaga aatagataaa aaggagaatt aaaagtgagt

1261 tgtaatgtta aagaatgtga agaaggttca aacgttatat attgtcctaa taggtttaca

1321 tttgatgaaa tgtgtaatat aaaaatgtca ttatgtttag caccgcaagt aattaaaggt

1381 tttattgaat ctgttgaaga ttacgaatcc ttaagtaaag aagaaaaagc tatgtttgat

1441 aatggaaaaa gtcttattaa gcaaatgggt aaattagaaa agaaagttaa tcgtttagtt

1501 ggatatgaaa aatgagaatg aaacataaaa acgcaccatt aacattacat ttttatgtta

1561 atgatgcgtt tttgttagtt ctttgcaatt ataaaattct ggtgttttat ctctagtatt

1621 actcatgtaa ttatatcata tcttcatttt tacataagta gaaagacaca ccattaacac

1681 gatattttcg tgttgatggt gtgtctttgt tagtctttta ataactctct aagtaagtgt

1741 tttccgtctt taaagcctat atcataaaat tctatagcta tattataaat atcttgtact

1801 aattcatttt ccatacaaat agcaaggtct tcatccgtat gatgtactat ctcaactaaa

1861 tgtttaatct tagtattatc gtataatcca ccatgctcat ttttatcatc aataaccctg

1921 tctcttaaat attcctttac aacatcaatt gtttttttac tcattttttc atacctccaa

1981 aatttataaa ttgacttaaa caattcctga aggtaaacta ttcttgtgtg tggatggttc

2041 cttcgggaac tgtctttcat ctttttttga cataattttt aatcttgtaa ataacttctc

2101 ccgcctaaat tttttatcat cattactctt gtggctattt tgctacccat atgcattgca

2161 tttcttcctg agccagtcga ataccacata tcacgtaaaa agcttggacc tttaattaga

2221 ttaatacaac atccaaataa aatcatgaaa ttataccagg tgaatttgtt acttaaagcc

2281 catgtaatac ccaacataca tatcaattga gttattatgg ttactacttg acttataagc

2341 tctctccacc atgtatccat gtaattaaag ttatctgaaa tcattgaaat ggatactgga

2401 actgaatata tctgtaaaaa tattaaatct acctgataaa cacacatttt tagaaaaaaa

2461 caaaagcatg aagctgcaac aaaaccaact attaagaata agaaaaaacc tgtagatatt

2521 gaactaatac cactagattt cacataacca tcgagtaagg ttgatgcatt tgcagacatt

2581 tggctaaaaa tccaattacc taaaggaaac gcaatttctc caactacaat ttttataaga

2641 aacgggcata taactatcat tgcacttgat tttatagttc tagttattaa ttcacccact

2701 gaataatcag cacagttatt acttatatca aacattaaat ttacaagttt agcaccaact

2761 attacagtca ttaaggttgc tccaatacct aaaaatatat tgtataaagt attagcgaag

2821 ctaggtaagc cttggggtgt aaaaatcaat ttttttgcta agtcaaatga gccacttaag

2881 acactttgag cacaagatgt aagatattga tttaataaat tacccataaa aaaacttcct

2941 ttctattgaa ctttattgat tttccattta ctatcttctt gcttcatctg cactttataa

3001 aacaataaat tgtctgaatt aatttcataa gtaactgtct tgtcatcttt gttaactacc

3061 ggcgtgtcaa ttttaaaatc atttttaaat gtaatcttat ttgaaatagc attgttattt

3121 gaaacagcgt catttaaata aaaaatatct ggattcatgg aaagaccatt ttcaatataa

3181 tttgttgtag tagttaaaaa atcttttgta aaataatctt tgaagtttgt gtttacatac

3241 tctttcacct tctctctatt ctggtcatta cttacaagta cctcatcatt tttatgagtt

3301 ttaaaaaatt gttgtaatgc ttgtttactg tctgtttttg cagttgtttc gttaaaacca

3361 ctgcagccag taataaaaaa gattagtaaa accatacttg taaaaatcat taaaactttt

3421 ttcataaaat cactccttta attctcttaa tataaaattt gttttttccc tttaatcact

3481 ctacatcaag agcaaaataa aacgtcaagg gcagagaaaa aaatatttta atgatccgga

3541 tttttatatc cgcagcatta aaatattttt ttctctggcg gagcgaaccc ttgacatttt

3601 atttgccttg ataaaatttt gtgataggga aaaaggaatt taaaattgtt gtttatgatt

3661 ttctttttag ggagttaagg gggaacgcct tacataggaa ctttatttgt aaagtatact

3721 atgctatgct ttacataaaa gcatttataa aaaacgaggt ggtttatcgt gagtttttta

3781 gtgtgtcata tagagaaata taagcgtgga aatttatatg gattgcaaaa acatgagcag

3841 agggaaaata aaaattacag caataaatcc gtagatttac agcgtacaaa atttaattat

3901 gatttggcta ataataaaaa aattaattat ttaaatagag cagatgaaat tataaattct

3961 caaagggtat ctaaaaaagc agtcagaaaa gatgcagtta ttgcaatagg cgttattgta

4021 tctagcgata aagatttctt tgataagctt gataaaacta aacaggataa attttttaga

4081 gatagccttg attacttcaa agagaatttt agtgataaaa atataataag tgctaacatc

4141 catttggatg aatccactcc acatatgcac ttaaatttcg ttcctatgac ttctgatggc

4201 tgtttaagtg ctaagaaggt aattactaaa gcaaaactta gagagcttca gagagggctt

4261 cctgcttatt taaagactaa agggtttgat attgaaagag gtgttgaaaa taatcaagcc

4321 aagcatatag agataactga attgaaaaat caaactttta aggaattgtc aaaagagtat

4381 gatgttaaat taaacgccct taaaaaagcc atagatgtgt ctaaaaatga tgaaaagatt

4441 ttaagtgacc taaaatccat taaaactaaa aaatcaattc taggtggcaa tataagctta

4501 aatgagggtg attataataa aatcgttgat ttagcttcac gtggacttgc tagagaaaag

4561 gaaattagta atttaaaaga aaaaattaaa gctttggaaa atactaattg tgaattgaaa

4621 aacaaaaatt caagccttaa ctttgataaa aataatttaa ataaacgtgt tgatgaatta

4681 aaggagcttg ttaaagttgc tgaaagtaaa cataaaaagg tttctgacag tttacatctt

4741 gcaactagat ttattaaatc agtacaggtt actttaaacg aacacgattt gatgaatgaa

4801 gcaaagaaat tatttgaaaa agatgaactg gaacgcaata gaaagttaaa cagaaataaa

4861 aatattggtt tatagcaaaa ggtgtgcttt tataatttta tgagtataaa actacaaaag

4921 cacaccttta ttttattatg tattgattta aaattttatt tattataaca tataagtaaa

4981 taaatggtaa taaatatatt gttatatagg ggtgtgttta taaatggatg ctgctgaata

5041 taaaaattta agcattgata agagggttga atatttaaat tctgaactta aaaatggcaa

5101 tagctcatct tatattttta ctaaaatcct tggaatttcg aagagtcagg ccagtattat

5161 taaaaagaat ggttatgtac ttaaagataa tcaatatatc aaagccgagg attgtcctgt

5221 ttccgagcct aaaaccgaaa ctaaaactaa aagtgtcggc aagagtgtcg ggcgtcctcc

5281 atcagatacg gaaaagacaa gtgctaattt aattttgaat aaaagagagt ataaaatcat

5341 gcaagtgtat gctcttttac atgatacgaa tgtaagtgaa attgtaaata attttattat

5401 caagtttgtt aaggacgaaa atttagattt aaatatttat aaaaaatagg ctttgaaaaa

5461 agccctattt ttttacgatt ttttggctaa aaaatcgtg

//
